# Stereoselective organocatalyzed glycosylations – thiouracil, thioureas and monothiophthalimide act as Brønsted acid catalysts at low loadings†
†Electronic supplementary information (ESI) available: Detailed experimental procedures, characterisation and copies of NMR spectra. See DOI: 10.1039/c8sc02788a


**DOI:** 10.1039/c8sc02788a

**Published:** 2018-10-22

**Authors:** G. A. Bradshaw, A. C. Colgan, N. P. Allen, I. Pongener, M. B. Boland, Y. Ortin, E. M. McGarrigle

**Affiliations:** a Centre for Synthesis & Chemical Biology , UCD School of Chemistry , University College Dublin , Belfield , Dublin 4 , Ireland . Email: eoghan.mcgarrigle@ucd.ie

## Abstract

Thiouracil catalyzes stereoselective glycosylations with galactals in loadings as low as 0.1 mol%. It is proposed that in these glycosylations thiouracil, monothiophthalimide, and the previously reported catalyst, Schreiner's thiourea, do not operate *via* a double H-bonding mechanism but rather by Brønsted acid/base catalysis. In addition to the synthesis of 2-deoxyglycosides and glycoconjugates, we report the first organocatalytic synthesis of 1,1′-linked trehalose-type sugars.

## Abstract 2

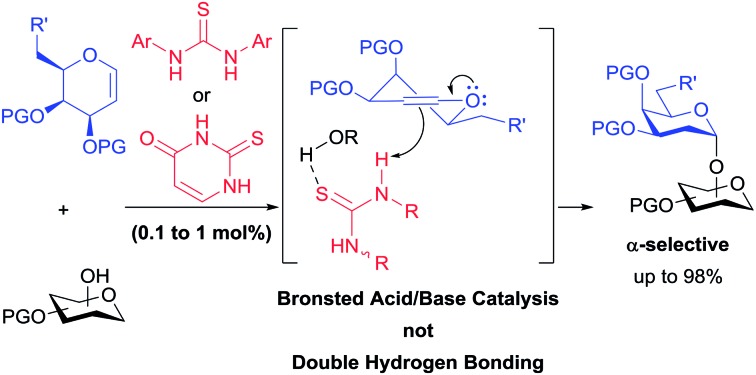
Thiouracil catalyzes stereoselective glycosylations with galactals in loadings as low as 0.1 mol%.

## Introduction

**Fig. 1:**
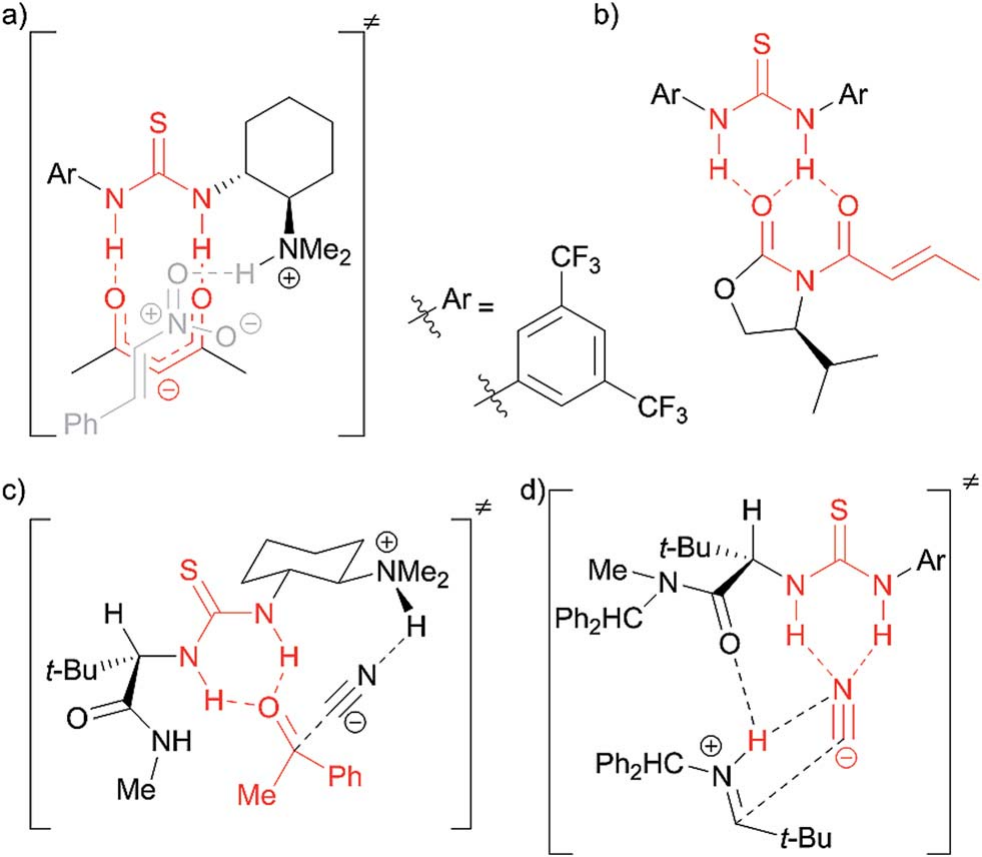
Selected examples of proposed interactions in thiourea catalysis *via* double H-bonding: (a) enolate binding in Pápai's model for enolate addition to nitroalkenes using Takemoto's catalyst;1d,e (b) proposed complex between Schreiner's catalyst and an *N*-acyloxazolidinone in Diels–Alder reaction;1*f* (c) ketone binding in Jacobsen's ketone cyanosilylation;1*g* (d) cyanide binding in Jacobsen's enantioselective Strecker reaction.1*h*

Thioureas have found many applications as organocatalysts and it has become generally accepted that they activate their substrates through double-hydrogen bonding.^1^ Prominent examples include hydrogen bonding to anions and carbonyl groups, thus creating more reactive dienophiles, electrophiles or nucleophiles (Fig. 1). Herein we present evidence that thioureas can activate molecules in at least one other way and describe how this opens the way for new organocatalyst designs, with specific applications in glycosylations detailed.^2^

**Scheme 1:**
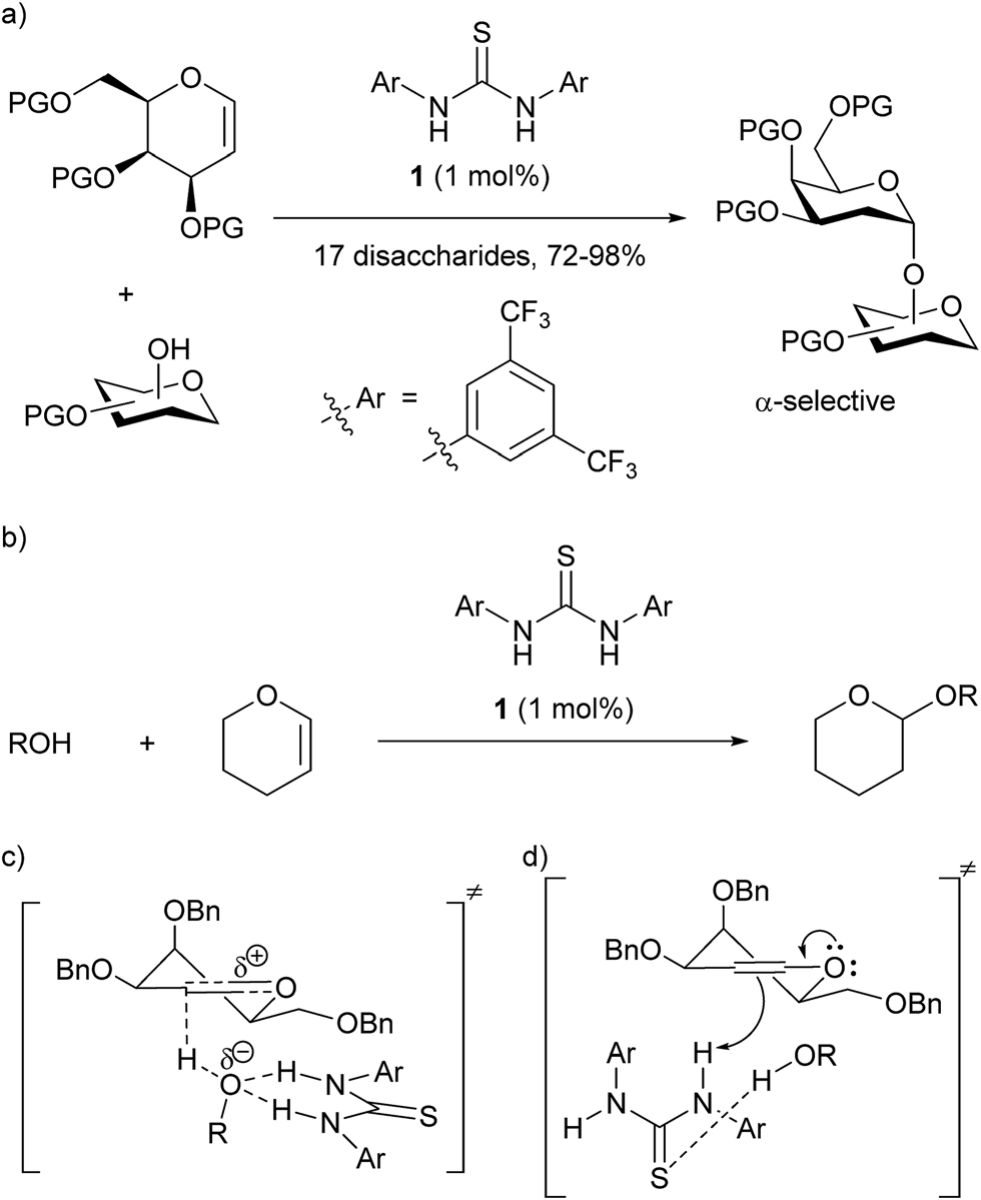
(a) Galan and McGarrigle's thiourea-catalyzed glycosylation reaction;^3^ (b) Schreiner's THP-protection of alcohols;^6^ (c) original proposal with catalyst acting as a double hydrogen-bond donor to alcohol;^3^ (d) analogue of Pápai^2^ proposal with catalyst acting as a Brønsted acid and accepting a H-bond from the alcohol.

In 2012 Galan, McGarrigle and co-workers reported^3^ that use of Schreiner's thiourea1f,^4^ **1** as a catalyst gave high α-selectivity in glycosylations with galactals to form 2-deoxydisaccharides (Scheme 1a).^5^ Based on experimental evidence, and calculations by Schreiner^6^ on the related THP-protection of alcohols (Scheme 1b), an ‘oxyanion hole’-type activation was previously proposed whereby the catalyst activated the alcohol by forming two hydrogen bonds to the oxygen, making the alcohol proton more acidic and thus catalysing C–H bond formation to C-2 of the galactal (Scheme 1c). A re-examination of this mechanistic proposal was prompted by recent work on the THP-protection of alcohols by Varga, Pápai and co-workers suggesting a Brønsted acid mode of action (see below),^2^ challenging the prevailing notion of thiourea catalysts acting as double H-bond donors. We now report the development of a new glycosylation catalyst that is >600-fold cheaper than **1** and is active at loadings as low as 0.1 mol% in the synthesis of disaccharides, glycoconjugates, and trehalose-type sugars. Our results support a Brønsted acid/base mechanism for our new catalysts *and* for Schreiner's thiourea in these glycosylations.

## Results and discussion

In contrast to the generally accepted mode of action for thiourea catalysts as H-bond donors, calculations by Varga, Pápai and co-workers favoured a Brønsted acid mode of action in the THP-protection of alcohols.^2^ An analogous transition state for our glycosylation reaction is represented in Scheme 1d. In contrast to Scheme 1c, where the N–H bonds need to be in a parallel orientation so as to enable the archetypal double-H-bonding, the alternative proposed transition state (Scheme 1d) doesn't require this parallel orientation. If such a mode of action were operative in our glycosylations, alternative catalysts designs would be feasible.

To test this new hypothesis, we first examined whether 2-thiouracil **2** was capable of catalysing the reaction between selected galactals **3**^7^ and diacetone galactose **4** (Table 1). Indeed, using just 1 mol% of thiouracil we found that high yields of disaccharides could be obtained with excellent α-selectivity.^7^ As 2-thiouracil is >600 times cheaper than Schreiner's catalyst, this is a significant improvement on our previously reported system.^8^ In fact not all of the thiouracil dissolves at this loading but it wasn't convenient to weigh out smaller amounts of catalyst, as on a *ca.* 0.5 mmol scale only 0.5 mg catalyst was used; we estimated the amount of dissolved catalyst to be <0.25 mol% under these conditions (see ESI†). In fact on larger scale the catalyst loading could be lowered further (*vide infra*). Galactals **3a–c** worked well, as they had with thiourea **1** as catalyst. Galactal **3d** was sluggish but gave a good yield when the reaction was carried out in 2-methyl-THF which allowed a higher reaction temperature.^9^ Per-acetylated **3e** was relatively unreactive, as had been observed with **1**. Per-benzylated glucal **3f** had been problematic previously, so the yield of up to 46% represented progress.^3^ We didn't pursue this further due to the formation of Ferrier-rearrangement products which were inseparable from the desired glycoside.^10^ Rhamnal **3g** also gave an inseparable mixture of Ferrier products and desired disaccharide (Entry 10). McGarrigle, Galan and co-workers previously reported a protecting group strategy for accessing α-linked disaccharides from glucal and rhamnal.10*a* Fucal **3h** gave a yield of 52% of α-**5h** (entry 11) although in this case a small amount of β-**5h** was also produced. We propose that the greater reactivity of galactals might be due to electrostatic stabilisation of the oxacarbenium ion by the 4-substituent.^11^ These results are consistent with a common mechanism being operative in thiouracil and thiourea-catalyzed glycosylations.

**Table 1:** Thiouracil-catalyzed glycosylations, exploring glycal scope^*a*^

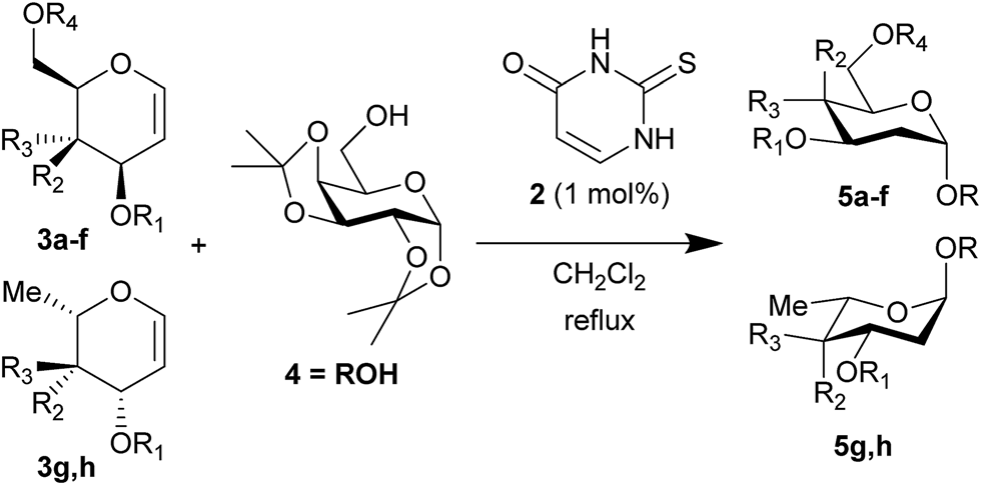								
Entry		R_1_	R_2_	R_3_	R_4_	*t* (h)	Yield (%)^*b*^	α/β^*c*^
1	**a**	Bn	OBn	H	Bn	18	95	α
2	**b**	Allyl	OAllyl	“	Allyl	18	82	α
3	**c**	Bn	OBn	“	Ac	18	85	α
4	**d**	TBS	OTBS	“	TBS	48	11	α
5		“	“	“	“	18	68^*d*^	α
6	**e**	Ac	OAc	“	Ac	18	0	—
7		“	“	“	“	18	7^*c*^ ^,^^*d*^ ^,^^*e*^	β
8	**f**	Bn	H	OBn	Bn	18	46^*e*^	5/1
9		“	“	“	“	18	39^*d*^ ^,^^*e*^	4/1
10	**g**	Bn	“	OBn	—	40	40^*e*^	4/1
11	**h**	Bn	OBn	H	—	18	52^*f*^	α (9/1)^*g*^

^*a*^1.2 equiv. of the glycal was used.

^*b*^Yield of Isolated product.

^*c*^Determined by ^1^H NMR.

^*d*^Solvent: 2-methyl-THF (reflux = 83 °C).

^*e*^Isolated with 10–20% inseparable impurities due to Ferrier rearrangement (see ESI for details).

^*f*^Isolated with *ca.* 10% hydration impurity (*vide infra*).

^*g*^Ratio prior to chromatography in parentheses.

Table 2 shows the scope of the reaction with respect to the acceptor.^12^ The reaction tolerates primary and secondary alcohols at any position on the ring (entries 1–4). In Schreiner's original report^6^ on thiourea-catalyzed THP-protection, it was noted that an amine-containing solid support was not compatible with the reaction. Later studies establishing the p*K*_a_ of **1** as 8.5 (DMSO) suggests that this might be due to deprotonation of the catalyst by amines.13a,b Catalyst **2** has a p*K*_a_ = 7.75 (H_2_O).13*c* Thus we predicted that appropriate protection group choice might enable us to use amino-containing substrates. Indeed, Boc-protected amino acids **6e–g** were glycosylated in excellent yields (entries 5–7). Glycoconjugates **7e** and **7f** thus give access to the 2-deoxy analogues of mucin-type motifs. Entry 7 shows that the reaction also tolerates phenols (although rare, glycosylated tyrosines have been found in nature).^14^ Finally, cholesterol also proved a viable substrate (entry 8), opening the way to glycoconjugate synthesis beyond amino acids.

**Table 2:** Thiouracil-catalyzed glycosylations, exploring acceptor scope^*a*^

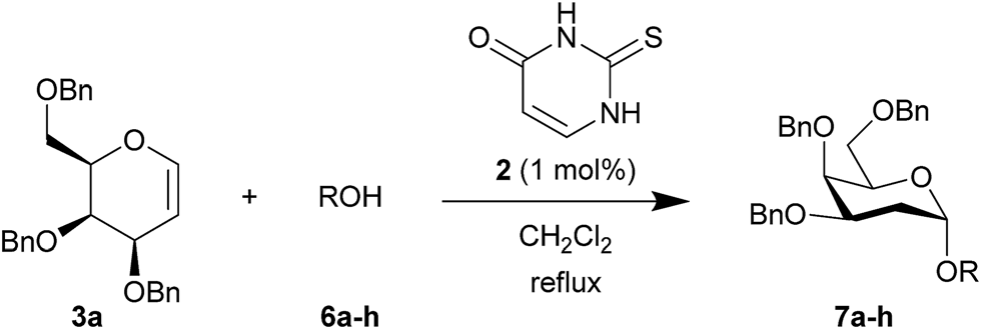					
	Yield^*b*^ (%), α/β^*c*^			Yield^*b*^ (%), α/β^*c*^	
1	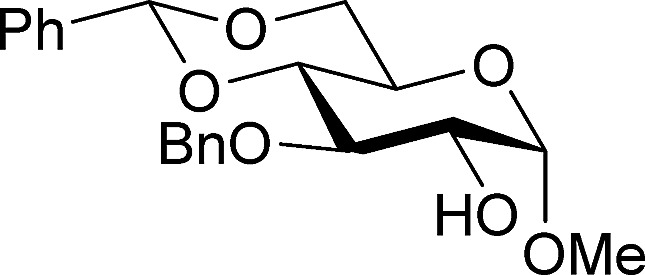	**a**, 84%,^*d*^ ^,^^*e*^ α	5	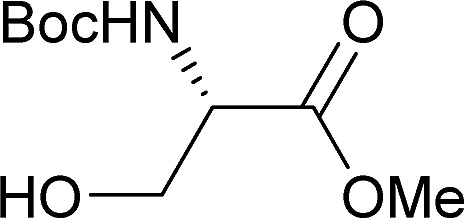	**e**, 94%, α
2	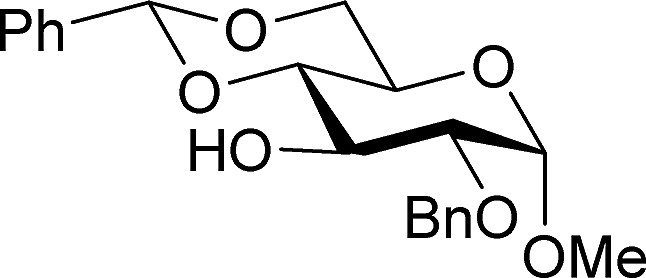	**b**, 89%, α	6	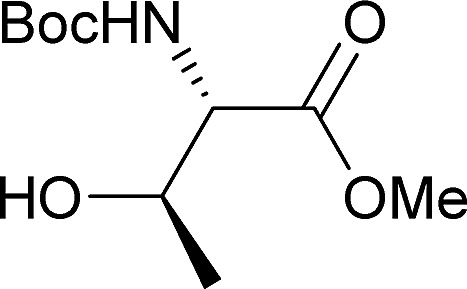	**f**, 91%,^*g*^ α
3	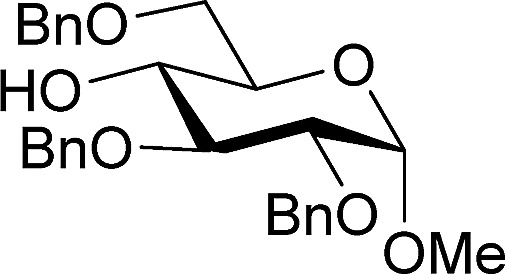	**c**, 55%,^*f*^ α	7	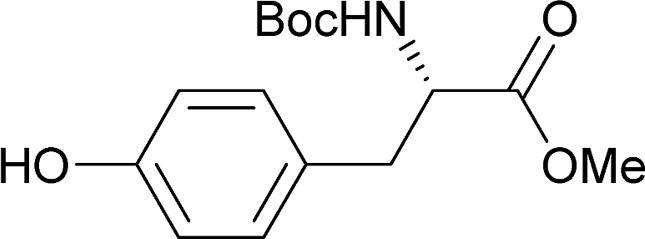	**g**, 94%, α
4	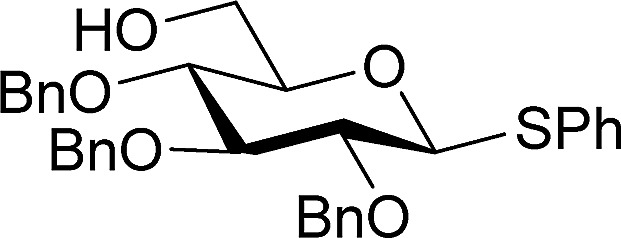	**d**, 82%, α	8	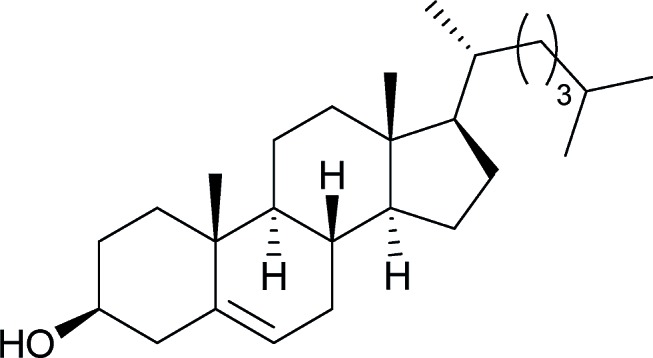	**h**, 98%,^*d*^ α

^*a*^1.2 equiv. of the galactal was used and 0.83 M acceptor in CH_2_Cl_2_ except where stated.

^*b*^Yield of Isolated product.

^*c*^Determined by ^1^H NMR.

^*d*^0.35 M solution w.r.t. acceptor instead of 0.83 M.

^*e*^2 equiv. of galactal w.r.t. acceptor was used.

^*f*^With 17% α,α-**9a** as an impurity.

^*g*^Solvent: 2-methyl-THF (reflux = 83 °C).

### 1,1′-Linked sugars and glycosyl sulfonamides

**Scheme 2:**
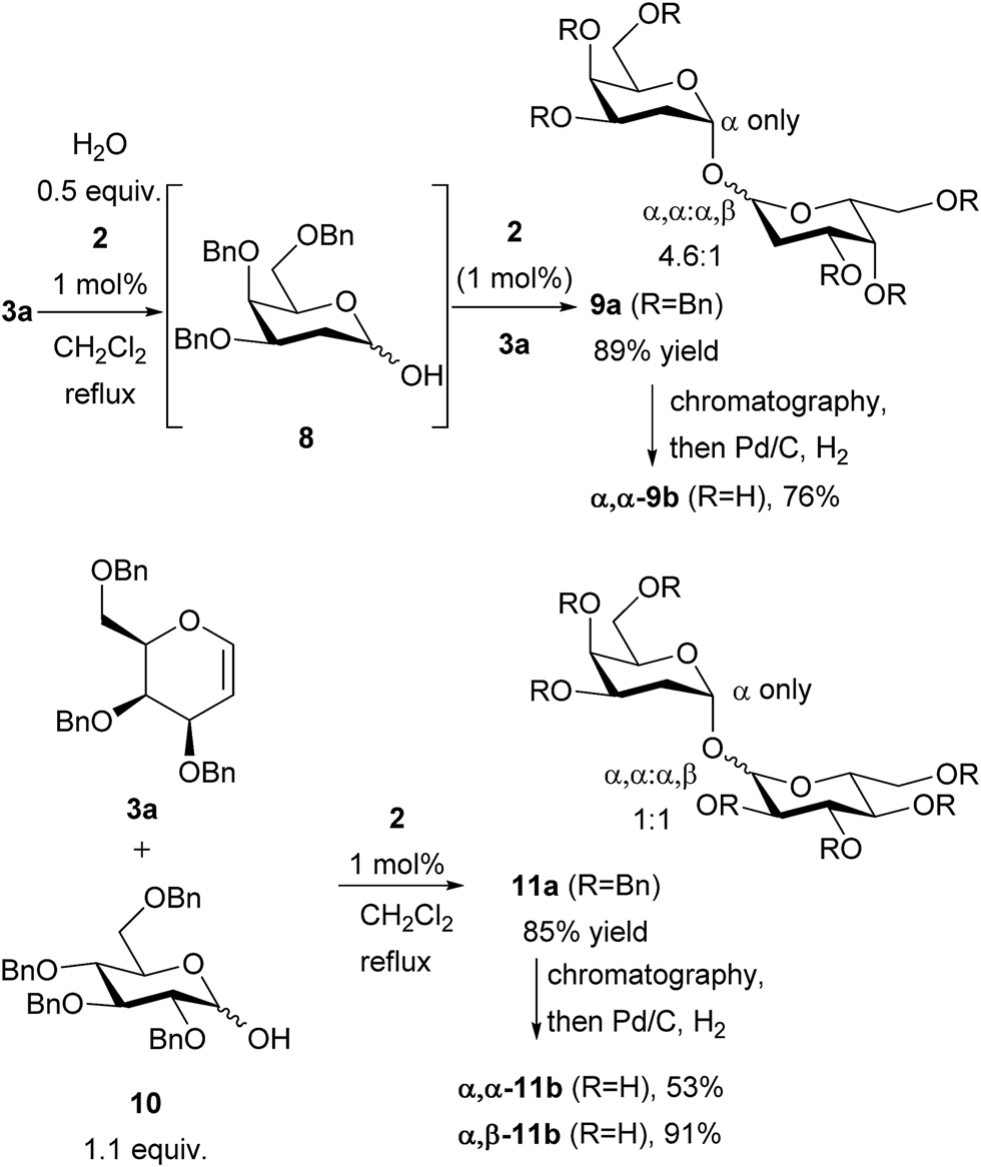
Synthesis of trehalose-type 1,1′-linked dimers and proposed intermediate **8**.

It was noted that the presence of water in the reaction of **3a** and **4** was detrimental to yields, and led to the formation of hydrated side-products. This was turned to advantage by replacing the alcohol with water, enabling the synthesis of 1,1′-linked trehalose-like dimer **9a** (Scheme 2). Sugars of this type are present in the cell wall of mycobacteria including tuberculosis (TB).^15^ The dimer **9a** was obtained as a mixture of α,α : α,β 4.6 : 1. No β,β-dimer was detected. No reaction took place in the absence of catalyst, thus this dimerization is also catalyzed by thiouracil. The stereochemical outcome can be rationalized as follows: following addition of water to galactal **3a** the resulting hemiacetal **8** rapidly epimerizes, but addition of **8** to a second galactal occurs with excellent α-selectivity. We suspect, but cannot prove, that the initial addition of water to the galactal is α-selective. The proposed pathway is supported by the stereoselective addition of hemiacetal **10** to galactal **3a** giving a mixture of α,α : α,β 1 : 1. The use of hemiacetals such as **10** thus demonstrates that heterodimeric trehalose-type structures can also be accessed through this methodology. The epimers were separable by chromatography and the disaccharides were deprotected by hydrogenation over Pd/C to give **9b** and **11b**. Thus the method gives easy access to symmetrical and unsymmetrical 1,1′-linked sugars. Compound **9b** is an inhibitor of mycolyltransesterase enzymes Ag85A, Ag85B and Ag85C which serve as essential mediators of cell envelope function and biogenesis in *Mycobacterium tuberculosis*,15*b* thus this method may enable access to compounds useful for studying/fighting TB. Further efforts in this regard are ongoing in our laboratory.

**Scheme 3:**
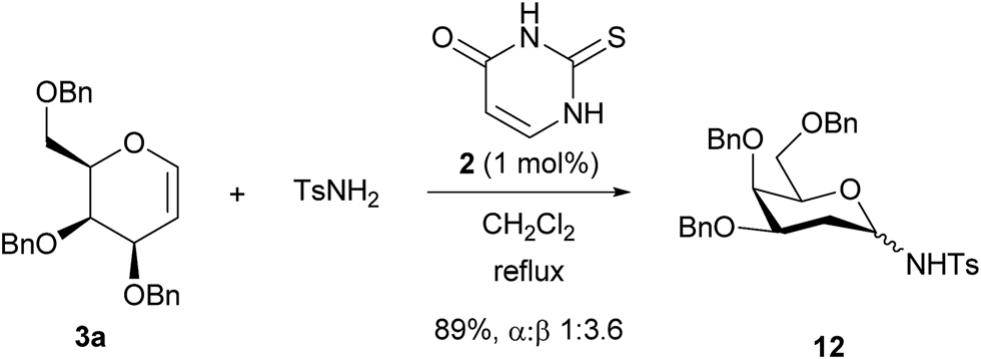
Glycosylation of *p*-toluenesulfonamide.

Glycosyl sulfonamides have been shown to inhibit growth of tumour cell lines. Reaction with TsNH_2_ gave the glycosylated product **12** in 89% yield as a 3.6 : 1 mixture of β : α anomers (Scheme 3), showing that *N*-glycosylation is also possible.^16^ The stereochemical outcome may arise from epimerisation of α-**12** to the β-product, indeed the ratio we observe is similar to that reported by Toste.16a,^17^

### Exploration of mechanism

**Scheme 4:**
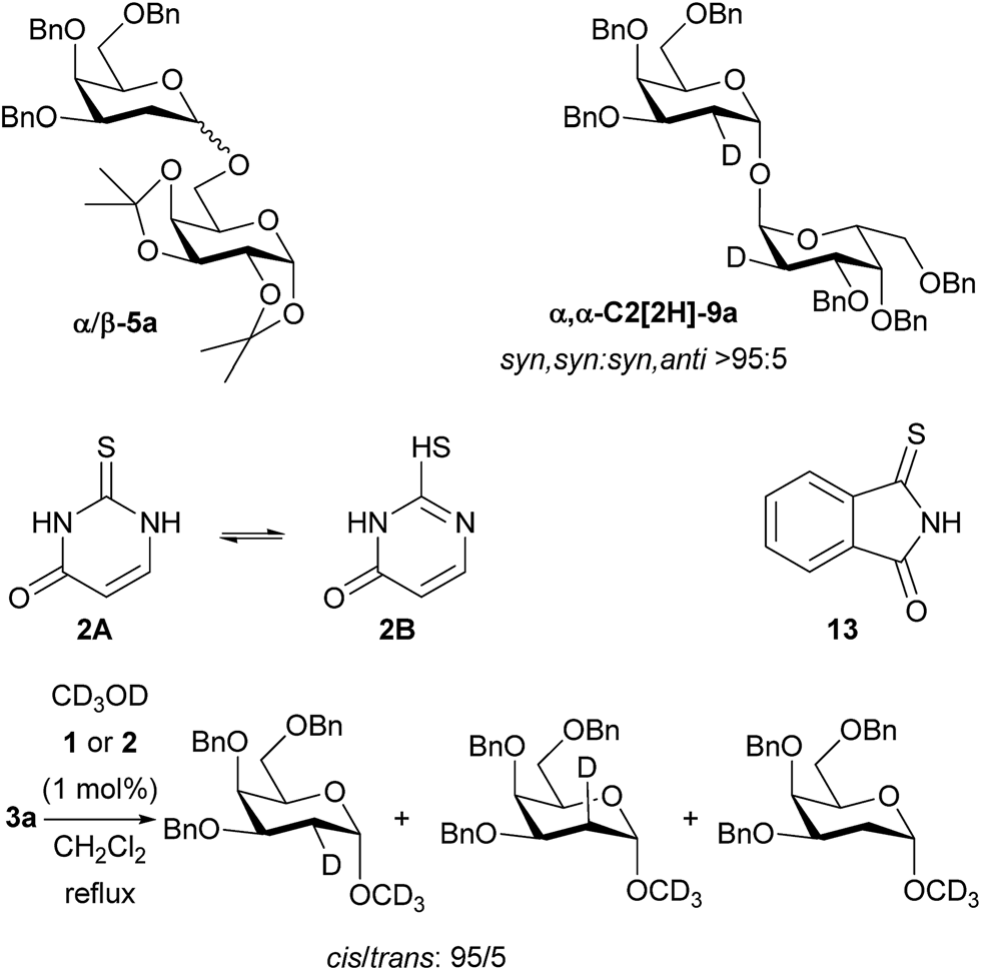
Mechanistic studies on glycosylation: α/β-**5a** doesn't epimerize under reaction conditions. **13** is a viable catalyst (84% yield of **5a**). Reaction of D_2_O with **3a** gives addition of C–D and C–O bonds to same face of **3a**. Schreiner's catalyst and thiouracil give the same outcomes in glycosylation of CD_3_OD.

With regard to the mechanism, we propose that the catalyst is operating *via* an acid/base-type mechanism, rather than through a double-hydrogen bonding mechanism, as we originally proposed. The reactions to form 2-deoxyglycosides, 1,1′-linked sugars and glycosylated sulfonamides all require catalyst – no reaction is observed in the absence of catalyst. When a mixture of α/β-**5a** was resubjected to the reaction conditions the α/β ratio remained constant (Scheme 4). Crossover tests also proved negative (see ESI† for details). Thus, the high α-selectivity observed is obtained kinetically, and is not the result of post-reaction anomerisation, except in the case of 1,1-linked disaccharides where prolonged reaction times increased the amount of α,α-dimer **9a**. The use of D_2_O in place of H_2_O in the formation of α,α-**9a** showed stereoselective (>95 : 5) *syn*-addition of deuterium and oxygen and gives easy access to C2-deuterated α,α-**9a**. Glycosylation of CD_3_OD resulted in a *cis* : *trans* ratio of 95 : 5 for both catalysts **1** and **2** (Scheme 4). This is more *cis*-selective than related reactions of dihydropyrans studied by Varga and Pápai,2*a* and Zimmerman and Nagorny,^18^ but goes against an asynchronous concerted mechanism.^6^ It might be proposed that **2** could be present in solution as the tautomer **2B**, and thus fulfil the role of a double H-bonding catalyst. To counter this, monothiophthalimide **13** was synthesized and shown to be a viable stereoselective catalyst at 1 mol% under our standard glycosylation reaction conditions giving 84% of α-**5a** (Scheme 4). At the very least, this demonstrates the existence of a catalytic pathway that does not require a double H-bonding catalyst, and given the calculations of Varga and Pápai, we contend that it is most likely that thiourea **1**, thiouracil **2**, and monothiophthalimide **13** all operate *via* a common mechanism (we are not aware of any other reports of monothioimides as organocatalysts).

**Scheme 5:**
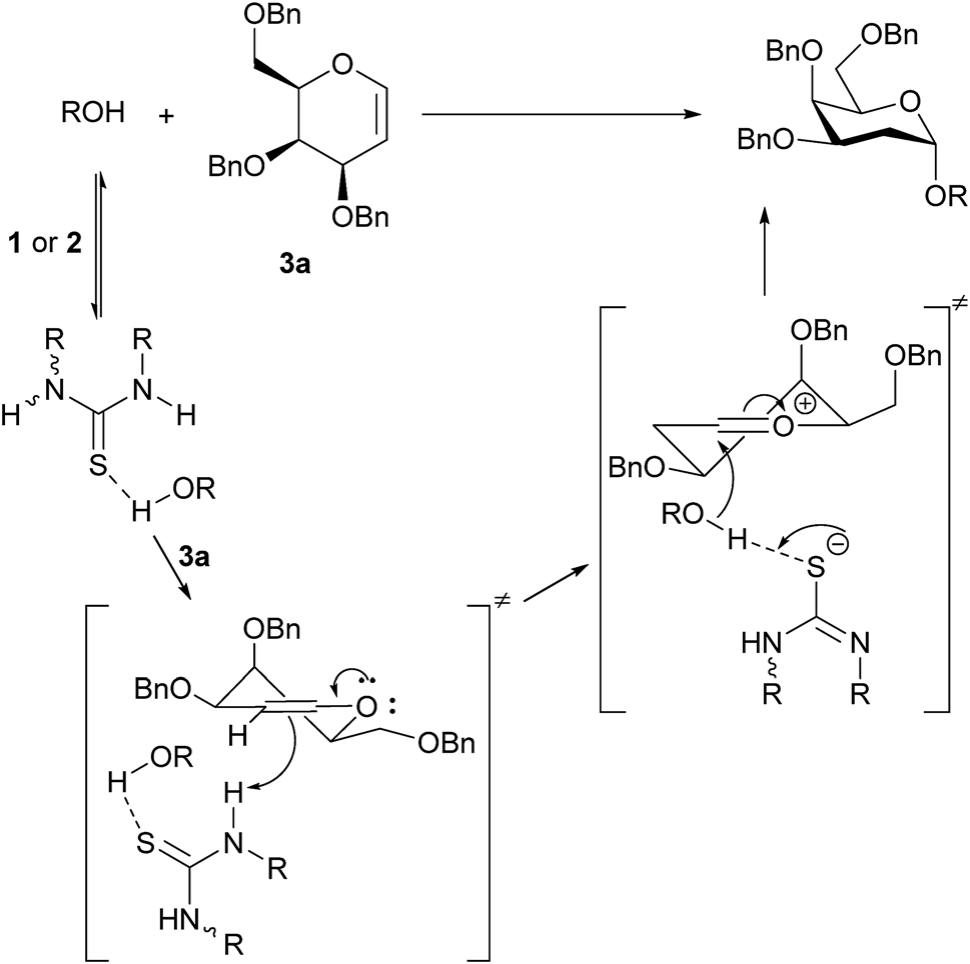
Proposed glycosylation mechanism.

Monothiophthalimide **13** has greater solubility in CH_2_Cl_2_ than thiouracil, thus enabling NMR studies. The chemical shift of the NH proton of **13** changed by 0.4 ppm when alcohol **4** (3.7 equiv.) was added to **13**, consistent with complex formation (see ESI† for details). In the context of our current proposal we can rationalize these results as follows (Scheme 5): we propose that the catalyst first accepts a H-bond from the alcohol substrate making it a stronger acid. We propose a H-bond to sulfur as uracil does not catalyse the reaction, but several structures are conceivable.^19^ It is this catalyst–alcohol complex that interacts with the galactal and undergoes proton transfer. Secondly, the anion formed is able to deprotonate the alcohol in the second step of the reaction as C–O bond formation occurs. We note that we had previously discounted^3^ a ‘simple’ acid-catalyzed mechanism for this reaction as neither Meldrum's acid (p*K*_a_ = 7.3 (DMSO)) nor Et_3_N·HCl (p*K*_a_ = 9.0 (DMSO)) catalyzed the reaction.^20^ It is clear that the chloride anion that would result from Et_3_N·HCl would not be sufficiently basic for this role. We suspect that the same is true of the conjugate base of Meldrum's acid if complexation with alcohol made it a strong enough acid for the first step. The α-stereoselectivity and *syn*-addition of H/D and O–R are consistent with the catalyst : ROH complex favouring approach from the bottom face and the C–O bond forming before the ion pair has time to rearrange.

### Catalyst loading and gram-scale reactions

**Scheme 6:**
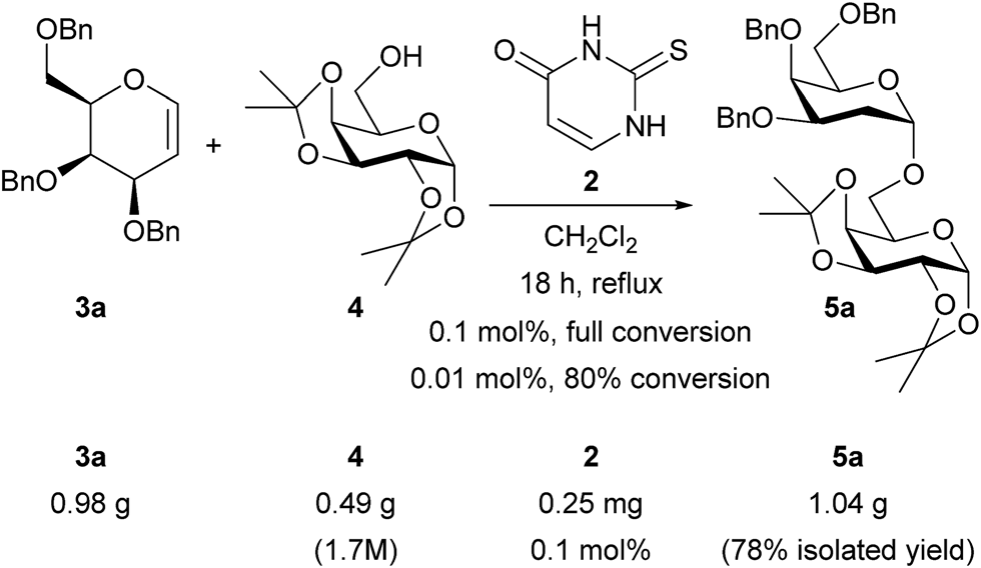
Lower catalyst loadings and gram-scale synthesis of **5a**.

We conducted larger scale experiments in order to explore the catalyst loadings and demonstrate practicality (Scheme 6). Thiouracil **2** was active at loadings down to 0.01 mol% but for reproducible reaction times we chose 0.1 mol% and carried out a 1 g scale reaction.^21^ The concentration was found to be important on bigger scale (1.7 M w.r.t. acceptor; on smaller scale the initial concentration was 0.8 M but some solvent loss occurred during the experiment so the final concentration was higher – see ESI†). Under these optimized conditions just 0.25 mg of catalyst was required to obtain an isolated yield of 1 g (78%) of **5a**.

## Conclusions

In summary, we have shown that thiouracil can replace Schreiner's thiourea in glycosylation reactions. Thus 2-thiouracil is a simple, cheap and effective catalyst for α-selective glycosylation with galactals of monosaccharides, steroids, and amino acids at loadings as low as 0.1 mol%. The method also enables the synthesis of symmetrical and unsymmetrical 1,1′-linked disaccharides including examples relevant to *M. tuberculosis*. Further work in this area is underway in our laboratories.

Furthermore, in line with Varga, Pápai and co-worker's publication on thiourea-catalyzed THP-protection, we are supporting a mechanism whereby the thiourea or thiouracil engages *via* general acid/base catalysis rather than acting as a double-hydrogen bond donor. When considering how a thiourea catalyst is activating substrates it is necessary to consider possibilities beyond double H-bonding. These findings should lead to new opportunities to catalyse reactions and to new organocatalyst designs beyond thioureas. We will report in due course on our own efforts in this regard.

## Conflicts of interest

There are no conflicts to declare.

## Supplementary Material

Supplementary information
