# Supplementary material for: Stereoselective organocatalyzed glycosylations – thiouracil, thioureas and monothiophthalimide act as Brønsted acid catalysts at low loadings
Source: Chem Sci. 2018 Oct 22;10(2):508–14. doi: 10.1039/c8sc02788a (PMC6334493; doi:10.1039/c8sc02788a)

Electronic Supplementary Material (ESI) for Chemical Science.
This journal is © The Royal Society of Chemistry 2018

Stereoselective Organocatalyzed Glycosylations – Thiouracil, Thioureas and Monothiophthalimide act as Brønsted acid catalysts at low loadings

G. A. Bradshaw, A. C. Colgan, N. P. Allen, I. Pongener, M. Boland, Y. Ortin, and E. M. McGarrigle*

Centre for Synthesis & Chemical Biology, UCD School of Chemistry, University College Dublin, Belfield, Dublin 4, Ireland

Supporting Information

Table of Contents

General Experimental 2
Synthesis of Glycal precursors 3
Synthesis of Acceptors 8
Thiouracil-catalysed Glycosylations 14
    2-Thiouracil-Catalysed Glycosylation: Catalyst Loading, Concentration and Gram-Scale Synthesis 29
1,1'-Linked Disaccharides 31
Procedures for Benzyl group removal 36
Mechanistic Studies 38
References 53
NMR Spectra of Compounds 55
    NMR Spectra of Glycals 55
    NMR Spectra of Acceptors and their precursors 66
    NMR Spectra of Disaccharides 71
    NMR Spectra of 1,1,-linked sugars 98
    NMR Spectra of Deprotected sugars 110
    NMR Spectra relating to mechanistic experiments 119

General Experimental

Chemicals were purchased and used without further purification, with the exception of boron trifluoride diethyl etherate, allyl bromide and benzaldehyde dimethyl acetal. These were distilled prior to use and stored under nitrogen ($BF_3.OEt_2$ was distilled from CaH_2).[1] Thiouracil was purchased as ≥99% general purpose reagent. 2-Methyltetrahydrofuran was transferred to a Schlenk tube and dried over freshly activated 4 Å molecular sieves and stored under nitrogen.

Solvents were dried using a Grubbs type still,[2] a Pure Solv-400-3-MD solvent purification system supplied by Innovative Technology Inc. design and stored in Strauss flasks over activated 4Å molecular sieves. Anhydrous DMF was purchased from commercial sources. Reactions requiring anhydrous conditions were performed under nitrogen; glassware was flame-dried immediately prior to use and allowed to cool under reduced pressure. Reactions monitoring by TLC was performed on Merck pre-coated Kieselgel $60F_{254}$ aluminium plates. Visualization was accomplished under UV light (254 nm), staining with ninhydrin solution, and/or by charring with 10% sulfuric acid in ethanol. Flash column chromatography was performed using either silica gel [Davisil, 230-400 mesh (40-63 μm)] or using a Biotage Isolera™ UV-VIS Flash Purification System Version 2.3.1 with SNAP Ultra (25 μm) or SNAP KP-Sil (50 μm) prepacked silica cartridges. High-resolution mass spectra were run on a Waters Micromass GCT system in electrospray ionization mode (ESI). Melting points were recorded on a Reichert thermovar, hot-stage microscope and are uncorrected. Extracts were concentrated *in vacuo* using both a rotary evaporator (bath temperatures up to 50 °C), and a high vacuum line at room temperature. For the removal of DMF, a rotary evaporator (equipped with a 1 L receiving flask; cooled using liquid N_2) connected to a high vacuum line (equipped with an additional collection trap (cooled using liquid N_2) and a rotary vane vacuum pump) was used. ^{1}H NMR and ^{13}C NMR spectra were measured in the solvent stated at 300MHz, 400MHz or 500MHz. Chemical shifts (δ) are quoted in parts per million (ppm) referenced to residual solvent peak (e.g., $CDCl_3$: ^{1}H – 7.26 ppm and ^{13}C – 77.16 ppm) or TMS (^{1}H – 0.00 ppm) and coupling constants (*J*) are given in Hertz. Multiplicities are abbreviated as: b (broad), s (singlet), d (doublet), t (triplet), q (quartet), m (multiplet) or combinations thereof. Assignments were made, where necessary, with the aid of COSY, HSQC and HMBC NMR experiments. For α/β mixtures only peaks that can clearly be assigned have been reported. The units of the specific rotation, (deg·mL)/(g·dm), are implicit and are not included with the reported value. Concentration *c* is given in g/100 mL.

Synthesis of Glycal precursors

1,5-Anhydro-2-deoxy-3,4,6-tri-*O*-benzyl-D-*lyxo*-hex-1-enitol (3a)

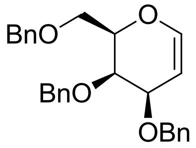

Under a N_2 atmosphere, D-galactal (1.8 g, 12 mmol) was dissolved in anhydrous DMF (50 ml). The flask was cooled to 0 °C (50:50; ice/water), and NaH (60% dispersion in mineral oil) (2.14 g, 53.5 mmol) was added to the reaction flask. The ice bath was removed, and the reaction was left to stir at room temperature for 30 min. The flask was again cooled to 0 °C, and BnBr (5.5 ml, 46 mmol) was added dropwise to the reaction mixture. The ice bath was removed, and the reaction mixture was left to stir at room temperature for 36 h. TLC analysis (4:1 cyclohexane/ethyl acetate; H_2SO_4 stain (10−15% EtOH)) showed that the starting galactal (baseline spot) had been consumed and three spots were present in the reaction mixture (R_f = 0.67, 0.33, 0.03). The reaction was quenched with MeOH (2 ml), and the solvents were removed using rotary evaporation. The crude mixture was dissolved in cyclohexane (100 ml) and washed with 1 M HCl (2 × 30 ml), then saturated $NaHCO_3$ (1 × 30 ml) and deionised H_2O (30 ml).[3] The organic layer was dried using $MgSO_4$, filtered using Büchner filtration, and concentrated *in vacuo*. Purification by column chromatography (98:2 to 85:15; cyclohexane/ethyl acetate) gave **3a** as a white solid (3.7 g, 74% yield). The compound was stored in the freezer under a N_2 atmosphere.

R_f = 0.28 (9:1; cyclohexane/ethyl acetate); mp 51−53 °C (cyclohexane/ethyl acetate) (lit.[4] 49.6−52.0 °C (cyclohexane/ethyl acetate)); ^{1}H NMR (400 MHz, Chloroform-*d*) δ 7.47 – 7.13 (m, 15H, Ph), 6.36 (dd, *J* = 6.3, 1.5 Hz, 1H, H-1), 4.90 – 4.83 (m, 2H, H-2, OC*H*HPh), 4.66 (d, *J* = 12.4 Hz, 1H, OC*H*HPh), 4.64 (d, *J* = 12.4 Hz, 1H, OC*H*HPh), 4.61 (d, *J* = 12.4 Hz, 1H, OC*H*HPh), 4.50 (d, *J* = 11.9 Hz, 1H, OC*H*HPh), 4.42 (d, *J* = 11.9 Hz, 1H, OC*H*HPh), 4.22 – 4.16 (m, 2H, H-3, H-5), 3.97 – 3.92 (m, 1H, H-4), 3.78 (dd, *J* = 10.2, 7.2 Hz, 1H, H-6a), 3.65 (dd, *J* = 10.1, 5.1 Hz, 1H, H-6b); ^{13}C NMR (101 MHz, Chloroform-*d*) δ 144.3 (C-1), 138.6 (4° C), 138.5 (4° C), 138.1 (4° C), 128.5 (CH), 128.5 (CH), 128.3 (CH), 128.0 (CH), 127.8 (CH), 127.7 (CH), 127.6 (CH), 100.1 (C-2), 75.8 (C-3), 73.6 (Ph*C*H_2), 73.5 (Ph*C*H_2), 71.4 (C-4), 71.0 (Ph*C*H_2), 70.9 (C-5), 68.6 (C-6). NMR data were consistent with literature data.[4]

1,5-Anhydro-2-deoxy-3,4,6-tri-*O*-allyl-D-*lyxo*-hex-1-enitol 3b

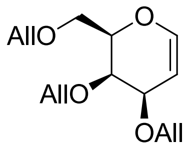

Under a nitrogen atmosphere, D-galactal (1.47 g, 10 mmol) was added to a flame-dried flask equipped with a stirrer bar, and dried *in vacuo* for one hour, before being dissolved fully in anhydrous *N,N'*-dimethylformamide (20 mL) and subsequently cooled to 0 °C . Sodium

hydride (60 wt% in mineral oil, 2.4 g, 60 mmol) was added portionwise with vigorous stirring; upon complete addition the reaction mixture was allowed to return to room temperature and then it was left to stir for 30 minutes. The mixture was re-cooled to 0 °C and freshly distilled allyl bromide (5.2 mL, 60 mmol) was added dropwise with stirring. The mixture was left to stir at ambient temperature for 16 h, becoming dark yellow-brown in appearance. Methanol (5 mL) was added slowly to the reaction to quench the reaction and the mixture was concentrated *in vacuo* to a yellow residue, which was taken up in dichloromethane (20 mL) and washed with water (3 × 20 mL) and brine (3 × 20 mL). The organic phase was dried with magnesium sulfate and concentrated to a pale yellow oil *in vacuo* (approx. 2 g). Purification via silica gel flash chromatography (6:1 to 4:1 cyclohexane:EtOAc, R_f = 0.41) afforded 1,2-dideoxy-3,4,6-tri-*O*-allyl-D-lyxo-1-hexenpyranose **3b** as a slightly pale yellow oil (1.50 g, 56%).

^{1}H NMR (400 MHz, $CDCl_3$) δ: 6.35 (dd, *J* = 6.3, 1.6 Hz, 1H, H-1), 5.99–5.87 (m, 3H, C*H*=CH_2), 5.32–5.24 (m, 3H, CH=C*H*H), 5.21–5.15 (m, 3H, CH=CH*H*), 4.79 (ddd, *J* = 6.3, 2.9, 1.3 Hz, 1H, H-2), 4.33 (ddt, *J* = 12.8, 5.6, 1.4 Hz, 1H, C*H*H), 4.18–3.97 (m, 7H, H-3, H-5, CH*H* and 2 × CH_2), 3.87 (ddd, *J* = 3.9, 2.4, 1.4 Hz, 1H, H-4), 3.75 (dd, *J* = 10.1, 7.0 Hz, 1H, H-6a), 3.69 (dd, *J* = 10.1, 5.4 Hz, H-6b). ^{13}C NMR (101 MHz, $CDCl_3$) δ: 144.2 (C-1), 135.2 (*C*H=CH_2), 135.0 (*C*H=CH_2), 134.6 (*C*H=CH_2), 117.5 (CH=*C*H_2), 117.4 (CH=*C*H_2), 116.8 (CH=*C*H_2), 100.3 (C-2), 75.7 (C-5), 72.9 (CH_2), 72.5 (CH_2), 71.2 (C-4), 70.8 (C-3), 70.0 (CH_2), 68.4 (C-6). NMR data were consistent with literature data.[5]

1,5-Anhydro-2-deoxy-3,4-di-*O*-benzyl-6-*O*-acetyl-D-*lyxo*-hex-1-enitol 3c

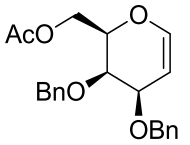

Following a literature procedure,[5] 3,4-di-*O*-benzyl-D-galactal[5] (2.78 g, 8.52 mmol) was dissolved in anhydrous pyridine (37 ml). Acetic anhydride (19 ml, 0.20 mol) was charged to the flask under N_2 and the reaction stirred for 18 h. The reaction was diluted with CH_2Cl_2 (120 ml) and the organic layer washed with 1M HCl (30 ml × 3), $NaHCO_3$ $_{\text{(sat. aq.)}}$ (30 ml × 2) and brine (40 ml), dried over $MgSO_4$ and filtered. Solvent was removed under reduced pressure and the crude oil purified by column chromatography (toluene:EtOAc; 9:1) to give **3c** as a white solid (2.98 g, 95%).

R_f = 0.3 (cyclohexane:EtOAc; 1:1); ^{1}H NMR (300 MHz; $CDCl_3$): δ 7.39-7.24 (m, 10H, Ph), 6.34 (dd, *J* = 6.3, 1.3 Hz, 1H, H-1), 4.91 (ddd, *J* = 6.3, 3.5, 0.9 Hz, 1H, H-2), 4.84 (d, *J* = 11.9 Hz, 1H, OC*H*HPh), 4.71 (d, *J* = 12.1 Hz, 1H, OC*H*HPh), 4.68-4.63 (m, 2H, OC*H*HPh x2), 4.47 (dd, *J* = 12.1, 8.5 Hz, 1H, H-6a), 4.31 (dd, *J* = 12.1, 3.5 Hz, 1H, H-6b), 4.31-4.29 (m, 1H, H-5), 4.17-4.12 (m, 1H, H-3), 3.91 (t, *J* = 3.6 Hz, 1H, H-4), 2.03 (s, 3H, CH_3); ^{13}C NMR (125 MHz; $CDCl_3$): δ 170.9 (C=O), 144.0 (C-1), 138.6 (4° C), 138.1 (4° C), 128.6 (CH), 128.5 (CH), 128.2 (CH), 128.0 (CH), 127.8 (CH), 127.6 (CH), 99.9 (C-2), 74.4 (C-5), 72.8 (OCH_2Ph), 71.9 (C-4), 71.2 (OCH_2Ph), 69.6 (C-3), 63.0 (C-6), 21.1 (CH_3); Spectra were consistent with literature data.[5]

1,5-Anhydro-2-deoxy-3,4,6-tri-*O*-*tert*-butyldimethylsilyl-D-*lyxo*-hex-1-enitol 3d

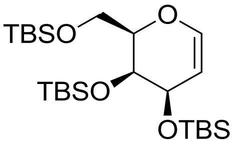

Following a modified literature procedure,[5] D-galactal (811 mg, 5.55 mmol), imidazole (2.90 g, 42.6 mmol) and DMAP (64 mg, 0.53 mmol) were charged to a flame-dried RBF under N_2. After dissolving with anhydrous DMF (31 ml), TBSCl (4.88 g, 32.4 mmol) was added and the reaction heated to 60 °C for 36 h until TLC showed full consumption of starting material (starting material $R_f = 0.25$; product $R_f = 0.55$; cyclohexane:EtOAc; 17:1). The reaction was diluted with pentane (80 ml) and quenched with crushed ice (~80 ml). The layers were separated and the aqueous layer was extracted with pentane (3 × 80 ml). The organic layers were combined and washed with water (2 × 30 ml), brine (2 × 30 ml), dried over Na_2SO_4 and filtered. The solvent was removed under reduced pressure to give **3d** as a colourless oil which was purified by column chromatography (cyclohexane:EtOAc; 17:1) to give the product as a clear colourless oil (1.82 g, 67%).

$R_f = 0.55$ (cyclohexane:EtOAc; 17:1); ^{1}H NMR (500 MHz; $CDCl_3$): δ 6.21 (d, J = 6.1 Hz, 1H, H-1), 4.65 (app t, J = 5.2 Hz, 1H), 4.15-3.95 (m, 4H), 3.91-3.80 (m, 1H), 0.91, 0.898, 0.896 (s x 3, 27H, $SiC(CH_3)_3$ × 3), 0.099, 0.096 (s x 2 6H, $SiCH_3$ × 2), 0.07, 0.06 (s x 2, 12H, $SiCH_3$ × 4); ^{13}C NMR (125.7 MHz; $CDCl_3$): δ 142.8 (C1), 102.3 (C2), 79.8 (br), 68.8 (br), 65.2 (br), 61.1, 26.2 ($SiC(\mathit{C}H_3)_3$), 26.13 ($SiC(\mathit{C}H_3)_3$), 26.06 $SiC(\mathit{C}H_3)_3$), 18.6 ($Si\mathit{C}(CH_3)_3$), 18.42 ($Si\mathit{C}(CH_3)_3$), 18.35 ($Si\mathit{C}(CH_3)_3$), -4.1 ($SiCH_3$), -4.2 ($SiCH_3$), -4.6 ($SiCH_3$), -4.8 ($SiCH_3$), -5.0 ($SiCH_3$), -5.1 ($SiCH_3$). Spectra were consistent with literature data.[5]

1,5-Anhydro-2-deoxy-3,4,6-tri-*O*-acetyl-D-*lyxo*-hex-1-enitol 3e

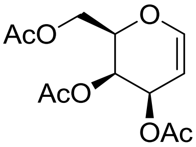

Following a literature procedure,[5] D-galactal (550 mg, 3.76 mmol) was dried under vacuum for 1 h in a flame-dried flask. Anhydrous pyridine (5.5 ml) and acetic anhydride (2.8 ml, 29 mmol) were charged to the flask under N_2 and the reaction was stirred for 18 h. The reaction was diluted with CH_2Cl_2 (55 ml) and the organic layer washed with 1M HCl (20 ml × 2), $NaHCO_3$ $_{\text{(sat. aq.)}}$ (20 ml × 2) and brine (20 ml), dried over Na_2SO_4 and filtered. Solvent was removed under reduced pressure and the crude oil purified by column chromatography (cyclohexane:EtOAc; 2:1) to give **3e** as a clear colourless oil (71 mg, 69%).

R_f = 0.5 (cyclohexane:EtOAc; 2:1; H_2SO_4 (10-15% EtOH) stain); ^{1}H NMR (300 MHz; $CDCl_3$): δ 6.46 (dd, J = 6.3, 1.8 Hz, 1H, H-1), 5.56 (m, 1H), 5.43 (dt, J = 4.4, 1.5 Hz, 1H), 4.73 (ddd, J = 6.3, 2.7, 1.4 Hz, 1H), 4.35-4.18 (m, 3H), 2.13 (s, 3H, CH_3), 2.09 (s, 3H, CH_3), 2.03 (s, 3H, CH_3); ^{13}C NMR (125 MHz; $CDCl_3$): δ 170.6 (C=O), 170.4 (C=O), 170.2 (C=O),

145.5 (C-1), 98.9 (C-2), 72.8, 64.0, 63.8, 62.0, 20.89 (CH_3), 20.83 (CH_3), 20.7 (CH_3). Spectra were consistent with literature data.[5]

1,5-Anhydro-2-deoxy-3,4,6-tri-*O*-benzyl-D-*arabino*-hex-1-enitol 3f

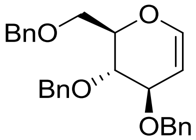

Following the same procedure used for **3a**, D-glucal (0.37 g, 2.5 mmol), NaH (60% dispersion in mineral oil) (0.68 g, 17 mmol), BnBr (1.5 ml, 13 mmol) and anhydrous DMF (15 ml) were used. Purification by column chromatography (98:2 to 90:10; cyclohexane/ethyl acetate) gave **72** as a white solid (0.75 g, 72% yield). The compound was stored in the freezer under a N_2 atmosphere.

R_f = 0.42 (9:1; cyclohexane/ethyl acetate); mp 54−56 ºC (cyclohexane/ethyl acetate) (lit.[6] 57−58 ºC (hexane/ethyl acetate); ^{1}H NMR (500 MHz, Chloroform-*d*) δ 7.44 – 7.18 (m, 15H, Ph), 6.42 (dd, *J* = 6.2, 1.3 Hz, 1H, H-1), 4.87 (dd, *J* = 6.1, 2.7 Hz, 1H, H-2), 4.83 (d, *J* = 11.3 Hz, 1H, OC*H*HPh), 4.64 (d, *J* = 11.3 Hz, 1H, OC*H*HPh), 4.63 (d, *J* = 11.7 Hz, 1H, OC*H*HPh), 4.58 (d, *J* = 12.1 Hz, 1H, OC*H*HPh), 4.54 (app d, *J* = 12.5 Hz, 2H, 2 × OC*H*HPh), 4.24 – 4.18 (m, 1H, H-3), 4.06 (ddd, *J* = 8.3, 5.1, 2.8 Hz, 1H, H-5), 3.86 (dd, *J* = 8.7, 6.2 Hz, 1H, H-4), 3.80 (dd, *J* = 10.8, 5.1 Hz, 1H, H-6a), 3.75 (dd, *J* = 10.7, 2.8 Hz, 1H, H-6b); ^{13}C NMR (126 MHz, Chloroform-*d*) δ 144.8 (C-1), 138.5 (4° C), 138.3 (4° C), 138.1 (4° C), 128.51 (CH), 128.49 (CH), 128.47 (CH), 128.0 (CH), 127.9 (CH), 127.8 (CH), 127.74 (CH), 127.73 (CH), 100.0 (C-2), 76.9 (C-5), 75.8 (C-3), 74.5 (C-4), 73.8 (Ph*C*H_2), 73.6 (Ph*C*H_2), 70.6 (Ph*C*H_2), 68.6 (C-6). Proton and carbon NMR data were consistent with literature data.[7]

3,4-*O*-Dibenzyl-L-rhamnal 3g

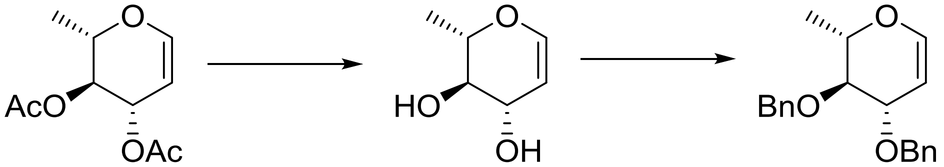

Following a literature procedure,[7] 3,4-*O*-diacetyl-L-rhamnal (1.00 g, 4.67 mmol) was dissolved in a solution of MeOH (8 mL), Et_3N (1.55 mL) and H_2O (1 mL) and stirred at room temperature. TLC analysis (2:1, cyclohexane/ethyl acetate, H_2SO_4 stain) after 38 h showed complete deacetylation of the starting material. The reaction mixture was concentrated *in vacuo* to afford L-rhamnal as a white solid. Under a N_2 atmosphere, L-rhamnal was dissolved in anhydrous THF (10 mL, 0.5 M). The flask was cooled to 0 ºC and NaH (60% dispersion in mineral oil) (717 mg, 17.9 mmol) was added to the solution. The ice-bath was removed and the reaction was stirred at room temperature for 30 minutes. The reaction mixture was again cooled to 0 ºC and treated slowly with BnBr (1.9 mL, 16 mmol). The ice-bath was removed and the reaction mixture was left to stir at room temperature. TLC analysis (3:1, cyclohexane/ethyl acetate, H_2SO_4 stain) after 18 h showed complete consumption of L-rhamnal. The reaction mixture was quenched with MeOH (4 mL), diluted with DCM (60 mL), washed with 1 M HCl (30 mL) followed by saturated $NaHCO_3$ (30 mL) and brine (30

mL). The organic layer was dried over anhydrous $MgSO_4$, filtered and concentrated *in vacuo* to give a yellow oil. Purification by column chromatography (100 to 98:2 pentane/ethyl acetate) afforded **3g** as a white solid (884 mg, 61% yield over two steps).

^{1}H NMR (500 MHz, Chloroform-*d*): δ 7.37 – 7.26 (m, 10H, Ph), 6.36 (dd, *J* = 6.1, 1.1 Hz, 1H, H-1), 4.88 (d, *J* = 11.4 Hz, 1H, OC*H*HPh), 4.86 (dd, *J* = 6.2, 2.5 Hz, 1H, H-2), 4.70 (d, *J* = 11.3 Hz, 1H, OC*H*HPh), 4.66 (d, *J* = 11.7 Hz, 1H, OC*H*HPh), 4.57 (d, *J* = 11.7 Hz, 1H, OC*H*HPh), 4.21 (ddd, *J* = 6.5, 2.5, 1.5 Hz, 1H, H-3), 3.95 (dq, *J* = 8.9, 6.4 Hz, 1H, H-5), 3.48 (dd, *J* = 8.9, 6.5 Hz, 1H, H-4), 1.38 (d, *J* = 6.4 Hz, 3H, 6-CH_3). ^{13}C NMR (101 MHz, Chloroform-*d*): δ 144.9 (C-1), 138.6 (4° C), 138.4 (4° C), 128.55 (CH), 128.53 (CH), 128.1 (CH), 127.9 (CH), 127.8 (CH), 100.3 (C-2), 79.7 (C-4), 76.6 (C-3), 74.2 (Ph*C*H_2), 74.1 (C-5), 70.7 (Ph*C*H_2), 17.6 (C-6). ^{1}H NMR data were in agreement with literature.[8]

3,4-*O*-Dibenzyl-L-fucal 3h

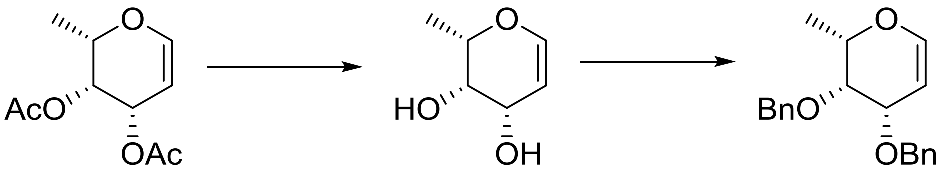

Following a literature procedure,[7] 3,4-*O*-diacetyl-L-fucal (250 mg, 1.17 mmol) was dissolved in a solution of MeOH (8 mL), Et_3N (1 mL) and H_2O (1 mL) and stirred at room temperature. TLC analysis (2:1, cyclohexane/ethyl acetate, H_2SO_4 stain) after 24 h showed complete deacetylation of the starting material. The reaction mixture was concentrated *in vacuo* to afford L-fucal as a white solid. Under a N_2 atmosphere, L-fucal was dissolved in anhydrous THF (3 mL, 0.4 M). The flask was cooled to 0 ºC and NaH (60% dispersion in mineral oil) (187 mg, 4.67 mmol) was added to the solution. The ice-bath was removed and the reaction was stirred at room temperature for 30 minutes. The reaction mixture was again cooled to 0 ºC and treated slowly with BnBr (0.50 mL, 4.2 mmol). The ice-bath was removed and the reaction mixture was left to stir at room temperature. TLC analysis (3:1, cyclohexane/ethyl acetate, H_2SO_4 stain) after 18 h showed complete consumption of L-fucal. The reaction mixture was quenched with MeOH (2 mL), diluted with DCM (40 mL), washed with 1 M HCl (15 mL) followed by saturated $NaHCO_3$ (15 mL) and brine (15 mL). The organic layer was dried over anhydrous Na_2SO_4, filtered and concentrated *in vacuo* to give a yellow oil. Purification by column chromatography (95:5 to 90:10 pentane/ethyl acetate) afforded **3h** as a white solid (254 mg, 70% yield over two steps).

^{1}H NMR (400 MHz, Chloroform-*d*): δ 7.42 – 7.21 (m, 10H, Ph), 6.36 (dd, *J* = 6.3, 1.4 Hz, 1H, H-1), 4.96 (d, *J* = 12.0 Hz, 1H, OC*H*HPh), 4.83 (ddd, 6.4, 2.5, 1.5 1H, H-2), 4.72 (d, *J* = 11.8 Hz, 1H, OC*H*HPh), 4.69 (d, *J* = 11.9 Hz, 1H, OC*H*HPh), 4.62 (d, *J* = 12.2 Hz, 1H, OC*H*HPh), 4.25 (m, 1H, H-3), 4.05 (br q, *J* = 6.6 Hz, 1H, H-5), 3.70 (dt, *J* = 3.9, 1.7 Hz, 1H, H-4), 1.28 (d, *J* = 6.6 Hz, 3H, 6-CH_3). ^{13}C NMR (101 MHz, Chloroform-*d*): δ 144.7 (C-1), 138.72 (4° C), 138.66 (4° C), 128.5 (CH), 128.43 (CH), 128.37 (CH), 127.74 (CH), 127.67 (CH), 127.5 (CH), 99.6 (C-2), 73.8 (Ph*C*H_2), 73.3 (C-4), 73.0 (C-5), 72.3 (C-3), 70.9 (Ph*C*H_2), 16.7 (C-6). NMR data were in agreement with literature.[9]

Synthesis of Acceptors

Methyl 4,6-*O*-benzylidene-α-D-glucopyranoside (14)

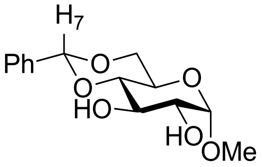

Following the literature procedure,[10] a solution of methyl α-D-glucopyranoside (10.1 g, 52.0 mmol), benzaldehyde dimethylacetal (9.5 ml, 63 mmol) and *p*-TsOH.H_2O (0.15 g, 0.79 mmol) in anhydrous DMF (100 ml) was heated on a rotary-evaporator (50 °C, 200 mbar) for two hours. TLC analysis (ethyl acetate; H_2SO_4 stain (10-15% EtOH)) of the reaction mixture against a sample of pure product showed the desired product had been formed (R_f= 0.43) and that the starting material had been consumed (base-line spot). The DMF was removed using rotary evaporation. The white solid obtained was dissolved in CH_2Cl_2 (100 ml) and washed with sat. $NaHCO_3$ solution (100 ml), followed by brine (100 ml). The organic layer was dried over Na_2SO_4. This was filtered using Büchner filtration and the solvent from the filtrate was removed using rotary evaporation. Purification by column chromatography (95:5; CH_2Cl_2/MeOH) gave **14** as a white solid (11.3 g, 77% yield).

R_f = 0.38 (95:5; CH_2Cl_2/MeOH); mp 164−166 °C (lit.[11] 161−162 °C (CH_2Cl_2/MeOH)); ^1H NMR (400 MHz, Chloroform-*d*) δ 7.55 – 7.44 (m, 2H, Ph), 7.41 – 7.30 (m, 3H, Ph), 5.50 (s, 1H, H-7), 4.73 (d, *J* = 3.9 Hz, 1H, H-1), 4.27 (dd, *J* = 9.6, 4.2 Hz, 1H, H-6a), 3.90 (app t, *J* = 9.3 Hz, 1H, H-3), 3.82 – 3.67 (m, 2H, H-5, H-6b), 3.58 (td, *J* = 8.6, 3.9 Hz, 1H, H-2), 3.45 (t, *J* = 9.3 Hz, 1H, H-4), 3.42 (s, 3H, OCH_3), 3.40 – 3.30 (m, 1H, OH), 2.78 (d, *J* = 8.7 Hz, 1H, OH); ^{13}C NMR (101 MHz, Chloroform-*d*) δ 137.2 (4° C), 129.4 (CH), 128.4 (CH), 126.4 (CH), 102.0 (C-7), 100.0 (C-1), 81.1 (C-4), 72.9 (C-2), 71.6 (C-3), 69.0 (C-6), 62.5 (C-5), 55.6 (OCH_3). Proton and carbon NMR data were consistent with literature data.[5]

It should be noted that at higher concentrations some peaks are subject to change e.g., anomeric (4.67 ppm in 0.4M $CDCl_3$ *vs.* 4.81 ppm in 0.0125M $CDCl_3$). This may be due to a complexation in solution.

Methyl 3-*O*-benzyl-4,6-*O*-benzylidene-α-D-glucopyranoside 6a and Methyl 2-*O*-benzyl-4,6-*O*-benzylidene-α-D-glucopyranoside 6b

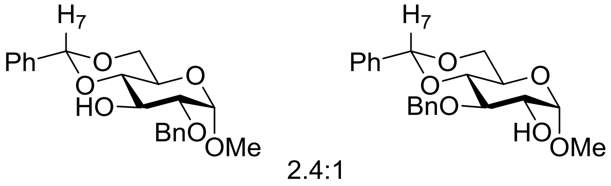

Following the literature procedure,[12] to a stirred solution of **14** (3.0 g, 11 mmol) in CH_2Cl_2 (100 ml), tetrabutylammonium hydrogensulfate (1.2 g, 3.5 mmol) was added followed by aq. NaOH (12.4 ml, 1mM). The mixture was stirred for 30 min at room temperature. BnBr (1.4 ml, 12 mmol) was then added to the reaction mixture. The mixture was heated at reflux for 3 days (time unoptimised). TLC analysis of the reaction mixture (ethyl acetate) showed a small amount of starting material remained (R_f = 0.35) and three new spots appeared in the reaction mixture (R_f = 0.85, 0.75 and 0.65). ^{1}H NMR analysis of the reaction mixture showed that a small amount of starting material remained (~5%) and that the desired products had formed (3-OH/2-OH; 2.4:1 based on integrations of H-7). The aqueous and organic layers were separated and the aqueous layer was extracted with CH_2Cl_2 (3 × 50 ml). The combined organic layers were washed with $NaHCO_3$ (100 ml), brine (100 ml), dried over $MgSO_4$ and filtered using Büchner filtration. The solvent was removed from the filtrate using rotary evaporation which gave a brown oil (9 g). Column chromatography was performed (75:25; cyclohexane/ethyl acetate) but did not lead to the isolation of pure products (R_f= 0.24 (3-OH) and 0.14 (2-OH)). Concentration of the solution of the mixed fractions containing the 3-OH product and other impurities, but free of 2-OH product, allowed the desired product **6b** to crystallise from the remaining ethyl acetate as a white solid (1.48 g, 37% yield). Mixed fractions containing the 2-OH product and other impurities, but free of the 3-OH product, were concentrated using rotary evaporation and dissolved using a mixture of CH_2Cl_2/cyclohexane/MeOH (4.75:0.25:5). Removal of the CH_2Cl_2 by rotary evaporation allowed the desired product **6a** to crystallize from the remaining cyclohexane/methanol as a white solid (0.58 g, 15% yield). 0.5 g (13% yield) remained as mixed fractions containing the two desired products plus unidentified impurities.

Methyl 3-*O*-benzyl-4,6-*O*-benzylidene-α-D-glucopyranoside (6a): R_f = 0.14 (75:25; cyclohexane/ethyl acetate); mp 180−182 °C (lit.[13] 188−189 °C (EtOH)); ^{1}H NMR (400 MHz, Chloroform-*d*) δ 7.54 – 7.43 (m, 2H, Ph), 7.42 – 7.26 (m, 8H, Ph), 5.57 (s, 1H, H-7), 4.96 (d, *J* = 11.6 Hz, 1H, OC*H*HPh), 4.81 (d, *J* = 4.0 Hz, 1H, H-1), 4.79 (d, *J* = 11.7 Hz, 1H, OC*H*HPh), 4.30 (dd, *J* = 9.8, 4.4 Hz, 1H, H-6a), 3.88 – 3.80 (m, 2H, H-3, H-5), 3.80 – 3.69 (m, 2H, H-2, H-6b), 3.64 (t, *J* = 9.2 Hz, 1H, H-4), 3.45 (s, 3H, OCH_3), 2.30 (d, *J* = 7.3 Hz, 1H, OH); ^{13}C NMR (101 MHz, Chloroform-*d*) δ 138.6 (4° C), 137.5 (4° C), 129.1 (CH), 128.5 (CH), 128.4 (CH), 128.1 (CH), 127.9 (CH), 126.2 (CH), 101.4 (C-7), 100.0 (C-1), 82.1 (C-4), 79.0 (C-3), 75.0 (PhCH_2), 72.6 (C-2), 69.2 (C-6), 62.7 (C-5), 55.6 (OCH_3). Proton and carbon NMR data were consistent with literature data.[5]

Methyl 2-*O*-benzyl-4,6-*O*-benzylidene-α-D-glucopyranoside (6b): R_f = 0.24 (75:25; cyclohexane/ethyl acetate); mp 126−128 °C (lit.[4] 129.7−130.4 °C (CH_2Cl_2)); ^{1}H NMR (400 MHz, Chloroform-*d*) δ 7.54 – 7.44 (m, 2H, Ph), 7.43 – 7.28 (m, 8H, Ph), 5.52 (s, 1H, H-7),

4.79 (d, J = 12.2 Hz, 1H, OC*H*HPh), 4.70 (d, J = 12.2 Hz, 1H, OC*H*HPh), 4.61 (d, J = 3.6 Hz, 1H, H-1), 4.26 (dd, J = 10.1, 4.7 Hz, 1H, H-6a), 4.15 (app td, J = 9.3, 1.7 Hz, 1H, H-3), 3.81 (ddd, J = 10.0, 9.7, 4.7 Hz, 1H, H-5), 3.70 (t, J = 10.2 Hz, 1H, H-6b), 3.49 (t, J = 9.3 Hz, 1H, H-4), 3.47 (dd, J = 9.2, 3.7 Hz, 1H, H-2), 3.38 (s, 3H, OCH_3), 2.55 (d, J = 1.5 Hz, 1H, OH); ^{13}C NMR (101 MHz, Chloroform-*d*) δ 138.0 (4° C), 137.2 (4° C), 129.3 (CH), 128.7 (CH), 128.4 (CH), 128.29 (CH), 128.27 (CH), 126.5 (CH), 102.1 (C-7), 98.8 (C-1), 81.4 (C-4), 79.7 (C-2), 73.5 (Ph*C*H_2), 70.4 (C-3), 69.1 (C-6), 62.2 (C-5), 55.5 (OCH_3). Proton and carbon NMR data were consistent with literature data.[5]

Methyl 2,3-di-*O*-benzyl-4,6-*O*-benzylidene-α-D-glucopyranoside 15

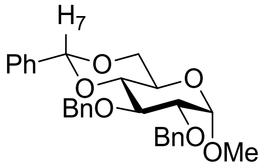

Under a N_2 atmosphere, **14** (1.99 g, 7.1 mmol) as weighed into the flask and dissolved in anhydrous DMF (70 ml). The solution was cooled to 0 °C (50:50; ice/water). NaH (60% dispersion in mineral oil) (1.19 g, 29.7 mmol) was added to the reaction mixture. The ice-bath was removed and the reaction mixture was left to stir at room temperature for 30 min. The reaction was again cooled to 0 °C and BnBr (3.5 ml, 29 mmol) was added dropwise to the reaction. The reaction was left stirring at room temperature, under a N_2 atmosphere, for 16.5 h (time unoptimised). TLC analysis (7:3; cyclohexane/ethyl acetate; H_2SO_4 stain (10-15% EtOH)) against a pure sample of product showed that the starting material was consumed in the reaction and the desired product had formed (R_f = 0.73). MeOH (1 ml) was added to the reaction mixture to quench the reaction. The solvents were removed using rotary evaporation which gave a yellow solid. The solid obtained was dissolved in CH_2Cl_2 (75 ml) and washed with deionised water (2 × 75 ml). The aqueous layer was then extracted with CH_2Cl_2 (3 × 75 ml). The organic layers were combined and washed with brine (150 ml) and then dried with $MgSO_4$, filtered and concentrated *in vacuo*. Purification by column chromatography (85:15; cyclohexane/ethyl acetate) gave a white solid (2.71 g, 82% yield). A small impurity (~10%) was present in this sample following column chromatography. 1.51 g of this sample was recrystallised using *n*-hexanes which gave **15** as white solid (1.28 g, 39% yield).

R_f = 0.29 (85:15; cyclohexane/ethyl acetate); mp; 94−95 °C (lit.[4] 93−95 °C (cyclohexane/ethyl acetate)). ^{1}H NMR (400 MHz, Chloroform-*d*) δ 7.55 – 7.44 (m, 2H, Ph), 7.43 – 7.21 (m, 13H, Ph), 5.54 (s, 1H, H-7), 4.91 (d, J = 11.3 Hz, 1H, OC*H*HPh), 4.85 (d, J = 12.1 Hz, 1H, OC*H*HPh), 4.83 (d, J = 11.3 Hz, 1H, OC*H*HPh), 4.69 (d, J = 12.2 Hz, 1H, OC*H*HPh), 4.60 (d, J = 3.7 Hz, 1H, H-1), 4.26 (dd, J = 10.1, 4.7 Hz, 1H, H-6a), 4.05 (t, J = 9.3 Hz, 1H, H-3), 3.83 (td, J = 9.9, 4.7 Hz, 1H, H-5), 3.70 (t, J = 10.2 Hz, 1H, H-6b), 3.60 (t, J = 9.4 Hz, 1H, H-4), 3.56 (dd, J = 9.3, 3.7 Hz, 1H, H-2), 3.40 (s, 3H, OCH_3); ^{13}C NMR (101 MHz, Chloroform-*d*) δ 138.8 (4° C), 138.3 (4° C), 137.5 (4° C), 129.0 (CH), 128.6 (CH), 128.4 (CH), 128.3 (CH), 128.2 (CH), 128.1 (CH), 128.0 (CH), 127.7 (CH), 126.1 (CH), 101.4 (C-7), 99.3 (C-1), 82.3 (C-4), 79.3 (C-2), 78.7 (C-3), 75.5 (Ph*C*H_2), 73.9 (Ph*C*H_2), 69.2

(C-6), 62.4 (C-5), 55.5 (OCH_3). Proton and carbon NMR data were consistent with literature data.[5]

Methyl 2,3,6-tri-*O*-benzyl-α-D-glucopyranoside 6c

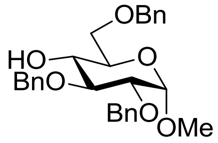

Based on literature procedures,[14,15] 4Å molecular sieves (powdered) (450 mg) were weighed into a three-necked round bottom flask. This flask was flame-dried using a propane torch, allowed to cool under vacuum and then switched to a N_2 atmosphere. The flask was equipped with a stir-bar, gas inlet and thermometer. Pyranoside **15** (1 g, 2 mmol) was added, followed by $NaCNBH_3$ (1.0M in THF, 35 ml, 35 mmol). The mixture was cooled to 0 °C (50:50; ice/water;). HCl (4.0M in dioxane, 10 ml) was added to the reaction mixture until the pH reached 1-2 and no more fizzing was observed upon addition. The reaction mixture was left to warm to room temperature. TLC analysis (4:1; cyclohexane/ethyl acetate) showed that the sugar starting material (R_f = 0.26) had been consumed in the reaction and two new spots appeared (R_f = 0.11 and a baseline spot). The reaction mixture was diluted with EtOAc (40 ml) and deionised H_2O (40 ml) and then filtered using Büchner filtration. The filtrate was transferred to a separating funnel and sat. $NaHCO_3$ solution (40 ml) was added. The aqueous layer was extracted with EtOAc (3 × 40 ml). The combined organic layers were washed with brine (80 ml). The organic layer was dried using Na_2SO_4, filtered and concentrated *in vacuo.* Purification by column chromatography (7:3; cyclohexane/ethyl acetate) gave **6c** as a colourless oil (0.76 g, 76% yield).

R_f = 0.37 (7:3; cyclohexane/ethyl acetate); ^{1}H NMR (400 MHz, Chloroform-*d*) δ 7.48 – 7.17 (m, 15H, Ph), 5.00 (d, *J* = 11.4 Hz, 1H, OC*H*HPh), 4.77 (d, *J* = 12.2 Hz, 1H, OC*H*HPh), 4.73 (d, *J* = 11.6 Hz, 1H, OC*H*HPh), 4.66 (d, *J* = 12.2 Hz, 1H, OC*H*HPh), 4.63 (d, *J* = 3.6 Hz, 1H, H-1), 4.59 (d, *J* = 12.2 Hz, 1H, OC*H*HPh), 4.54 (d, *J* = 12.2 Hz, 1H, OC*H*HPh), 3.78 (app t, *J* = 9.1 Hz, 1H, H-3), 3.74 – 3.65 (m, 3H, H-5, H-6a, H-6b), 3.60 (td, *J* = 9.1, 2.3 Hz, 1H, H-4), 3.53 (dd, *J* = 9.5, 3.6 Hz, 1H, H-2), 3.38 (s, 3H, OCH_3), 2.33 (d, *J* = 2.4 Hz, 1H, OH) ^{13}C NMR (101 MHz, Chloroform-*d*) δ 138.9 (4° C), 138.2 (4° C), 138.1 (4° C), 128.7 (CH), 128.6 (CH), 128.5 (CH), 128.3 (CH), 128.14 (CH), 128.10 (CH), 128.0 (CH), 127.78 (CH), 127.77 (CH), 98.3 (C-1), 81.6 (C-3), 79.8 (C-2), 75.6 (Ph*C*H_2), 73.7 (Ph*C*H_2), 73.3 (Ph*C*H_2), 70.9 (C-4), 70.0 (C-5), 69.6 (C-6), 55.4 (OCH_3). Proton and carbon NMR data were consistent with literature data.[5]

Phenyl 2,3,4-tri-*O*-benzyl-β-*O*-D-thioglucopyranoside 6d

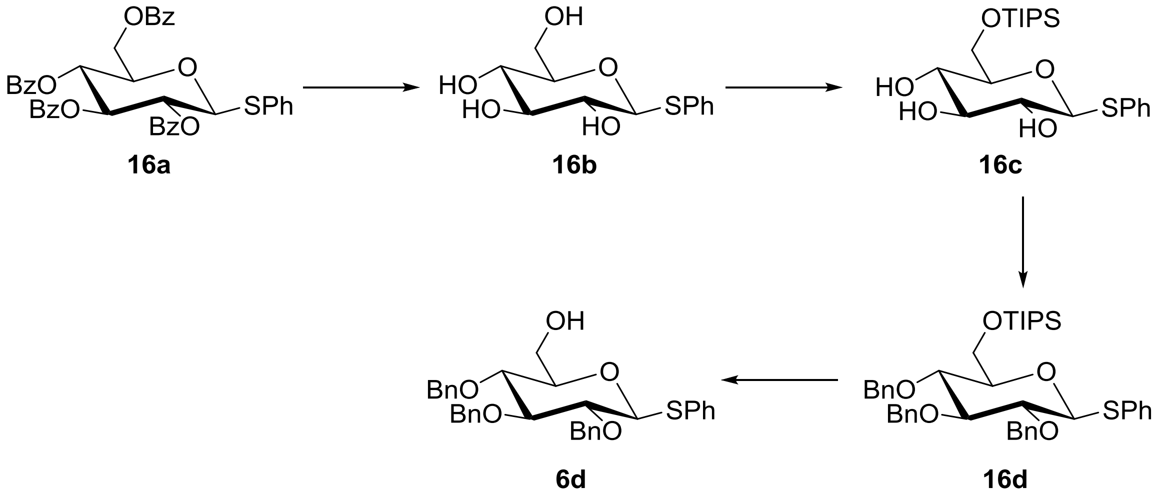

Following modified literature procedures,[5,16] **16a** (3.9 g, 5.7 mmol) was dissolved in methanol (150 ml) and THF (30 ml). 3M $NaOH_{aq}$ (24 ml) was added and the reaction was stirred for 16 h. TLC (cyclohexane:EtOAc; 2:1) showed full consumption of starting material (R_f = 0.5) and a new product at R_f = 0.0. The reaction was concentrated under reduced pressure to remove the THF and MeOH. The resulting solution was neutralised (pH 7) with 2M HCl. The aqueous layer was extracted with EtOAc (15 ml × 15) and solvent removed under reduced pressure to give crude **16b** as an off-white solid (1.32 g, 88%).

R_f = 0.0 (cyclohexane:EtOAc; 2:1); ^{1}H NMR (500 MHz; $CDCl_3$): δ 7.65-7.54 (m, 2H, Ph), 7.48-7.35 (m, 3H, Ph), 3.90 (dd, *J* = 12.5, 2.2 Hz, 1H, H-6a), 3.72 (dd, *J* = 12.5, 5.5 Hz, 1H, H-6b), 3.58-3.31 (m, 4H, 4 x CH); ^{13}C NMR (From HSQC; 125.7 MHz; $CDCl_3$): δ 131.6 (CH), 129.3 (CH), 128.1 (CH), 87.2 (C-1), 79.2 (CH), 77.1 (CH), 71.6 (CH), 69.3 (CH), 60.6 (C-6).

Crude **16b** (960 mg, 3.53 mmol) and imidazole (490 mg, 7.2 mmol) were charged to a RBF and dried under high vacuum for 3 h. Anhydrous DMF (12.5 ml) was added to the reaction and when everything was in solution TIPSCl (2 ml, 9.4 mmol) was added to the RBF under N_2 and stirred for 18 h. The solution was concentrated under reduced pressure and the residue was dissolved in CH_2Cl_2 (50 ml), washed with water (35 ml), brine (30 ml), dried over $MgSO_4$, filtered and concentrated under reduced pressure to give **16c**. Selected peaks from ^{1}H NMR (500 MHz; $CDCl_3$): δ 7.55-7.50 (m, 2H, Ph), 7.31-7.26 (m, 3H, Ph), 4.55 (d, *J* = 9.7 Hz, 1H, H-1), 4.04 (dd, *J* = 10.2, 5.0 Hz, 1H), 3.93 (dd, *J* = 10.3, 6.0 Hz, 1H), 3.64-3.57 (m, 2H), 3.45 (dt, *J* = 8.8, 5.9 Hz, 1H), 3.38-3.33 (m, 1H).

The intermediate **16c** was then dissolved in anhydrous DMF (16 ml) and the solution was cooled in an ice bath. NaH (60% dispersion in mineral oil) (790 mg, 19.8 mmol) was charged to the flask and the reaction was stirred for 1 h at RT. Then the reaction was cooled in an ice bath before benzyl bromide (2.3 ml, 19 mmol) was added dropwise. The reaction was left stirring under N_2 at RT for 72 h. TLC analysis (cyclohexane:EtOAc; 2:1; H_2SO_4 (10-15% EtOH) stain) showed that intermediates were still present. NaH (60% dispersion in mineral

oil) (20 mg, 5.0 mmol) was charged to the flask and the reaction was stirred for 1 h. Benzyl bromide (0.6 ml, 5 mmol) was added dropwise and the reaction stirred overnight (time unoptimised). The reaction was quenched with MeOH (5 ml) and solvent concentrated under reduced pressure. The residue was then dissolved in CH_2Cl_2 (50 ml), washed with water (35 ml), brine (30 ml), dried over $MgSO_4$, filtered and concentrated under reduced pressure to give **16d**. Following purification by column chromatography (cyclohexane:EtOAc; 98:2) phenyl 2,3,4-tri-*O*-benzy-6-*O*-triisopropylsilyl-β-*O*-D-thio glucopyranoside **16d** was obtained as a white solid (1.48 g, 60%).

R_f = 0.3 (cyclohexane:EtOAc; 98:2); ^{1}H NMR (400 MHz; $CDCl_3$): δ 7.62-7.49 (m, 2H, Ph), 7.42-7.19 (m, 18H, Ph), 4.90-4.56 (dd, *J* = 12.5, 5.5 Hz, 6H), 4.86 (dd, *J* = 12.5, 5.5 Hz, 1H), 4.04-3.85 (m, 2H), 3.76-3.62 (m, 2H), 3.55-3.42 (m, 1H), 3.34 (ddd, *J* = 9.5, 3.8, 1.8 Hz, 1H), 1.12-1.01 (m, 21H, $^i Pr_3Si$).

Intermediate **16d** (1.14 g, 1.63 mmol) was charged to a round bottom flask under N_2. A 1M TBAF solution in THF (3.0 mL, 3 mmol) was added to the flask and the resulting solution stirred under N_2 at RT. After 2 h TLC showed full consumption of starting material (R_f = 0.85) and the presence of product at R_f = 0.55 (cyclohexane:EtOAc; 1:1.5). The reaction was diluted with CH_2Cl_2 (50 mL) and washed with water (25 mL), brine (25 mL), and dried over $MgSO_4$ and filtered. The solvent was removed under reduced pressure to give a cloudy white oil. Following purification by column chromatography (cyclohexane:EtOAc; 1:1.5), phenyl 2,3,4-tri-*O*-benzyl-β-*O*-D-thioglucopyranoside **6d** was obtained as a white solid (600 mg, 68%).

R_f = 0.3 (cyclohexane:EtOAc; 1:1.5); ^{1}H NMR (500 MHz; $CDCl_3$): δ 7.53-7.49 (m, 2H, Ph), 7.40-7.36 (m, 2H, Ph), 7.36-7.22 (m, 16H, Ph), 4.93-4.83 (m, 4H, PhC*H*$_2$, PhC*H*H × 2), 4.76 (d, *J* = 10.2 Hz, 1H, PhC*H*H), 4.72 (dd, *J* = 9.8, 1.1 Hz, 1H, H-1), 4.65 (d, *J* = 10.9 Hz, 1H, PhC*H*H), 3.87 (ddd, *J* = 12.0, 5.5, 2.5 Hz, 1H, H-6a), 3.73 (t, *J* = 9.0 Hz, 1H, H-3), 3.72-3.66 (m, H-6b), 3.58 (t, *J* = 9.4 Hz, 1H, H-4), 3.49 (app t, *J* = 9.3 Hz, 1H, H-2), 3.39 (ddd, *J* = 9.0, 4.5, 2.4 Hz, 1H, H-5), 2.00-1.94 (m, 1H, OH). ^{13}C NMR (125.7 MHz; $CDCl_3$): δ 138.4 (4° C), 138.02 (4° C), 137.96 (4° C), 133.6 (4° C), 132.0 (CH), 129.2 (CH), 128.6 (CH), 128.59 (CH), 128.55 (CH), 128.3 (CH), 128.2 (CH), 128.1 (CH), 128.0 (CH), 127.90 (CH), 127.87 (CH), 127.8 (CH), 87.7 (C-1), 86.7 (C-3), 81.2 (C-2), 79.5 (C-5), 77.7 (C-4), 75.9 (Ph*C*H$_2$), 75.7 (Ph*C*H$_2$), 75.2 (Ph*C*H$_2$), 62.3 (C-6). Spectra were consistent with literature data.

Acceptors **6e**-**h** were purchased from commercial suppliers and used without further purification.

Thiouracil-catalysed Glycosylations

General Procedure 1

A 5 ml RBF equipped with a stir-bar, gas-inlet and reflux condenser was set up under a N_2 atmosphere. The glycal donor (1.2 eq) was weighed into the round-bottomed flask and placed under vacuum for *ca.* 30 min. An anhydrous solution of acceptor (0.8M in CH_2Cl_2) was made by charging a known quantity of acceptor to a flask under N_2. Anhydrous CH_2Cl_2 was added to make up a 0.8M solution. The stock solution was dried by adding $MgSO_4$ (1:1 mol/mol w.r.t. acceptor) and let sit for 30 min. The stock solution of acceptor in CH_2Cl_2 (1 eq) was then decanted by syringe and added to the glycal donor under N_2. After everything was in solution, 2-thiouracil (1 mol%) was added giving a suspension. The reaction was refluxed under N_2 for 18 h or until TLC or NMR analysis showed the reaction was complete. Some solvent loss was noted over the course of the reaction and the higher concentration that results is believed to be beneficial. The solution was then concentrated under reduced pressure and purified by column chromatography.

General Procedure 2

The glycal donor (0.6 mmol) and acceptor (0.5 mmol) were weighed into a round bottomed flask equipped with a stirring bar under N_2 and put under vacuum for 40 minutes. The flask was refilled with N_2 before anhydrous CH_2Cl_2 (0.6 ml) was added to make a 0.8M solution wrt the acceptor. After everything was in solution 2-thiouracil (1 mol%) was added to the solution under N_2. The reaction was refluxed under N_2 for 18 h or until TLC or NMR analysis showed the reaction was complete. Some solvent loss was noted over the course of the reaction and the higher concentration that results is believed to be beneficial. The solution was then concentrated under reduced pressure and purified by column chromatography.

Table 1

6-*O*-(3,4,6-Tri-*O*-benzyl-2-deoxy-α-D-lyxo-hexapyranosyl)-1,2:3,4-di-*O*-isopropylidene-α-D-galactopyranose 5a

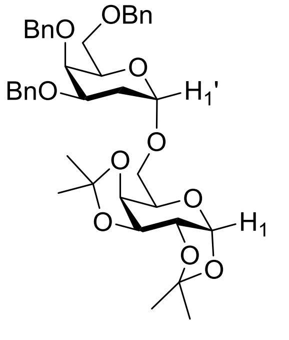

Following General Procedure 1, galactal **3a** (100 mg, 0.24 mmol), an anhydrous CH_2Cl_2 solution of galactose acceptor **4** (0.8M, 0.24 ml, 0.2 mmol) and 2-thiouracil (0.3 mg, 2 μmol) were used. The mixture was heated at reflux temperature for 18 h. Following purification by column chromatography (4:1; cyclohexane/ethyl acetate) the product was obtained as a pale yellow oil (128 mg, 95% yield).

R_f = 0.4 (cyclohexane:EtOAc; 4:1); ^{1}H NMR (400 MHz, Chloroform-*d*) δ 7.45 – 7.09 (m, 15H, Ph), 5.52 (d, *J* = 5.0 Hz, 1H, H-1), 5.03 (d, *J* = 3.3 Hz, 1H, H-1'), 4.92 (d, *J* = 11.6 Hz, 1H, OC*H*HPh), 4.61 (d, *J* = 11.4 Hz, 1H, OC*H*HPh), 4.60 – 4.56 (m, 3H, H-3, 2 × OC*H*HPh), 4.49 (d, *J* = 11.8 Hz, 1H, OC*H*HPh), 4.42 (d, *J* = 11.8 Hz, 1H, OC*H*HPh), 4.30 (dd, *J* = 5.0, 2.4 Hz, 1H, H-2), 4.21 (dd, *J* = 8.0, 1.9 Hz, 1H, H-4), 4.01 – 3.88 (m, 4H, H-5, H-3', H-4', H-5'), 3.74 (dd, *J* = 10.7, 6.7 Hz, 1H, H-6a'), 3.69 – 3.64 (m, 1H, H-6b'), 3.62 (dd, *J* = 9.4, 7.6 Hz, 1H, H-6a), 3.54 (dd, *J* = 9.2, 5.6 Hz, 1H, H-6b), 2.22 (td, *J* = 12.3, 3.6 Hz, 1H, H-2a'), 2.07 – 1.95 (m, 1H, H-2b'), 1.51 (s, 3H, CH_3), 1.42 (s, 3H, CH_3), 1.33 (s, 6H, 2 × CH_3); ^{13}C NMR (101 MHz, Chloroform-*d*) δ 139.1 (4° C), 138.8 (4° C), 138.3 (4° C), 128.5 (CH), 128.4 (CH), 128.3 (CH), 128.0 (CH), 127.8 (CH), 127.6 (CH), 127.4 (CH), 109.5 ($O_2C(CH_3)_2$), 108.7 ($O_2C(CH_3)_2$), 97.7 (C-1'), 96.5 (C-1), 74.9 (CH), 74.5 (Ph*C*H_2), 73.5 (Ph*C*H_2), 73.1 (CH), 71.2 (C-4), 70.82, 70.77 (C-2, C-3), 70.6 (Ph*C*H_2), 70.0 (CH), 69.4 (C-6), 66.0 (CH), 65.7 (C-6'), 31.8 (C-2'), 26.3 (CH_3), 26.1 (CH_3), 25.1 (CH_3), 24.7 (CH_3). Proton and carbon NMR data were consistent with literature data.[5]

6-*O*-(3,4,6-Tri-*O*-allyl-2-deoxy-α-D-lyxo-hexapyranosyl)-1,2:3,4-di-*O*-isopropylidene-α-D-galactopyranose 5b

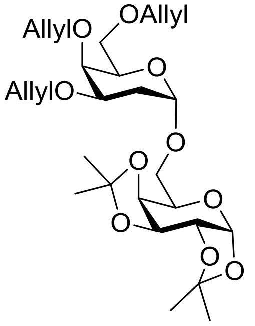

Following General Procedure 1, tri-*O*-allyl-D-galactal **3b** (107 mg, 0.40 mmol) and anhydrous CH_2Cl_2 solution of galactose **4** (0.8M, 0.39 mL, 0.31 mmol) and 2-thiouracil (0.4 mg, 0.003 mmol, 1 mol%) for 14 h. Purification by flash chromatography (95:5 to 80:20 cyclohexane/ethyl acetate) afforded the product as a colourless oil (134 mg, 82%).

R_f = 0.27 (8:2; cyclohexane/ethyl acetate); ^{1}H NMR (400 MHz, $CDCl_3$) δ: 6.01–5.85 (m, 3H, C*H*=CH_2), 5.52 (d, *J* = 5.0 Hz, 1H, H-1), 5.34–5.08 (m, 6H, CH=C*H*$_2$), 5.00 (d, *J* = 3.0 Hz, H-1'), 4.60 (dd, *J* = 8.0, 2.4 Hz, 1H, H-3), 4.35 (ddt, *J* = 12.7, 5.6, 1.2 Hz, 1H, OC*H*HCH=CH_2), 4.31 (dd, *J* = 5.0, 2.4 Hz, 1H, H-2), 4.23 (dd, *J* = 7.9, 1.9 Hz, 1H, H-4), 4.15–3.93 (m, 6H, 5 × OCH*H*CH=CH_2, H-5), 3.91 (app t, *J* = 6.7 Hz, 1H, H-5'), 3.83–3.77 (m, 2H, H-3', H-4'), 3.74 (ddd, *J* = 10.6, 6.7 Hz, 1H, H-6a), 3.68–3.59 (m, 2H, H-6a', H-6b), 3.51 (1H, dd, *J* = 9.3, 5.6 Hz, H-6b'), 2.10 (td, *J* = 12.2, 3.5 Hz, 1H, H-2a'), 1.96 – 1.87 (m, 1H, H-2b'), 1.53 (s, 3H, CH_3), 1.43 (s, 3H, CH_3), 1.33 (s, 6H, 2 × CH_3); ^{13}C NMR (101 MHz, $CDCl_3$) δ: 135.8 (*C*H=CH_2), 135.0 (*C*H=CH_2), 134.7 (*C*H=CH_2), 117.2 (CH=*C*H_2), 116.9 (CH=*C*H_2), 116.6 (CH=*C*H_2), 109.4 ($O_2C(CH_3)_2$), 108.7 ($O_2C(CH_3)_2$), 97.7 (C-1'), 96.5 (C-1), 74.1 (C-3'), 73.7 (O*C*H_2CH), 72.6, 72.4 (C-4', O*C*H_2CH), 71.2 (C-4), 70.81, 70.77 (C-2, C-3), 69.7 (C-5'), 69.4 (O*C*H_2CH), 69.1 (C-6'), 66.1 (C-5), 65.7 (C-6), 31.3 (C-2'), 26.2 (CH_3), 26.1 (CH_3), 25.1 (CH_3), 24.7 (CH_3). Data are consistent with literature.[5]

6-*O*-(6-*O*-Acetyl-3,4-di-*O*-benzyl-2-deoxy-α-D-lyxo-hexapyranosyl)-1,2:3,4-di-*O*-isopropylidene-α-D-galactopyranose 5c

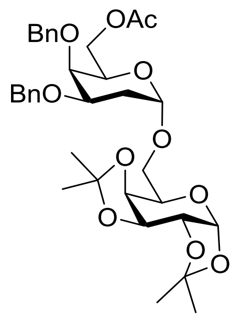

Following General Procedure 1, galactal **3c** (222 mg, 0.603 mmol), an anhydrous CH_2Cl_2 solution of galactose **4** (0.83 M, 0.6 ml, 0.5 mmol), and 2-thiouracil (0.6 mg, 5 μmol) were used. Following purification by column chromatography (pentane:EtOAc; 3:1), the product was obtained as a pale yellow oil (267 mg, 85%).

R_f = 0.3 (pentane:EtOAc; 3:1); ^{1}H NMR (500 MHz; $CDCl_3$): δ 7.38-7.24 (m, 10H, Ph), 5.51 (d, *J* = 5.0 Hz, 1H, H-1), 5.04 (d, *J* = 2.9 Hz, 1H, H-1'), 4.95 (d, *J* = 11.7 Hz, 1H, OC*H*HPh), 4.65 (d, *J* = 11.7 Hz, 1H, OC*H*HPh), 4.63-4.59 (m, 3H, H-3, OCH_2Ph), 4.30 (dd, *J* = 5.0, 2.4 Hz, 1H, H-2), 4.21 (dd, *J* = 7.9, 1.9 Hz, 1H, H-4), 4.15 (dd, *J* = 11.2, 7.1 Hz, 1H, H-6a'), 4.11 (dd, *J* = 11.2, 5.6 Hz, 1H, H-6b'), 3.97-3.92 (m, 3H, H-5, H-3', H-5'), 3.83 (br s, 1H, H-4'), 3.73 (dd, *J* = 10.7, 6.9 Hz, 1H, H-6a), 3.64 (dd, *J* = 10.7, 3.7 Hz, 1H, H-6b), 2.22 (td, *J* = 12.3, 3.7 Hz, 1H, H-2a'), 2.06-2.01 (m, 1H, H-2b'), 1.98 (s, 3H, OC(O)CH_3), 1.51 (s, 3H, CH_3), 1.43 (s, 3H, CH_3), 1.330 (s, 3H, CH_3), 1.325 (s, 3H, CH_3); ^{13}C NMR (125 MHz; $CDCl_3$): δ 170.8 (C=O), 138.62 (4° C), 138.58 (4° C), 128.52 (CH), 128.49 (CH), 128.4 (CH), 127.8 (CH), 127.76 (CH), 127.4 (CH), 109.4 (O_2*C*Me_2), 108.6 (O_2*C*Me_2), 97.5 (C-1'), 96.4 (C-1), 74.9 (CH), 74.1 (PhCH_2), 72.7 (C-4'), 71.3 (C-4), 70.8, 70.68, 70.66 (PhCH_2, C-2, C-3), 69.1 (CH), 66.2 (CH), 65.8 (C-6), 64.1 (C-6'), 31.0 (C-2'), 26.2 (CH_3), 26.1 (CH_3), 25.1 (CH_3), 24.6 (CH_3), 21.0 (OC(O)*C*H_3). Spectra were consistent with literature data.[5]

6-*O*-(3,4,6-Tri-*O*-*tert*-butyldimethylsilyl-2-deoxy-α-D-lyxo-hexapyranosyl)-1,2:3,4-di-*O*-isopropylidene-α-D-galactopyranose 5d

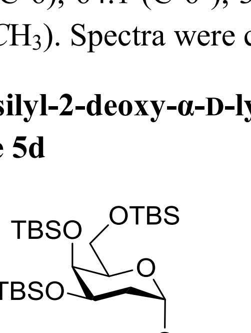

Following General Procedure 1, galactal **3d** (113 mg, 0.231 mmol), an anhydrous 2-Me-THF solution of galactose **4** (0.83 M, 0.24 ml, 0.20 mmol), and 2-thiouracil (0.2 mg, 2 μmol) were

used. Following purification by column chromatography (cyclohexane:EtOAc; 19:1), the product was obtained as a pale yellow oil (102 mg, 68%).

R_f = 0.25 (cyclohexane:EtOAc; 19:1); ^{1}H NMR (300 MHz; $CDCl_3$): δ 5.50 (d, J = 5.0 Hz, 1H, H-1), 4.91 (d, J = 3.1 Hz, 1H, H-1'), 4.60 (dd, J = 7.9, 2.4 Hz, 1H, H-3), 4.30 (dd, J = 5.0, 2.4 Hz, 1H, H-2), 4.20 (dd, J = 7.9, 1.9 Hz, 1H, H-4), 4.09-4.01 (m, 1H, H-3'), 3.97-3.87 (m, 1H, H-5), 3.84 (br s, 1H, H-4'), 3.75 (dd, J = 10.3, 6.9 Hz, 1H, H-6a), 3.71-3.65 (m, 2H, H-5', H-6a'), 3.64-3.53 (m, 2H, H-6b, H-6b'), 2.09 (td, J = 12.0, 3.6 Hz, 1H, H-2a'), 1.62 (dd, J = 12.4, 4.3 Hz, 1H, H-2b'), 1.52 (s, 3H, CH_3), 1.43 (s, 3H, CH_3), 1.33 (6H, CH_3 × 2), 0.905, 0.899, 0.886 (s, 27H, $SiC(CH_3)_3$), 0.10 (s, 3H, $SiCH_3$), 0.082 (s, 3H, $SiCH_3$), 0.077 (s, 3H, $SiCH_3$), 0.071 (s, 3H, $SiCH_3$), 0.05 (s, 6H, $SiCH_3$ × 2); ^{13}C NMR (125 MHz; $CDCl_3$): δ 109.4 (O_2CMe$_2$), 108.6 (O_2CMe$_2$), 97.6 (C-1'), 96.5 (C-1), 72.6 (C-5'), 71.4 (C-4), 70.9 (C-2, C-3), 70.1 (C-4'), 68.4 (C-3'), 66.7 (C-5), 65.4 (C-6), 62.2 (C-6'), 33.8 (C-2'), 26.39 (SiC(CH$_3$)$_3$), 26.31 (SiC(CH$_3$)$_3$), 26.27 (CH_3), 26.1 (CH_3), 26.0 (SiC(CH$_3$)$_3$), 25.2 (CH_3), 24.6 (CH_3), 18.8 (SiC(CH$_3$)$_3$), 18.7 (SiC(CH$_3$)$_3$), 18.4 (SiC(CH$_3$)$_3$), -3.8 (SiCH$_3$), -4.2 (SiCH$_3$), -4.6 (SiCH$_3$), -4.8 (SiCH$_3$), -5.1 (SiCH$_3$), -5.2 (SiCH$_3$). Spectra were consistent with literature data.[5]

6-*O*-(3,4,6-Tri-*O*-benzyl-2-deoxy-α/β-D-*erythro*-hexapyranosyl)-1,2:3,4-di-*O*-isopropylidene-α-D-galactopyranose 5fa and 6-*O*-(4,6-di-*O*-benzyl-2,3-dideoxy-α/β-D-*erythro*-hex-2-enopyranosyl)-1,2:3,4-di-*O*-isopropylidene-α-D-galactopyranose 5fb

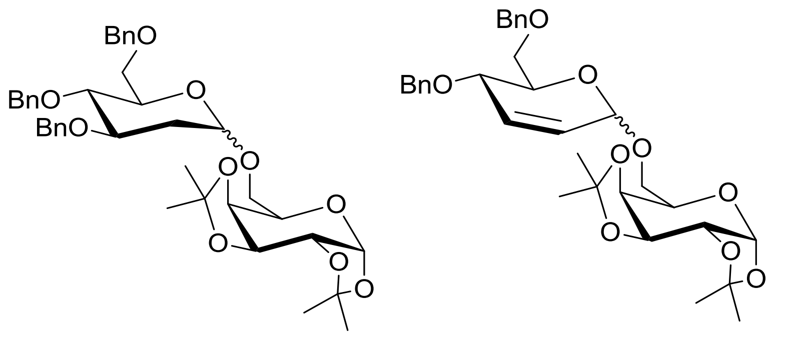

5fa **5fb**

Following General Procedure 1, glucal **3f** (290 mg, 0.70 mmol), an anhydrous CH_2Cl_2 solution of galactose **4** (0.83 M, 0.7 ml, 0.58 mmol) and 2-thiouracil (0.8 mg, 6 μmol) were used. Analysis of the ^{1}H NMR spectrum of the reaction material prior to column chromatography gave an α:β 6:1. Following purification by column chromatography (cyclohexane:EtOAc; 5:1) the products were inseparable. **5fa** and **5fb** were obtained as a white foam 236 mg (^{1}H NMR of the white foam showed **5fa:5fb** = 4.6:1), yield of **5fa** 46%. **5fa**: α/β: 5:1**;** **5fb**: α/β: 5:1

R_f= 0.3 (cyclohexane:EtOAc; 5:1);

^{1}H NMR (500 MHz; $CDCl_3$):

The following were observed for all four diastereomers: δ 7.38-7.17 (m, 15 H).

5fa α-anomer: δ 5.51 (d, *J* = 5.0 Hz, 1H, H-1), 5.02 (br d, *J* = 2.8 Hz, 1H, H-1'), 4.88 (d, *J* = 10.8 Hz, 1H, OCH*H*Ph), 4.68-4.63 (m, 3H, OCH*H*Ph, OCH_2Ph), 4.59 (m, 1H, H-3), 4.51 (app t, *J* = 11.4, 2H, 2 x OCH*H*Ph), 4.30 (dd, *J* = 5.0, 2.4 Hz, 1H, H-2), 4.22 (dd, *J* = 7.9, 1.9 Hz, 1H, H-4), 4.01-3.96 (m, 1H, H-3'), 3.94 (td, *J* = 6.8, 1.7 Hz, 1H, H-5), 3.82-3.76 (m, 2H, H-5', H-6a'), 3.73 (dd, *J* = 10.4, 6.6 Hz, 1H, H-6a), 3.68-3.62 (m, 3H, H-4', H-6b, H-6b'), 2.32 (ddd, *J* = 13.0, 5.1, 1.4 Hz, 1H, H-2a'), 1.72 (ddd, *J* = 12.9, 11.6, 3.7 Hz, 1H, H-2b'), 1.51 (s, 3H, CH_3), 1.43 (s, 3H, CH_3), 1.33 (s, 3H, CH_3), 1.32 (s, 3H, CH_3).

5fa β-anomer (selected signals): δ 4.91 (d, *J* = 10.7 Hz, 1H, OCH*H*Ph), 4.08 (dd, *J* = 11.0 Hz, 1H, OCH*H*Ph), 3.53 (t, *J* = 9.2 Hz, 1H, CH), 3.39 (ddd, *J* = 9.5, 4.3, 2.3 Hz, 1H, CH), 2.45 (ddd, *J* = 12.5, 5.0, 1.6 Hz, 1H, H-2a'), 1.69-1.62 (m, 1H, H-2b').

5fb α-anomer (selected signals): δ 6.06 (d, *J* = 10.2 Hz, 1H, H-2'), 5.77 (ddd, *J* = 10.2, 2.6, 2.0 Hz, 1H, H-3'), 5.55 (d, *J* = 5.7 Hz, 1H, H-1), 5.09 (br s, 1H, H-1'), 4.43 (d, *J* = 11.5 Hz, 1H, OC*H*HPh), 4.27 (dd, *J* = 7.9, 1.9 Hz, 1H), 3.86 (dd, *J* = 10.2, 6.1 Hz, 1H, H-6a).

5fb β-anomer (selected signals): δ 6.02 (ddd, *J* = 10.3, 2.9, 1.7 Hz, 1H, CH), 5.90 (dt, *J* = 10.1, 1.3 Hz, 1H, CH), 5.20 (dd, *J* = 2.8, 1.3 Hz, 1H), 4.12 (d, *J* = 7.2 Hz, 1H).

^{13}C NMR (126 MHz; $CDCl_3$):

5fa α-anomer: δ 138.8 (4°), 138.6 (4°), 138.2 (4°), 128.4 (CH), 128.3 (CH), 128.3 (CH), 127.95 (CH), 127.91 (CH), 127.6 (CH), 127.69 (CH), 109.3 (O_2*C*Me_2), 108.6 (O_2*C*Me_2), 97.3 (C-1'), 96.3 (C-1), 78.2 (C-4'), 77.6 (C-3'), 75.0 (Ph*C*H_2), 73.5 (Ph*C*H_2), 71.8 (Ph*C*H_2), 70.97 (C-4/C-5'), 70.95 (C-4/C-5'), 70.65 (C-2/C-3), 70.64 (C-2/C-3), 68.8 (C-6'), 65.7 (C-5), 65.4 (C-6), 35.4 (C-2'), 26.2 (CH_3), 26.0 (CH_3), 24.9 (CH_3), 24.6 (CH_3).

5fa β-anomer (selected signals): δ 100.4 (C-1'), 78.0 (CH), 75.1 (CH), 74.9 (Ph*C*H_2), 68.8 (Ph*C*H_2), 36.6 (C-2').

5fb α-anomer (selected signals): δ 130.7 (C-2'), 126.5 (C-3'), 108.7 (O_2*C*Me_2), 96.4 (C-1), 95.1 (C-1'), 71.2 (Ph*C*H_2), 70.8 (CH), 70.2 (C-5'), 66.8 (C-6).

5fb β-anomer from HSQC (selected signals): δ 129.2 (C-2'), 128.3 (C-3'), 96.4 (C-1').

Spectra were consistent with literature data; α-**5fa** and β-**5fa**;[17] α-**5fb** and **5fb**.[18]

(3,4-Di-*O*-benzyl-2,6-dideoxy-α/β-L-*erythro*-hexapyranosyl)-(1→6)-1,2:3,4-di-*O*-isopropylidene-α-D-galactopyranoside 5ga and 4-*O*-(benzyl)-2,3,6-trideoxy-α/β-L-hex-2-enopyranosyl-(1→6)-1,2;3,4-di-*O*-isopropylidene-α-D-galactopyranoside 5gb

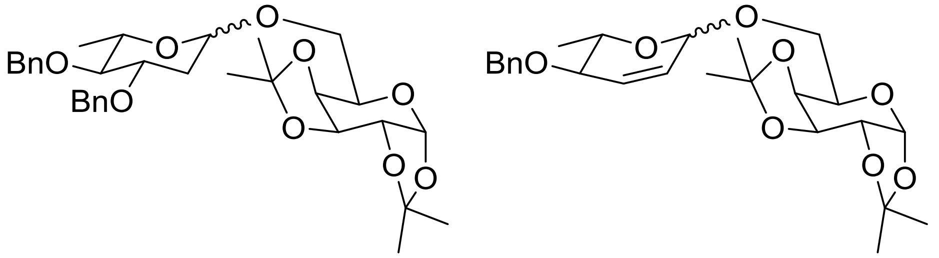

Following General Procedure 3, rhamnal **3g** (100 mg, 0.32 mmol), an anhydrous CH_2Cl_2 solution of galactose acceptor **4** (1.7 M, 0.16 mL, 0.27 mmol) and 2-thiouracil (0.3 mg, 2 μmol) were used. The reaction mixture was heated at reflux for 40 h. Analysis of the ^{1}H NMR spectrum of the crude reaction mixture gave an α:β of 81:19 for **5ga**. Following purification by column chromatography (95:5 to 90:10 cyclohexane/ethyl acetate) the products **5ga** and **5gb** were inseparable and a cloudy syrup (84 mg) was obtained (^{1}H NMR spectrum of the syrup showed **5ga**:**5gb** = 74:26), yield of **5ga** 40% (α:β =81:19) and yield of **5gb** (major:minor = 83:17)

^{1}H NMR (600 MHz, Chloroform-*d*): The following were observed for all four diastereomers: δ 7.37 – 7.26 (m, 10H).

5ga α-anomer: δ 5.53 (dd, *J* = 5.1 Hz, 1H, H-1), 4.94 (d, *J* = 11.0 Hz, 1H, OC*H*HPh), 4.92 (d, *J* = 3.1 Hz, 1H, H-1'), 4.68–4.63 (m, 2H, 2 × OC*H*HPh), 4.62–4.58 (m, 2H, OC*H*HPh, H-3), 4.30 (dd, *J* = 5.1, 2.5 Hz, 1H, H-2), 4.22 (dd, *J* = 7.9, 1.9 Hz, 1H, H-4), 3.97–3.90 (m, 2H, H-5, H-3'), 3.81–3.77 (m, 2H, H-6a, H-5'), 3.53 (dd, *J* = 10.4, 6.8 Hz, 1H, H-6b), 3.13 (t, *J* = 9.2 Hz, 1H, H-4'), 2.35 (ddd, *J* = 13.1, 5.0, 1.1 Hz, 1H, H-2a'), 1.66 (ddd, *J* = 12.9, 11.4, 3.7 Hz, 1H, H-2b'), 1.54 (s, 3H, CH_3), 1.44 (s, 3H, CH_3), 1.33 (s, 3H CH_3), 1.32 (s, 3H, CH_3), 1.27 (d, *J* = 6.3 Hz, 3H, 6'-CH_3). Data were in agreement with literature.[19]

5ga β-anomer (selected signals): δ 5.51 (d, *J* = 5.1 Hz, 1H, H-1), 4.94 (d, *J* = 11.0 Hz, 1H, OC*H*HPh), 4.47 (dd, *J* = 9.8, 1.8 Hz, 1H, H-1'), 4.02 (ddd, *J* = 8.0, 5.9, 2.1 Hz, 1H, H-5), 3.86 (dd, *J* = 10.1, 6.0 Hz, 1H, H-6a), 3.77–3.73 (m, 1H, H-6b), 3.62–3.58 (m, 1H, CH), 3.32 (dq, *J* = 9.3, 6.2 Hz, 1H, H-5'), 3.12 (t, *J* = 8.9 Hz, 1H, H-4'), 2.37 (m, 1H, H-2a'), 1.70–1.58 (m, 1H, H-2b'), 1.53 (s, 3H), 1.44 (s, 3H), 1.35 (s, 3H), 1.31 (d, *J* = 6.2 Hz, 3H, 6'-CH_3). Data were in agreement with literature.[20]

The following were observed for both the major and minor products of **5gb**: δ 6.04 (dt, *J* = 10.3, 1.3 Hz, 1H, CH=CH), 4.27 (dd, *J* = 8.0, 1.5 Hz, 1H, CH), 3.98–3.90 (m, 2H, H-6a, H-5'), 3.70 (dd, *J* = 9.1, 1.7 Hz, 1H, H-4'), 3.67–3.62 (m, 1H, H-6b), 1.29 (d, *J* = 6.3 Hz, 3H, 6'-CH_3).

5gb major (selected signals): δ 5.79 (dt, *J* = 10.2, 2.3 Hz, 1H, CH=CH), 4.99 (br s, 1H, H-1').

5gb minor (selected signals): δ 5.83 (dd, *J* = 10.3, 1.4 Hz, 1H, CH=CH), 5.20 (br d, *J* = 1.7 Hz, 1H, H-1').

^{13}C NMR (151 MHz, Chloroform-*d*):

5ga α-anomer: δ 138.9 (4° C), 138.8 (4° C), 128.48 (CH), 128.46 (CH), 128.0 (CH), 127.8 (CH), 109.39 ($O_2C(CH_3)_2$), 108.69 ($O_2C(CH_3)_2$), 97.4 (C-1'), 96.4 (C-1), 84.4 (C-4'), 77.5 (C-3'), 75.2 (Ph*C*H$_2$), 71.8 (Ph*C*H$_2$), 71.3 (C-4), 70.77 (C-2 or C-3), 70.73 (C-2 or C-3), 67.4 (C-5), 67.3 (C-5'), 65.7 (C-6), 35.8 (C-2'), 26.3 (CH_3), 26.1 (CH_3), 25.1 (CH_3), 24.6 (CH_3), 18.2 (C-6').

5ga β-anomer (selected signals): δ 109.30 ($O_2C(CH_3)_2$), 108.66 ($O_2C(CH_3)_2$), 100.3 (C-1'), 96.5 (C-1), 83.8 (C-4'), 79.4, 75.4 (Ph*C*H$_2$), 71.5 (C-5'), 67.7 (C-6), 66.0 (C-5), 37.0 (C-2'), 18.3 (C-6').

The following were observed for both the major and minor products of **5gb**: δ 130.7 (C-3'), 76.6 (C-4'), 71.26 (CH), 66.4 (C-6), 65.9 (C-5'), 18.31 (C-6').

5gb major (selected signals): 127.0 (C-2'), 94.5 (C-1')

5gb minor (selected signals): 97.4 (C-1')

HRMS-ESI (*m/z*): $[M + Na]^+$ calc'd for $C_{32}H_{42}O_9Na$ (**5ga**+Na^+), 593.2727; found 593.2709; calc'd for $C_{25}H_{34}O_8Na$ (**5gb**+Na^+): 485.2151; found: 485.2175.

(3,4-Di-*O*-benzyl-2-deoxy-α/β-L-fucopyranosyl)-(1→6)-1,2:3,4-di-*O*-isopropylidene-α-D-galactopyranoside 5h and 3,4-Di-*O*-benzyl-2-deoxy-α/β-L-fucopyranosyl-(1→1)-(3',4'-Di-*O*-benzyl-2-deoxy-α/β-L-fucopyranosyl 9h

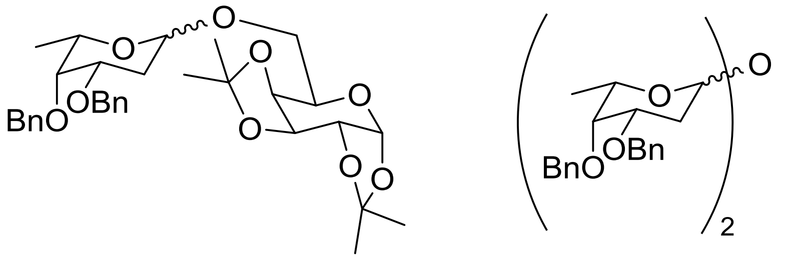

Following General Procedure 3, fucal **3h** (102 mg, 0.329 mmol), an anhydrous CH_2Cl_2 solution of galactose acceptor **4** (1.7 M, 0.16 mL, 0.27 mmol) and 2-thiouracil (0.3 mg, 2 μmol) were used. The reaction mixture was heated at reflux for 18 h. Analysis of the ^1H NMR spectrum of the crude reaction mixture gave an α:β of 92:8. Following purification by column chromatography (95:5 to 80:10 pentane/ethyl acetate) the products **α-5h** and **α,α-9h** were inseparable and a cloudy syrup (92 mg) was obtained (^1H NMR spectrum of the syrup showed **α-5h**:**α,α-9h** = 89:11); yield of **α-5h** 52%. **β-5h** was isolated with an inseparable unidentified impurity as a syrup (8 mg, 4%).

^1H NMR (400 MHz, Chloroform-*d*): The following were observed for all four diastereomers: δ 7.44 – 7.20 (m, 10H, Ph).

5h α-anomer: δ 5.53 (d, *J* = 5.0 Hz, 1H, H-1), 5.00 (d, *J* = 3.1 Hz, 1H, H-1'), 4.97 (d, *J* = 11.8 Hz, 1H, OC*H*HPh), 4.69 (d, *J* = 11.8 Hz, 1H, OC*H*HPh), 4.62 (d, *J* = 11.9 Hz, 1H,

OC*H*HPh), 4.59 (dd, *J* = 7.9, 2.5 Hz, 1H, H-3), 4.58 (d, *J* = 11.9 Hz, 1H, OC*H*HPh), 4.31 (dd, *J* = 5.0, 2.4 Hz, 1H, H-2), 4.20 (dd, *J* = 7.9, 1.9 Hz, 1H, H-4), 3.96 – 3.84 (m, 3H, H-5, H-3', H-5'), 3.77 (dd, *J* = 10.1, 6.4 Hz, 1H, H-6a), 3.59 (br s, 1H, H-4'), 3.55 (dd, *J* = 10.1, 6.7 Hz, 1H, H-6b), 2.18 (td, *J* = 12.3, 3.7 Hz, 1H, H-2a'), 2.03 (dd, *J* = 12.6, 4.7 Hz, 1H, H-2b'), 1.53 (s, 3H, CH_3), 1.42 (s, 3H, CH_3), 1.33 (s, 3H, CH_3), 1.30 (s, 3H, CH_3), 1.16 (d, *J* = 6.5 Hz, 3H, 6'-CH_3).

5h β-anomer (impure): ^{1}H NMR (600 MHz, Chloroform-*d*): δ 5.49 (d, *J* = 5.0 Hz, 1H, H-1), 4.96 (d, *J* = 11.9 Hz, 1H, OC*H*HPh), 4.72 (d, *J* = 11.9 Hz, 1H, OC*H*HPh), 4.62 (d, *J* = 12.3 Hz, 1H, OC*H*HPh), 4.60–4.58 (m, 1H, H-3 or H-4), 4.56 (d, *J* = 12.2 Hz, 1H, OC*H*HPh), 4.41 (dd, *J* = 9.5, 2.2 Hz, 1H, H-1'), 4.37 (dd, *J* = 8.0, 1.6 Hz, 1H, H-3 or H-4), 4.28 (dd, *J* = 5.0, 2.3 Hz, 1H, H-2), 4.06–4.02 (m, 1H, H-5), 3.86 (dd, *J* = 9.8, 5.5 Hz, 1H, H-6a), 3.76 (t, *J* = 9.4 Hz, 1H, H-6b), 3.53 (ddd, *J* = 11.9, 4.5, 2.6 Hz, 1H, H-3'), 3.48 (br s, 1H, H-4'), 3.35 (q, *J* = 6.4 Hz, 1H, H-5'), 2.11–2.07 (m, 1H, H-2a'), 2.04 (td, *J* = 11.9, 9.4 Hz, 1H, H-2b'), 1.51 (s, 3H, CH_3), 1.44 (s, 3H, CH_3), 1.35 (s, 3H, CH_3), 1.31 (s, 3H, CH_3), 1.18 (d, *J* = 6.3 Hz, 3H, 6'-CH_3).

9h α,α-anomer (selected signals): δ 5.43 (d, *J* = 4.5 Hz, 1H, H-1), 4.05–3.99 (m, 1H, H-5), 2.30–2.23 (m, 1H, H-2a), 2.00–1.94 (m, 1H, H-2b), 1.19 (d, *J* = 6.5 Hz, 1H, 6-CH_3).

^{13}C NMR:

5h α-anomer (101 MHz, Chloroform-*d*): δ 139.1 (4º C), 138.8 (4º C), 128.49 (CH), 128.48 (CH), 128.3 (CH), 127.58 (CH), 127.57 (CH), 127.4 (CH), 109.3 ($O_2C(CH_3)_2$), 108.6 ($O_2C(CH_3)_2$), 97.9 (C-1'), 96.4 (C-1), 75.9 (C-4'), 75.4 (C-3'), 74.4 (Ph*C*H_2), 71.3 (C-4), 70.76 (C-3), 70.73 (C-2), 70.5 (Ph*C*H_2), 66.9 (C-5'), 66.8 (C-5), 65.4 (C-6), 30.6 (C-2'), 26.2 (CH_3), 26.1 (CH_3), 25.1 (CH_3), 24.7 (CH_3), 17.4 (C-6').

5h β-anomer (impure) (151 MHz, Chloroform-*d*): δ 138.9 (4º C), 138.5 (4º C), 128.7 (CH), 128.6 (CH), 128.5 (CH), 128.3 (CH), 127.7 (CH), 127.6 (CH), 127.4 (CH), 109.1 ($O_2C(CH_3)_2$), 108.7 ($O_2C(CH_3)_2$), 101.3 (C-1'), 96.4 (C-1), 78.1 (C-3'), 74.42 (Ph*C*H_2), 74.38 (C-4'), 71.01 (C-5'), 70.97 (C-2), 70.7 (C-3 or C-4), 70.6 (C-3 or C-4), 70.4 (Ph*C*H_2), 67.5 (C-6), 65.9 (C-5), 32.3 (C-2'), 26.3 (CH_3), 26.1 (CH_3), 25.1 (CH_3), 24.6 (CH_3), 17.4 (C-6').

9h α,α-anomer (101 MHz, Chloroform-*d*) (selected signals): δ 138.9 (4º C), 138.5 (4º C), 128.55 (CH), 128.43 (CH), 128.31 (CH), 127.70 (CH), 127.65 (CH), 127.37 (CH), 100.2 (C-1), 75.7 (CH), 74.8 (CH), 74.6 (Ph*C*H_2), 70.6 (Ph*C*H_2), 67.7 (C-5), 28.5 (C-2), 17.6 (C-6).

NMR data for **5h** were in agreement with literature.[21]

Table 2

Methyl 3-*O*-benzyl-2-*O*-(3,4,6-tri-*O*-benzyl-2-deoxy-α-D-lyxo-hexapyranosyl)-4,6-*O*-benzylidene-α-D-glucopyranoside 7a

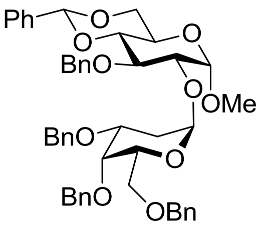

In a slight modification to the general procedure 1 (due to the low solubility of **6a**), galactal **3a** (290 mg, 0.69 mmol), an anhydrous CH_2Cl_2 solution of glucose **6a** (0.35M, 1.0 ml, 0.35 mmol) and 2-thiouracil (0.6 mg, 5 μmol) were used. Following purification by column chromatography (4:1; cyclohexane/ethyl acetate) the product **7a** was obtained as a white solid (232 mg, 84% yield).

R_f = 0.18 (4:1; cyclohexane/ethyl acetate); ^{1}H NMR (400 MHz, Chloroform-*d*) δ 7.53 – 7.42 (m, 2H, Ph), 7.41 – 7.14 (m, 23H, Ph), 5.51 (s, 1H, H-7), 5.14 (app d, *J* = 3.4 Hz, 1H, H-1'), 4.95 – 4.88 (m, 2H, H-1, OC*H*HPh), 4.85 (d, *J* = 11.4 Hz, 1H, OC*H*HPh), 4.65 (d, *J* = 11.4 Hz, 1H, OC*H*HPh), 4.58 (d, *J* = 11.5 Hz, 1H, OC*H*HPh), 4.52 (s, 2H, 2 × OC*H*HPh), 4.37 (s, 2H, 2 × OC*H*HPh), 4.27 (dd, *J* = 10.1, 4.7 Hz, 1H, H-6a), 4.19 (t, *J* = 6.5 Hz, 1H, H-5'), 3.97 – 3.85 (m, 3H, H-2, H-3, H-3'), 3.80 (td, *J* = 9.9, 4.7 Hz, 1H, H-5), 3.75 (br s, 1H, H-4'), 3.69 (t, *J* = 10.2 Hz, 1H, H-6b), 3.58 (dd, *J* = 9.7, 6.6 Hz, 1H, H-6a'), 3.54 (t, *J* = 9.0 Hz, 1H, H-4), 3.45 (dd, *J* = 9.7, 6.5 Hz, 1H, H-6b'), 3.42 (s, 3H, OCH_3), 2.25 (td, *J* = 12.4, 3.6 Hz, 1H, H-2a'), 2.07 (dd, *J* = 12.7, 4.6 Hz, 1H, H2b'); ^{13}C NMR (101 MHz, Chloroform-*d*) δ 138.9 (4° C), 138.8 (4° C), 138.6 (4° C), 138.53 (4° C), 137.49 (4° C), 129.0 (CH), 128.6 (CH), 128.34 (CH), 128.32 (CH), 128.31 (CH), 128.30 (CH), 128.1 (CH), 127.7 (CH), 127.63 (CH), 127.61 (CH), 127.5 (CH), 127.4 (CH), 126.1 (CH), 101.3 (C-7), 97.3 (C-1), 94.1 (C-1'), 82.3 (C-4), 77.4 (C-3), 75.5 ($PhCH_2$), 74.54, 74.45 (C-3', $PhCH_2$), 73.1, 72.92, 72.91 (C-2, C-4', $PhCH_2$), 70.5 ($PhCH_2$), 69.64, 69.57 (C-5', C-6'), 69.1 (C-6), 62.5 (C-5), 55.3 (OCH_3), 31.0 (C-2'). Spectra were consistent with literature data.[5]

Methyl 2-*O*-benzyl-3-*O*-(3,4,6-tri-*O*-benzyl-2-deoxy-α-D-lyxo-hexapyranosyl)-4,6-*O*-benzylidene-α-D-glucopyranoside 7b

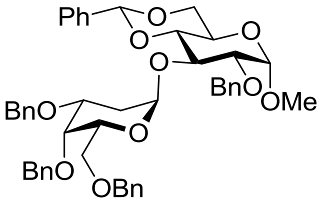

Following general procedure 1, galactal **3a** (250 mg, 0.60 mmol), an anhydrous CH_2Cl_2 solution of glucose **6b** (0.83M, 0.6 ml, 0.50 mmol) and 2-thiouracil (0.6 mg, 5 μmol) were used. Following purification by column chromatography (81:18; cyclohexane/ethyl acetate) the product **7b** was obtained as a white solid (352 mg, 89% yield).

R_f = 0.32 (82:18; cyclohexane/ethyl acetate); ^{1}H NMR (400 MHz, Chloroform-*d*) δ 7.45 – 7.38 (m, 2H, Ph), 7.37 – 7.18 (m, 23H, Ph), 5.52 (app d, *J* = 3.4 Hz, 1H, H-1'), 5.49 (s, 1H, H-7), 4.89 (d, *J* = 11.5 Hz, 1H, OC*H*HPh), 4.69 (d, *J* = 12.2 Hz, 1H, OC*H*HPh), 4.61 (d, *J* = 11.5 Hz, 1H, OC*H*HPh), 4.58 – 4.53 (m, 2H, 2 × OC*H*HPh), 4.52 (d, *J* = 3.7 Hz, 1H, H-1), 4.49 (d, *J* = 12.3 Hz, 1H, OC*H*HPh), 4.42 (d, *J* = 11.7 Hz, 1H, OC*H*HPh), 4.37 (d, *J* = 11.7 Hz, 1H, OC*H*HPh), 4.33 – 4.25 (m, 2H, H-3, H-5'), 4.22 (dd, *J* = 10.0, 4.7 Hz, 1H, H-6a), 3.96 – 3.87 (m, 2H, H-3', H-4'), 3.78 (app td, *J* = 9.9, 4.7 Hz, 1H, H-5), 3.66 (t, *J* = 10.3 Hz, H-6b), 3.65 – 3.58 (m, 2H, H-6a' H-6b'), 3.54 (t, *J* = 9.4 Hz, 1H, H-4), 3.43 (dd, *J* = 9.5, 3.7 Hz, 1H, H-2), 3.32 (s, 3H, OCH_3), 2.20 (app td, *J* = 12.3, 3.8 Hz, 1H, H-2a'), 2.06 – 1.99 (m, 1H, H-2b'); ^{13}C NMR (101 MHz, Chloroform-*d*) δ 139.1 (4° C), 138.7 (4° C), 138.6 (4° C), 138.2 (4° C), 137.3 (4° C), 129.1 (CH), 128.6 (CH), 128.5 (CH), 128.37 (CH), 128.36 (CH), 128.29 (CH), 128.27 (CH), 128.1 (CH), 128.0 (CH), 127.61 (CH), 127.57 (CH), 127.5 (CH), 127.4 (CH), 126.1 (CH), 101.4 (C-7), 99.1 (C-1), 97.9 (C-1'), 83.1 (C-4), 78.2 (C-2), 74.6, 74.4 (C-3', Ph*C*H_2), 73.6 (Ph*C*H_2), 73.2, 73.1 (C-4', Ph*C*H_2), 72.6 (C-3), 70.2 (Ph*C*H_2), 69.6 (C-5'), 69.2, 69.1 (C-6, C-6'), 62.1 (C-5), 55.3 (OCH_3), 31.3 (C-2'). Spectra were consistent with literature data.[5]

Methyl 2,3,6-tri-*O*-benzyl-4-*O*-(3,4,6-tri-*O*-benzyl-2-deoxy-α-D-lyxo-hexapyranosyl)-α-D-glucopyranoside 7c

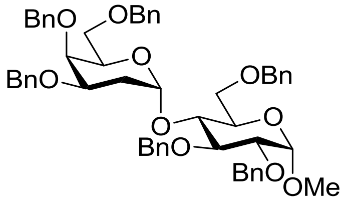

In a slight modification to the general procedure, galactal **3a** (259 mg, 0.62 mmol, 2 eq), an anhydrous CH_2Cl_2 solution of glucose **6c** (0.83M, 0.35 ml, 0.29 mmol) and 2-thiouracil (0.4 mg, 3 μmol) were used. After 18 h ^{1}H NMR showed the acceptor had been completely consumed in the reaction. Column chromatography (85:15; cyclohexane/ethyl acetate) was carried out, however the desired product **7c** could not be isolated from a close running impurity (R_f = 0.28 (impurity), 0.23 (product)). Column chromatography was repeated but desired product **7c** free of impurity α,α-**9a** was not isolated. The product **7c** was obtained as a cloudy oil (184 mg, 55% yield), containing α,α-**9a** (17%). This result was reproduced several times and each time desired product **7c** was isolated but contained α,α-**9a** as an impurity.

R_f = 0.23 (85:15; cyclohexane/ethyl acetate); ^{1}H NMR (400 MHz, Chloroform-*d*) δ 7.38 – 7.09 (m, 30H), 5.47 (d, *J* = 3.7 Hz, 1H, H-1'), 5.00 (d, *J* = 10.9 Hz, 1H, OC*H*HPh), 4.88 (d, *J* = 11.6 Hz, 1H, OC*H*HPh), 4.73 (d, *J* = 12.0 Hz, 1H, OC*H*HPh), 4.65 (d, *J* = 11.0 Hz, 1H, OC*H*HPh), 4.63 – 4.56 (m, 3H, H-1, 2 × OC*H*HPh), 4.54 (d, *J* = 11.8 Hz, 1H, OC*H*HPh), 4.52 (d, *J* = 11.9 Hz, 1H, OC*H*HPh), 4.49 (d, *J* = 11.7 Hz, 1H, OC*H*HPh), 4.38 (d, *J* = 12.1 Hz, 1H, OC*H*HPh) 4.37 (d, *J* = 11.7 Hz, 1H, OC*H*HPh), 4.31 (d, *J* = 11.7 Hz, 1H, OC*H*HPh), 3.96 – 3.75 (m, 4H, H-3, H-3', H-4', H-5'), 3.75 – 3.58 (m, 4H, H-4, H-5, H-6a/b), 3.51 (dd, *J* = 9.5, 3.7 Hz, H-2), 3.49 – 3.42 (m, 2H, H-6a'/b'), 3.39 (s, 3H, OCH_3), 2.13 (td, *J* = 12.4, 3.9 Hz, 1H, H-2a'), 1.87 (app, dd, *J* = 12.9, 4.4 Hz, 1H, H-2b'); ^{13}C NMR (101 MHz,

Chloroform-*d*) δ 138.9 (4° C), 138.6 (4° C), 138.56 (4° C), 138.50 (4° C), 138.2 (4° C), 138.15 (4° C), 128.6 (CH), 128.52 (CH), 128.46 (CH), 128.4 (CH), 128.33 (CH), 128.3 (CH), 128.25 (CH), 128.1 (CH), 128.0 (CH), 127.77 (CH), 127.76 (CH), 127.72 (CH), 127.67 (2 × CH), 127.63 (CH), 127.44 (CH), 127.42 (CH), 99.8 (C-1'), 97.9 (C-1), 82.2 (C-3), 80.2 (C-2), 76.0 (C-4), 75.7 (Ph*C*H_2), 74.6 (C-3'), 74.4 (Ph*C*H_2), 73.6 (Ph*C*H_2), 73.4 (Ph*C*H_2), 73.2 (Ph*C*H_2), 72.9 (C-4'), 70.8 (C-5'), 70.52 (Ph*C*H_2), 70.0 (C-5), 69.72, 69.68 (C-6, C-6'), 55.34 (OCH_3), 31.7 (C-2'). Spectra were consistent with literature data.[5]

Phenyl 2,3,4-tri-*O*-benzyl-6-*O*-(3,4,6-tri-*O*-benzyl-2-deoxy-α-D-lyxo-hexapyranosyl)-β-D-thioglucopyranoside 7d

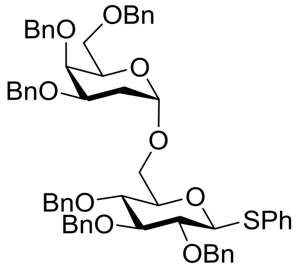

Following General Procedure 2, galactal **3a** (200 mg, 0.48 mmol), acceptor **6d** (0.217 g, 0.40 mmol), anhydrous CH_2Cl_2 (0.48 ml) and 2-thiouracil (0.5 mg, 4 μmol) were used. Following purification by column chromatography (cyclohexane:EtOAc; 4:1), the product was obtained as a white solid (315 mg, 82%).

R_f = 0.3 (cyclohexane:EtOAc; 4:1); ^{1}H NMR (300 MHz; $CDCl_3$): δ 7.56-7.52 (m, 2H, Ph), 7.42-7.16 (m, 33H, Ph), 5.07 (d, *J* = 3.1 Hz, 1H, H-1'), 4.94-4.84 (m, 4H, OCH*H*Ph × 4), 4.79 (d, *J* = 10.9 Hz, 1H, OCH*H*Ph), 4.74 (d, *J* = 10.3 Hz, 1H, OCH*H*Ph), 4.65 (d, *J* = 9.9 Hz, 1H, H-1), 4.61 (d, *J* = 11.9 Hz, 1H, OCH*H*Ph), 4.58 (s, 2H, O*CH*$_2$Ph), 4.53 (d, *J* = 10.9 Hz, 1H, OCH*H*Ph), 4.46 (d, *J* = 11.9, Hz, 1H, OCH*H*Ph), 4.39 (d, *J* = 11.9, Hz, 1H, OCH*H*Ph), 3.93-3.84 (m, 3H, H-3', H-4', H-5'), 3.81 (dd, *J* = 11.5, 4.4 Hz, 1H, H-6a), 3.73-3.65 (m, 2H, H-3, H-6b), 3.56-3.50 (m, 2H, H-6a', H-6b'), 3.50-3.44 (m, 3H, H-2, H-4, H-5), 2.24 (ddd, *J* = 12.7, 11.8, 3.3 Hz, 1H, H-2a'), 2.03 (dd, *J* = 12.7, 4.2 Hz, 1H, H-2b'); ^{13}C NMR (100 MHz; $CDCl_3$): δ 139.0 (4° C), 138.6 (4° C), 138.5 (4° C), 138.4 (4° C), 138.1 (4° C), 138.1 (4° C), 133.8 (4° C), 132.1 (CH), 132.0 (CH), 128.96 (CH), 128.60 (CH), 128.59 (CH), 128.56 (CH), 128.4 (CH), 128.3 (CH), 128.0 (CH), 127.97 (CH), 127.94 (CH), 127.9 (CH), 127.88 (CH), 127.8 (CH), 127.7 (CH), 127.68 (CH), 127.61 (CH), 127.6 (CH), 127.4 (CH), 98.5 (C-1'), 87.2 (C-1), 86.9 (C-3), 80.9 (C-2), 78.6, 78.2 (C-4, C-5), 76.0 ($PhCH_2$), 75.5 ($PhCH_2$), 75.2 ($PhCH_2$), 74.7 (C-3'), 74.4 ($PhCH_2$), 73.4 ($PhCH_2$), 73.1 (C-4'), 70.5 ($PhCH_2$), 70.0 (C-5'), 69.6 (C-6'), 66.3 (C-6), 31.2 (C-2'). Spectra were consistent with literature data.[5]

2-Deoxy-3,4,6-tri-*O*-benzyl-α-D-lyxo-hexapyranosyl-(1→*O*)-*N*-*tert*-butoxycarbonyl-L-serine methyl ester 7e

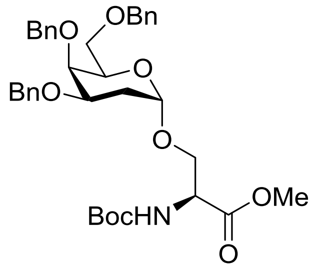

Following General Procedure 2, benzyl galactal **3a** (148 mg, 0.35.5 mmol) and *N*-(*tert*-butoxycarbonyl)-L-serine methyl ester (68 mg, 0.31 mmol) and 2-thiouracil (0.4 mg, 0.003 mmol, 1 mol%) were used, and refluxed in dichloromethane for 18 h. Purification via flash chromatography (toluene:ethyl acetate 18:1, $R_f = 0.35$) afforded **7e** as colourless oil (185 mg, 94%).

^{1}H NMR (500 MHz, $CDCl_3$) δ: 7.40–7.20 (m, 15H, ArH), 5.45 (d, J = 9.0 Hz, 1H, NH), 4.95–4.87 (m, 2H, PhC*H*H and H-1), 4.62–4.55 (m, 3H, 3 × PhC*H*H), 4.52 (d, 1H, J = 11.9 Hz, PhCH*H*), 4.49–4.40 (m, 2H, CHN and PhCH*H*), 3.95–3.79 (m, 5H, H-3, H-4, H-5, CH_2CHN), 3.72 (s, 3H, OMe), 3.62–3.52 (m, 2H, H-6a, H-6b), 2.20 (td, J = 12.4, 3.7 Hz, 1H, H-2a), 1.94 (dd, J = 12.7, 4.5 Hz, 1H, H-2b), 1.44 (s, 9H, Boc-CH_3); ^{13}C NMR (126 MHz, $CDCl_3$) δ: 171.2 (C=O), 155.6 (C=O), 138.9 (4°C), 138.5 (4°C), 138.1 (4°C), 128.52 (CH), 128.49 (CH), 128.31$_4$ (CH), 128.31 (CH), 127.9 (CH), 127.8 (CH), 127.7 (CH), 127.6 (CH), 127.5 (CH), 99.0 (C-1), 80.1 (CMe_3), 74.4 (C3/C4 + CH_2Ph), 73.6 (CH_2Ph), 72.8 (C3/C4), 70.5 (CH_2Ph), 70.4 (C5), 69.3 (C6), 68.7 (CH_2CHN), 54.1 (CHN), 52.52 ($COCH_3$), 31.1 (C-2), 28.4 (3 × CH_3). The spectra are consistent with those in the literature although our assignments differ slightly.[22]

2-Deoxy-3,4,6-tri-*O*-benzyl-α-D-lyxo-hexopyranosyl-(1→*O*)-*N*-*tert*-butoxycarbonyl-L-threoine methyl ester 7f

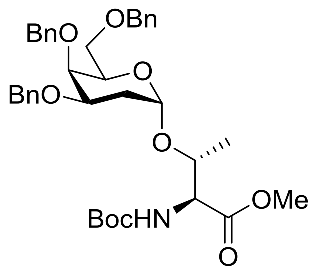

Following General Procedure 2, benzyl galactal **3a** (250 mg, 0.60 mmol) and *N*-(*tert*-butoxycarbonyl)-L-threonine methyl ester (117 mg, 0.50 mmol) and 2-thiouracil (0.36 mg, 0.005 mmol, 1 mol%) were used, and refluxed in 2-methyl THF for 18 h. The dried residue was purified via flash chromatography (cyclohexane:ethyl acetate 4:1, $R_f = 0.41$) affording **7f** as a white solid (295 mg, 91%).

MP 110–112 °C; ^{1}H NMR (400 MHz, $CDCl_3$) δ: 7.41–7.20 (m, 15H, ArH), 5.13 (d, J = 9.8 Hz, 1H, NH), 4.93–4.88 (m, 2H, H-1, C*H*HPh), 4.67–4.54 (m, 3H, 3 × CH*H*Ph), 4.49 (d, J =

11.8 Hz, 1H, CH*H*Ph), 4.41 (d, *J* = 11.8 Hz, 1H, CH*H*Ph), 4.33–4.23 (m, 2H, 2 × CH), 3.95–3.87 (m, 2H, H-4, H-5), 3.83 (ddd, *J* = 12.1, 4.6, 2.4 Hz, 1H, H-3), 3.72 (s, 3H, OCH_3), 3.60–3.50 (m, 2H, H-6a, H-6b), 2.14 (td, *J* = 12.4, 3.8 Hz, 1H, H-2a), 1.84 (dd, *J* = 12.7, 4.5 Hz, 1H, H-2b), 1.48 (s, 9H, 3 × CH_3), 1.24 (d, *J* = 6.3 Hz, CH-C*H₃*); ^{13}C NMR (101 MHz, $CDCl_3$) δ: 171.7 (C=O), 156.1 (C=O), 138.9 (4°C), 138.5 (4°C), 138.2 (4°C), 128.6 (CH), 128.5 (CH), 128.34 (CH), 128.29 (CH), 127.8 (CH), 127.7 (CH), 127.64 (CH), 127.56 (CH), 99.5 (C-1), 80.2 (CMe_3), 75.5 (*C*HMe), 74.42, 74.40 (C-3, CH_2), 73.6 (CH_2), 73.1 (C-4), 70.52, 70.49 (CH_2, C-5), 69.6 (C-6), 58.5 (*C*HNHBoc), 52.4 (OCH_3), 31.4 (C-2), 28.5 (3 × CH_3), 18.7 (CH*C*H₃); HRMS-ESI (*m/z*): $[M + Na]^+$ calc'd for $C_{37}H_{47}NO_9Na$, 672.3144; found 672.3149. Spectra are in consistent with literature although there are minor differences in assignments of signals.[23]

2-Deoxy-3,4,6-tri-*O*-benzyl-α-D-lyxo-hexopyranosyl-(1→*O*)-*N*-*tert*-butoxycarbonyl-L-tyrosine methyl ester 7g

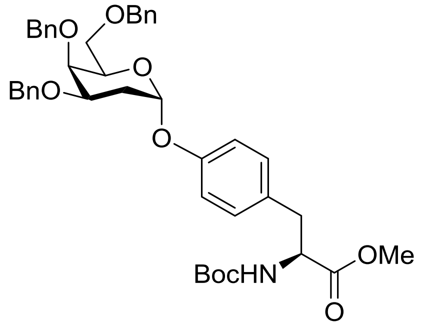

Following General Procedure 2, galactal **3a** (250 mg, 0.60 mmol), *N*-*tert*-butoxycarbonyl-L-tyrosine methyl ester (148 mg, 0.50 mmol), anhydrous CH_2Cl_2 (0.6 mL) and 2-thiouracil (0.6 mg, 0.5 µmol) were used. Following purification by column chromatography (cyclohexane:EtOAc 4:1), the product **7g** was obtained as a colourless oil (335 mg, 94%).

R_f = 0.35 (cyclohexane:EtOAc; 4:1); ^{1}H NMR (400 MHz; $CDCl_3$) δ 7.41 – 7.18 (m, 15H, ArH), 7.04 – 6.92 (m, 4H, ArH), 5.65 (d, *J* = 2.7 Hz, 1H, H-1), 4.96 (app d, *J* = 11.4 Hz, 2H, NH + PhC*H*H), 4.71–4.59 (m, 3H, 3 × PhC*H*H), 4.59–4.48 (m, 1H, CHN), 4.41 (d, *J* = 11.6 Hz 1H, PhC*H*H), 4.35 (d, *J* = 11.6 Hz, 1H, PhC*H*H), 4.11 (ddd, *J* = 12.0, 4.5, 2.4 Hz 1H, H-3/4/5), 4.06–3.98 (m, 2H, 2 of H-3/4/5), 3.67 (s, 3H, OCH_3), 3.66–3.62 (m, 1H, CH, H-6a), 3.51 (dd, *J* = 9.2, 5.5 Hz, 1H, H-6b), 3.03 (dd, *J* = 13.9, 5.7 Hz, 1H, C*H*HCHN), 2.97 (dd, *J* = 14.1, 6.2 Hz, 1H, CH*H*CHN), 2.39 (td, *J* = 12.4, 3.6 Hz, 1H, H-2a), 2.17 (dd, *J* = 12.4, 3.6 Hz, 1H, H-2b), 1.41 (br s, 9H, $(CH_3)_3$); ^{13}C NMR (101 MHz, $CDCl_3$) δ: 172.4 (C=O), 156.1 (4°C), 155.2 (4°C), 138.9 (4°C), 138.5 (4°C), 138.1 (4°C), 130.3 (CH), 129.4 (4°C), 128.5 (CH), 128.4 (CH), 128.3 (CH), 128.27 (CH), 127.8 (CH), 127.7 (CH), 127.67 (CH), 127.61 (CH), 127.4 (CH), 116.8 (CH), 96.8 (C-1), 79.9 (CMe_3), 74.6, 74.5 (CH + CH_2Ph), 73.4 (CH_2Ph), 72.9 (CH), 70.7, 70.6 (CH + CH_2Ph), 69.1 (C-6), 54.6 (CHN), 52.2 (OCH_3), 37.6 (*C*H₂CHN), 31.4 (C-2), 28.4 (3 × CH_3); $[\alpha]_D$ = +12 (*c* 0.18, $CHCl_3$); IR ν_{max}, neat/cm^{-1}: 3428 (br, N-H), 1743 (C=O), 1691 (C=O). HRMS-ESI (*m/z*): $[M + Na]^+$ calc'd for $C_{42}H_{49}NO_9Na$, 734.3300; found 734.3312.

Cholesteryl (3,4,6-tri-*O*-benzyl-2-deoxy-α-D-lyxo-hexapyranosyl) 7h

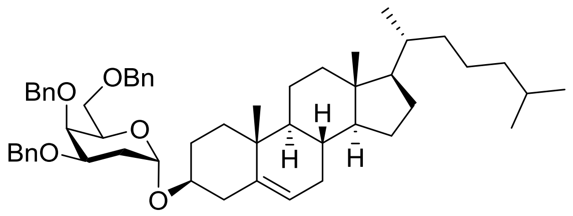

In a slight modification (due to lower solubility of cholesterol) of General Procedure 2, galactal **3a** (70 mg, 0.17 mmol), cholesterol (54 mg, 0.14 mmol), anhydrous CH_2Cl_2 (0.4 ml) and 2-thiouracil (0.2 mg, 1 μmol) were used. Following purification by column chromatography (cyclohexane:EtOAc; 6:1), the product **7h** was obtained as a white solid (110 mg, 98%).

R_f = 0.5 (cyclohexane:EtOAc; 6:1); ^{1}H NMR (500 MHz; $CDCl_3$): δ 7.37-7.22 (m, 15H, Ph), 5.26 (d, *J* = 5.4 Hz, 1H, C=CH), 5.15 (d, *J* = 3.4 Hz, 1H, H-1), 4.94 (d, *J* = 11.6 Hz, 1H, OC*H*HPh), 4.67-4.58 (m, 3H, 3 × OCH*H*Ph), 4.51 (d, *J* = 11.7 Hz, 1H, OC*H*HPh), 4.44 (d, *J* = 11.7 Hz, 1H, OCH*H*Ph), 4.01 (t, *J* = 6.4 Hz, 1H, H-5), 3.99-3.92 (m, 2H, H-3, H-4), 3.65-3.53 (app hept, *J* = 6.6 Hz, 2H, H-6a/b), 3.51-3.41 (m, 1H, CH), 2.32-2.20 (m, 3H, CH_2, H-2a), 2.06-1.91 (m, 3H, CH_2, C*H*H), 1.90-1.78 (m, 3H, CH_2, C*H*H), 1.61-1.05 (m, 17 H), 1.04-0.96 (m, 3H, CH, CH_2), 1.00 (s, 3H, CH_3), 0.95-0.90 (m, 1H, CH), 0.92 (d, *J* = 6.5 Hz, 3H, CH_3), 0.88 (d, *J* = 6.6 Hz, 3H, CH_3), 0.87 (d, *J* = 6.6 Hz, 3H, CH_3), 0.68 (s, 3H, CH_3); ^{13}C NMR (125 MHz; $CDCl_3$): δ 141.0 (*C*=CH), 139.1 (4° C), 138.8 (4° C), 138.3 (4° C), 128.51 (CH), 128.49 (CH), 128.32 (CH), 128.31 (CH), 127.9 (CH), 127.7 (CH), 127.60 (CH), 127.58 (CH), 127.4 (CH), 121.8 (C=*C*H), 95.8 (C-1), 76.3 (CH), 75.2 (C-3), 74.4 ($PhCH_2$), 73.6 ($PhCH_2$), 73.3 (C-4), 70.6 ($PhCH_2$), 70.0 (C-5), 69.8 (C-6), 56.9 (CH), 56.3 (CH), 50.3 (CH), 42.5 (4° C), 40.2 (CH_2), 39.9 (CH_2), 39.7 (CH_2), 37.3 (CH_2), 36.9 (4° C), 36.3 (CH_2), 35.9 (CH), 32.09 (CH_2), 32.04 (CH), 31.8 (CH_2), 28.3 (CH_2), 28.2 (CH), 28.0 (CH_2), 24.5 (CH_2), 24.0 (CH_2), 23.0 (CH_3), 22.7 (CH_3), 21.2 (CH_2), 19.5 (CH_3), 18.9 (CH_3), 12.0 (CH_3). HRMS (ESI) for $C_{54}H_{74}NaO_5^+$ (MNa^+) calculated: 825.5434; found: 825.5438. $[\alpha]_D^{20}$ = +35 (*c* = 0.010, $CHCl_3$). Spectra were consistent with literature data.[24]

Scheme 5

3,4,6-Tri-*O*-benzyl-2-deoxy-α/β-D-galactopyranosyl *p*-toluenesulfonamide 12

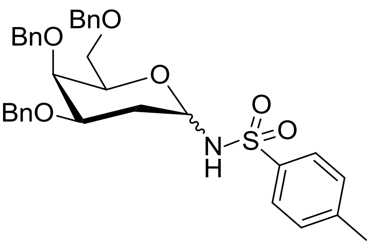

Following General Procedure 2, galactal **3a** (230 mg, 0.55 mmol), *p*-toluenesulfonamide (79 mg, 0.46 mmol), anhydrous CH_2Cl_2 (0.55 ml) and 2-thiouracil (0.9 mg, 7 μmol) were used. Following purification by column chromatography (cyclohexane:EtOAc; 5:1), the product

was obtained as a white solid (240 mg, 89%, 1.0:3.6 α/β mixture). R_f = 0.15 (cyclohexane:EtOAc; 5:1);

^{1}H NMR (500 MHz; $CDCl_3$):

The following were observed for both diastereomers: δ 7.37-7.19 (m, 15 H)

α anomer: δ 7.77 (d, *J* = 8.0 Hz, 2H, 2 × Ar*H*), 7.17 (d, *J* = 8.2 Hz, 2H, 2 × Ar*H*), 5.94-5.86 (m, 1H, N*H*), 5.39 (app t, *J* = 5.4 Hz, 1H, H-1), 4.82 (app d, *J* = 11.6 Hz, 1H, OC*H*HPh), 4.56-4.52 (m, 3H, OCH*H*Ph, OCH_2Ph), 4.24 (s, 2H, OCH_2Ph), 3.82 (br s, 1H, H-4), 3.71-3.66 (m, 1H, H-3), 3.53-3.49 (m, 1H, H-5), 3.45 (app d *J* = 8.7 Hz, 1H, H-6a), 2.82 (dd, *J* = 8.8, 5.0 Hz, 1H, H-6b), 2.34 (app td, *J* = 12.4, 5.2 Hz, 1H, H-2a), 2.33 (s, 3H, CH_3), 1.84 (br dd, *J* = 12.6, 4.6 Hz, 1H, H-2b).

β anomer: δ 7.72 (d, *J* = 8.2 Hz, 2H, 2 × Ar*H*), 7.08 (d, *J* = 8.1 Hz, 2H, 2 × Ar*H*), 5.38-5.32 (m, 1H, N*H*), 4.85 (d, *J* = 11.6 Hz, 1H, OCH*H*Ph), 4.77 (td, *J* = 10.7, 2.3 Hz, 1H, H-1), 4.58-4.54 (m, 3H, OC*H*HPh, OCH_2Ph), 4.33 (d, *J* = 12.0 Hz, 1H, OC*H*HPh), 4.30 (d, *J* = 11.9 Hz, 1H, OC*H*HPh), 3.78 (br s, 1H, H-4), 3.56 (ddd, *J* = 11.7, 4.4, 2.6 Hz, 1H, H-3), 3.41 (app t, *J* = 6.3 Hz, 1H, H-5), 3.34 (t, *J* = 9.0 Hz, 1H, H-6a), 3.15 (dd, *J* = 9.2, 5.4, Hz, 1H, H-6b), 2.29 (s, 3H, CH_3), 2.10-2.04 (m, 1H, H-2a), 1.96 (ddd, *J* = 11.7, 11.6, 11.6 Hz, 1H, H-2b).

^{13}C NMR (125 MHz; $CDCl_3$):

The following were observed for both diastereomers: δ 127.8 (CH), 127.74 (CH), 127.73 (CH), 127.72 (CH).

α anomer: δ 143.3 (4° C), 138.6 (4° C), 138.16 (4° C), 128.15 (4° C), 138.0 (4° C), 129.4 (CH), 128.4 (CH), 128.2 (CH), 128.0 (CH), 127.6 (CH), 127.5 (CH), 127.3 (CH), 79.3 (C-1, $^1J_{C1H1}$ = 168 Hz, from coupled HSQC), 74.2 (Ph*C*H_2), 73.8 (C-3), 73.4 (Ph*C*H_2), 72.4 (C-4), 70.5 (Ph*C*H_2), 70.2 (C-5), 68.0 (C-6), 30.7 (C-2), 21.5 (CH_3).

β anomer: δ 143.2 (4° C), 138.6 (4° C), 138.4 (4° C), 137.94 (4° C), 137.89 (4° C), 129.2 (CH), 128.5 (CH), 128.49 (CH), 128.44 (CH), 128.3 (CH), 127.42 (CH), 127.38 (CH), 81.1 (C-1, $^1J_{C1H1}$ = 155 Hz, from coupled HSQC), 77.5 (C-3), 75.3 (C-5), 74.5 (Ph*C*H_2), 73.3 (Ph*C*H_2), 71.4 (C-4), 70.5 (Ph*C*H_2), 68.5 (C-6), 32.9 (C-2), 21.4 (CH_3).

Spectra were consistent with literature data except our assignment of H4/5 &C3/4/5 for **12β** differs from that of Colinas and Bravo.[17,25]

2-Thiouracil-Catalysed Glycosylation: Catalyst Loading, Concentration and Gram-Scale Synthesis

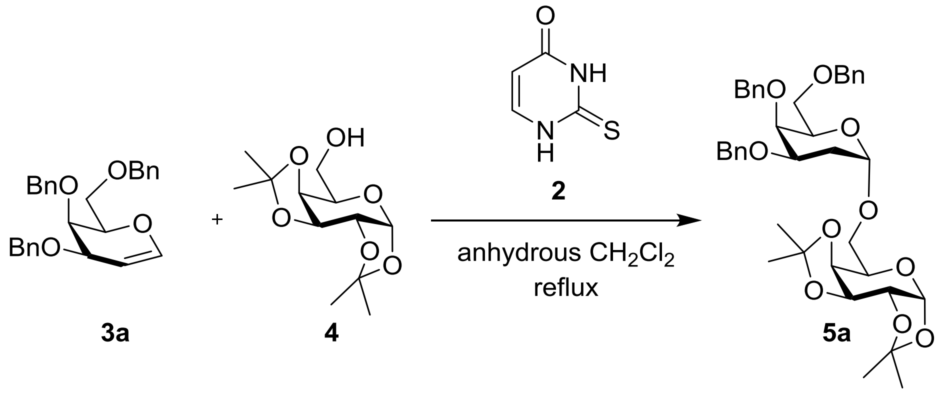

General Procedure 3: 2-Thiouracil-catalysed glycosylation in a sealed vessel

The galactal donor (1.2 eq. w.r.t. acceptor) was weighed into a crimp top vial (20 ml volume) equipped with a magnetic stir-bar, followed by 2-thiouracil. The crimp top vial was sealed (crimp seal with PTFE septum) and placed under vacuum before switching to a N_2 atmosphere. In a separate flask, under a N_2 atmosphere, an anhydrous solution of acceptor was made by adding a known quantity of acceptor and dissolving in anhydrous CH_2Cl_2. The stock solution was dried by addition of $MgSO_4$ (1/1 mol/mol w.r.t. acceptor) and let sit for 30 min. The stock solution of acceptor in CH_2Cl_2 was then decanted by syringe and added to the sealed vial. The reaction was heated at reflux (under N_2) for 18 h or until TLC/NMR analysis showed the reaction was complete. The solution was then concentrated under reduced pressure and purified by column chromatography.

Gram-scale synthesis

Following General Procedure 3, galactal **3a** (977 mg, 2.35 mmol), an anhydrous CH_2Cl_2 solution of galactose acceptor **4** (1.7M, 1.1 ml, 1.9 mmol), and 2-thiouracil (0.246 mg, 2 µmol, 0.1 mol%) were used. Purification by column chromatography afforded the product **5a** as a yellow oil (1.04 g, 78% yield). The proton and carbon NMR data were consistent with the data provided above.

Investigation of catalyst solubility

Using a volumetric flask, 2-thiouracil **2** (1.1 mg ± 0.3, 8.6 µmol) was dissolved in MeOH (10 ml; 0.86mM). Serial dilutions were made by removal of a known aliquot from the stock solution (using a pipette) and making up to a known volume using a volumetric flask. The following serial dilutions were obtained:

2.5 ml of stock solution made up to 5 ml MeOH; 0.43 mM

2.5 ml of stock solution made up to 10 ml MeOH; 0.21 mM

1.25 ml of stock solution made up to 10 ml MeOH; 0.1 mM

0.625 ml of stock solution made up to 10 ml MeOH; 0.05 mM

0.313 ml of stock solution made up to 10 ml MeOH; 0.026 mM

The solutions were analysed by reverse phase HPLC (observed at 254 nm). A calibration curve was constructed by plotting concentration of 2-thiouracil solution in MeOH (mM; x-axis) *vs.* area observed by HPLC (mAu; y-axis).

HPLC conditions: Phenomenex® Kinetex 5 µm polar 100 Å C18 column (250 mm length, 4.6 mm diameter; H_2O/Acetonitrile (95:5); 1 ml/min; 10 min run.

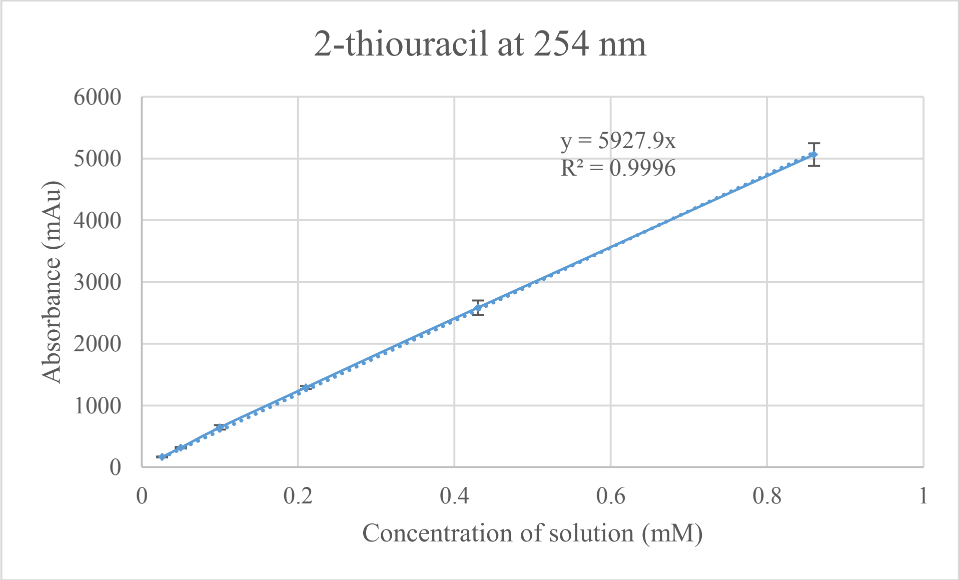

Figure S1: Calibration curve: concentration of 2-thiouracil solution in MeOH (mM; x-axis) *vs.* area observed by HPLC (mAu; y-axis)

Determination of concentration of 2-thiouracil (2) in CH_2Cl_2:

2-Thiouracil **2** (150 mg, 1.17 mmol) was weighed into a Young's flask (under a N_2 atmosphere) and anhydrous CH_2Cl_2 (10 ml) was added. The vessel was sealed and the suspension was heated at 50 °C for 1 h. The solution was filtered while hot using syringe filter (PTFE; 0.4 µm pore size) into a separate Young's flask (under N_2). Using a pipette, 1 ml of this solution was removed and made up to 2 ml using MeOH in a volumetric flask. The sample was analysed by HPLC three times and by relation to the calibration curve above the concentration was determined ($x=(y/m)\times 2$).

Area of 2-thiouracil trace in HPLC chromatogram (observed at 254 nm):

Run 1 = 93.7832

Run 2 = 93.8652

Run 3 = 93.5310

Average area = 93.7265

$x = (93.7265/5927.9) \times 2 = 0.032$ mM

Investigation of catalyst loading

2-Thiouracil: 0.1 mol% loading

In a slight modification to General Procedure 3, 2-thiouracil (from catalyst stock solution; concentration determined by HPLC as 0.032 mM) was added to the crimp top vial (13 ml, 0.42 µmol, 0.1 mol%). The anhydrous CH_2Cl_2 (from catalyst stock solution) was removed under vacuum. Galactal **3a** (210 mg, 0.5 mmol), an anhydrous CH_2Cl_2 solution of galactose acceptor **4** (2.1M, 0.2 ml, 0.42 mmol) were used. Analysis of the ^{1}H NMR spectrum of the crude mixture after 18 h showed that the reaction was complete.

2-Thiouracil: 0.01 mol% loading

In a slight modification to General Procedure 3, 2-thiouracil (from stock solution; concentration determined by HPLC as 0.032 mM) was added to the crimp top vial (1.3 ml, 0.042 µmol, 0.01 mol%). The anhydrous CH_2Cl_2 (from stock solution) was removed under vacuum. Galactal **3a** (210 mg, 0.5 mmol), an anhydrous CH_2Cl_2 solution of galactose acceptor **4** (2.1M, 0.2 ml, 0.42 mmol) were used. Analysis of the ^{1}H NMR spectrum of the crude mixture after 18 h showed that the reaction had proceeded but was not complete (~80% conversion of acceptor). However, when the experiment was repeated and the level of conversion (80% in 18 h) was not reproduced. The level of conversion in this reaction varied from experiment to experiment (81% conversion w.r.t. acceptor **4** after 18 h *vs.* 14% conversion w.r.t. acceptor **4** after 19 h).

2-Thiouracil: 0.001 mol% loading

In a slight modification to General Procedure 3, 2-thiouracil (from stock solution; concentration determined by HPLC as 0.034 mM) was added to the crimp top vial (124 µl, 0.0042 µmol, 0.001 mol%). The anhydrous CH_2Cl_2 (from stock solution) was removed under vacuum. Galactal **3a** (203 mg, 0.49 mmol), an anhydrous CH_2Cl_2 solution of galactose acceptor **4** (1.9M, 0.25 ml, 0.46 mmol) were used. Analysis of the ^{1}H NMR spectrum of the crude mixture after 18 h showed only the starting materials.

1,1’-Linked Disaccharides

2,3,4,6-Tetra-*O*-benzyl-α-*O*-D-glucopyranoside 10

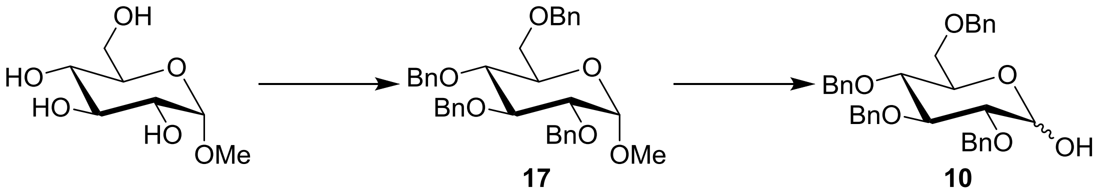

Following a modified literature procedure,[26] α-D-methyl glucopyranoside (20.32 g, 0.105 mol) was charged to a dry flask under N_2. Anhydrous DMF (250 ml) was added and the solution stirred for 30 min. The solution was cooled with an ice bath and sodium hydride (60% in oil, 25.2 g, 0.63 mol) was added in 3 portions. The solution was stirred for 1 h until

all effervescence had stopped, after which benzyl bromide (74.8 ml, 0.63 mol) was added dropwise. The flask was heated to 30 °C and left to stir overnight. When TLC (cyclohexane:EtOAc; 4:1) showed no product remained, methanol (100 ml) was slowly added to the flask and the mixture was left to stir for 30 min. Then the methanol was removed on a rotary evaporator before DMF was removed by high vacuum. The crude yellow oil was extracted with CH_2Cl_2 (400 ml) and washed with water (250 ml × 2), then brine (250 ml), dried over $MgSO_4$ and concentrated. The resulting yellow oil was purified by column chromatography (cyclohexane:EtOAc; 4:1) to give the product **17** as a clear yellow oil (42.33 g, 74%).

^{1}H NMR (500 MHz; $CDCl_3$): δ 7.43-7.24 (m, 18H, Ph), 7.17-7.12 (m, 2H, Ph), 4.99 (d, J = 10.9 Hz, 1H), 4.85-4.79 (m, 3H), 4.70-4.59 (m, 1H), 4.64 (d, J = 3.6 Hz, 1H, H-1), 4.51-4.46 (m, 2H), 4.00 (t, J = 9.3 Hz, 1H, H-3), 3.79-3.71 (m, 2H), 3.67-3.62 (m, 2H), 3.57 (dd, J = 9.7, 3.5 Hz, 1H, H-2), 3.39 (s, 3H, OCH_3). Spectra were consistent with literature data.[26]

The oil **17** was dissolved in glacial acetic acid (393 ml) and solution then heated to 90 °C. After 2 h, sulfuric acid (2M, 98 ml) was slowly added and the reaction stirred overnight at 90 °C. When TLC (cyclohexane:EtOAc; 3.5:1), showed no starting material remained (R_f = 0.4), water (400 ml) was added and the solution cooled to 0 °C. A white solid precipitated. This was removed by filtration and washed with a solution of methanol (MeOH:H_2O; 3:1; v/v). Recrystallization from cyclohexane gave **10** as white solid (17.79 g, 45%) as an α/β mixture (1/0.40).

M.p. 141-143 °C (cyclohexane) [Lit.[27] 152-154 °C (cyclohexane)].

Setting α anomer to 1H, α:β = 1:0.40. ^{1}H NMR (400 MHz; $CDCl_3$): δ 7.37-7.22 (m, 25.2H, Ph), 7.17-7.12 (m, 2.8H, Ph), 5.23 (t, J = 2.7 Hz, 1H, H-1α), 4.97-4.90 (m, 1.4H, C*H*Hα + C*H*Hβ), 4.86-4.66 (m, 6H, C*H*Hα × 2 + CH_2α + C*H*Hβ × 2 + CH_2β +H-1β), 4.62-4.46 (m, 4.2H, C*H*Hα + CH_2α + C*H*Hβ + CH_2β), 4.03 (ddd, J = 10.0, 3.3, 2.1 Hz, 1H, C*H*α), 3.96 (t, J = 9.3 Hz, 1H, C*H*α), 3.74-3.50 (m, 6H, CH_2α + C*H*α × 2 + CH_2β + C*H*β × 3), 3.39 (dd, J = 9.0, 7.7 Hz, 0.4H, C*H*β), 3.14 (d, J = 5.5 Hz, 0.4H, OHβ), 2.88 (d, J = 1.7Hz, 1H, OHα); ^{13}C NMR (400 MHz; $CDCl_3$): δ 138.8 (4° C), 138.6 (4° C), 138.5 (4° C), 138.3 (4° C), 138.1 (4° C), 138.0 (4° C), 137.93 (4° C), 137.86 (4° C), 128.6 (CH), 128.51 (CH), 128.50 (CH), 128.48 (CH), 128.46 (CH), 128.24 (CH), 128.15 (CH), 128.1 (CH), 128.0 (CH), 128.97 (CH), 127.96 (CH), 127.9 (CH), 127.84 (CH), 127.81 (CH), 127.8 (CH), 127.7 (CH), 97.6 (C-1β), 91.4 (C-1α), 84.7 (CHβ), 83.2 (CHβ), 81.9 (CHα), 80.1 (CHα), 77.94 (CHβ), 77.85 (CHα), 75.82 (CH_2α), 75.77 (CH_2β), 75.11 (CH_2α), 75.09 (CH_2β), 74.82 (CH_2β or CHβ), 74.75 (CH_2β or CHβ), 73.60 (CH_2β), 73.57 (CH_2α), 73.3 (CH_2α), 70.3 (CHα), 69.0 (CH_2β), 68.7 (CH_2α). Spectra were consistent with literature.[26,28]

Scheme 2

Synthesis of 3,4,6-tri-*O*-benzyl-α-D-lyxo-hexapyranosyl-(1→1')-3',4',6'-tri-*O*-benzyl-α-D-lyxo-hexapyranoside α,α-9a and 3,4,6-tri-*O*-benzyl-α-D-lyxo-hexapyranosyl-(1→1')-3',4',6'-tri-*O*-benzyl-β-D-lyxo-hexapyranoside α,β-9a

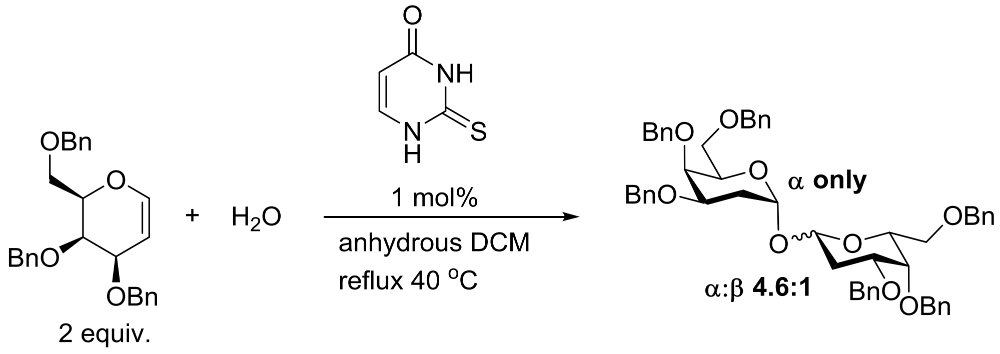

A 5 ml RBF equipped with a reflux condenser, stir-bar and gas-inlet was set-up under a N_2 atmosphere. Galactal **3a** (202 mg, 0.49 mmol) was weighed into the reaction flask and dried under vacuum for 30 min. The flask was then switched to a N_2 atmosphere and **3a** was dissolved in anhydrous CH_2Cl_2 (0.9M w.r.t H_2O, 0.3 ml). 2-Thiouracil **2** (0.30 mg, 2.4 μmol) was added to the reaction flask followed by deionised H_2O (5.0 μl, 0.27 mmol). The mixture was heated to reflux for 18 h. Some solvent loss was noted over the course of the reaction and the higher concentration that results is believed to be beneficial. Analysis of the ^{1}H NMR spectrum of the crude mixture showed the α,α/α,β dimer ratio to be 4.6:1. Purification by column chromatography (9:1; cyclohexane/ethyl acetate) gave the desired products as colourless oils; α,α-**9a** (118 mg, 57% yield); mixed fractions containing α,α/α,β-**9a** (47 mg, 23% yield); α,β-**9a** (19 mg, 9% yield).

We note that **9a** underwent anomerisation under extended reaction times with the amount of α,α increasing relative to α,ß over time. Thus after 112 h at reflux the α,α:α,ß ratio was determined to be 13:1 by ^{1}H NMR spectroscopy. Some degradation was also noted. A similar time-dependent anomerisation was previously observed by Yoshimura and co-workers in their synthesis of α,α-trehalose.[29]

3,4,6-Tri-*O*-benzyl-α-D-*lyxo*-hexapyranosyl-(1→1')-3',4',6'-tri-*O*-benzyl-α-D-*lyxo*-hexapyranoside (α,α-9a): R_f = 0.33 (85:15; cyclohexane/ethyl acetate); ^{1}H NMR (400 MHz, Chloroform-*d*) δ 7.43 – 7.16 (m, 15H, Ph), 5.24 (d, *J* = 3.3 Hz, 1H, H-1), 4.93 (d, *J* = 11.7 Hz, 1H, OC*H*HPh), 4.61 (d, *J* = 11.7 Hz, 1H, OC*H*HPh), 4.59 (d, *J* = 11.9 Hz, 1H, OC*H*HPh), 4.55 (d, J = 11.8 Hz, 1H, OC*H*HPh), 4.48 (d, *J* = 11.7 Hz, 1H, OC*H*HPh), 4.40 (d, *J* = 11.7 Hz, 1H, OC*H*HPh), 3.93 (br s, 1H, H-4), 3.88 – 3.79 (m, 2H, H-3, H-5), 3.62 (dd, *J* = 9.2, 7.3 Hz, 1H, H-6a), 3.53 (dd, *J* = 9.2, 5.7 Hz, 1H, H-6b), 2.24 (td, *J* = 12.4, 3.7 Hz, 1H, H-2a), 1.85 (dd, *J* = 12.6, 4.5 Hz, 1H, H-2b);

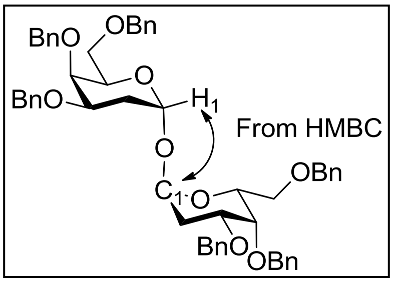

^{13}C NMR (101 MHz, Chloroform-*d*) δ 139.0 (4° C), 138.6 (4° C), 138.2 (4° C), 128.6 (CH), 128.5 (CH), 128.4 (CH), 128.3 (CH), 128.0 (CH), 127.9 (CH), 127.7 (CH), 127.6 (CH),

127.5 (CH), 93.5 (C-1), 74.5, 74.4 (C-3 or C-5, Ph*C*H$_2$), 73.7 (Ph*C*H$_2$), 73.1 (C-4), 70.6, 70.5 (C-3 or C-5, Ph*C*H$_2$), 69.5 (C-6), 31.0 (C-2); ESI-HRMS for $C_{54}H_{58}NaO_9^+$ (MNa$^+$) calculated: 873.3979; found: 873.4015.

3,4,6-Tri-*O*-benzyl-α-D-*lyxo*-hexapyranosyl-(1→1')-3',4',6'-tri-*O*-benzyl-β-D-*lyxo*-hexapyranoside (α,β-9a): R_f = 0.25 (85:15; cyclohexane/ethyl acetate); ^{1}H NMR (400 MHz, Chloroform-*d*) δ 7.52 – 6.99 (m, 30H, Ph), 5.19 (d, *J* = 3.4 Hz, 1H, H-1(α)), 4.90 (d, *J* = 11.6 Hz, 1H, OC*H*HPh), 4.89 (d, *J* = 11.6 Hz, 1H, OC*H*HPh), 4.64 (d, *J* = 11.7 Hz, 1H, OC*H*HPh), 4.61 – 4.57 (m, 2H, OC*H*HPh, H-1' (β) [HSQC shows a crosspeak for ^{13}C 99.6 and ^{1}H 4.59 (d, $J_{H1,H2}$ *ca.* 10 Hz)]), 4.57 – 4.52 (m, 4H, OC*H*HPh), 4.39 (d, *J* = 12.0 Hz, 1H, OC*H*HPh), 4.33 (d, *J* = 10.6 Hz, 3H, 3 × OC*H*HPh), 4.25 (app t, *J* = 6.7 Hz, 1H, H-5 (α)), 3.98 (ddd, *J* = 12.0, 4.6, 2.4 Hz, 1H, H-3 (α)), 3.93 (br, s, 1H, H-4 (α)), 3.84 (br s, 1H, H-4' (β)), 3.67 – 3.54 (m, 2H, H-6a/a'), 3.54 – 3.44 (m, 4H, H-6b/b', H-3' (β), H-5' (β)), 2.19 (td, *J* = 12.4, 3.5 Hz, 1H, H-2a (α)), 2.15 – 2.07 (m, 1H, H-2a'(β)), 2.06 – 1.93 (m, 2H, H-2b (α), H-2b'(β)); ^{13}C NMR (101 MHz, Chloroform-*d*) δ 139.2 (4° C), 139.1 (4° C), 138.7 (4° C), 138.5 (4° C), 138.4 (4° C), 138.1 (4° C), 128.6 (CH), 128.52 (CH), 128.51 (CH), 128.48 (CH), 128.4 (CH), 128.30 (CH), 128.28 (CH), 127.93 (CH), 127.92 (CH), 127.83 (CH), 127.76 (CH), 127.67 (CH), 127.66 (CH), 127.63 (CH), 127.62 (CH), 127.52 (CH), 127.45 (CH), 127.43 (CH), 99.6 (C-1' (β)), 98.2 (C-1 (α)), 77.5 (C-3' (β)), 74.7 (C-3 (α)), 74.5 (Ph*C*H$_2$), 74.4 (Ph*C*H$_2$), 74.1 (C-5 (β)), 73.5 (Ph*C*H$_2$), 73.3 (Ph*C*H$_2$), 73.2 (C-4 (α)), 71.5 (C-4' (β)), 70.6 (Ph*C*H$_2$), 70.4 (Ph*C*H$_2$), 70.2 (C-5 (α)), 69.3 (C-6 (α)), 68.8 (C-6' (β)), 33.2 (C-2' (β)), 31.3 (C-2 (α)); ESI-HRMS for $C_{54}H_{58}NaO_9^+$ (MNa$^+$) calculated: 873.3979; found: 873.3978.

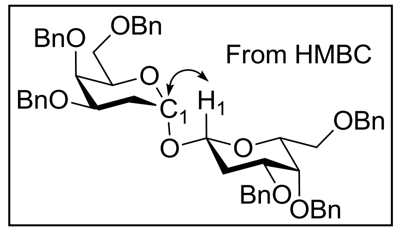

Synthesis of 2,3,4,6-tetra-*O*-benzyl-α-D-glucopyranosyl-(1→1')-3',4',6'-tri-*O*-benzyl-α-D-lyxo-hexapyranoside α,α-11a and of 2,3,4,6-tetra-*O*-benzyl-β-D-glucopyranosyl-(1→1')-3',4',6'-tri-*O*-benzyl-α-D-lyxo-hexapyranoside α,β-11a

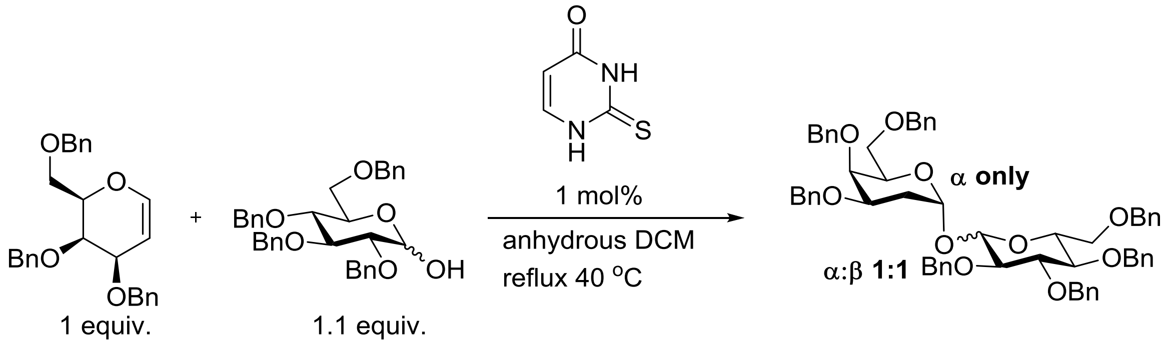

In a slight modification to General Procedure 1, galactal **3a** (201 mg, 0.48 mmol), an anhydrous CH_2Cl_2 acceptor solution of 2,3,4,6-tetra-*O*-benzyl-D-glucopyranose **10** (0.36M, 1.5 ml, 0.54 mmol) and 2-thiouracil (0.60 mg, 5 μmol, 1 mol%) were used. The mixture was heated to reflux for 18 h. Analysis of the ^{1}H NMR spectrum of the crude mixture showed the α,α/α,β product ratio to be 1:1. Purification by column chromatography (9:1; cyclohexane/ethyl acetate) gave the desired products as colourless oils; α,α-**11a** (179 mg,

39% yield); mixed fractions containing α,α/α,β-**11a** (118 mg, 26% yield), α,β-**11a** (90 mg, 20% yield).

2,3,4,6-Tetra-*O*-benzyl-α-D-glucopyranosyl-(1→1')-3',4',6'-tri-*O*-benzyl-α-D-*lyxo*-hexapyranoside (α,α-11a): R_f = 0.51 (85:15; cyclohexane/ethyl acetate); ^{1}H NMR (400 MHz, Chloroform-*d*) δ 7.41 – 7.08 (m, 35H, Ph), 5.30 (app d, *J* = 3.4 Hz, 2H, H-1 (α), H-1'(α)), 4.94 (d, *J* = 10.9 Hz, 1H, OC*H*HPh), 4.92 (d, *J* = 11.5 Hz, 1H, OC*H*HPh), 4.83 (d, *J* = 10.7 Hz, 1H, OC*H*HPh), 4.80 (d, *J* = 11.0 Hz, 1H, OC*H*HPh), 4.72 (d, *J* = 12.0 Hz, 1H, OC*H*HPh), 4.65 – 4.57 (m, 4H, 4 × OC*H*HPh), 4.55 (d, *J* = 12.0 Hz, 1H, OC*H*HPh), 4.48 (d, *J* = 10.6 Hz, 1H, OC*H*HPh) 4.47 (d, *J* = 12.1 Hz, 1H, OC*H*HPh), 4.43 (d, *J* = 11.8 Hz, 1H, OC*H*HPh), 4.35 (d, *J* = 11.8 Hz, 1H, OC*H*HPh), 4.21 (app t, *J* = 6.4 Hz, 1H, H-5'), 4.03 (ddd, *J* = 12.1, 4.5, 2.2 Hz, 1H, H-3'), 3.94 (t, *J* = 9.3 Hz, 1H, H-3), 3.92 (br s, 1H, H-4'), 3.82 – 3.61 (m, 4H, H-5, H-4, H-6a/b), 3.57 (dd, *J* = 9.7, 3.6 Hz, 1H, H-2), 3.55 – 3.50 (m, 2H, H-6a'/b'), 2.29 (td, *J* = 12.4, 3.7 Hz, 1H, H-2a'), 1.91 (dd, *J* = 12.7, 4.5 Hz, 1H, H-2b'); ^{13}C NMR (101 MHz, Chloroform-*d*) δ 139.0 (4° C), 138.99 (4° C), 138.5 (4° C), 138.29 (4° C), 138.27 (4° C), 138.25 (4° C), 138.1 (4° C), 128.6 (CH), 128.54 (CH), 128.51 (CH), 128.50 (CH), 128.4 (CH), 128.3 (CH), 128.2 (CH), 128.0 (CH), 127.93 (CH), 127.92 (CH), 127.88 (CH), 127.82 (CH), 127.7 (CH), 127.69 (CH), 127.68 (CH), 127.65 (CH), 127.61 (CH), 93.8 (C-1', $^1J_{C1,H1}$ = 170 Hz (α), from coupled HSQC), 92.3 (C-1, $^1J_{C1,H1}$ = 170 Hz (α), from coupled HSQC), 81.8 (C-3), 79.4 (C-2), 77.8 (C-4), 75.6 (Ph*C*H$_2$), 75.4 (Ph*C*H$_2$), 74.5 (2 × C, C-3', Ph*C*H$_2$), 73.64 (2 × C, Ph*C*H$_2$), 73.2 (C-4'), 72.6 (Ph*C*H$_2$), 71.0 (C-5), 70.7 (Ph*C*H$_2$), 70.6 (C-5'), 69.8 (C-6'), 68.5 (C-6), 31.1 (C-2'); ESI-HRMS for $C_{61}H_{64}NaO_{10}^+$ (MNa^+) calculated: 979.4397; found: 979.4361; $[\alpha]_D$ = +73 (*c*, 1.0, CH_2Cl_2).

2,3,4,6-Tetra-*O*-benzyl-β-D-glucopyranosyl-(1→1')-3',4',6'-tri-*O*-benzyl-α-D-*lyxo*-hexapyranoside (α,β-11a): R_f = 0.41 (85:15; cyclohexane/ethyl acetate); ^{1}H NMR (400 MHz, Chloroform-*d*) δ 7.53 – 7.05 (m, 35H, Ph), 5.25 (d, *J* = 3.3 Hz, 1H, H-1' (α)), 4.92 (d, *J* = 11.6 Hz, 1H, OC*H*HPh), 4.88 (d, *J* = 11.1 Hz, 1H, OC*H*HPh), 4.79 (d, *J* = 11.1 Hz, 1H, OC*H*HPh), 4.79 (d, *J* = 11.3 Hz, 1H, OC*H*HPh), 4.78 (d, *J* = 10.8 Hz, 1H, OC*H*HPh) 4.73 (d, *J* = 11.1 Hz, 1H, OC*H*HPh), 4.61 (d, *J* = 11.6 Hz, 1H, OC*H*HPh), 4.61 (d, *J* = 12.0 Hz, 1H, OC*H*HPh), 4.57 (d, *J* = 10.6 Hz, 1H, OC*H*HPh) 4.56-4.49 (m, 3H, 2 × OC*H*HPh, H-1 (β)), 4.42 (d, *J* = 11.8 Hz, 1H, OC*H*HPh),), 4.38 (d, *J* = 12.5 Hz, 1H, OC*H*HPh), 4.35 (d, *J* = 12.0 Hz, 1H, OC*H*HPh), 4.28 (t, *J* = 6.7 Hz, 1H, H-4' or H-5'), 4.04 – 3.94 (m, 2H, H-3', H-4' or H-5'), 3.72 – 3.49 (m, 6H, H-6a/b, H-6a'/b', H-3, and H-4 or H-5), 3.42 (t, *J* = 8.5 Hz, 1H, H-2), 3.40 – 3.34 (m, 1H, H-4 or H-5), 2.24 (td, *J* = 12.3, 3.7 Hz, 1H, H-2a'), 2.01 – 1.87 (m, 1H, H-2b'); ^{13}C NMR (101 MHz, Chloroform-*d*) δ 139.1 (4° C), 138.7 (4° C), 138.6 (4° C), 138.5 (4° C), 138.4 (4° C), 128.6 (CH), 128.53 (CH), 128.51 (CH), 128.47 (CH), 128.44 (CH), 128.43 (CH), 128.3 (CH), 128.2 (CH), 128.1 (CH), 128.0 (CH), 127.99 (CH), 127.91 (CH), 127.89 (CH), 127.88 (CH), 127.79 (CH), 127.74 (CH), 127.71 (CH), 127.68 (CH), 127.66 (CH), 127.5 (CH), 127.4 (CH), 102.3 (C-1, $^1J_{C1,H1}$ = 160 Hz (β), from coupled HSQC), 100.1 (C-1', $^1J_{C1,H1}$ = 170 Hz (α), from coupled HSQC), 85.04 (C-3), 82.50 (C-2), 77.7 (C-4 or C-5), 75.8 (Ph*C*H$_2$), 75.2 (C-4 or C-5), 75.2 (Ph*C*H$_2$), 75.0 (Ph*C*H$_2$), 74.53 (C-3'), 74.48 (Ph*C*H$_2$), 73.7 (Ph*C*H$_2$), 73.5 (Ph*C*H$_2$), 73.0 (C-4' or C-5'), 70.6 (C-4' or C-5'), 70.57 (Ph*C*H$_2$), 69.3 (C-6 or C-6'), 68.8 (C-6 or C-6'), 31.4 (C-2'); ESI-HRMS for $C_{61}H_{64}NaO_{10}^+$ (MNa^+) calculated: 979.4397; found: 979.4361; $[\alpha]_D$ = +39 (*c*, 1.0, CH_2Cl_2).

Procedures for Benzyl group removal

General Procedure for removal of benzyl protecting groups by hydrogenation:

The protected 2-deoxyglycoside was weighed into a round bottom flask and dissolved in a mixture of methanol/ethyl acetate (9:1). Pd (10% on carbon) (10 mol% for each benzyl group to be removed) was then added to the solution. The atmosphere was changed to hydrogen first by placing the reaction solution under house vacuum (200 mbar), closing the reaction flask to house vacuum and then purging the flask with hydrogen (using a hydrogen balloon). The cycle was repeated three times and on the final purge with hydrogen the hydrogen balloon was left in place. The reaction was monitored using TLC. The reaction mixture was filtered using Celite®. The solution was concentrated using rotary evaporation and purified using column chromatography (8:2; dichloromethane/methanol; 2% H_2O). For further purification the product isolated from column chromatography was passed through a plug of Octadecyl-C18-Silica (8:2; methanol/H_2O).

2,2'-Dideoxy-*lyxo*-trehalose (9b)

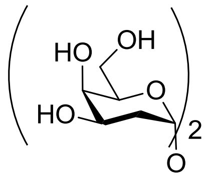

Following the general procedure for removal of benzyl protecting groups, α,α-**9a** (100 mg, 0.12 mmol), Pd (10% on carbon) (96 mg, 0.09 mmol, ~60 mol%) and methanol/ethyl acetate (9:1; 7 ml) were used. 2,2'-Dideoxy-*lyxo*-trehalose **9b** was obtained as a white solid (28 mg, 76% yield).

^{1}H NMR (400 MHz, methanol-d_4) δ 5.29 (d, J = 3.3 Hz, 1H, H-1 (α)), 3.98 (ddd, J = 12.0, 5.0, 2.9 Hz, 1H, H-3), 3.83 – 3.72 (m, 3H, H-4, H-5, H-6a), 3.69 (dd, J = 10.5, 4.6 Hz, 1H, H-6b), 2.02 (td, J = 12.6, 3.8 Hz, 1H, H-2a), 1.77 (dd, J = 12.9, 5.1 Hz, 1H, H-2b); ^{13}C NMR (101 MHz, methanol-d_4) δ 93.8 (C-1), 73.1 (C-5), 69.7 (C-4), 66.6 (C-3), 63.3 (C-6), 33.2 (C-2); ESI-HRMS for $C_{12}H_{22}NaO_9^+$ (MNa^+) calculated: 333.1162; found: 333.1147.

α-D-Glucopyranosyl-(1→1')-α-D-*lyxo*-hexapyranoside (α,α-11b)

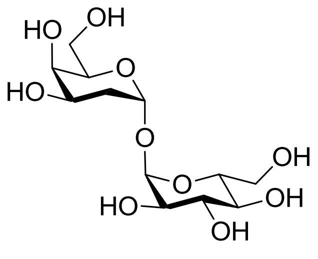

Following the general procedure for removal of benzyl protecting groups, α,α-**11a** (142 mg, 0.15 mmol), Pd (10% on carbon) (119 mg, 0.11 mmol, ~60 mol%) and methanol/ethyl acetate (9:1; 7 ml) were used. Disaccharide α,α-**11b** was obtained as a colourless oil (26 mg, 53% yield).

^1H NMR (500 MHz, methanol-d_4) δ 5.31 (d, *J* = 3.4 Hz, 1H, H-1'(α)), 5.15 (d, *J* = 3.8 Hz, 1H, H-1(α)), 4.14 (ddd, *J* = 12.0, 5.0, 3.0 Hz, 1H, H-3'), 4.00 (t, *J* = 6.1 Hz, 1H, H-5'), 3.83 (app dd, *J* = 11.9, 2.4 Hz, 2H, H-4', H-6a), 3.76 (dd, *J* = 11.4, 7.0 Hz, 1H, H-6a'), 3.73 – 3.65 (m, 3H, H-3, H-6b', H-6b), 3.60 (ddd, *J* = 10.0, 5.8, 2.3 Hz, 1H, H-5), 3.49 (dd, *J* = 9.8, 3.8 Hz, 1H, H-2), 3.35 – 3.30 (m, 1H, H-4), 2.04 (td, *J* = 12.6, 3.8 Hz, 1H, H-2a'), 1.81 (dd, *J* = 12.9, 5.1 Hz, 1H, H-2b'); ^{13}C NMR (126 MHz, methanol-d_4) δ 94.7 (C-1), 93.8 (C-1'), 74.8 (C-3), 74.2 (C-5), 73.1 (C-2), 72.6 (C-5'), 72.0 (C-4), 69.8 (C-4'), 66.3 (C-3'), 63.3 (C-6'), 62.7 (C-6), 33.2 (C-2'); ESI-HRMS for $C_{12}H_{22}NaO_{10}^+$ (MNa$^+$) calculated: 349.1111; found: 349.1104.

β-D-Glucopyranosyl-(1→1')-α-D-*lyxo*-hexapyranoside (α,β-11b)

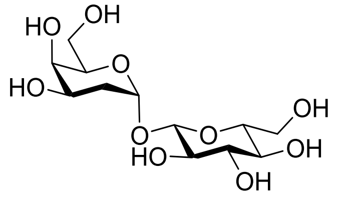

Following the general procedure for removal of benzyl protecting groups, α,β-**11a** (63 mg, 0.066 mmol), Pd (10% on carbon) (52 mg, 0.048 mmol, ~60 mol%) and methanol/ethyl acetate (9:1; 7 ml) were used. Disaccharide α,β-**11b** was obtained as a colourless oil (20 mg, 91% yield).

^1H NMR (500 MHz, methanol-d_4) δ 5.25 (t, *J* = 2.4 Hz, 1H, H-1' (α)), 4.49 (d, *J* = 7.9 Hz, 1H, H-1 (β)), 4.19 (dd, *J* = 7.7, 4.2 Hz, 1H, H-5'), 3.99 (ddd, *J* = 9.8, 6.9, 3.0 Hz, 1H, H-3'), 3.89 (dd, *J* = 11.9, 2.2 Hz, 1H, H-6a), 3.78 (dd, *J* = 11.4, 7.7 Hz, 1H, H-6a'), 3.77 (br s, 1H, H-4') 3.69 (dd, *J* = 11.4, 4.2 Hz, 1H, H-6b'), 3.63 (dd, *J* = 11.8, 6.8 Hz, 1H, H-6b), 3.42 – 3.34 (m, 2H, H-5, H-3 or H-4), 3.27 – 3.20 (m, 2H, H-2 and H-3 or H-4), 2.00 – 1.92 (m, 2H, H-2a'/b'); ^{13}C NMR (126 MHz, methanol-d_4) δ 103.5 (C-1), 100.7 (C-1'), 78.3, 78.1, 75.1, 73.1 (C-5'), 71.7, 69.9 (C-4'), 66.5 (C-3'), 63.8 (C-6'), 63.1 (C-6), 33.2 (C-2'); ESI-HRMS for $C_{12}H_{22}NaO_{10}^+$ (MNa$^+$) calculated: 349.1111; found: 349.1102.

Mechanistic Studies

Glycosylation reactions in the absence of the catalyst

Control reaction in reflux condenser

In a slight modification to General Procedure 1, galactal donor **3a** (251 mg, 0.6 mmol) and an anhydrous CH_2Cl_2 solution of galactose acceptor **4** (0.8M 0.6 ml, 0.5 mmol) were used. Analysis of the ^1H NMR spectrum of the crude mixture after 20 h showed only the starting materials. An analogous reaction with glucal donor **3f** also showed no reaction in the absence of catalyst.

Control reaction with uracil instead of thiouracil as catalyst

In a slight modification to General Procedure 1, galactal donor **3a** (200 mg, 0.48 mmol) and an anhydrous CH_2Cl_2 solution of galactose acceptor **4** (0.83M 0.48 ml, 0.40 mmol) and uracil (0.4 mg, 4 µmol) were used. Analysis of the ^1H NMR spectrum of the crude mixture after 19 h did not detect any disaccharide product.

Control reaction in sealed vessel

Following General Procedure 3, galactal donor **3a** (201 mg, 0.48 mmol) and an anhydrous CH_2Cl_2 solution of galactose acceptor **4** (1.8M (w.r.t. acceptor and solvent total volume), 0.25 ml, 0.5 mmol) were used. Analysis of the ^1H NMR spectrum of the crude mixture after 18 h showed only the starting materials.

No reaction of Galactal with Water in the absence of catalyst

In a slight modification to the General Procedure 1, galactal donor **3a** (255 mg, 0.6 mmol), deionised H_2O (9 µl, 0.5 mmol), and anhydrous CH_2Cl_2 (0.8M) were used. Analysis of the ^1H NMR spectrum of the crude reaction mixture after 16 h showed only the starting materials.

No reaction of galactal and *p*-toluenesulfonamide in the absence of catalyst:

In a slight modification to General Procedure 1, galactal **3a** (200 mg, 0.48 mmol), *p*-toluenesulfonamide (68 mg, 0.40 mmol) and anhydrous CH_2Cl_2 (0.48 ml) were used. TLC analysis during the reaction showed no consumption of **3a**. NMR analysis of the crude reaction material after 20 h showed only unreacted starting materials.

Acid Test

In a slight modification to the general procedure 1, galactal **3a** (50 mg, 0.12 mmol), an anhydrous CH_2Cl_2 solution of galactose acceptor **4** (0.4M, 0.3 ml, 0.12 mmol) and $Et_3N{\cdot}HCl$ (20 mg, 0.14 mmol) ($pK_a = 9.0$, DMSO).[30] Analysis of the ^1H NMR spectrum of the crude mixture after 4 h showed only the starting materials.

Anomerisation tests

α/β mixture of 6-*O*-(3,4,6-Tri-*O*-benzyl-D-lyxo-hexapyranosyl)-1,2:3,4-di-*O*-isopropylidene-α-D-galactopyranose

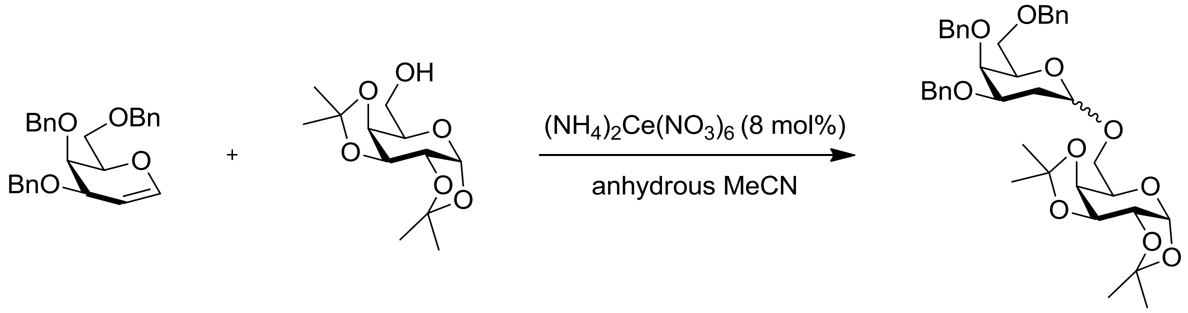

In a slight modification to general procedure 1,[31] galactal donor **3a** (76 mg, 0.18 mmol), and an anhydrous CH_3CN solution of galactose acceptor **4** (0.25M, 0.75 ml, 0.19 mmol) and cerium ammonium nitrate (8 mg, ~8 mol%) were used. Purification by column chromatography (4:1; cyclohexane/ethyl acetate) yielded the desired product α/β-**5a** as a pale yellow oil (53 mg, 43% yield, α/β = 5.3:1). Relevant NMR signals: ^{1}H NMR (500 MHz, Chloroform-*d*) δ 5.53 (d, *J* = 5.0 Hz, 0.18H, H-1 (β)), 5.51 (d, *J* = 5.0 Hz, 1H, H-1 (α)), 5.03 (d, *J* = 2.9 Hz, 1H, H-1' (α)), 2.12 – 2.06 (m, 0.18H, H-2a' or b' (β)), 2.06 – 1.98 (m, 1H, H-2b' (α)); ^{13}C NMR (126 MHz, Chloroform-*d*) δ 100.9 (C-1', $^1J_{C1,H1}$ = 160 Hz (β), from coupled HSQC), 97.6 (C-1', $^1J_{C1,H1}$ = 170 Hz (α), from coupled HSQC), 96.5(C-1, $^1J_{C1,H1}$ = 175 Hz (α), from coupled HSQC), 32.6 (C-2'(β anomer)), 31.1 (C-2' (α anomer)).

Disaccharide Mixture Submitted to Standard Reaction Conditions

In a slight modification to the general procedure 1, the α/β-**5a** (53 mg, 0.08 mmol) was submitted to the standard 2-thiouracil-catalysed glycosylation conditions. 2-Thiouracil (0.1 mg, 1 mol%) and anhydrous CH_2Cl_2 (0.31M, 0.26 ml) were used. Analysis of the ^{1}H NMR spectrum of the crude mixture after 16 h showed only the unchanged starting material α/β-**5a** (no change in the α/β ratio was observed).

3,4,6-Tri-*O*-benzyl-2-deoxy-α/β-D-galactopyranosyl *p*-toluenesulfonamide 12

Following a literature procedure,[25] the sulfonamide **12** was prepared as an α/β mixture (α:β = 8:92). The title compound was subjected to the standard 2-thiouracil-catalysed conditions (General Procedure 3). The reaction mixture was heated to reflux for 18 h. Analysis of the ^{1}H NMR spectrum of the crude reaction mixture showed no significant change in the α/β ratio.

1,1'-Linked disaccharides

We note that 1,1'-linked disaccharides did undergo anomerisation under extended reaction times with the amount of α,α increasing relative to α,ß over time.

Test to probe the possibility of a reversible reaction (cross-over experiment)

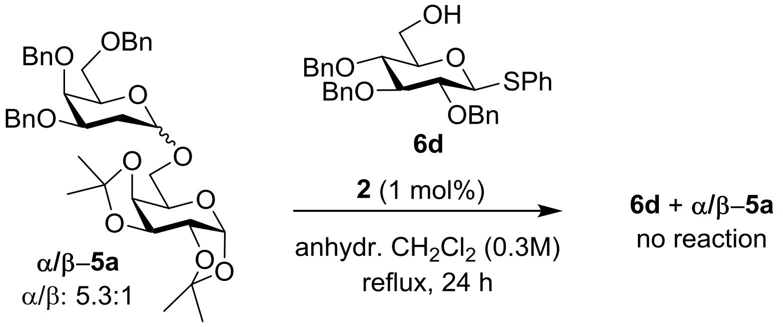

In a slight modification to general procedure 1, the α/β-**5a** (53 mg, 0.08 mmol), phenyl 2,3,4-tri-*O*-benzyl-β-D-thioglucopyranoside **6d** (43 mg, 0.08 mmol), 2-thiouracil (0.1 mg, 1 mol%) and anhydrous CH_2Cl_2 (0.31M, 0.26 ml) were used. Analysis of the ^1H NMR spectrum of the crude mixture after 24 h showed only the unchanged starting materials. A new disaccharide had not been formed.

Cross-over and Catalyst poison test

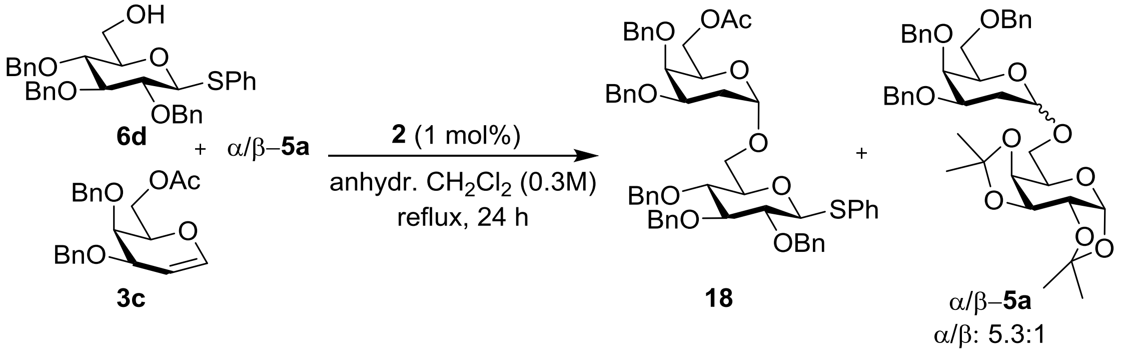

In a slight modification to general procedure 1, the α/β-**5a** (53 mg, 0.08 mmol), 6-*O*-acetyl-1,2-dideoxy-3,4-di-*O*-benzyl-D-*lyxo*-hexenopyranose **5c** (30 mg, 0.08 mmol), phenyl 2,3,4-tri-*O*-benzyl-β-D-thioglucopyranoside **6d** (43 mg, 0.08 mmol), 2-thiouracil (0.1 mg, 1 mol%) and anhydrous CH_2Cl_2 (0.31M, 0.26 ml) were used. Analysis of the ^1H NMR spectrum of the crude mixture after 24 h showed the disaccharide α/β-**5a** remained unchanged and a new disaccharide **18** had formed. Relevant signals: ^1H NMR (500 MHz, Chloroform-*d*) δ 5.06 (d, *J* = 2.6 Hz, 1H, H-1' (α) of newly formed disaccharide **18**), 4.68 (H-1 of newly formed disaccharide, observed in HSQC spectrum); ^{13}C NMR (126 MHz, Chloroform-*d*) δ 98.3 (C-1' of newly formed disaccharide **18**, observed in HSQC spectrum), 87.1 (C-1 of newly formed disaccharide **18** observed in HSQC spectrum). These signals showed that no crossover between the disaccharides occurred (i.e., disaccharides **7d**, **5c** were not formed).[5]

Investigation into the Stereochemistry of Alcohol Addition (*syn vs. anti*-addition)

Methyl 3,4,6-tri-*O*-benzyl-α-D-*lyxo*-hexapyranoside (19)

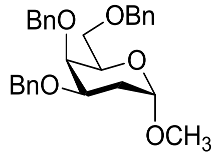

In a slight modification to General Procedure 3, galactal donor **3a** (250 mg, 0.6 mmol), anhydrous CH_3OH (20 µl, 0.49 mmol) and 2-thiouracil (0.7 mg, 6 µmol, 1 mol%) were weighed into a crimp top vial. The vial was sealed and the atmosphere was changed to nitrogen. The mixture was dissolved in anhydrous CH_2Cl_2 (2.5M w.r.t. acceptor, 0.2 ml) and heated at reflux for 18 h. Purification by column chromatography (95:5 to 9:1; pentane/ethyl acetate (unoptimised)) afforded the product as a yellow oil (180 mg, 82% yield).

R_f = 0.11 (95:5; pentane/ethyl acetate); ^{1}H NMR (500 MHz, Chloroform-*d*) δ 7.39 – 7.18 (m, 15H), 4.93 (d, *J* = 11.7 Hz, 1H, OC*H*HPh), 4.87 (d, *J* = 3.1 Hz, 1H, H-1), 4.62 (d, *J* = 11.6 Hz, 1H, OC*H*HPh), 4.59 (s, 2H, 2 × OC*H*HPh), 4.51 (d, *J* = 11.8 Hz, 1H, OC*H*HPh), 4.43 (d, *J* = 11.8 Hz, 1H, OC*H*HPh), 3.95 – 3.84 (m, 3H, H-3, H-4, H-5), 3.60 (dd, *J* = 9.4, 6.6 Hz, 1H, H-6a), 3.58 (dd, *J* = 9.4, 6.3 Hz, 1H, H-6b), 3.32 (s, 3H, OCH_3), 2.22 (td, *J* = 12.4, 3.6 Hz, 1H, H-2a), 1.99 (dd, *J* = 12.7, 4.5 Hz, 1H, H-2b); ^{13}C NMR (126 MHz, Chloroform-*d*) δ 139.0 (4° C), 138.7 (4° C), 138.3 (4° C), 128.53 (CH), 128.51 (CH), 128.37 (CH), 128.34 (CH), 127.9 (CH), 127.8 (CH), 127.64 (CH), 127.63 (CH), 127.4 (CH), 99.1 (C-1), 74.9 (C-3 or C-4), 74.4 (PhCH_2), 73.6 (PhCH_2), 73.2 (C-3 or C-4), 70.6 (PhCH_2), 69.9 (C-5), 69.8 (C-6), 54.9 (OCH_3), 31.3 (C-2); ESI-HRMS for $C_{28}H_{32}NaO_5^+$ (MNa^+) calculated: 471.2147; found: 471.2126. Proton and carbon NMR data were consistent with the literature data with the exception of coupling constant assignments for H-6a/b (AB pattern); reported as 3.60 (dd, *J* = 6.4, 1.8 Hz, 2H, H-6a, H-6b) and 3.59 (dd, *J* = 6.5, 3.0 Hz, 2H), respectively.[32,33]

Control Reaction of galactal 3a with CD_3OD in the absence of catalyst

In a slight modification to General Procedure 3, galactal donor **3a** (250 mg, 0.59 mmol) and CD_3OD (20 µl, 0.49 mmol) were weighed into a crimp top vial. The vial was sealed and the atmosphere was changed to nitrogen. The mixture was dissolved in anhydrous CH_2Cl_2 (2.5M w.r.t. acceptor, 0.2 ml) and heated at reflux for 24 h. TLC and ^{1}H NMR analysis of the crude reaction mixture showed the desired product had not formed and some breakdown of the galactal donor had occurred (aldehyde peak at 10.1 ppm in the ^{1}H NMR spectrum). The mixture was heated at reflux for a further 24 h, however TLC and ^{1}H NMR showed no desired product was formed and further breakdown of the galactal donor was observed.

d_3-Methyl (OCD$_3$) 3,4,6-tri-*O*-benzyl-α-D-*lyxo*-hexapyranoside (*syn/anti*-[^{2}H]-19) and d_3-Methyl (OCD$_3$) 3,4,6-tri-*O*-benzyl-(2-^{2}H)-α-D-*lyxo*-hexapyranoside (CD$_3$-19) using 2 as catalyst

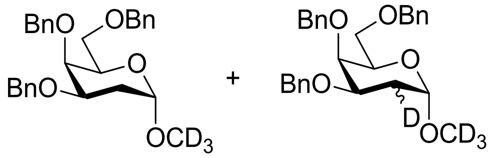

In a slight modification to General Procedure 3, glycal donor **3a** (246 mg, 0.59 mmol), CD_3OD (20 µl, 0.49 mmol) and 2-thiouracil (0.8 mg, 6 µmol, 1 mol%) were weighed into a crimp top vial. The vial was sealed and the atmosphere was changed to nitrogen. The mixture was dissolved in anhydrous CH_2Cl_2 (2.5M w.r.t. acceptor, 0.2 ml) and heated at reflux for 18 h. Purification by column chromatography (95:5 to 9:1; pentane/ethyl acetate (unoptimised)) afforded the products *syn/anti*-[^{2}H]-**19** and CD$_3$-**19** as a colourless oil (130 mg, 59% yield). Analysis of the ^{1}H NMR spectrum showed the ratio of ^{2}H-equatorial/^{2}H-axial in [^{2}H]-**19** to be approximately 95:5 with deuterium incorporation at C-2 approximately 88% (ratio of [^{2}H]-**19** to CD$_3$-**19**) (see excerpts from spectra below).

R_f = 0.11 (95:5; pentane/ethyl acetate); ^{1}H NMR (500 MHz, Chloroform-*d*) δ 7.37 – 7.20 (m, 15H, Ph), 4.93 (d, *J* = 11.6 Hz, 1H, OC*H*HPh), 4.87 (d, *J* = 3.6 Hz, 1H, H-1), 4.62 (d, *J* = 11.6 Hz, 1H, OC*H*HPh), 4.59 (s, 2H, 2 × OC*H*HPh), 4.51 (d, *J* = 11.8 Hz, 1H, OC*H*HPh), 4.43 (d, *J* = 11.8 Hz, 1H, OC*H*HPh), 3.94 – 3.84 (m, 3H, H-3, H-4, H-5), 3.60 (dd, *J* = 9.4, 6.6 Hz, 1H, H-6a), 3.57 (dd, *J* = 9.4, 6.3 Hz, 1H, H-6b), 2.27 – 2.16 (m, 1H, H-2a, H_{ax}-CH_{eq} and H_{ax}-C[^{2}H]$_{eq}$), 1.99 (app dd, *J* = 12.8, 4.4 Hz, 0.18H, H_{eq}-CH_{ax} and H_{eq}-C[^{2}H]$_{ax}$); ^{13}C NMR (126 MHz, Chloroform-*d*) δ 139.0 (4° C), 138.7 (4° C), 138.3 (4° C), 128.53 (CH), 128.50 (CH), 128.4 (CH), 128.3 (CH), 127.9 (CH), 127.8 (CH), 127.64 (CH), 127.63 (CH), 127.41 (CH), 99.0 (C-1), 74.8 (C-3 or C-4), 74.4 (Ph*C*H$_2$), 73.6 (Ph*C*H$_2$), 73.1 (C-3 or C-4), 70.6 (Ph*C*H$_2$), 69.9 (C-5), 69.8 (C-6), 54.1 (hept, *J* = 21.6 Hz, CD$_3$), 31.3 (C-2(H)), 30.9 (t, *J* = 19.5 Hz, C-2(D)); ESI-HRMS for $C_{28}H_{28}{}^2H_4NaO5^+$ (MNa$^+$) calculated: 475.2399; found: 475.2418.

d_3-Methyl (OCD$_3$) 3,4,6-tri-*O*-benzyl-α-D-*lyxo*-hexapyranoside (*syn/anti*-[^{2}H]-19) and d_3-Methyl (OCD$_3$) 3,4,6-tri-*O*-benzyl-(2-^{2}H)-α-D-*lyxo*-hexapyranoside (CD$_3$-19) using 1 as catalyst

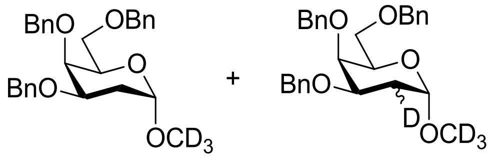

In a slight modification to General Procedure 3, galactal donor **3a** (246 mg, 0.59 mmol), CD_3OD (20 µl, 0.49 mmol) and Schreiner's catalyst **1** (0.8 mg, 6 µmol, 1 mol%) were weighed into a crimp top vial. The vial was sealed and the atmosphere was changed to nitrogen. The mixture was dissolved in anhydrous CH_2Cl_2 (2.5M w.r.t. acceptor, 0.2 ml) and

heated at reflux for 21 h. Purification by column chromatography (98:2 to 4:1; cyclohexane/ethyl acetate (unoptimised)) afforded the products *syn/anti*-[^{2}H]-**19** and CD_3-**19** as a colourless oil (100 mg, 45% yield). Analysis of the ^{1}H NMR spectrum showed the ratio of ^{2}H-equatorial/^{2}H-axial in [^{2}H]-**19** to be approximately 93:7 with deuterium incorporation at C-2 approximately 85% (ratio of [^{2}H]-**19** to CD_3-**19**) (see excerpts from spectra below). The proton and carbon NMR data were consistent with the data provided above when 2-thiouracil was used as a catalyst for the reaction.

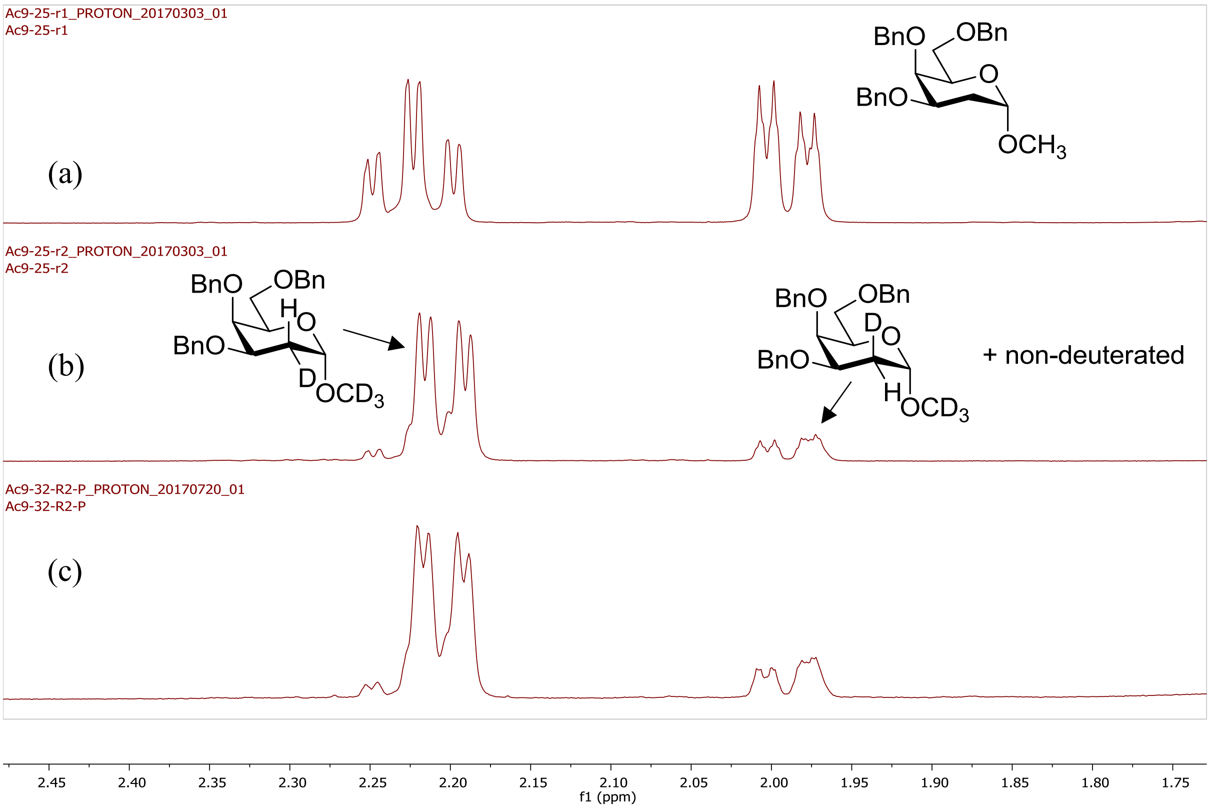

Figure S2: Excerpts from ^{1}H NMR spectra for investigation of stereochemistry of alcohol addition ($CDCl_3$, 500 MHz): (a) non-deuterated **19**. (b) syn/anti-[^{2}H]-**19** and **19**; with thiouracil as catalyst. (c) *syn/anti*-[^{2}H]-**19** and **19**; with Schreiner's catalyst. All ^{1}H NMR spectra are of products following column chromatography.

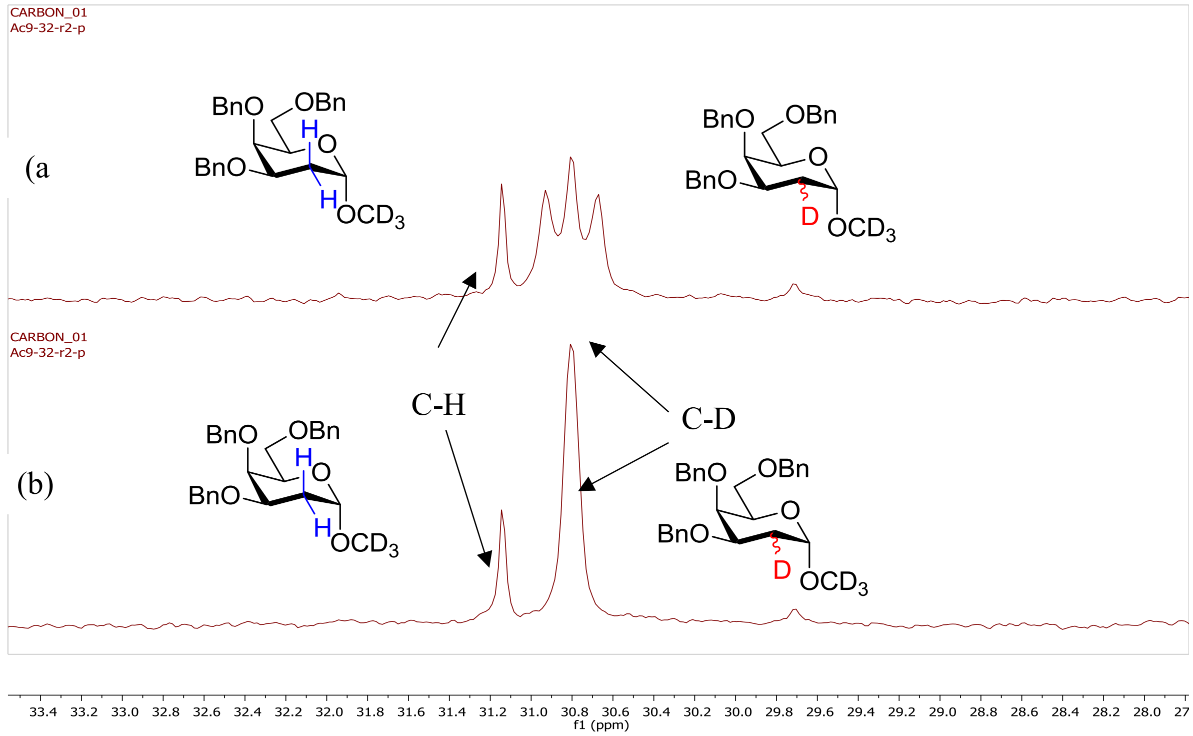

Figure S3: Excerpts from ^{13}C NMR spectra for mixture of syn/anti-[^{2}H]-19 and CD_3-19 ($CDCl_3$, 151 MHz): (a) ^{13}C NMR spectrum C-D coupled; (b) ^{13}C NMR spectrum C-D decoupled.

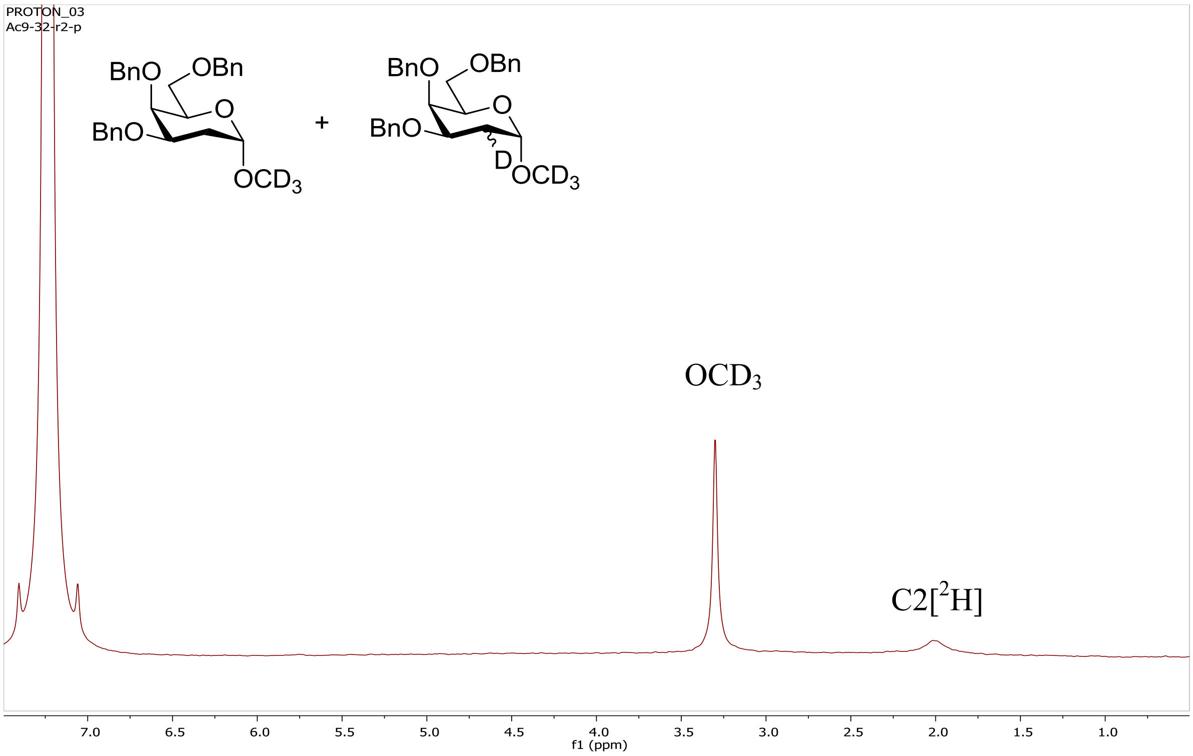

Figure S4: ^{2}H NMR spectrum ($CDCl_3$, 92.3 MHz): mixture of compounds *syn/anti*-[^{2}H]-19 and CD_3-19

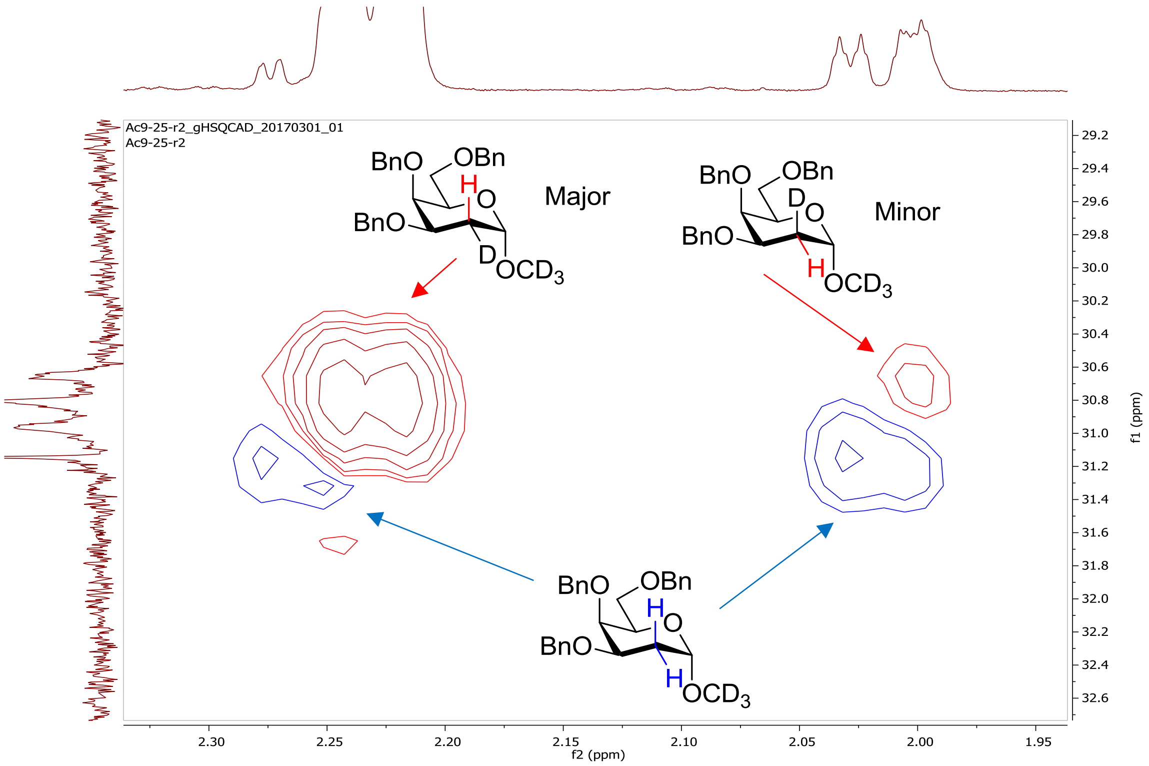

Figure S5: HSQC (500 MHz, Chloroform-d) showing the presence of *syn*/*anti*-[^{2}H]-**19** & CD_3-**19**

Probing *syn*/*anti* addition in the synthesis of 1,1'-linked disaccharides

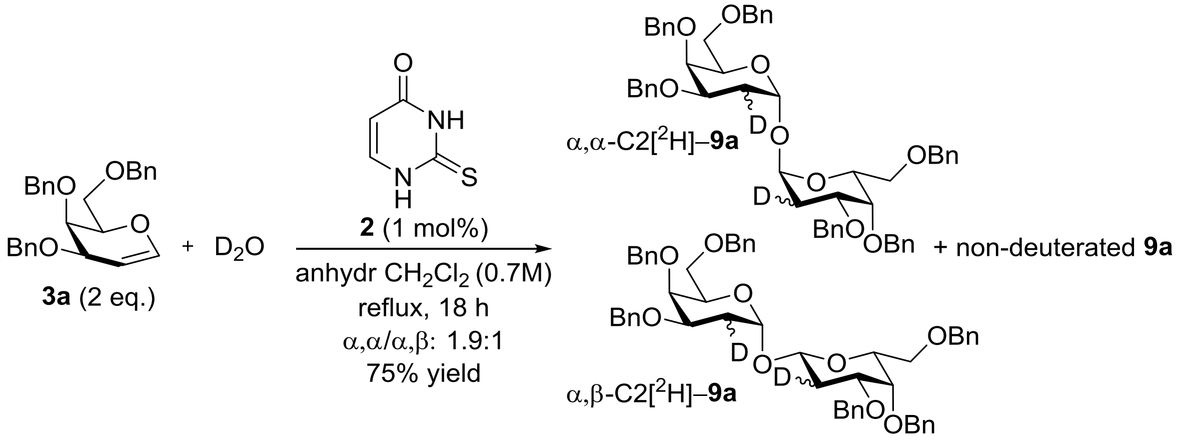

In a slight modification to General Procedure 1, galactal **3a** (251 mg, 0.603 mmol) was weighed into the reaction flask and dried under vacuum for 30 min. The flask was then switched to a N_2 atmosphere and **3a** was dissolved in anhydrous CH_2Cl_2 (0.7M, 0.4 ml). 2-Thiouracil (0.4 mg, 3 μmol) was added to the reaction flask followed by D_2O (5.0 μl, 0.28 mmol). The mixture was heated to reflux for 18 h. Analysis of the ^{1}H NMR spectrum of the crude mixture showed the reaction was ~76% complete (based on the integration of the starting galactal **3a** (H-1) *vs.* products α,α:α,β-**9a** (H-1)). The α,α/α,β ratio was 1.9:1. Purification by column chromatography (95:5 to 8:2; cyclohexane/ethyl acetate) gave the

desired products as colourless oils; α,α-C2[^{2}H]-**9a** (110 mg, 43% yield); mixed fractions containing α,α/α,β- C2[^{2}H]-**9a** (30 mg, 12% yield), α,β-C2[^{2}H]-**9a** (50 mg, 21% yield).

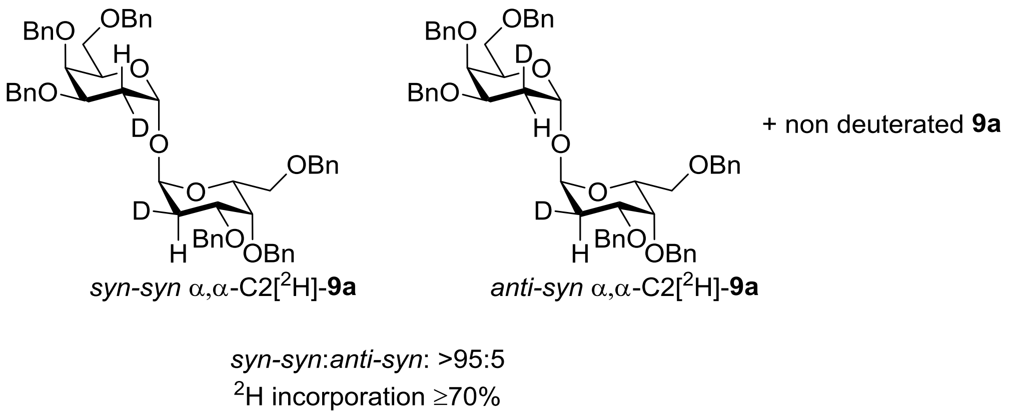

α,α-C2[^{2}H]-9a: R_f = 0.49 (85:15; cyclohexane/ethyl acetate); ^{1}H NMR (500 MHz, Chloroform-*d*) δ 7.43 – 7.16 (m, 15H, Ph), 5.24 (d, *J* = 3.7 Hz, 1H, H-1), 4.93 (d, *J* = 11.7 Hz, 1H, OC*H*HPh), 4.61 (d, *J* = 11.7 Hz, 1H, OC*H*HPh), 4.59 (d, *J* = 11.9 Hz, 1H, OC*H*HPh), 4.55 (d, J = 11.8 Hz, 1H, OC*H*HPh), 4.48 (d, *J* = 11.7 Hz, 1H, OC*H*HPh), 4.40 (d, *J* = 11.7 Hz, 1H, OC*H*HPh), 3.93 (br s, 1H, H-4), 3.88 – 3.79 (m, 2H, H-3, H-5), 3.62 (dd, *J* = 9.2, 7.3 Hz, 1H, H-6a), 3.53 (dd, *J* = 9.2, 5.7 Hz, 1H, H-6b), 2.22 (dd, *J* = 12.1, 3.7 Hz, H-2_{ax}, *syn-syn* α,α-C2[^{2}H]-**9a**), 1.84 (m, H-2_{eq}, *anti-syn* α,α-C2[^{2}H]-**9a**, identified from HSQC cross-peak ^{13}C 30.4 to ^{1}H 1.84);^{13}C NMR (126 MHz, Chloroform-*d*) δ 139.0 (4° C), 138.6 (4° C), 138.1 (4° C), 128.52 (CH), 128.51 (CH), 128.3 (CH), 128.2 (CH), 127.9 (CH), 127.9 (CH), 127.7 (CH), 127.6 (CH), 127.5 (CH), 93.47 (C-1), 74.42, 74.41 (C-3 or C-5, Ph*C*H$_2$), 73.7 (Ph*C*H$_2$), 73.04 (C-4), 70.6, 70.5 (C-3 or C-5, Ph*C*H$_2$), 69.4 (C-6), 30.6 (br peak, C-2 (C-D)); ESI-HRMS for $C_{54}H_{56}{}^2H_2NaO_9^+$ (MNa$^+$) calculated: 875.4120; found: 875.4064. The minimum level of deuterium incorporation was determined to be ≥ 70% from the combined integrations of the resonances for H-2_{ax} of *syn-syn* and H-2_{eq} of *anti-syn* α,α-C-2[^{2}H]-**9a** *vs.* the integration of the resonance for H-2_{eq} of α,α-C-2[^{1}H]-**9a** in the ^{1}H NMR spectrum. The ratio was also determined from the relative integrations of the same resonances in the HSQC spectrum and the same result was obtained; The ratio of *syn-syn*:*anti-syn* α,α-C2[^{2}H]-**9a** products was determined from the relative integrations of H-2_{ax} *syn-syn*:H-2_{eq} *anti-syn* in the HSQC spectrum. See below for excerpts from the NMR spectra.

Non-deuterated α,α-9a, signals observed: ^{1}H NMR (500 MHz, Chloroform-*d*) δ 2.24 (td, *J* = 12.4, 3.7 Hz, 1H, H-2a), 1.85 (dd, *J* = 12.6, 4.5 Hz, 1H, H-2b); ^{13}C NMR (126 MHz, Chloroform-*d*) δ 93.50 (C-1), 74.47, 73.07 (C-4), 31.0 (C-2 (H)),

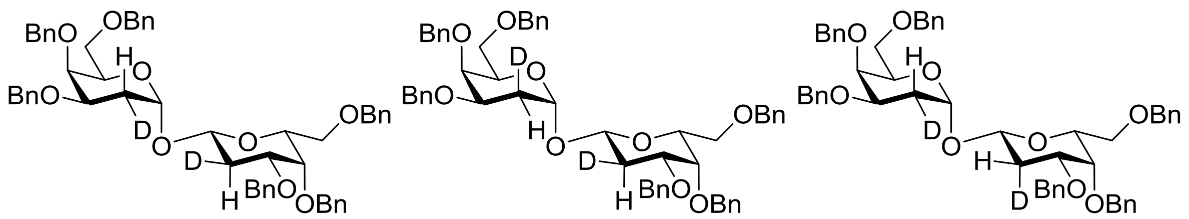

syn-syn α,β–C2[^{2}H]-**9a** *anti-syn* α,β–C2[^{2}H]-**9a** *syn-anti* α,β–C2[^{2}H]-**9a**

+ non-deuterated **9a**

^{2}H incorporation ≥68%

α,β-C2[^{2}H]-9a: R_f = 0.32 (85:15; cyclohexane/ethyl acetate); ^{1}H NMR (500 MHz, Chloroform-*d*) δ 7.52 – 6.99 (m, 30H, Ph), 5.19 (d, *J* = 3.6 Hz, 1H, H-1(α)), 4.90 (d, *J* = 11.6 Hz, 1H, OC*H*HPh), 4.89 (d, *J* = 11.6 Hz, 1H, OC*H*HPh), 4.64 (d, *J* = 11.7 Hz, 1H, OC*H*HPh), 4.61 – 4.57 (m, 2H, OC*H*HPh, H-1' (β)[HSQC shows a cross-peak for ^{13}C 99.5 and ^{1}H 4.59 (d, $J_{H1,H2}$ *ca.* 10Hz)], 4.57 – 4.52 (m, 4H, OC*H*HPh), 4.39 (d, *J* = 12.0 Hz, 1H, OC*H*HPh), 4.33 (app d, *J* = 10.6 Hz, 3H, 3 × OC*H*HPh), 4.25 (app t, *J* = 6.7 Hz, 1H, H-5 (α)), 3.98 (dd, *J* = 12.0, 2.6 Hz, 1H, H-3 (α)), 3.93 (br, s, 1H, H-4 (α)), 3.84 (d, *J* = 2.5 Hz, 1H, H-4'(β)), 3.67 – 3.54 (m, 2H, H-6a/a'), 3.54 – 3.44 (m, 4H, H-6b/b', H-3' (β), H-5' (β)), 2.18 (dd, J = 12.1, 3.7 Hz, 1H, H-2$_{ax}$ (α), *syn-syn* α,β-C2[^{2}H]-**9a**), 2.10 (dd, J = 12.2, 10.1 Hz, 1H, H-2$_{ax}$ (β), *syn-syn* α,β-C2[^{2}H]-**9a**), 2.00 (m, H-2$_{eq}$ (β), identified from HSQC cross-peak ^{13}C 32.5 to ^{1}H 2.00), 1.98 (m, H-2$_{eq}$ (α), identified from HSQC cross-peak ^{13}C 30.7 to ^{1}H 1.98); ^{13}C NMR (126 MHz, Chloroform-*d*) δ 139.1 (4° C), 139.0 (4° C), 138.7 (4° C), 138.5 (4° C), 138.4 (4° C), 138.1 (4° C), 128.53 (CH), 128.46 (CH), 128.42 (CH), 128.39 (CH), 128.25 (CH), 128.24 (CH), 128.23 (CH), 127.87 (CH), 127.78 (CH), 127.71 (CH), 127.63 (CH), 127.58 (CH), 127.56 (CH), 127.47 (CH), 127.40 (CH), 127.39 (CH), 99.50 (C-1' (β)), 98.14 (C-1 (α)), 77.4 (C-3' (β)), 74.60 (C-3 (α)), 74.5 (Ph*C*H$_2$), 74.4 (Ph*C*H$_2$), 74.1 (C-5' (β)), 73.5 (Ph*C*H$_2$), 73.3 (Ph*C*H$_2$), 73.10 (C-4 (α)), 71.41 (C-4'(β)), 70.6 (Ph*C*H$_2$), 70.3 (Ph*C*H$_2$), 70.2 (C-5 (α)), 69.2 (C-6 (α)), 68.7 (C-6' (β)), 32.8 (br peak, C-2' (β) (C-D)), 30.9 (br peak, C-2 (α) (C-D)); ESI-HRMS for $C_{54}H_{56}{}^2H_2NaO_9{}^+$ (MNa$^+$) calculated: 875.4120; found: 875.4091. The minimum level of deuterium incorporation was determined to be ≥68% from the relative integrations of the resonances for H-2$_{ax}$ (α) of *syn-syn* and H-2$_{eq}$ (α) of *anti-syn* α,β-C2[^{2}H]-**9a** *vs.* the resonance for H-2$_{eq}$ (α) of α,β-C2[^{1}H]-**9a** in the HSQC spectrum. It wasn't possible to determine a ratio of *syn-syn*:*anti:syn*.

Non-deuterated α,β-9a, signals observed: ^{1}H NMR (500 MHz, Chloroform-*d*) 2.19 (td, *J* = 12.4, 3.5 Hz, 1H, H-2a (α)), 2.15 – 2.07 (m, 1H, H-2a'(β)), 2.06 – 1.93 (m, 2H, H-2b (α), H-2b'(β));^{13}C NMR (126 MHz, Chloroform-*d*) δ 99.56 (C-1' (β)), 98.18 (C-1 (α)), 77.5 (C-3' (β)), 74.65 (C-3 (α)), 73.13 (C-4 (α)), 71.45 (C-4'(β)), 33.1 (C-2' (β) (H)), 31.3 (C-2 (α)),

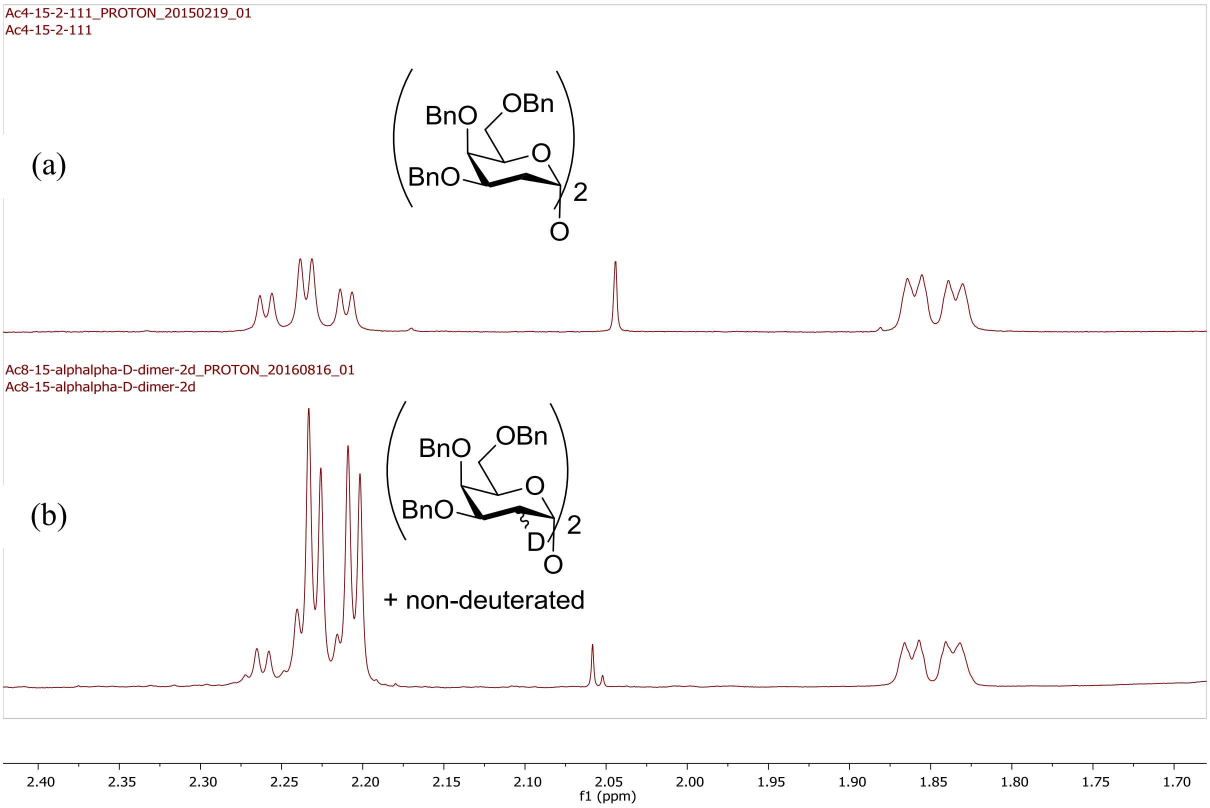

Figure S6: Excerpts from 1H NMR spectra for investigation of stereochemistry of H_2O addition in α,α-dimer **9a** formation: ($CDCl_3$, 500 MHz): (a) non-deuterated product α,α-**9a** (signal at 2.03 ppm corresponds to residual ethyl acetate); (b) deuterated products α,α-C2[^{2}H]-**9a** and α,α-dimer **9a**.

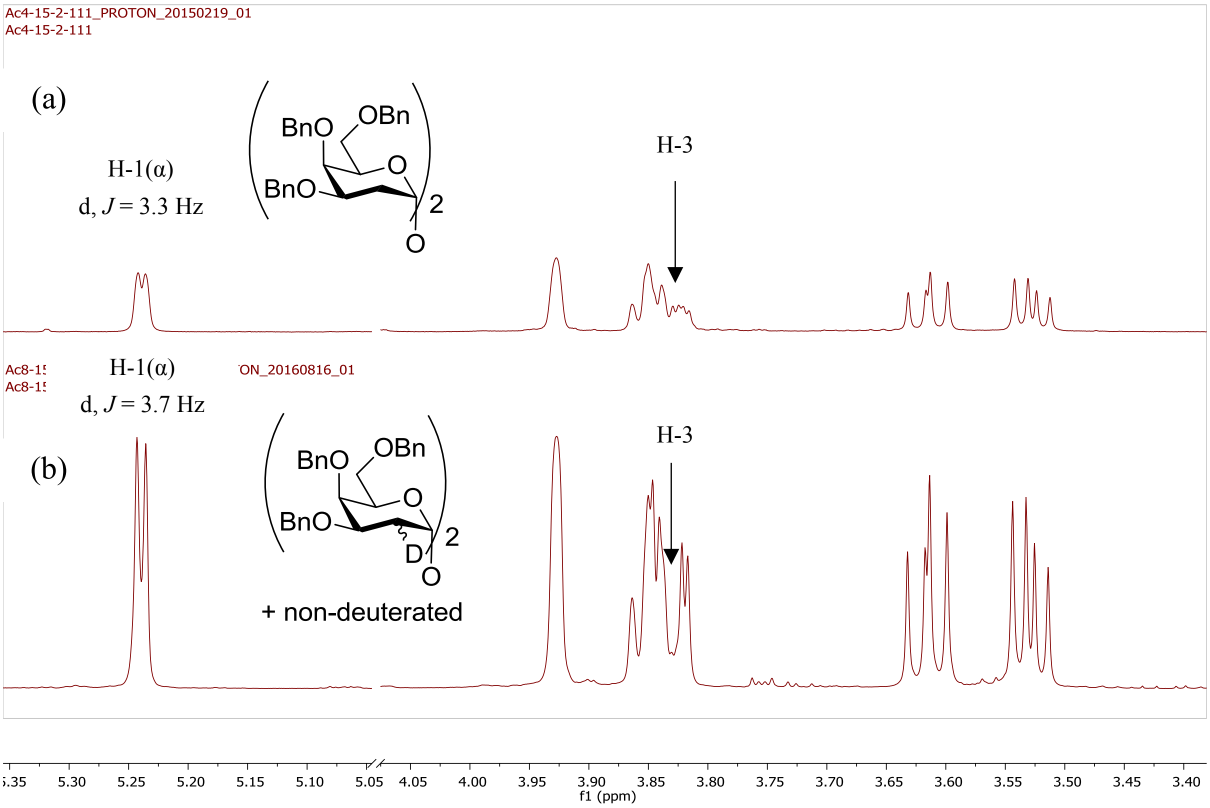

Figure S7: Excerpts from ^{1}H NMR spectra showing change in other 1H resonances ^{2}H incorporation at C-2 of α,α-**9a** ($CDCl_3$, 500 MHz): (a) α,α-**9a**; (b) α,α-C2[^{2}H]-**9a** and α,α-dimer **9a**.

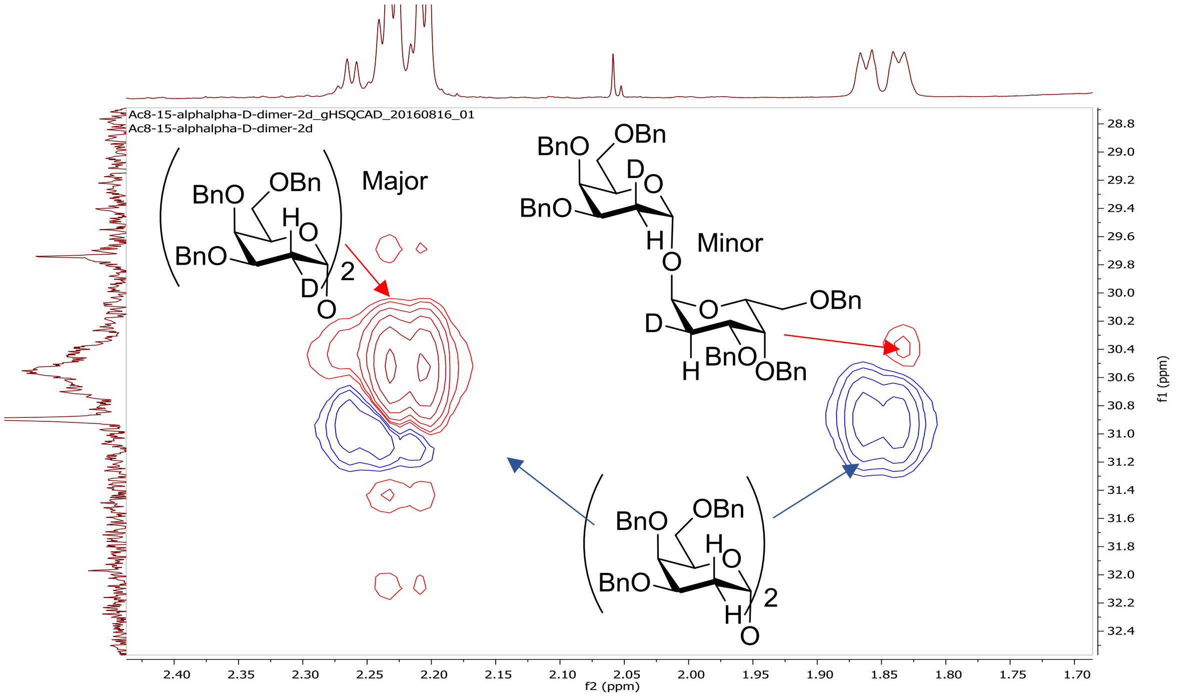

Figure S8: HSQC of *syn-syn*, *anti-syn* α,α-C2[^{2}H]-**9a** and α,α-dimer-**9a**.

Synthesis of Monothiophthalimide 13

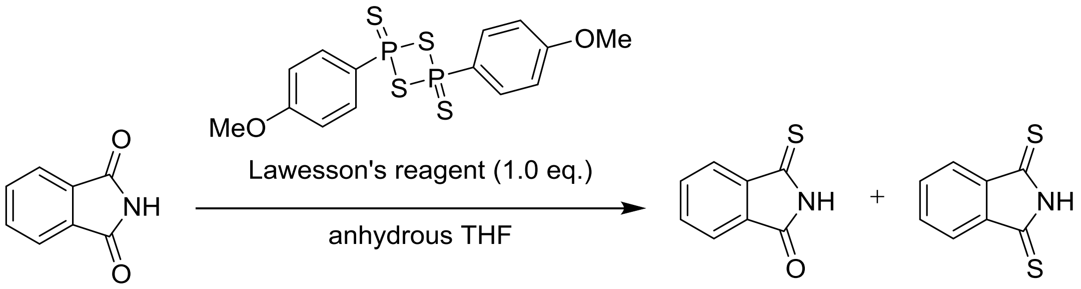

Following the literature procedure,[34] a round bottom flask equipped with a magnetic stir-bar, a condenser and gas inlet (all flame-dried) was placed under a N_2 atmosphere. Phthalimide (3.00 g, 20.4 mmol) was weighed into the reaction flask and dissolved in anhydrous THF (45 ml). Lawesson's reagent (8.34 g, 20.6 mmol) was added and the mixture was heated to 60 °C. The mixture was heated at 60 °C for 11 h. A strong colour change from yellow (time = 0 h) to dark purple (time = 11 h) was observed. TLC analysis (4:1; cyclohexane/ethyl acetate) showed that the phthalimide starting material (R_f = 0.14) remained and two new phthalimide-derived spots appeared (R_f = 0.25, 0.18). Several other spots related to Lawesson's reagent were also observed (R_f = 0.51, 0.41, 0.07 and a baseline spot). The reaction mixture was concentrated using a rotary evaporator. The dark solid obtained was purified by column chromatography (95:5; cyclohexane/ethyl acetate) which gave the desired product as a pink solid (1.56 g, 47% yield) and dithiophthalimide as a brown solid (0.93 g, 50% yield). Analysis of the ^{1}H NMR spectra showed low levels of impurities still present in both the mono- and di-thiophthalimide. The solids were recrystallised using toluene. Monothiophthalimide **13** was obtained as a pink solid (0.3 g, 9% yield). Dithiophthalimide **20** was obtained as a brown solid (0.15 g, 8% yield).

Monothiophthalimide (13)

R_f = 0.25 (4:1; cyclohexane/ethyl acetate); mp 176−177 °C (lit.[35] 174 °C (AcOH)) ^{1}H NMR (400 MHz, Chloroform-*d*) δ 9.05 (s, 1H, NH), 8.05 – 7.91 (m, 1H, CH), 7.85 – 7.70 (m, 3H, CH); ^{13}C NMR (101 MHz, Chloroform-*d*) δ 197.1 (C=S), 170.2 (C=O), 137.5 (4° C), 134.5 (CH), 133.9 (CH), 128.1 (4° C), 124.1 (CH), 123.2 (CH); Calc'd for C_8H_5NOS: C, 58.88; H, 3.04; N, 8.58; S, 19.65; (%), Found C, 58.49; H, 3.02; N, 8.36; S, 19.93 (%). Carbon NMR data were consistent with literature data.[36]

Dithiophthalimide (20)

R_f = 0.18 (4:1; cyclohexane/ethyl acetate); mp 197−198 °C (lit.[37] 199−201 °C (CH_2Cl_2)) ^{1}H NMR (400 MHz, Chloroform-*d*) δ 9.71 (s, 1H, NH), 7.96 – 7.82 (m, 2H, CH), 7.81 – 7.65 (m, 2H, CH); ^{13}C NMR (101 MHz, Chloroform-*d*) δ 197.5 (C=S), 135.2 (°C), 133.7 (CH), 123.3 (CH). Carbon NMR data were consistent with literature data.[36]

Control reaction with monothiophthalimide 13 instead of thiouracil as catalyst

In a slight modification to General Procedure 1, galactal donor **3a**, an anhydrous CH_2Cl_2 solution of galactose acceptor **4** and monothiophthalimide **13** (1 mol%) were used. The disaccharide **5a** was obtained in 84% yield after purification.

Probing Catalyst-Substrate Interactions using ^{1}H NMR spectroscopy

Under a nitrogen atmosphere, a solution of monothiophthalimide **13** (4.9 mg, 0.03 mmol) was prepared in CD_2Cl_2 (1.5 ml, concentration 0.02M) and dried over 4Å molecular sieves. An aliquot (0.7 ml) of this solution was analysed by ^{1}H NMR spectroscopy, the NH proton was observed at δ 8.84 ppm. A CD_2Cl_2 solution of galactal donor **3a** (24.0 mg, 0.058 mmol) and **13** was prepared from the parent stock solution (0.8 ml, 0.072M w.r.t. **3a**, and 0.02M w.r.t. **13**) and dried over 4Å molecular sieves. The mixture of **3a** and **13** was analysed by ^{1}H NMR spectroscopy. In the presence of galactal **3a** (3.7 equiv.), a small downfield shift for the NH proton of **13** was observed in the ^{1}H NMR spectrum (δ 8.84 changed to 8.94 ppm).

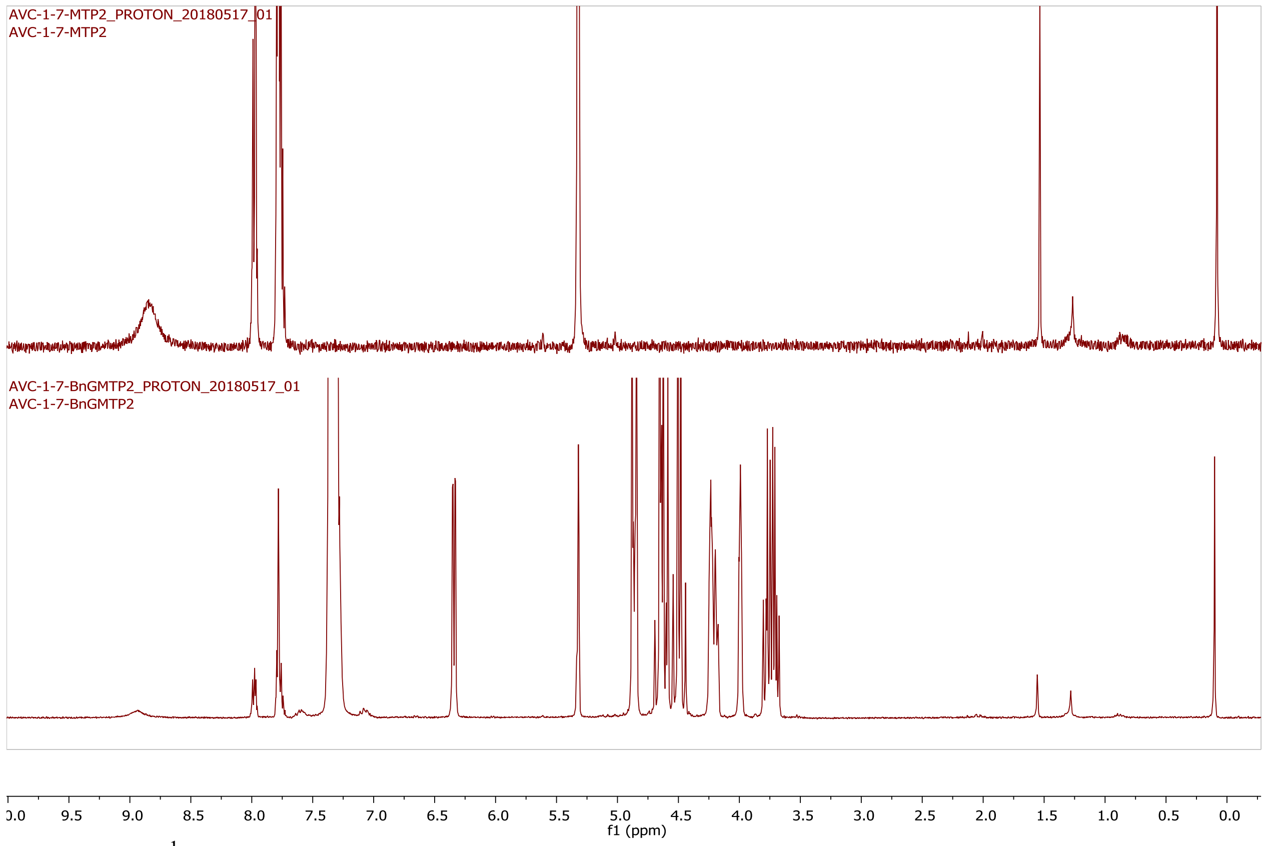

Figure S9: ^{1}H NMR (300 MHz, CD_2Cl_2; 8 scans; 25 second relaxation delay); (a) **13** (0.02M); (b) **13** (0.02M) + **3a** (0.073M); spectra are referenced to the CD_2Cl_2 solvent resonance (δ 5.32 ppm).

Under a nitrogen atmosphere, a solution of monothiophthalimide **13** (8.3 mg, 0.051 mmol) was prepared in CD_2Cl_2 (2.5 ml, concentration 0.02M) and dried over 4Å molecular sieves. An aliquot (0.7 ml) of this solution was analysed by ^{1}H NMR spectroscopy, the NH proton was observed at δ 8.86 ppm. A CD_2Cl_2 solution of diacetone galactose **4** (43.3 mg, 0.166

mmol) and **13** was prepared from the parent stock solution (1.0 ml, 0.166M w.r.t. **4**, and 0.02M w.r.t. **13**) and dried over 4Å molecular sieves. The solution of **13** and **4** was added (0.6 mL) to sample of **13** and the mixture was analysed by ^{1}H NMR spectroscopy. In the presence of alcohol **4** (3.7 equiv.), a downfield shift for the NH proton of **13** was observed in the ^{1}H NMR spectrum (δ 8.86 changed to 9.26 ppm). The OH proton also sharpened and showed a shift from 2.11 to 2.15 ppm.

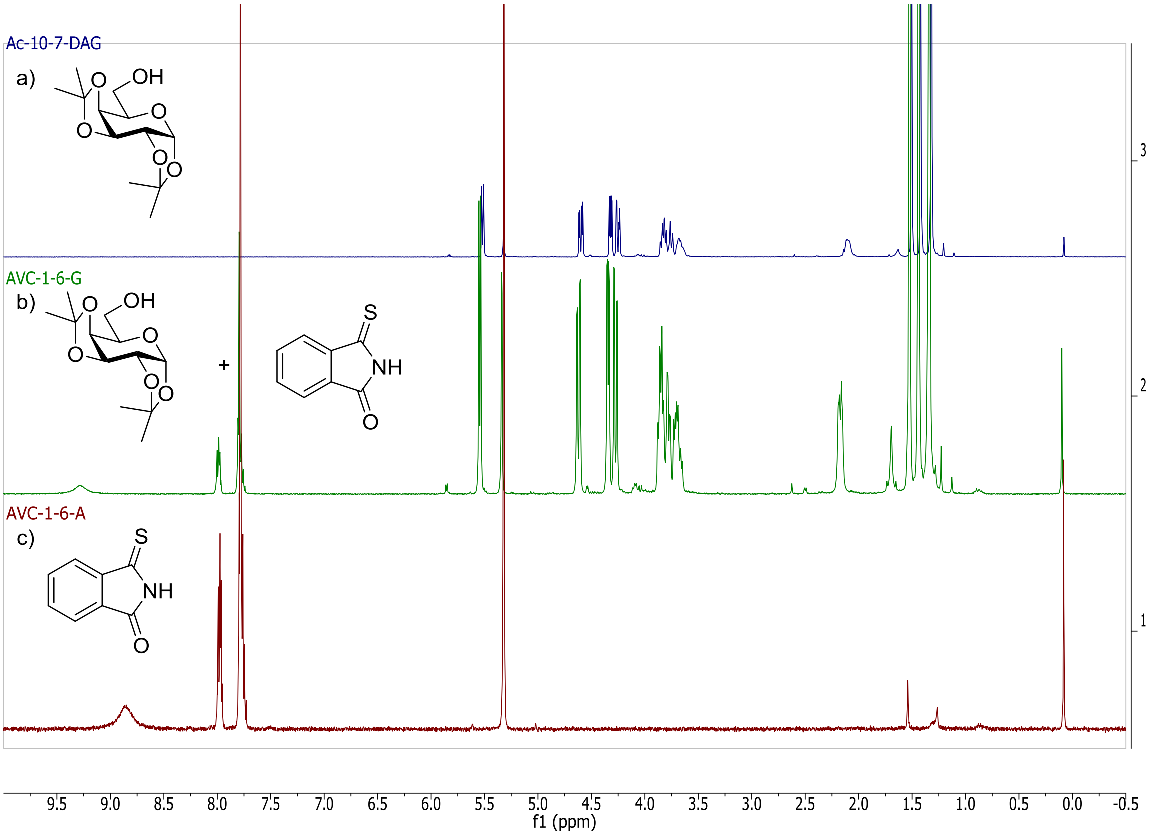

Figure S10: ^{1}H NMR (300 MHz, CD_2Cl_2; 8 scans; 25 second relaxation delay); (a) **4** (0.08M); (b) **13** (0.02M) + **4** (0.073M); (c) **13** (0.02M); spectra are referenced to the CD_2Cl_2 solvent resonance (δ 5.32 ppm).

References

1. W. L. F. Armarego, C. L. L. Chai, *Purification Of Laboratory Chemicals*; 7th ed.; Butterworth-Heinemann, Oxford, U.K., 2013; pp 717.

2. Pangborn, A. B.; Giardello, M. A.; Grubbs, R. H.; Rosen, R. K.; Timmers, F. J. *Organometallics* **1996**, *15*, 1518.

3. Colgan, A. C.; Müller-Bunz, H.; McGarrigle, E. M. *J. Org. Chem.* **2016**, *81*, 11394-11396.

4. Durantie, E.; Bucher, C.; Gilmour, R. *Chem. Eur. J.* **2012**, *18*, 8208–8215.

5. Balmond, E. I.; Coe, D. M.; Galan, M. C.; McGarrigle, E. M. *Angew. Chem. Int. Ed.* **2012**, *51*, 9152–9155.

6. Bucher, C.; Gilmour, R. *Angew. Chem. Int. Ed.* **2010**, *49*, 8724–8728.

7. Balmond, E. I.; Benito-Alifonso, D.; Coe, D. M.; Alder, R. W.; McGarrigle, E. M.; Galan, M. C. *Angew. Chem. Int. Ed.* **2014**, *53*, 8190-8194.

8. Thiem, J.; Klaffke, W.; Springer, D. *Carbohydr. Res.*, **1988**, *174*, 201-210.

9. Hanessian, S. Rogel, O. *J. Org. Chem.*, **2000**, *65*, 2667-2674.

10. Roën, A.; Padrón, J. I.; Vázquez, J. T. *J. Org. Chem.* **2003**, *68*, 4615–4630.

11. Matwiejuk, M.; Thiem, J. *Chem. Commun.* **2011**, *47*, 8379.

12. Garegg, P. J.; Iversen, T.; Oscarson, S. *Carbohydr. Res.* **1976**, *50* (2), C12–C14. Mydock, L. K.; Kamat, M. N.; Demchenko, A. V. *Org. Lett.* **2011**, *13*, 2928–2931.

13. Ogawa, T.; Kaburagi, T. *Carbohydr. Res.* **1982**, *103*, 53–64.

14. Wang, C.-C.; Kulkarni, S. S.; Lee, J.-C.; Luo, S.-Y.; Hung, S.-C. *Nat. Protoc.* **2008**, *3*, 97–113.

15. Garegg, P. J.; Hultberg, H.; Wallin, S. *Carbohydr. Res.* **1982**, *108*, 97–101.

16. Zhang, Z.; Yua, B.; Schmidt, R. R. *Synthesis* **2006**, *8*, 1301-1306; Bao, K.; Fan, A.; Dai, Y.; Zhang, L.; Zhang, W.; Cheng, M.; Yao, X. *Org. Biomol. Chem.* **2009**, *7*, 5084-5090.

17. Sherry, B. D.; Loy, R. N.; Toste, F. D. *J. Am. Chem. Soc.* **2004**, *126*, 4510-45115.

18. Kim, H.; Hongbin, M.; Lee, C., *J. Am. Chem. Soc.* **2004**, *126*, 1336-1337.

19. Nogueira, J. M.; Bylsma, M.; Bright, D. K.; Bennett, C. S. *Angew. Chem. Int. Ed.*, **2016**, *55*, 10088-10092.

20. Issa, J. P.; Bennet, C. S. *J. Am. Chem. Soc.*, **2014**, *136*, 5740-5744.

21. Schene, H.; Waldmann, H. *Synthesis*, **1999**, 1411-1422.

22. Lin, H. C.; Pan, J. F.; Chen, Y. B.; Lin, Z. P.; Lin, C. H. *Tetrahedron* **2011**, *67*, 6362-6368.

23. Palo-Nieto, C.; Sau, A.; Williams, R.; Galan, M. C. *J. Org. Chem.* **2017**, *82*, 407−414.

24. Nogueira, A. M.; Issa, J. P.; Chu, A. A.; Sisel, J. A.; Schum, R. S.; Bennett, C. S. *Eur. J. Org. Chem.* **2012**, 4927-4936.

25. Colinas, P. A.; Bravo, R. D. *Org. Lett.* **2003**, *5*, 4509-4511.

26. Bernardes, G. J. L.; Grayson, E. J.; Thompson, S.; Chalker, J. M.; Errey, J. C.; Oualid, F. E.; Ti, D.; Claridge, W.; Davis, B. G. *Angew. Chem. Int. Ed.* **2008**, *47*, 2244-2247.

27. Kuzuhara, H.; Fletcher, H. G. *J. Org. Chem.* **1967**, *32*, 2531-2534.

28. Jogula, S.; Dasari, B.; Khatravath, M.; Chandrasekar, G.; Kitambi, S. S.; Arya, P. *Eur. J. Org. Chem.* **2013**, *23*, 5036-5040.

29. Yoshimura, J.; Hara, K.; Sato, T.; Hashimoto, H. *Chem. Lett.* **1983**, *12*, 319–320.

30. Kolthoff, I. M.; Chantooni Jr, M. K.; Bhowmik, S. *J. Am. Chem. Soc.* **1968**, *90*, 23–28.

31. Pachamuthu, K.; Vankar, Y. D. *J. Org. Chem.* **2001**, *66*, 7511–7513.

32. Sau, A.; Williams, R.; Palo-Nieto, C.; Franconetti, A.; Medina, S.; Galan, M. C. *Angew. Chem. Int. Ed.* **2017**, *56*, 3640–3644.

33. Das, S.; Pekel, D.; Neudörfl, J.-M.; Berkessel, A. *Angew. Chem. Int. Ed.* **2015**, *54*, 12479–12483.

34. Yde, B.; Yousif, N. M.; Pedersen, U.; Thomsen, I.; Lawesson, S.-O. *Tetrahedron* **1984**, *40*, 2047–2052.

35. Drew, H. D. K.; Kelly, D. B. *J. Chem. Soc.* **1941**, 625–630.

36. Leznoff, C. C.; Greenberg, S.; Khouw, B.; Lever, B. P. *Can. J. Chem.* **1987**, *65*, 1705–1713.

37. Lin, P.-Y.; Ku, W.-S.; Shiao, M.-J. *Synthesis* **1992**, *12*, 1219–1220.

NMR Spectra of Compounds

NMR Spectra of Glycals

^{1}H NMR (400 MHz, $CDCl_3$), 3,4,6-tri-*O*-benzyl-D-galactal 3a

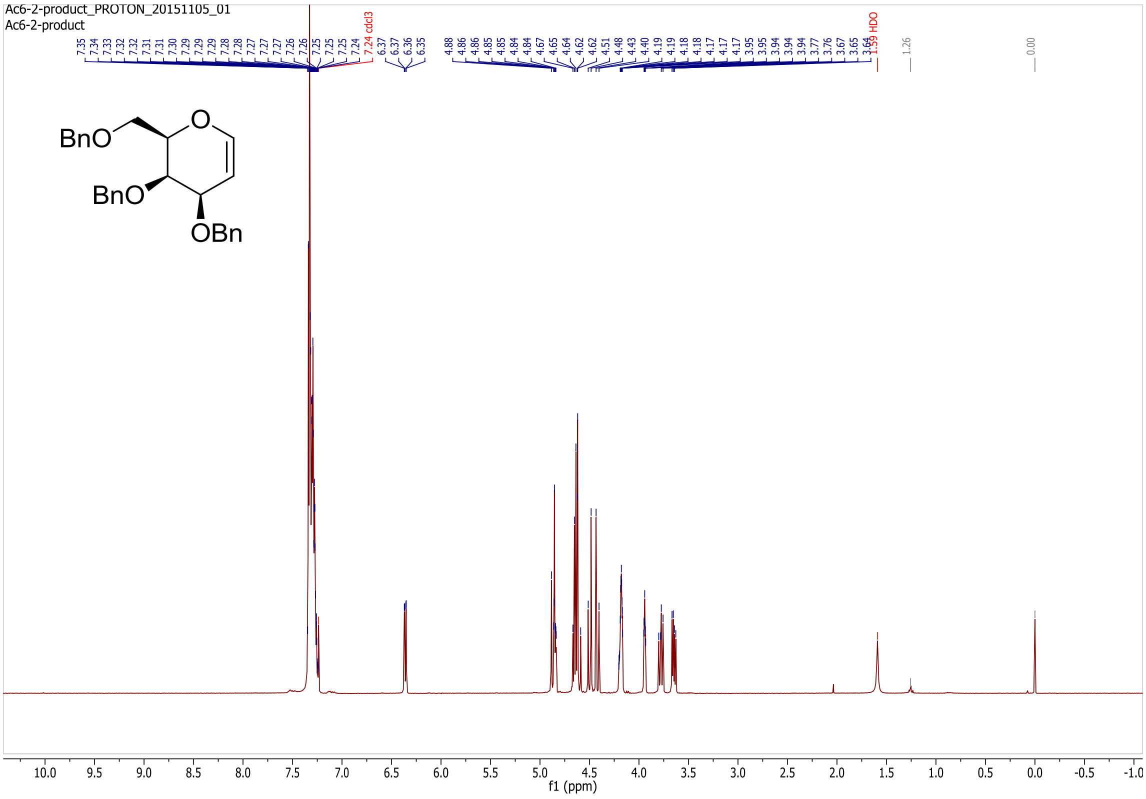

^{13}C NMR (101 MHz, $CDCl_3$), 3,4,6-tri-*O*-benzyl-D-galactal 3a

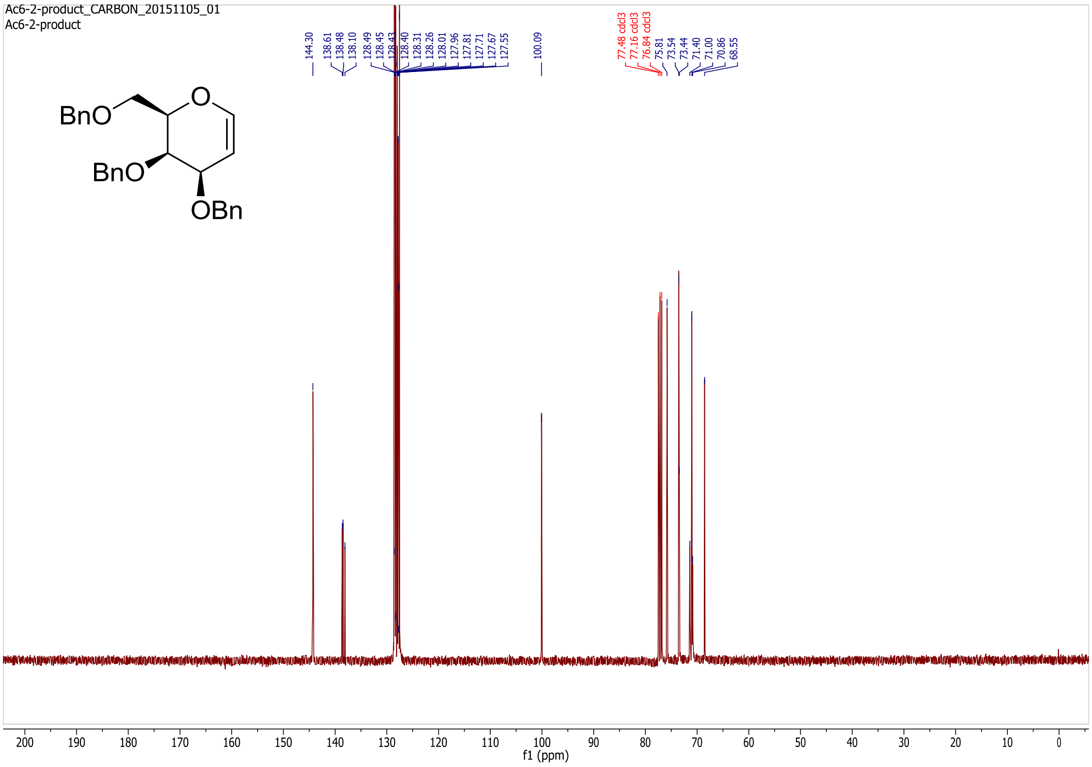

^{1}H NMR (400 MHz, $CDCl_3$), 3,4,6-tri-*O*-allyl-D-galactal 3b

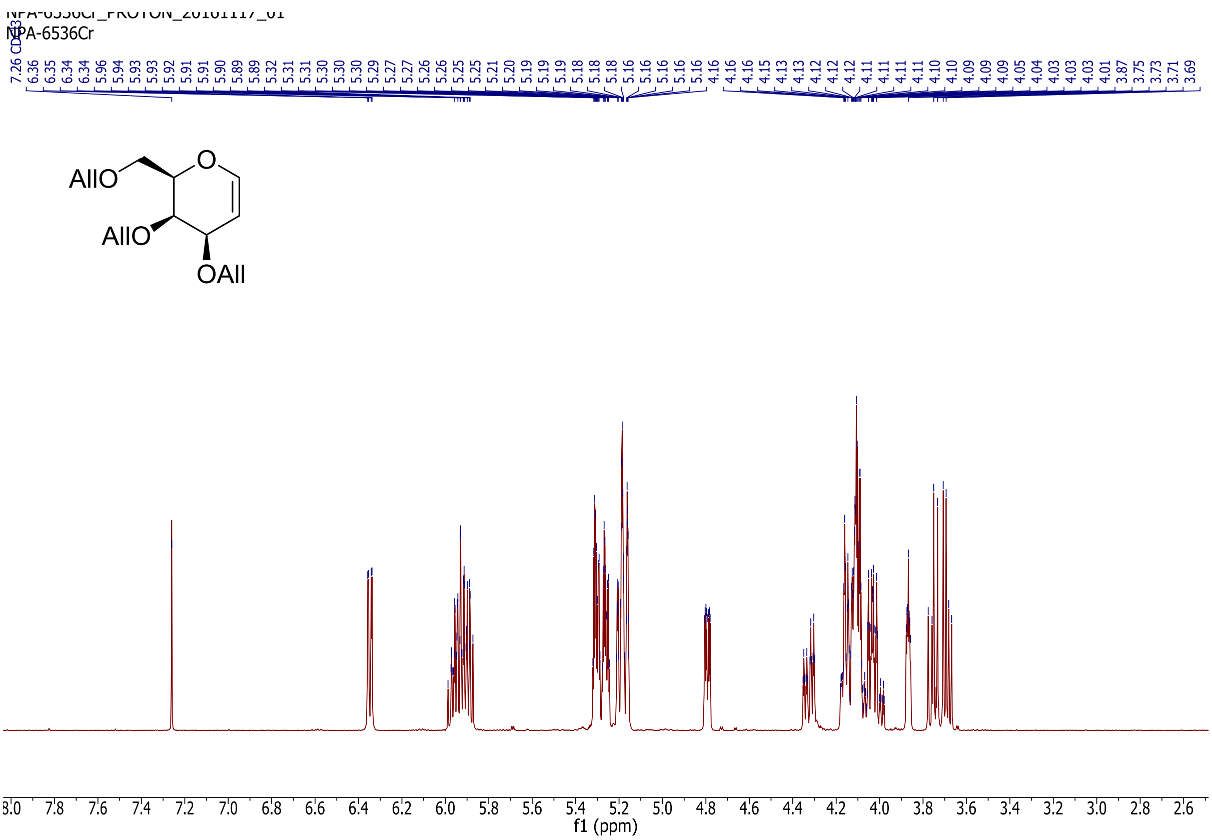

^{13}C NMR (101 MHz, $CDCl_3$), 3,4,6-tri-*O*-allyl-D-galactal 3b

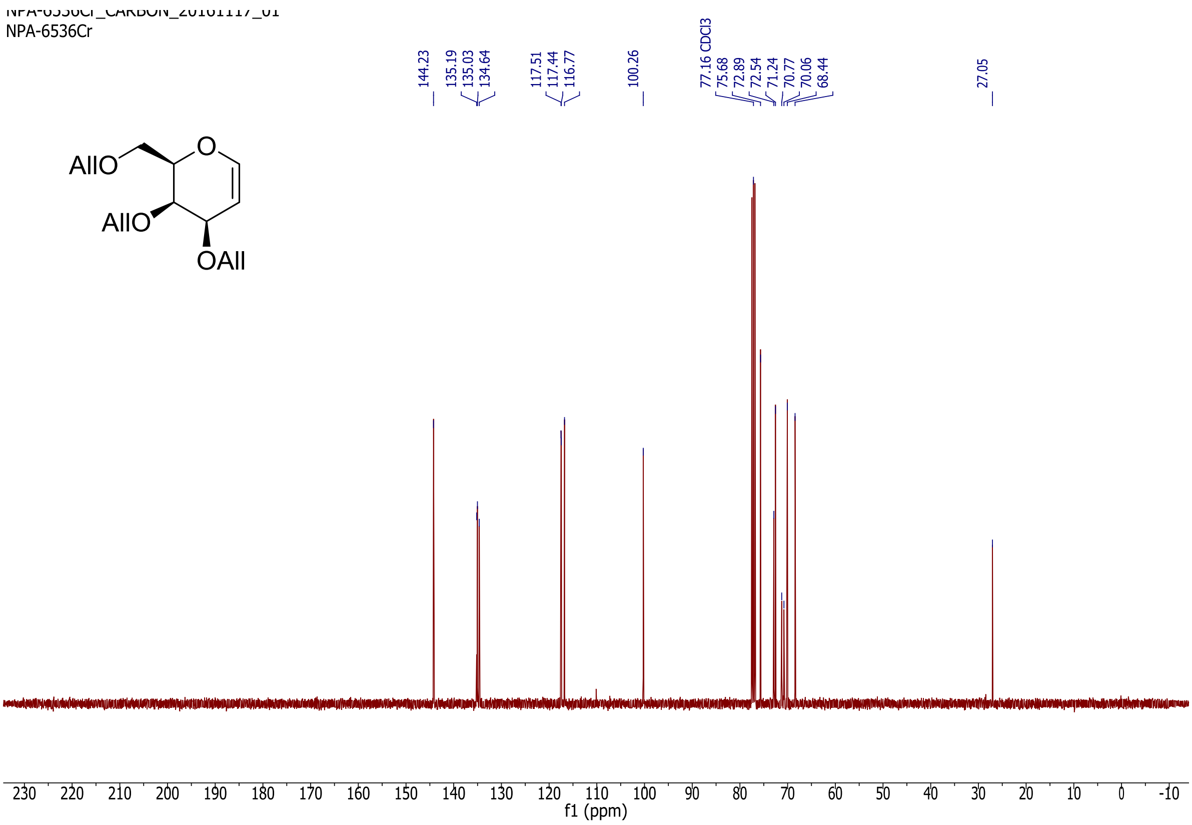

^{1}H NMR (500 MHz, $CDCl_3$), 1,5-anhydro-2-deoxy-3,4-di-*O*-benzyl-6-*O*-acetyl-D-*lyxo*-hex-1-enitol 3c

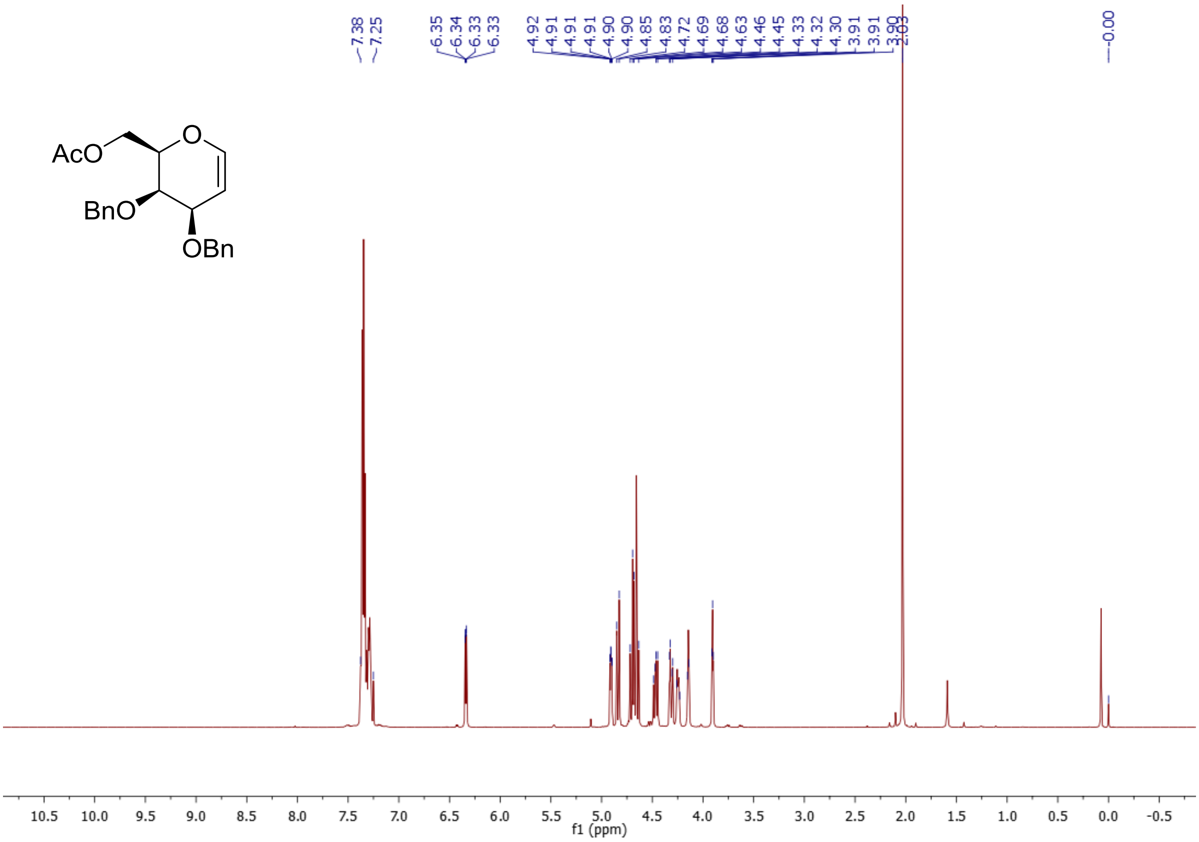

^{13}C NMR (125 MHz, $CDCl_3$), 1,5-anhydro-2-deoxy-3,4-di-*O*-benzyl-6-*O*-acetyl-D-*lyxo*-hex-1-enitol 3c

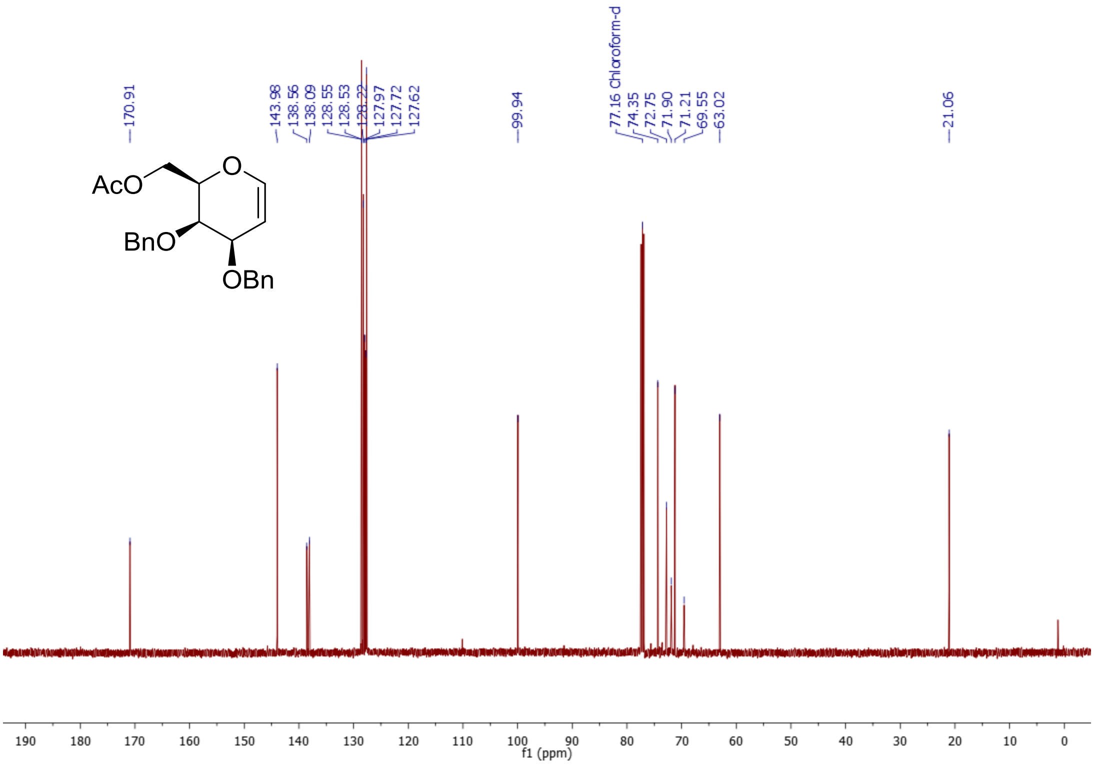

^{1}H NMR (500 MHz, $CDCl_3$), 1,5-Anhydro-2-deoxy-3,4,6-tri-*O*-*tert*-butyldimethylsilyl-D-*lyxo*-hex-1-enitol 3d

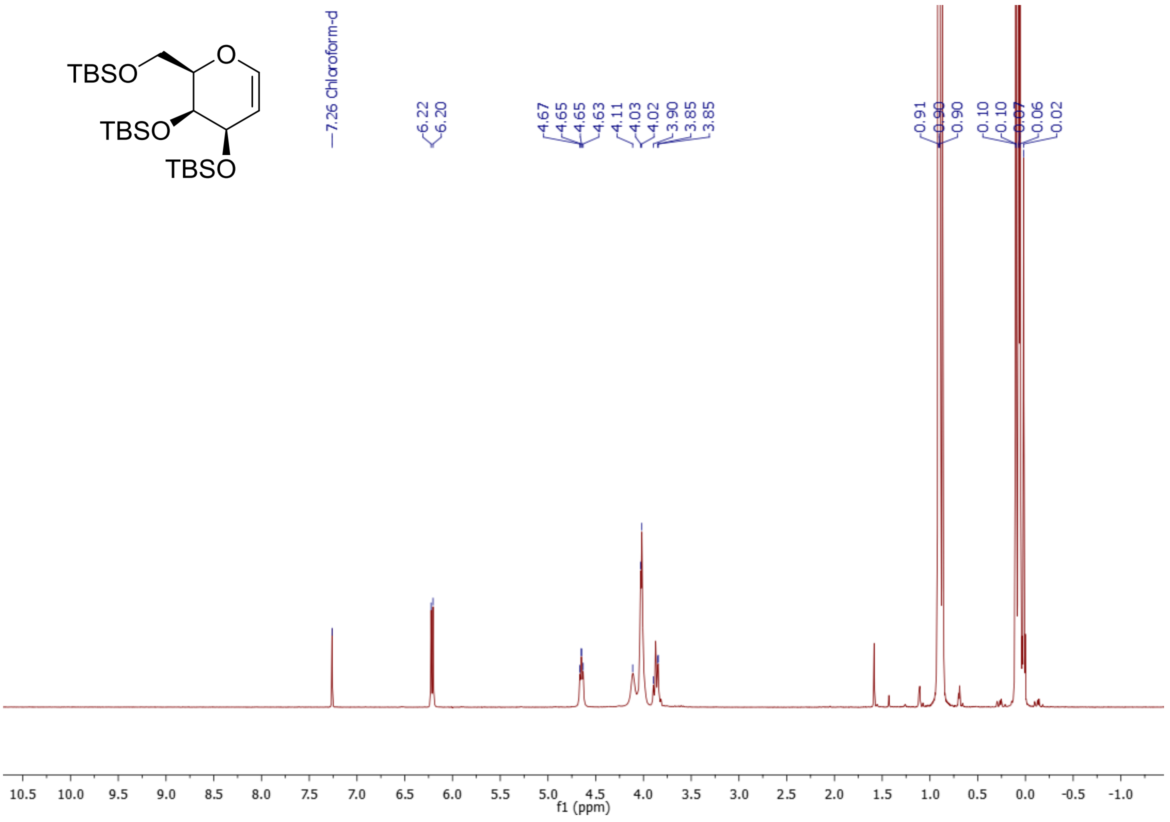

^{13}C NMR (125 MHz, $CDCl_3$), 1,5-Anhydro-2-deoxy-3,4,6-tri-*O*-*tert*-butyldimethylsilyl-D-*lyxo*-hex-1-enitol 3d

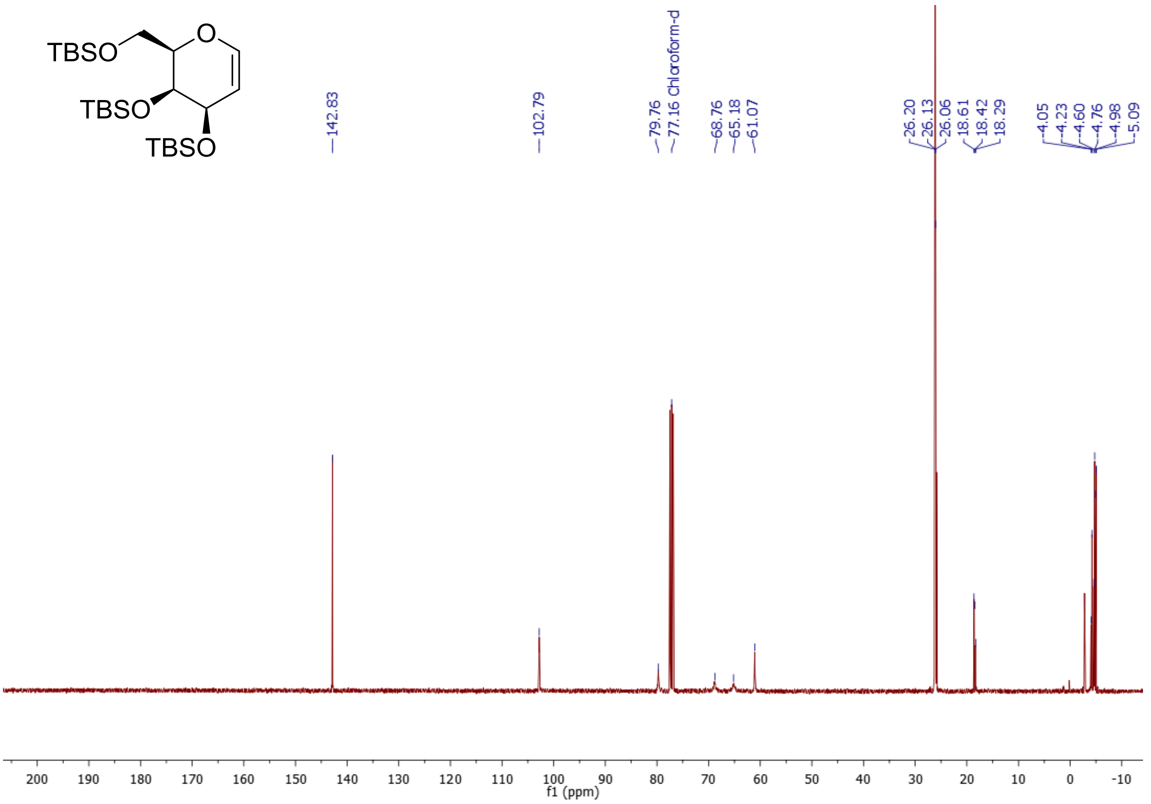

^{1}H NMR (300 MHz, $CDCl_3$), 1,5-Anhydro-2-deoxy-3,4,6-tri-*O*-acetyl-D-*lyxo*-hex-1-enitol 3e

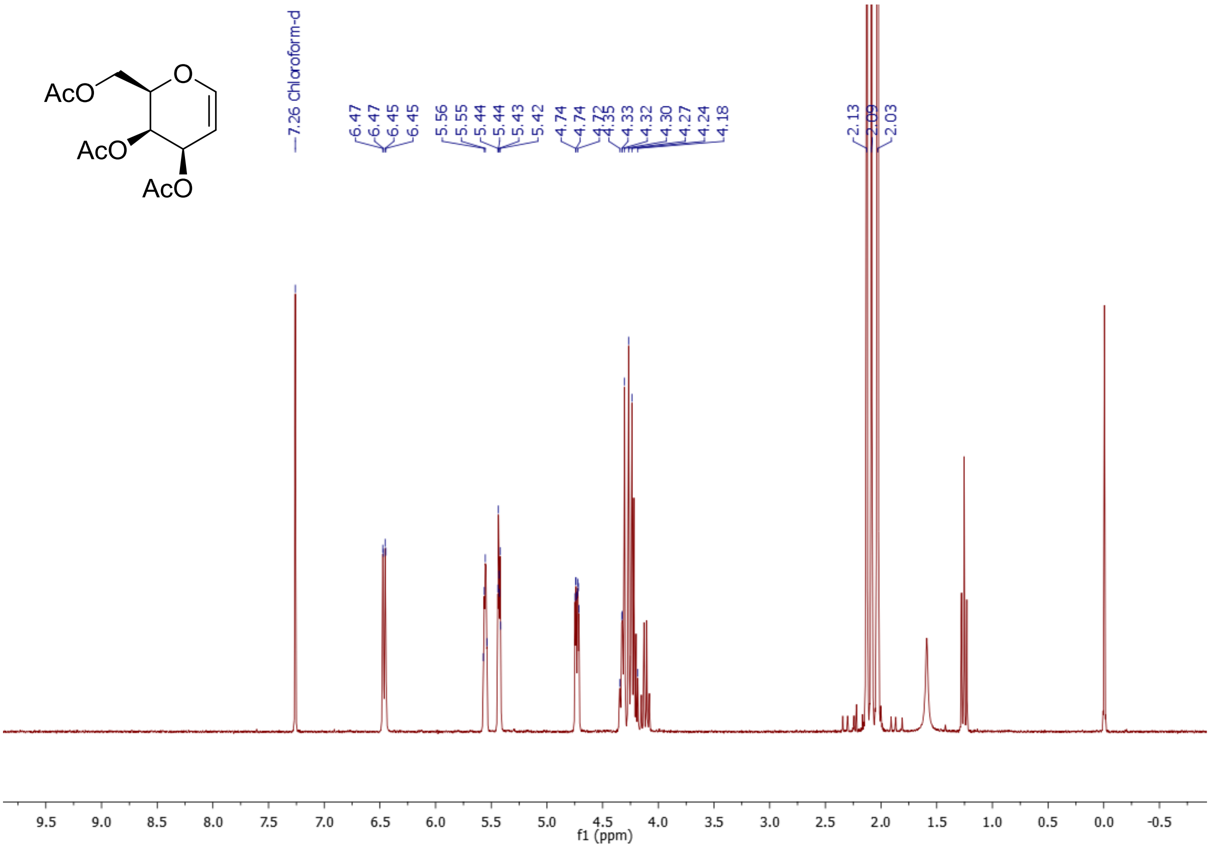

^{13}C NMR (125 MHz, $CDCl_3$), 1,5-Anhydro-2-deoxy-3,4,6-tri-*O*-acetyl-D-*lyxo*-hex-1-enitol 3e

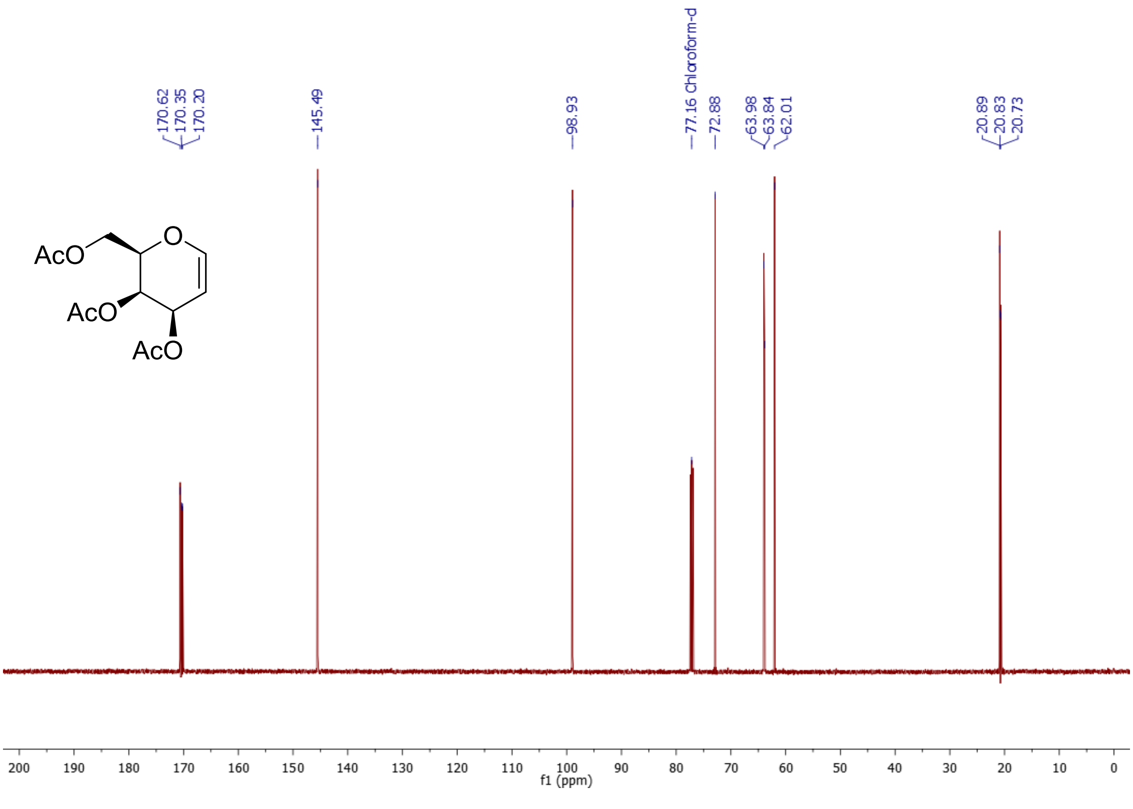

^{1}H NMR (500 MHz, CDCl$_3$), 3,4,6-tri-*O*-benzyl-D-glucal 3f

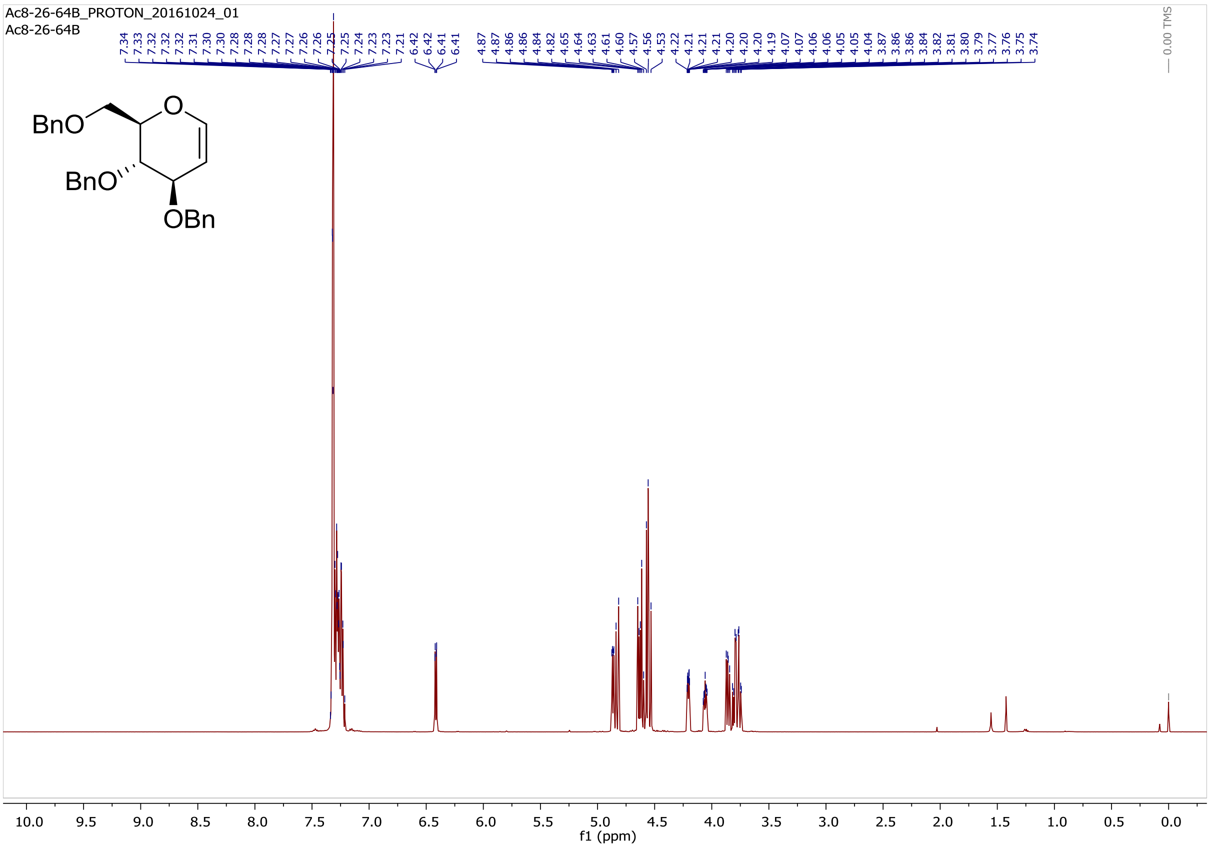

^{13}C NMR (126 MHz, CDCl$_3$), 3,4,6-tri-*O*-benzyl-D-glucal 3f

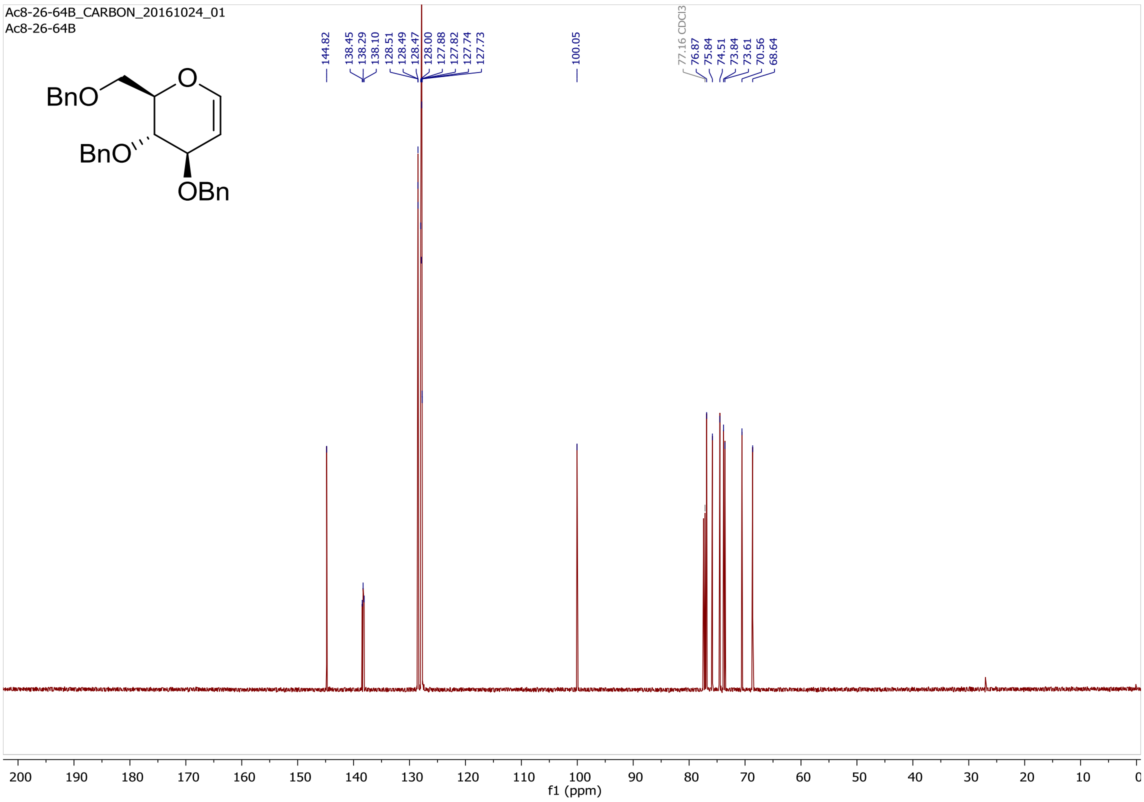

^{1}H NMR (500 MHz, Chloroform-*d*), 3,4-*O*-Dibenzyl-L-rhamnal 3g

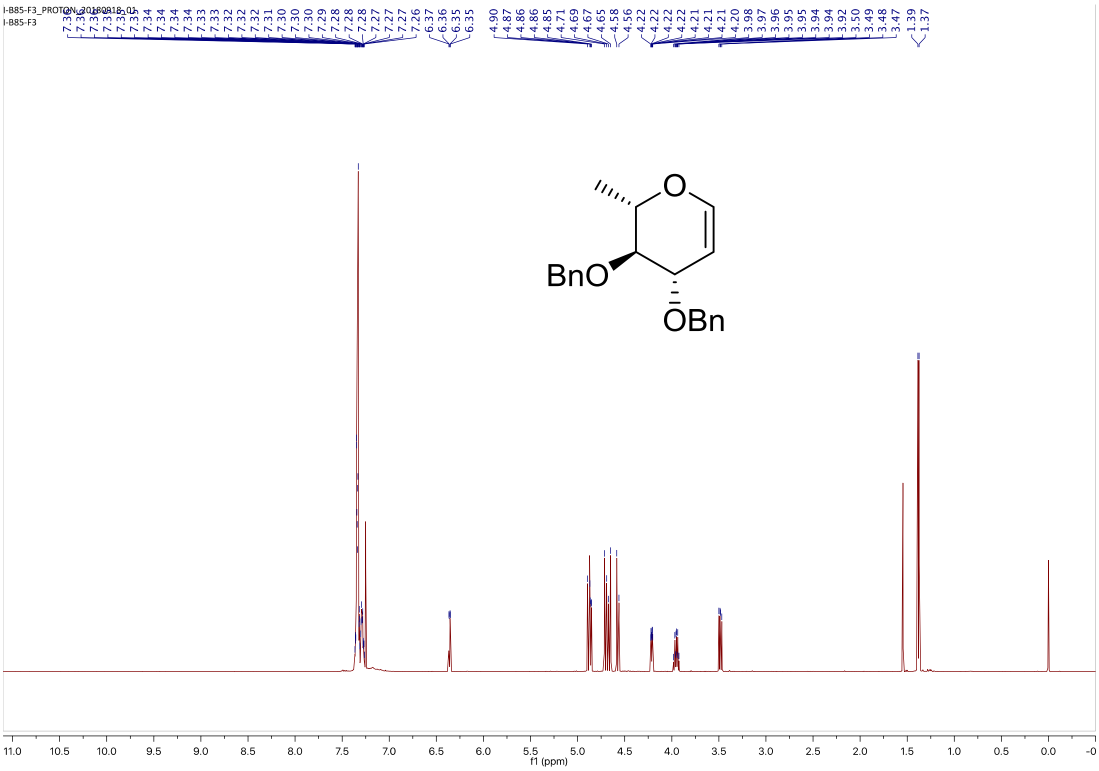

^{13}C NMR (101 MHz, Chloroform-*d*), 3,4-*O*-Dibenzyl-L-rhamnal 3g

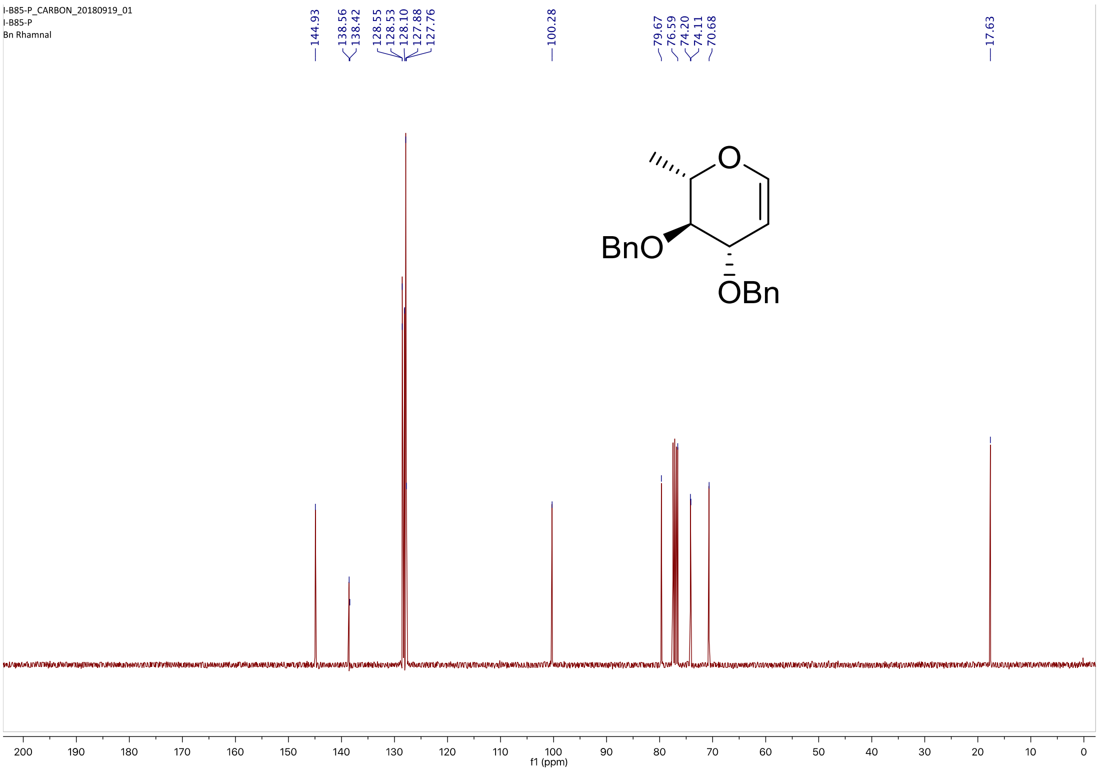

COSY (400 MHz, Chloroform-*d*), 3,4-*O*-Dibenzyl-L-rhamnal 3g

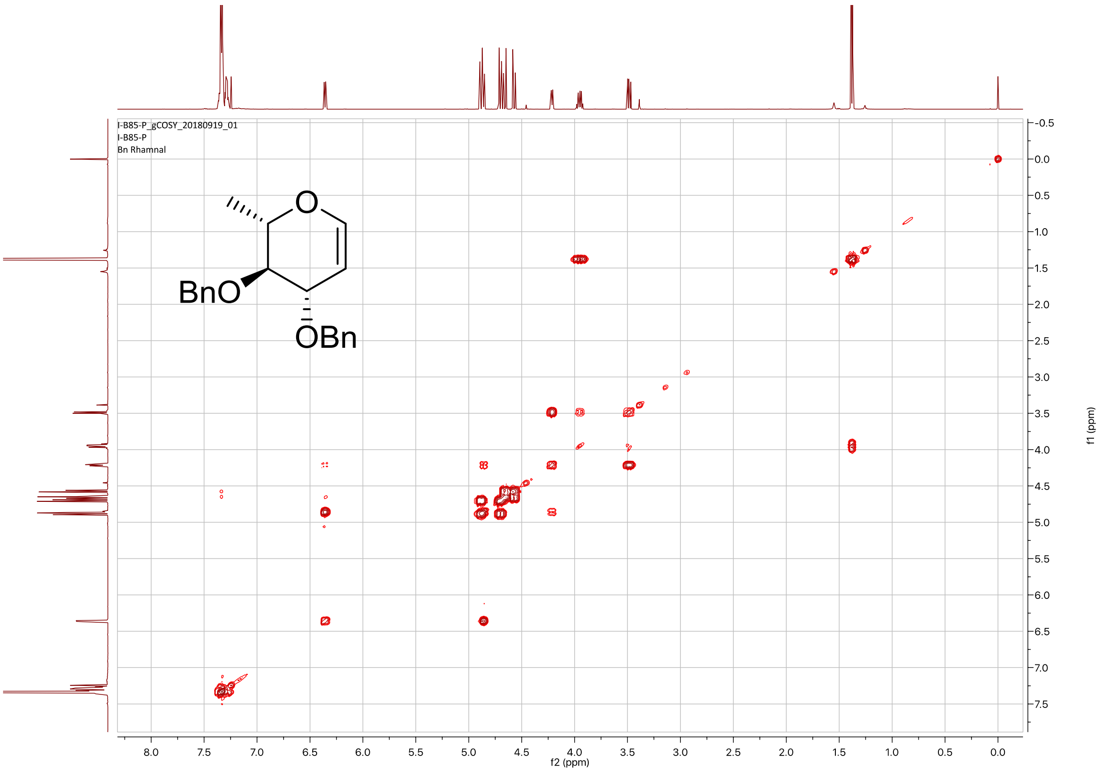

HSQC (500 x 101 MHz, Chloroform-*d*), 3,4-*O*-Dibenzyl-L-rhmanal 3g

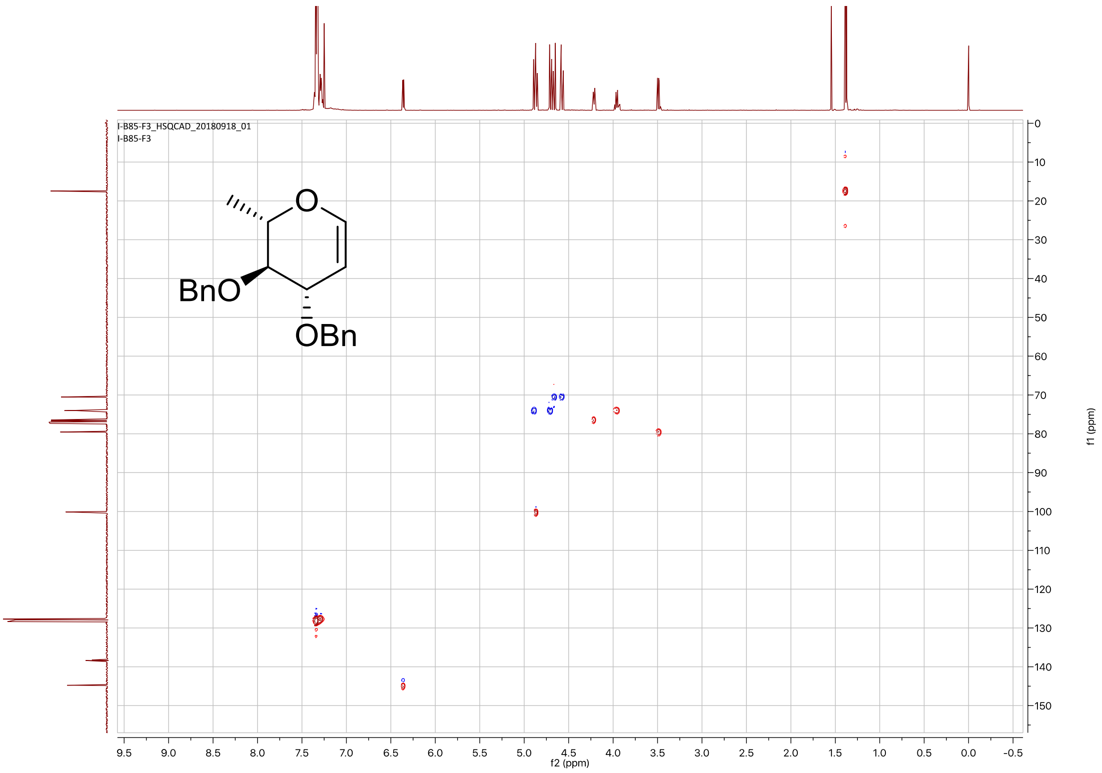

DEPT (101 MHz, Chloroform-*d*), 3,4-*O*-Dibenzyl-L-rhamnal 3g

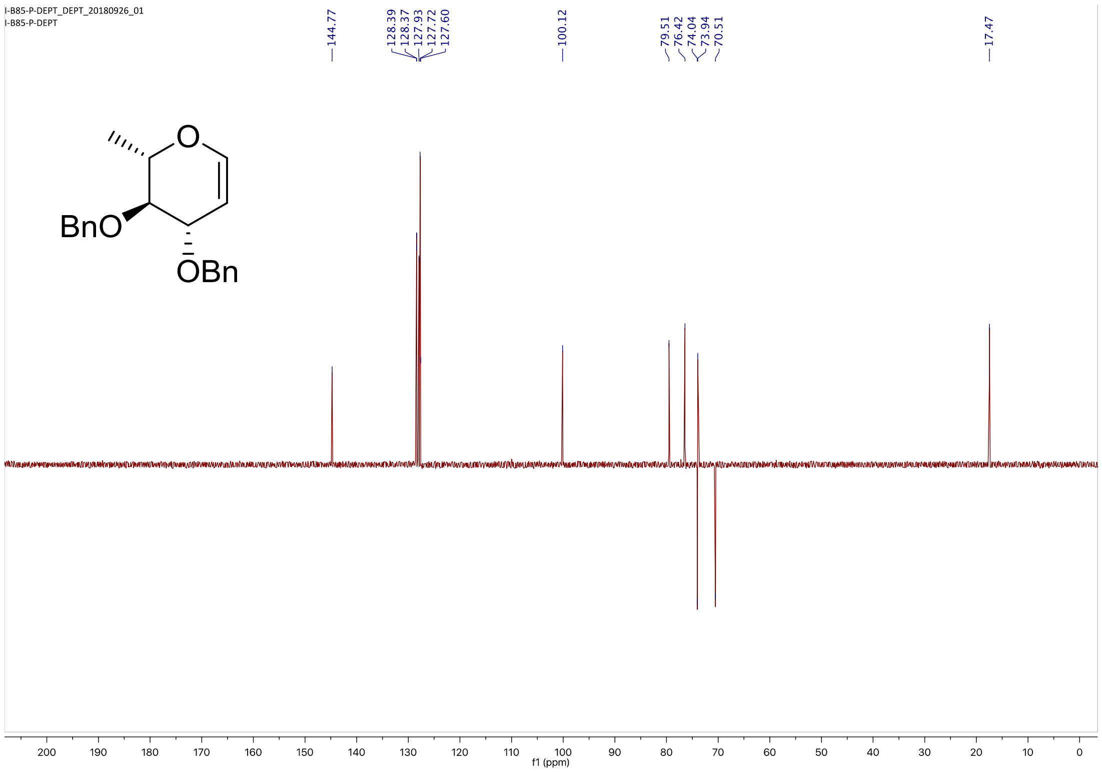

1H NMR (500 MHz, Chloroform-*d*), 3,4-*O*-Dibenzyl-L-fucal 3h

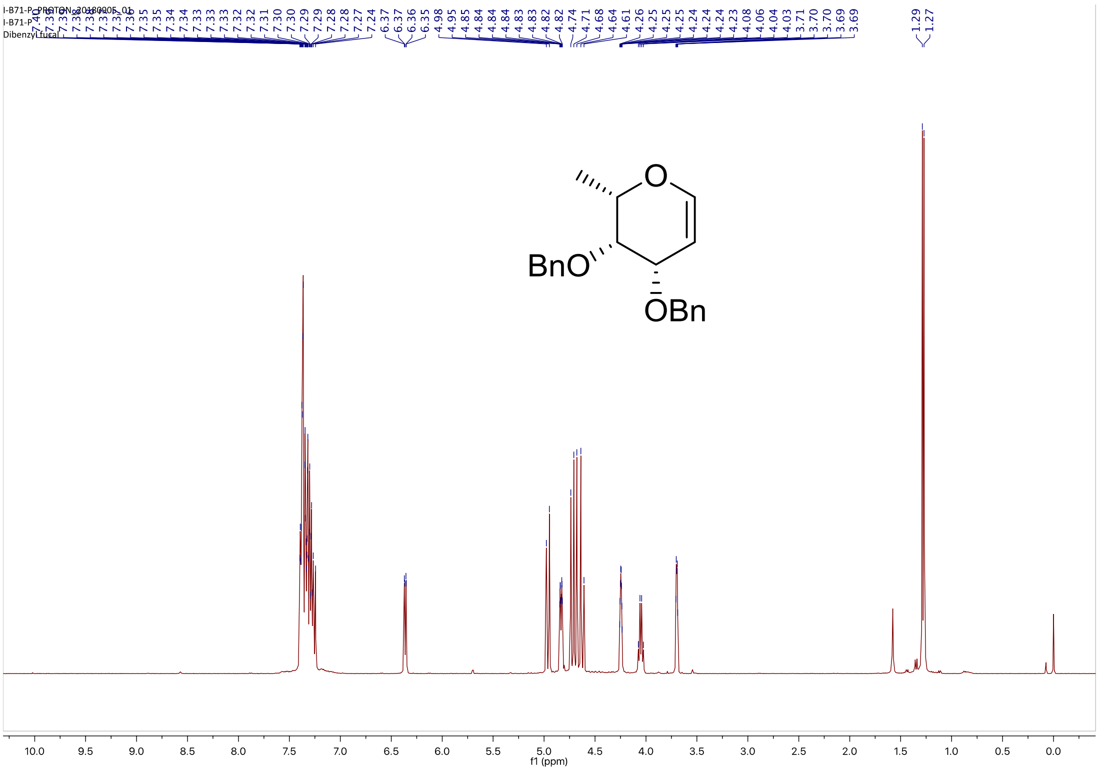

13C NMR (101 MHz, Chloroform-*d*), 3,4-*O*-Dibenzyl-L-fucal 3h

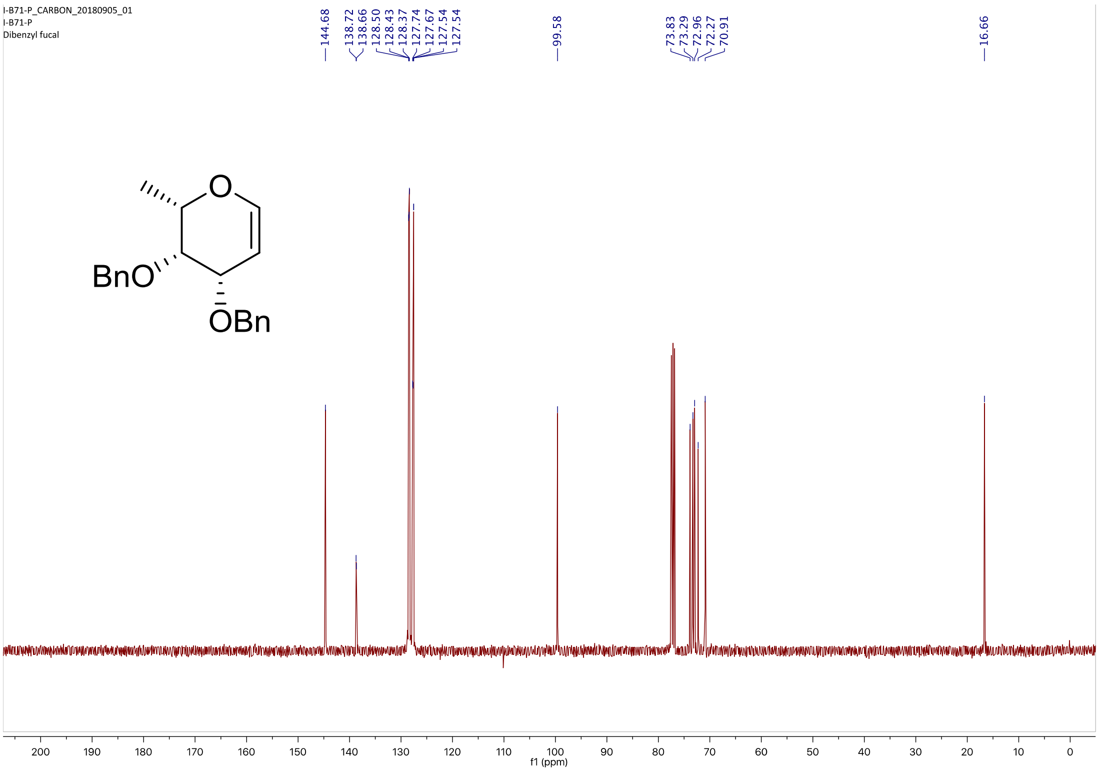

COSY (500 MHz, Chloroform-*d*), 3,4-*O*-Dibenzyl-L-fucal 3h

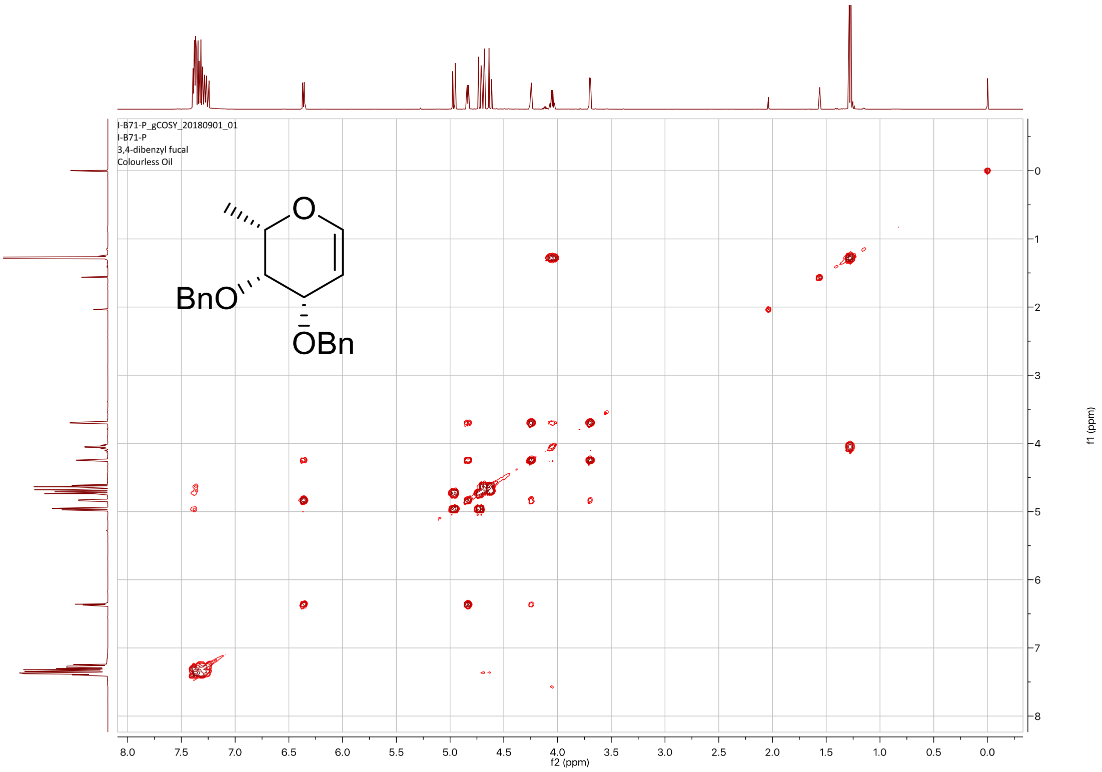

HSQC (500 × 101 MHz, Chloroform-*d*), 3,4-*O*-Dibenzyl-L-fucal 3h

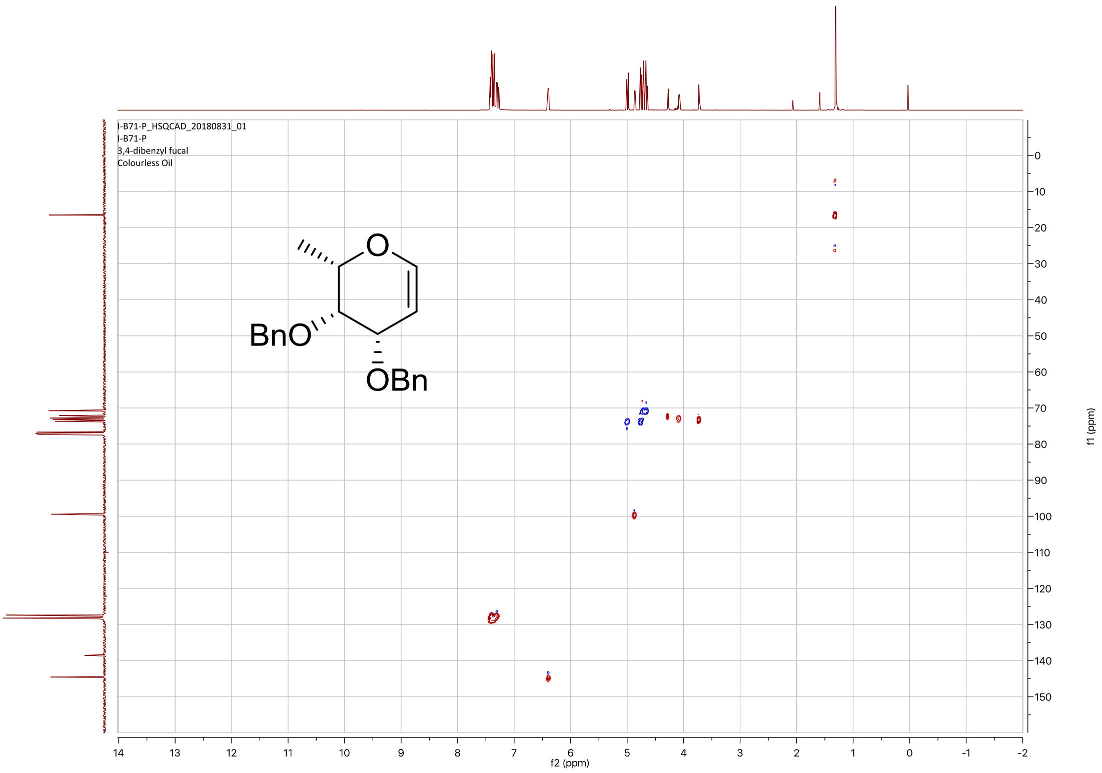

NMR Spectra of Acceptors and their precursors

^{1}H NMR (400 MHz, $CDCl_3$), methyl 4,6-*O*-benzylidene-α-D-glucopyranoside 14

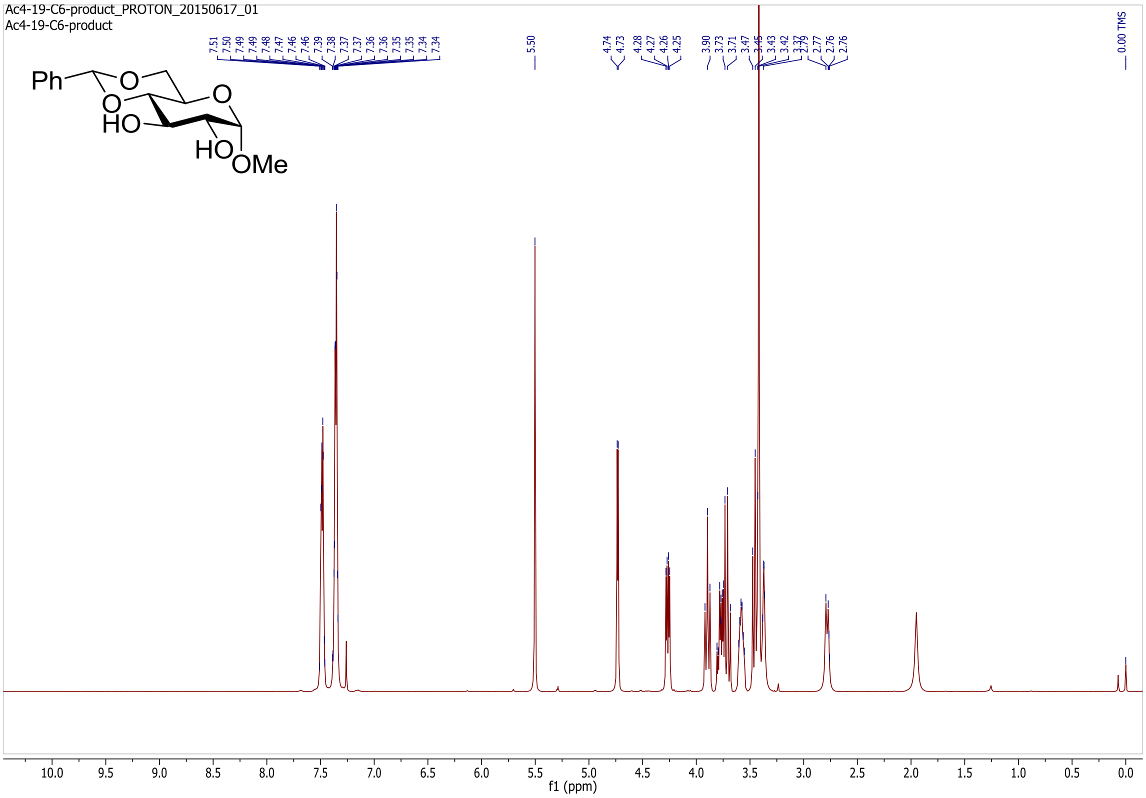

^{13}C NMR (101 MHz, $CDCl_3$), methyl 4,6-*O*-benzylidene-α-D-glucopyranoside 14

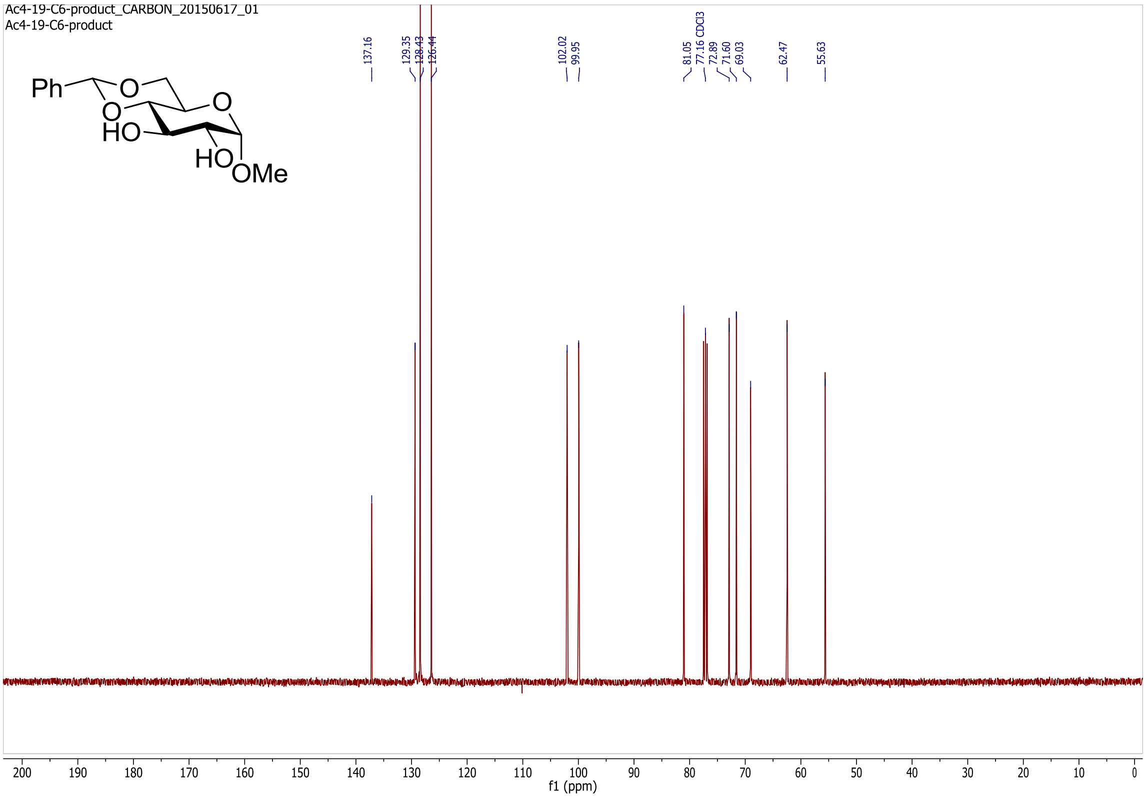

^{1}H NMR (400 MHz, $CDCl_3$), methyl 3-*O*-benzyl-4,6-*O*-benzylidene-α-D-glucopyranoside 6a

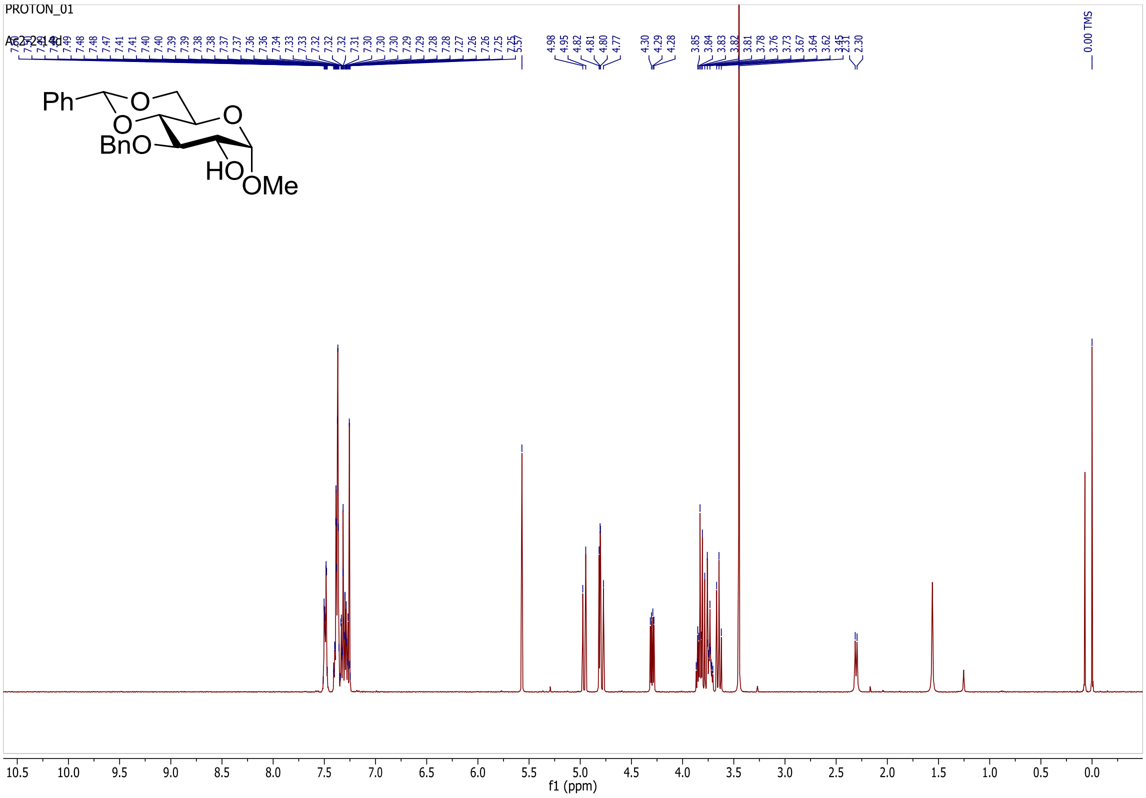

^{13}C NMR (101 MHz, $CDCl_3$), methyl 3-*O*-benzyl-4,6-*O*-benzylidene-α-D-glucopyranoside 6a

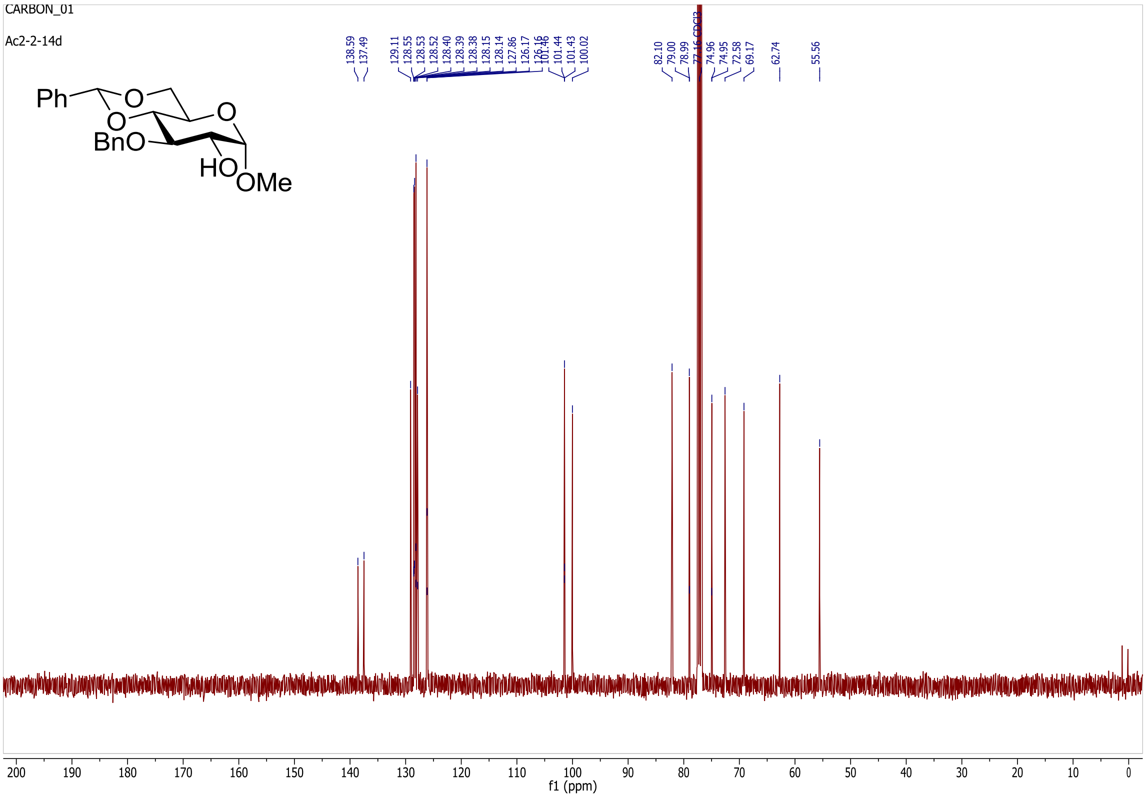

^{1}H NMR (400 MHz, $CDCl_3$), methyl 2-*O*-benzyl-4,6-*O*-benzylidene-α-D-glucopyranoside 6b

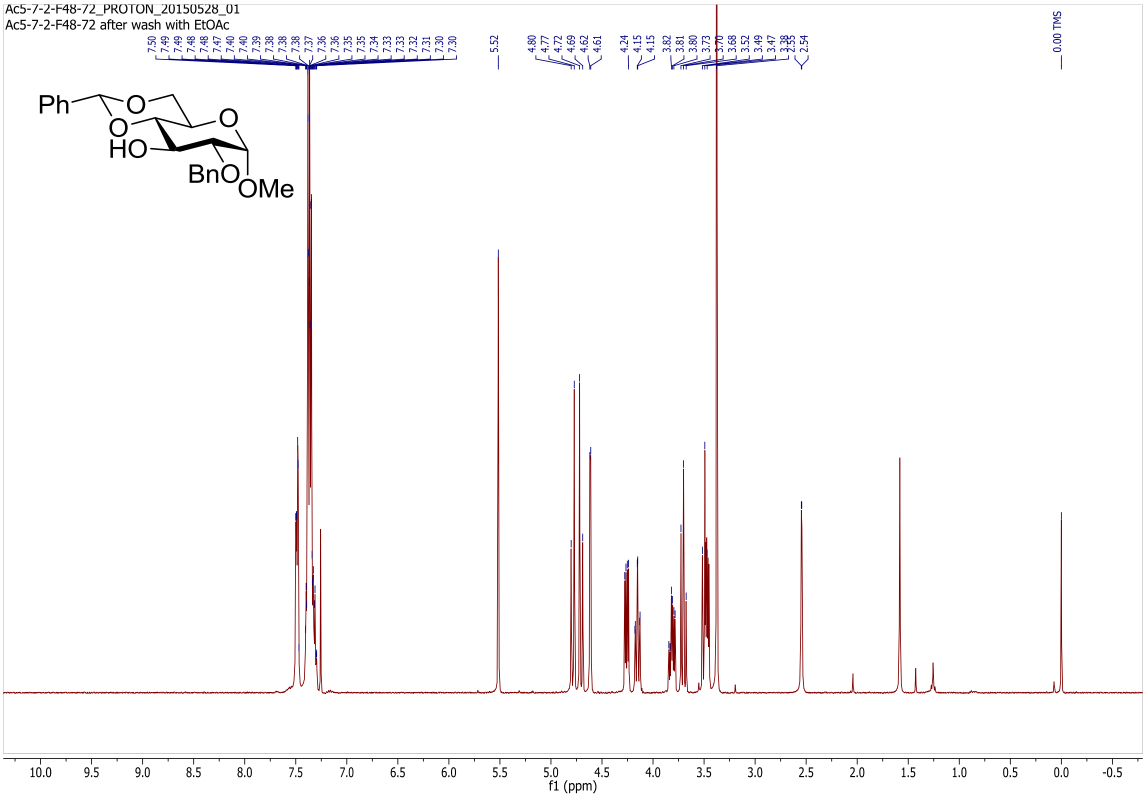

^{13}C NMR (101 MHz, $CDCl_3$), methyl 2-*O*-benzyl-4,6-*O*-benzylidene-α-D-glucopyranoside 6b

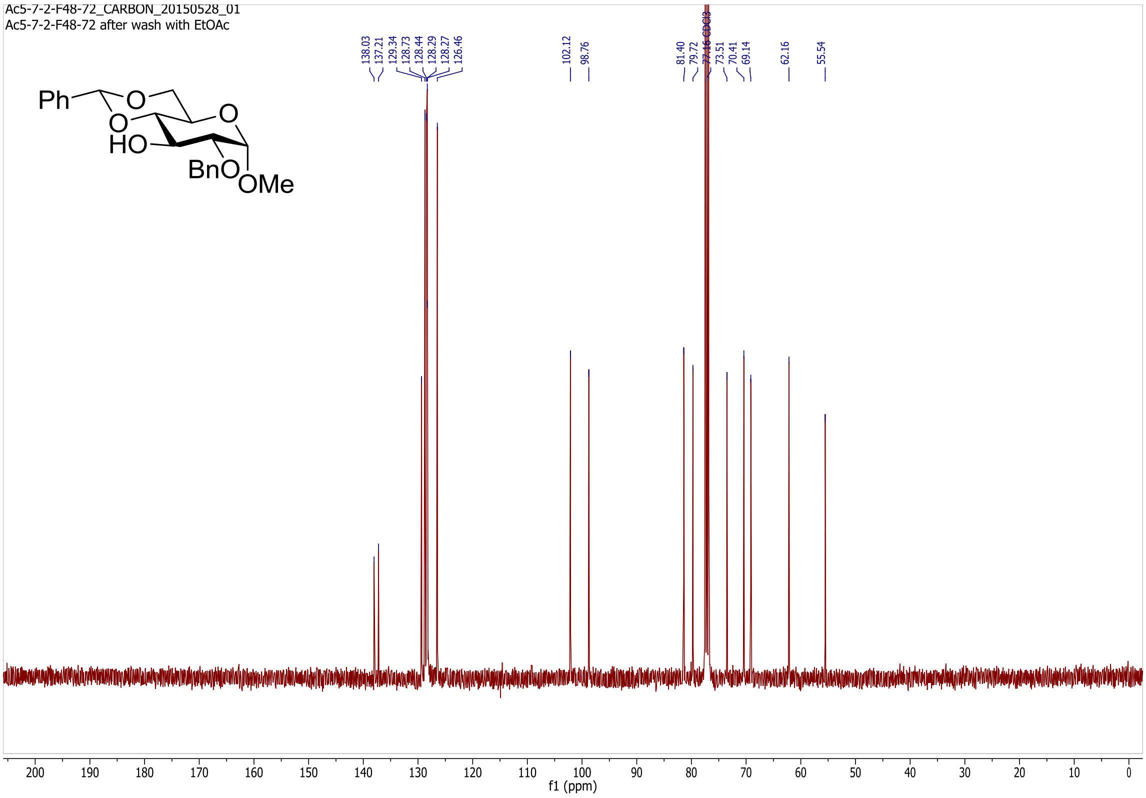

^{1}H NMR (400 MHz, $CDCl_3$), methyl 2,3-*O*-benzyl-4,6-*O*-benzylidene-α-D-glucopyranoside 15

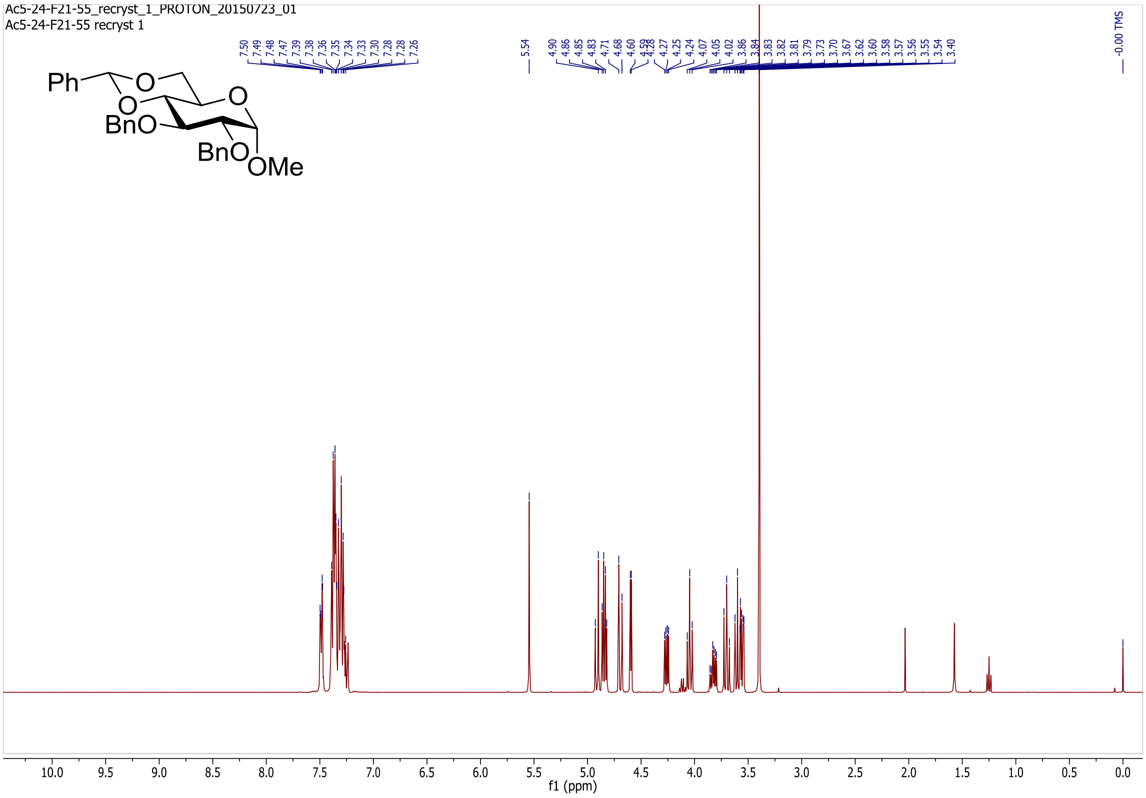

^{13}C NMR (101 MHz, $CDCl_3$), methyl 2,3-*O*-benzyl-4,6-*O*-benzylidene-α-D-glucopyranoside 15

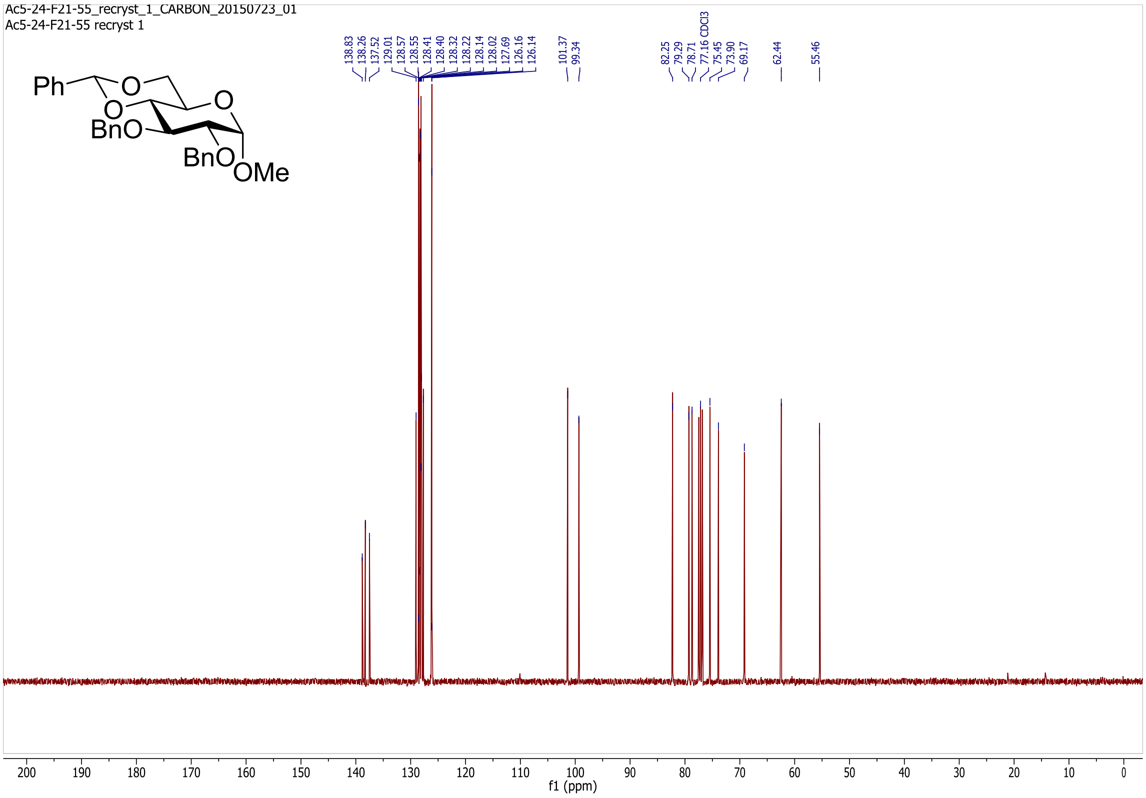

^{1}H NMR (400 MHz, CDCl$_3$), methyl 2,3,6-tri-*O*-benzyl-α-D-glucopyranoside 6c

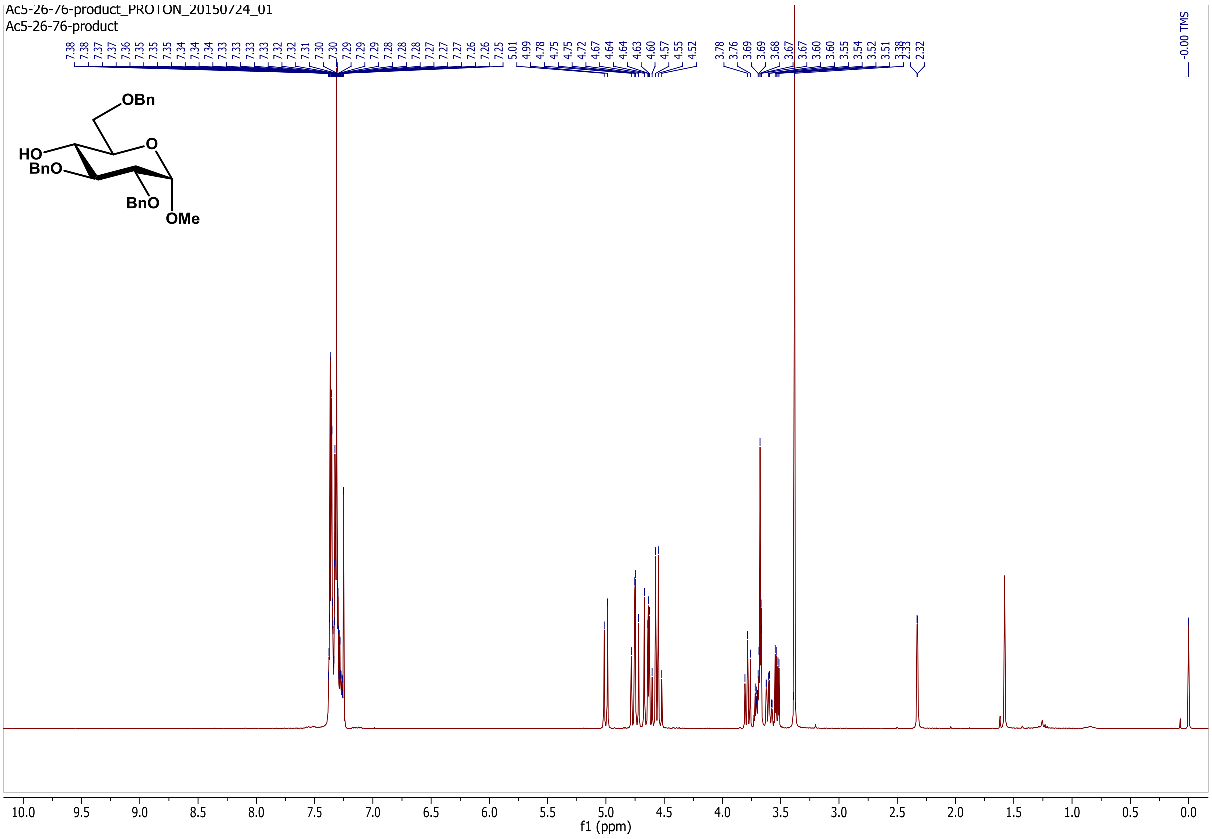

^{13}C NMR (101 MHz, CDCl$_3$), methyl 2,3,6-tri-*O*-benzyl-α-D-glucopyranoside 6c

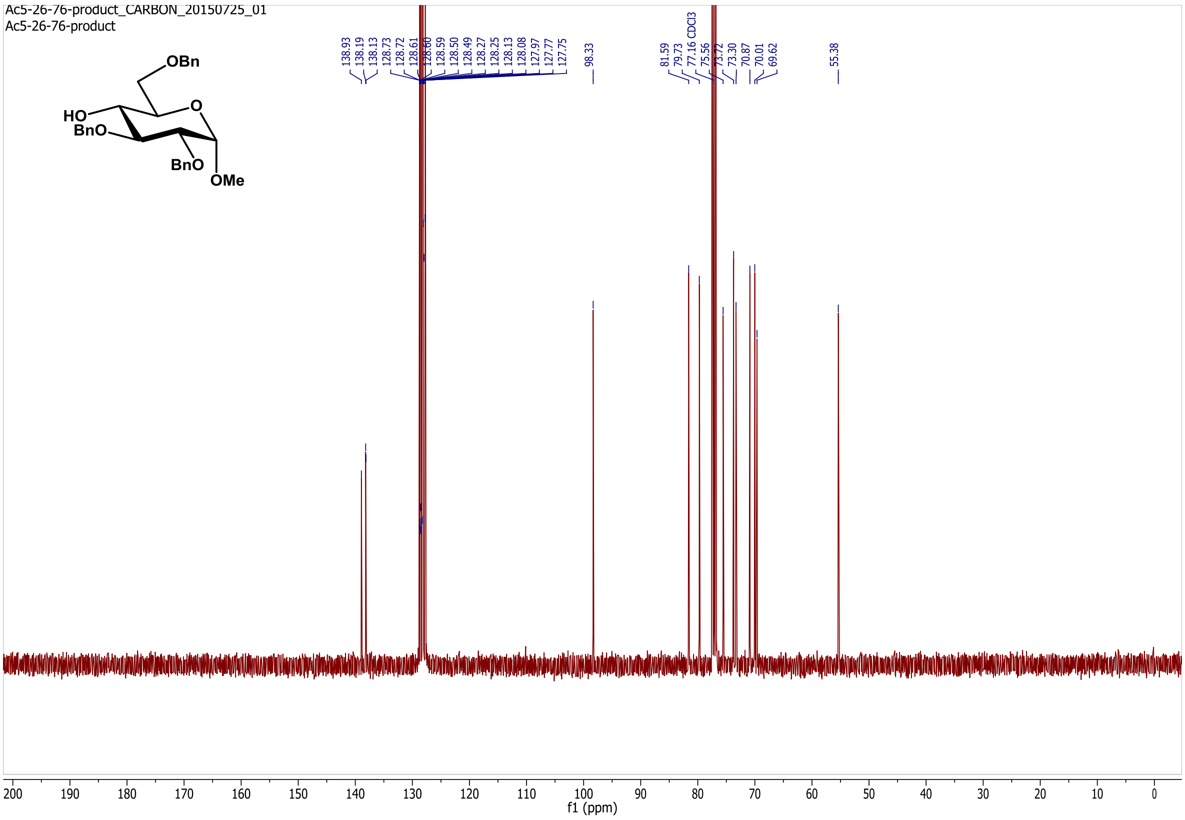

NMR Spectra of Disaccharides

^{1}H NMR (300 MHz, CDCl$_3$), 6-*O*-(3,4,6-tri-*O*-benzyl-2-deoxy-α-D-lyxo-hexapyranosyl)-1,2:3,4-di-*O*-isopropylidene-α-D-galactopyranose 5a

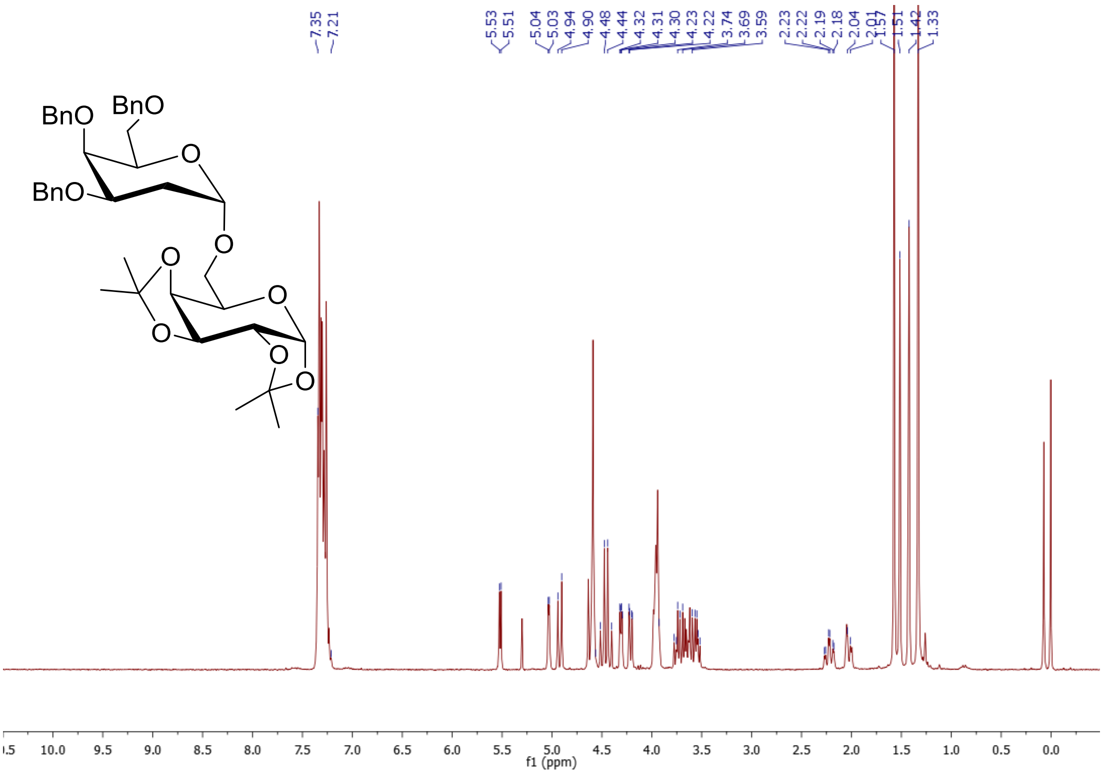

^{13}C NMR (125 MHz, CDCl$_3$), 6-*O*-(3,4,6-tri-*O*-benzyl-2-deoxy-α-D-lyxo-hexapyranosyl)-1,2:3,4-di-*O*-isopropylidene-α-D-galactopyranose 5a

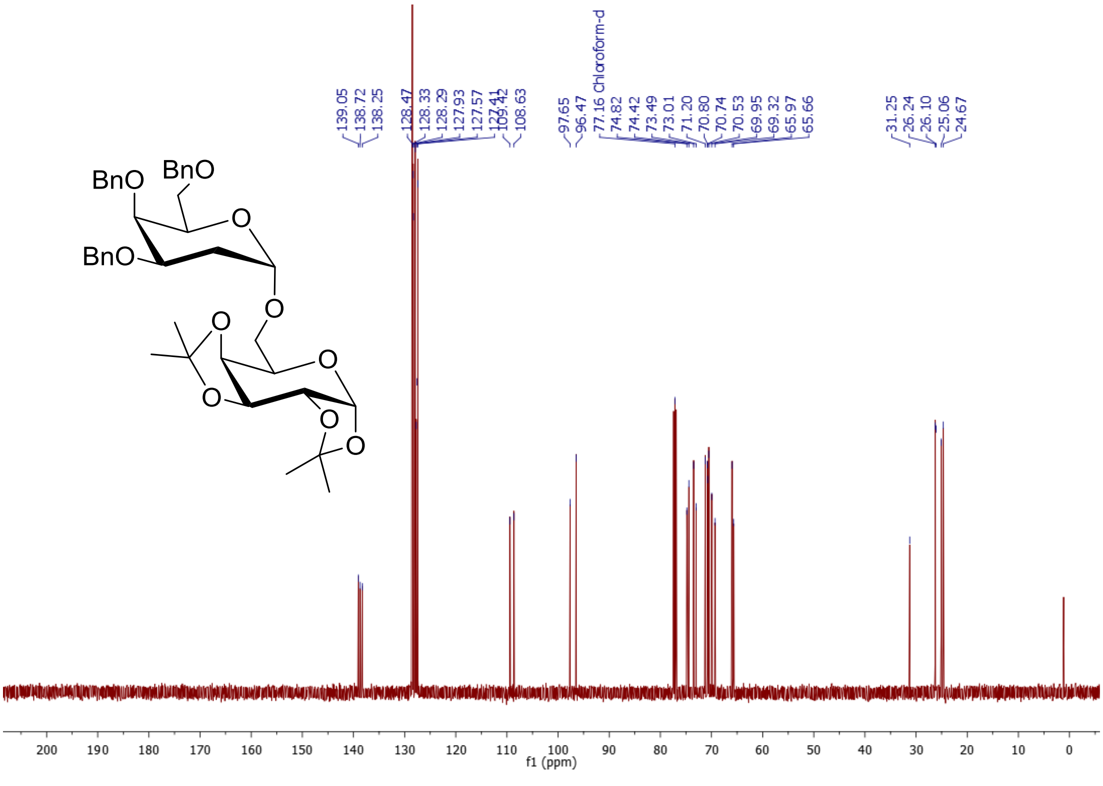

^{1}H NMR (400 MHz, $CDCl_3$), 6-*O*-(3,4,6-tri-*O*-allyl-2-deoxy-α-D-lyxo-hexapyranosyl)-1,2:3,4-di-*O*-isopropylidene-α-D-galactopyranose 5b

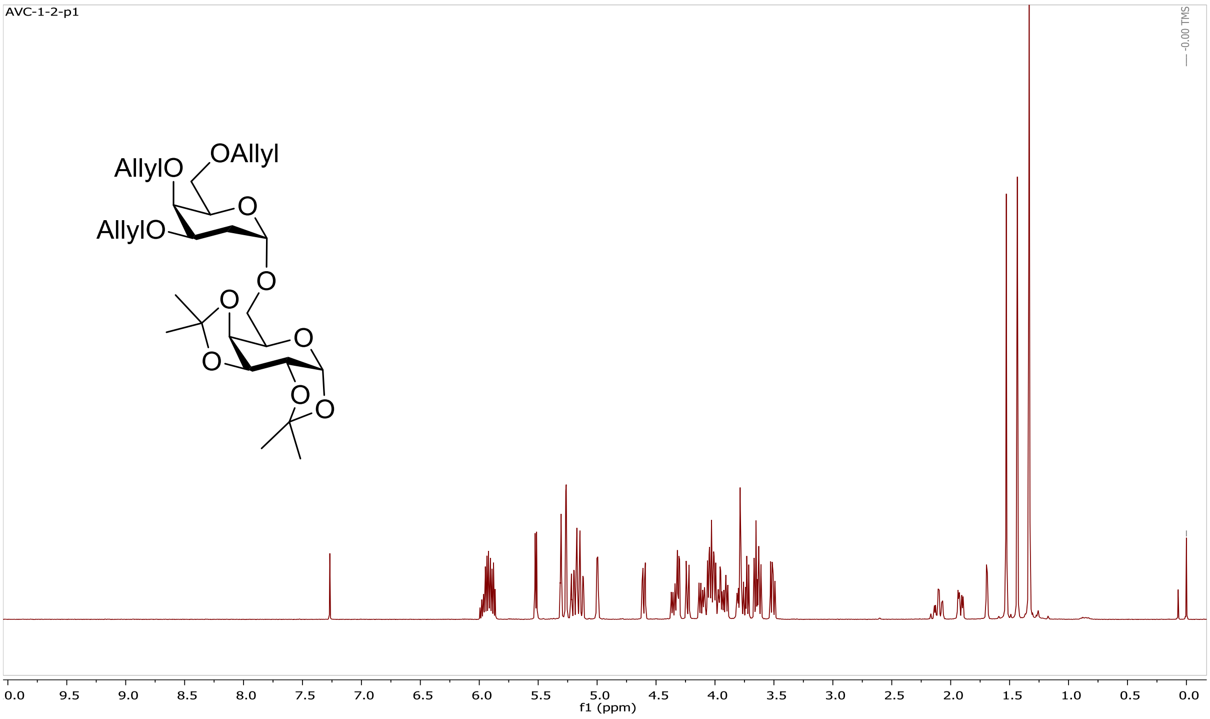

^{13}C NMR (101 MHz, $CDCl_3$), 6-*O*-(3,4,6-tri-*O*-allyl-2-deoxy-α-D-lyxo-hexapyranosyl)-1,2:3,4-di-*O*-isopropylidene-α-D-galactopyranose 5b

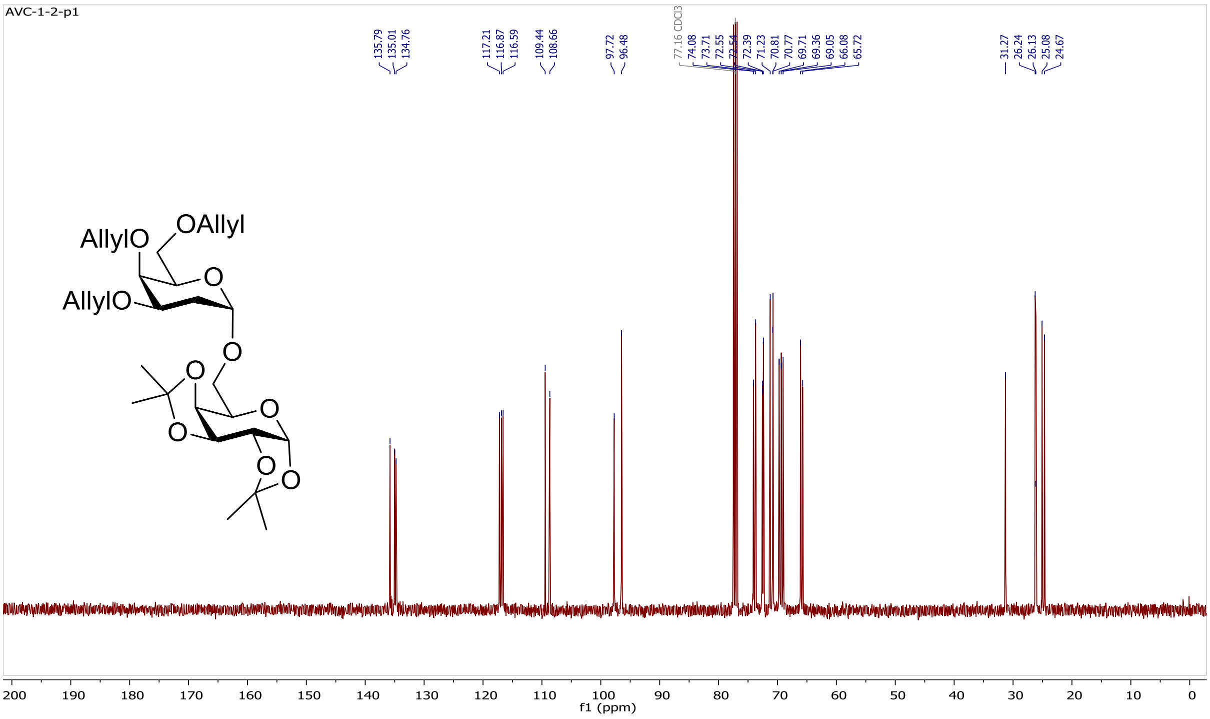

^{1}H NMR (500 MHz, $CDCl_3$), 6-*O*-(6-*O*-acetyl-3,4-di-*O*-benzyl-2-deoxy-α-D-lyxo-hexapyranosyl)-1,2:3,4-di-*O*-isopropylidene-α-D-galactopyranose 5c

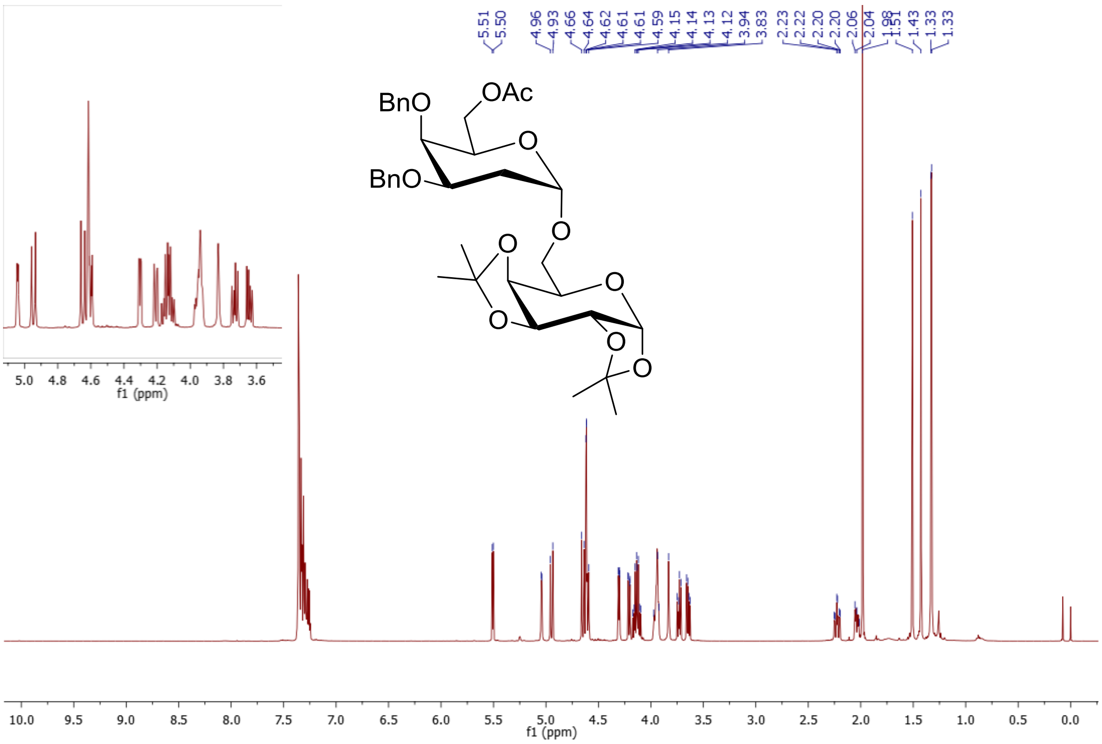

^{13}C NMR (125 MHz, $CDCl_3$), 6-*O*-(6-*O*-acetyl-3,4-di-*O*-benzyl-2-deoxy-α-D-lyxo-hexapyranosyl)-1,2:3,4-di-*O*-isopropylidene-α-D-galactopyranose 5c

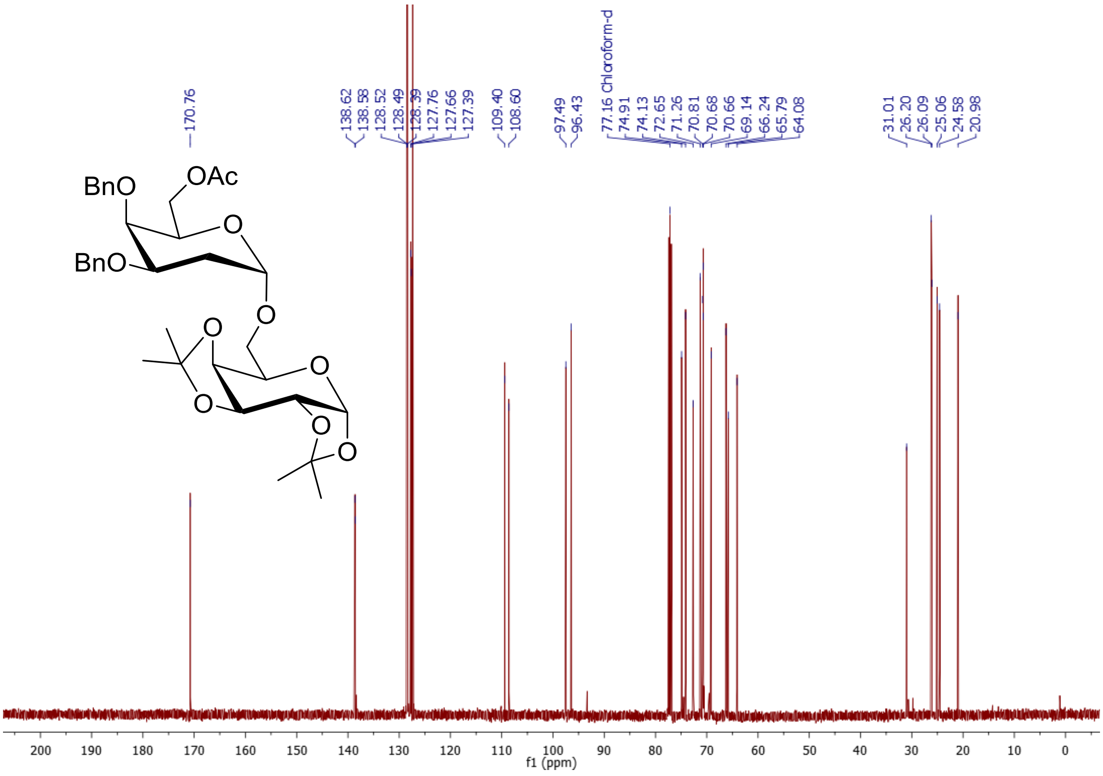

^{1}H NMR (300 MHz, CDCl$_3$), 6-*O*-(3,4,6-tri-*O*-*tert*-butyldimethylsilyl-2-deoxy-α-D-lyxo-hexapyranosyl)-1,2:3,4-di-*O*-isopropylidene-α-D-galactopyranose 5d

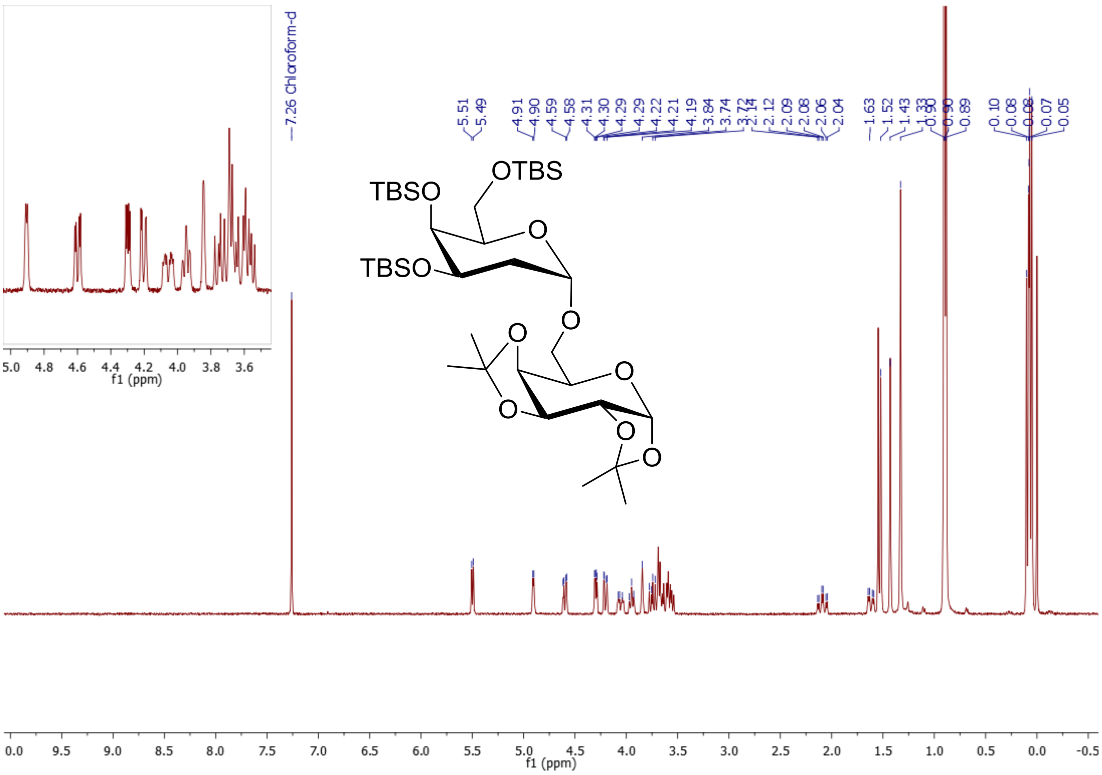

^{13}C NMR (125 MHz, CDCl$_3$), 6-*O*-(3,4,6-tri-*O*-*tert*-butyldimethylsilyl-2-deoxy-α-D-lyxo-hexapyranosyl)-1,2:3,4-di-*O*-isopropylidene-α-D-galactopyranose 5d

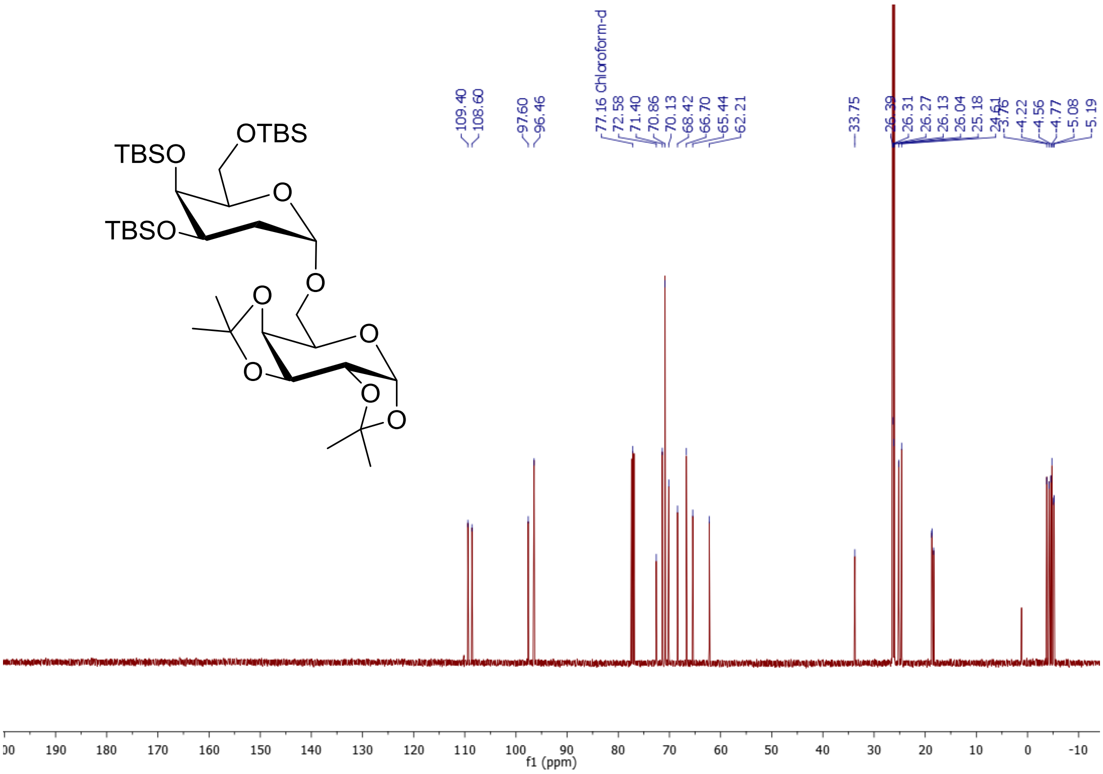

^{1}H NMR (500 MHz, $CDCl_3$), 6-*O*-(3,4,6-tri-*O*-benzyl-2-deoxy-α/β-D-*erythro*-hexapyranosyl)-1,2:3,4-di-*O*-isopropylidene-α-D-galactopyranose 5fa and 6-*O*-(4,6-di-*O*-benzyl-2,3-dideoxy-α/β-D-*erythro*-hex-2-enopyranosyl)-1,2:3,4-di-*O*-isopropylidene-α-D-galactopyranose 5fb

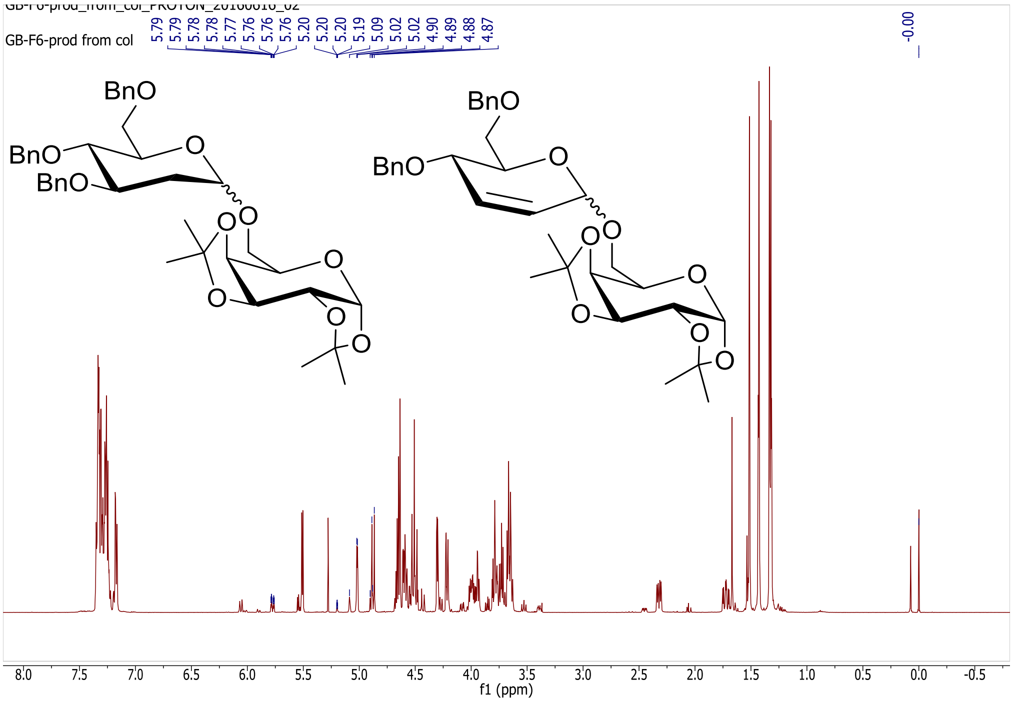

^{13}C NMR (126 MHz, $CDCl_3$), 6-*O*-(3,4,6-tri-*O*-benzyl-2-deoxy-α/β-D-*erythro*-hexapyranosyl)-1,2:3,4-di-*O*-isopropylidene-α-D-galactopyranose 5fa and 6-*O*-(4,6-di-*O*-benzyl-2,3-dideoxy-α/β-D-*erythro*-hex-2-enopyranosyl)-1,2:3,4-di-*O*-isopropylidene-α-D-galactopyranose 5fb

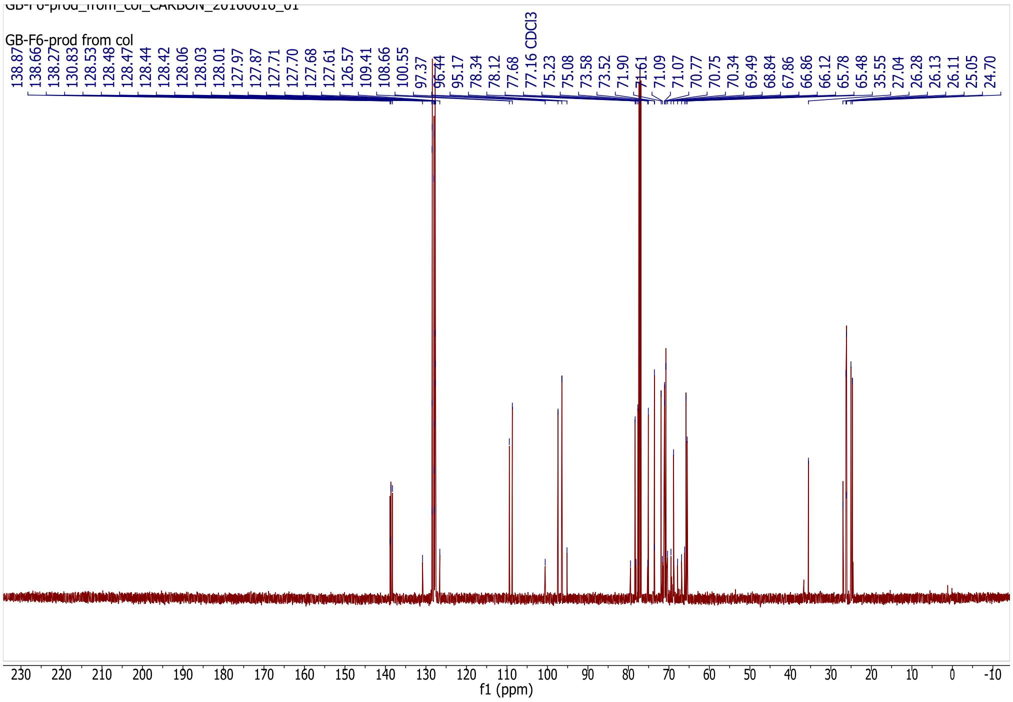

HSQC NMR (500 ×126 MHz, $CDCl_3$), 6-*O*-(3,4,6-tri-*O*-benzyl-2-deoxy-α/β-D-*erythro*-hexapyranosyl)-1,2:3,4-di-*O*-isopropylidene-α-D-galactopyranose 5fa and 6-*O*-(4,6-di-*O*-benzyl-2,3-dideoxy-α/β-D-*erythro*-hex-2-enopyranosyl)-1,2:3,4-di-*O*-isopropylidene-α-D-galactopyranose 5fb

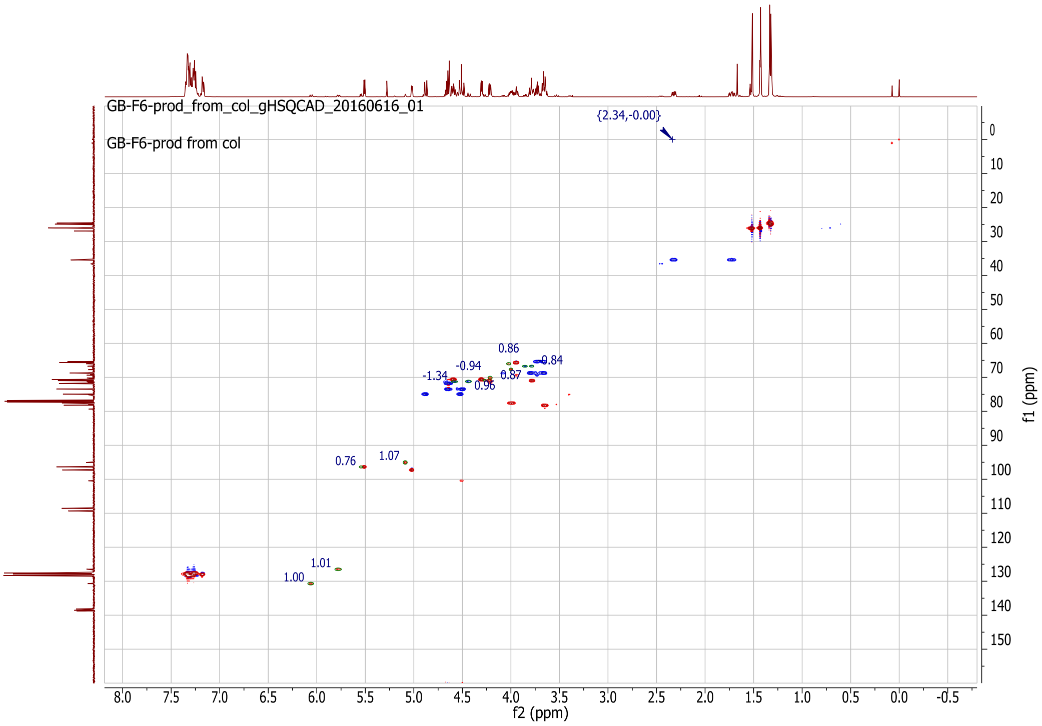

HMBC NMR (500 ×126 MHz, $CDCl_3$), 6-*O*-(3,4,6-tri-*O*-benzyl-2-deoxy-α/β-D-*erythro*-hexapyranosyl)-1,2:3,4-di-*O*-isopropylidene-α-D-galactopyranose 5fa and 6-*O*-(4,6-di-*O*-benzyl-2,3-dideoxy-α/β-D-*erythro*-hex-2-enopyranosyl)-1,2:3,4-di-*O*-isopropylidene-α-D-galactopyranose 5fb

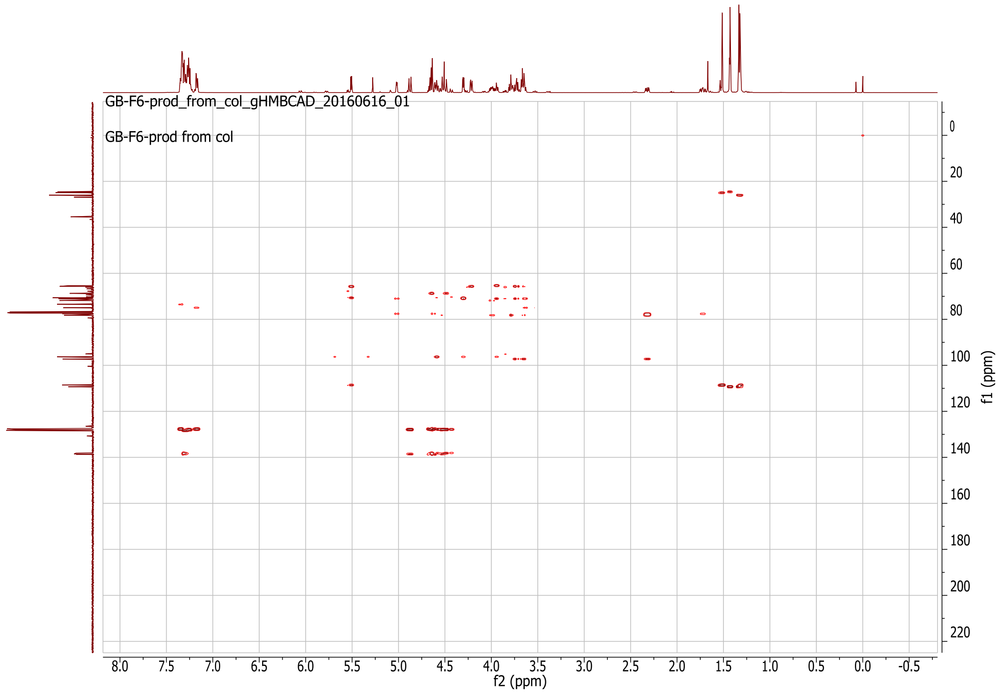

^{1}H NMR (600 MHz, Chloroform-*d*), (3,4-Di-*O*-benzyl-2,6-dideoxy-α/β-L-*erythro*-hexapyranosyl)-(1→6)-1,2:3,4-di-*O*-isopropylidene-α-D-galactopyranoside 5ga and 4-*O*-(benzyl)-2,3,6-trideoxy-α/β-L-hex-2-enopyranosyl-(1→6)-1,2;3,4-di-*O*-isopropylidene-α-D-galactopyranoside 5gb

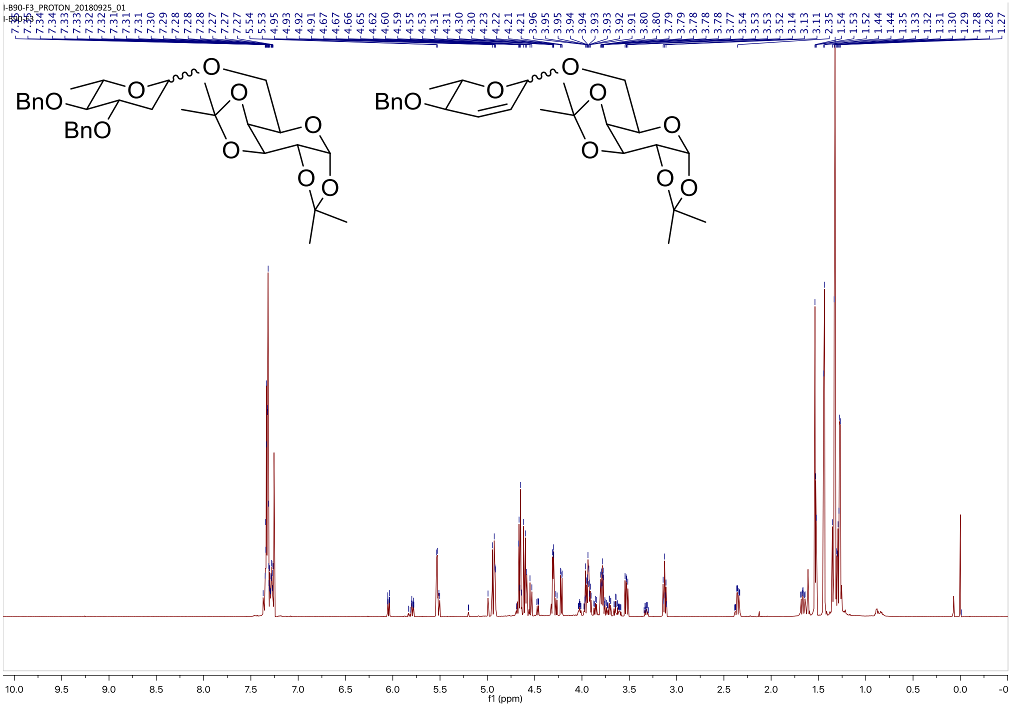

^{13}C NMR (151 MHz, Chloroform-*d*), (3,4-Di-*O*-benzyl-2,6-dideoxy-α/β-L-*erythro*-hexapyranosyl)-(1→6)-1,2:3,4-di-*O*-isopropylidene-α-D-galactopyranoside 5ga and 4-*O*-(benzyl)-2,3,6-trideoxy-α/β-L-hex-2-enopyranosyl-(1→6)-1,2;3,4-di-*O*-isopropylidene-α-D-galactopyranoside 5gb

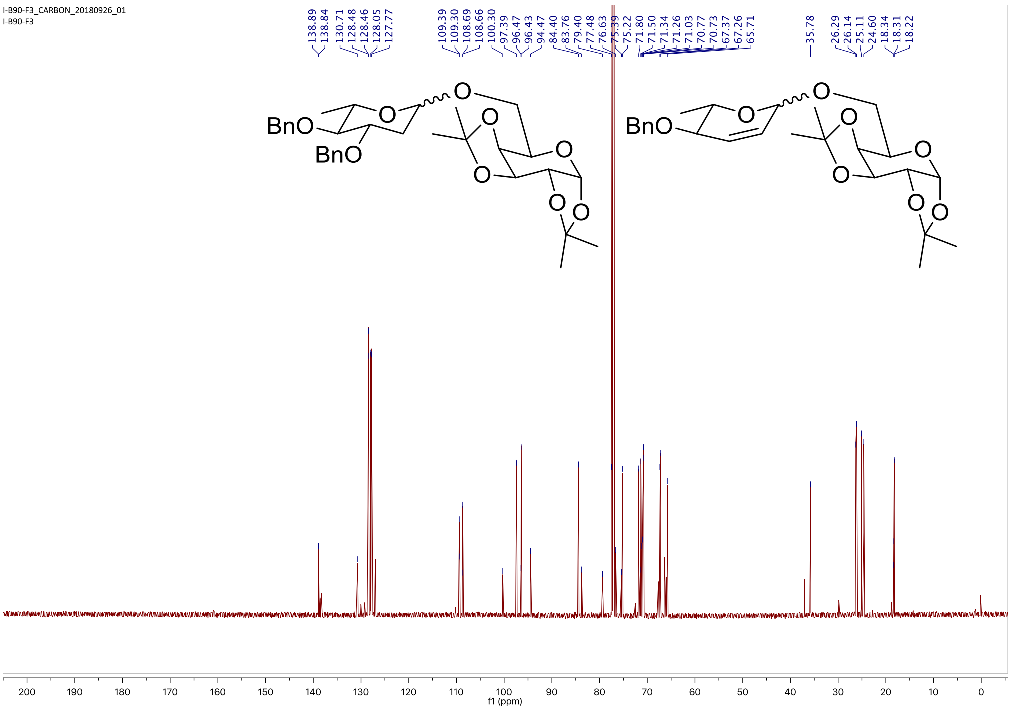

COSY (600 MHz, Chloroform-*d*), (3,4-Di-*O*-benzyl-2,6-dideoxy-α/β-L-*erythro*-hexapyranosyl)-(1→6)-1,2:3,4-di-*O*-isopropylidene-α-D-galactopyranoside 5ga and 4-*O*-(benzyl)-2,3,6-trideoxy-α/β-L-hex-2-enopyranosyl-(1→6)-1,2;3,4-di-*O*-isopropylidene-α-D-galactopyranoside 5gb

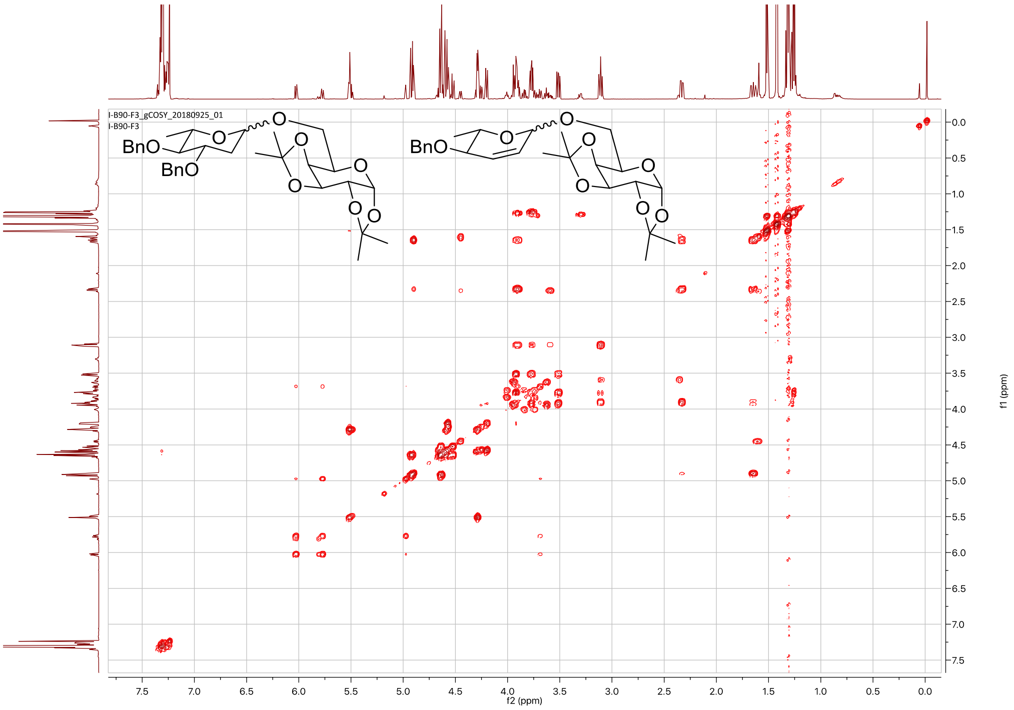

HSQC (600 x 151 MHz, Chloroform-*d*), (3,4-Di-*O*-benzyl-2,6-dideoxy-α/β-L-*erythro*-hexapyranosyl)-(1→6)-1,2:3,4-di-*O*-isopropylidene-α-D-galactopyranoside 5ga and 4-*O*-(benzyl)-2,3,6-trideoxy-α/β-L-hex-2-enopyranosyl-(1→6)-1,2;3,4-di-*O*-isopropylidene-α-D-galactopyranoside 5gb

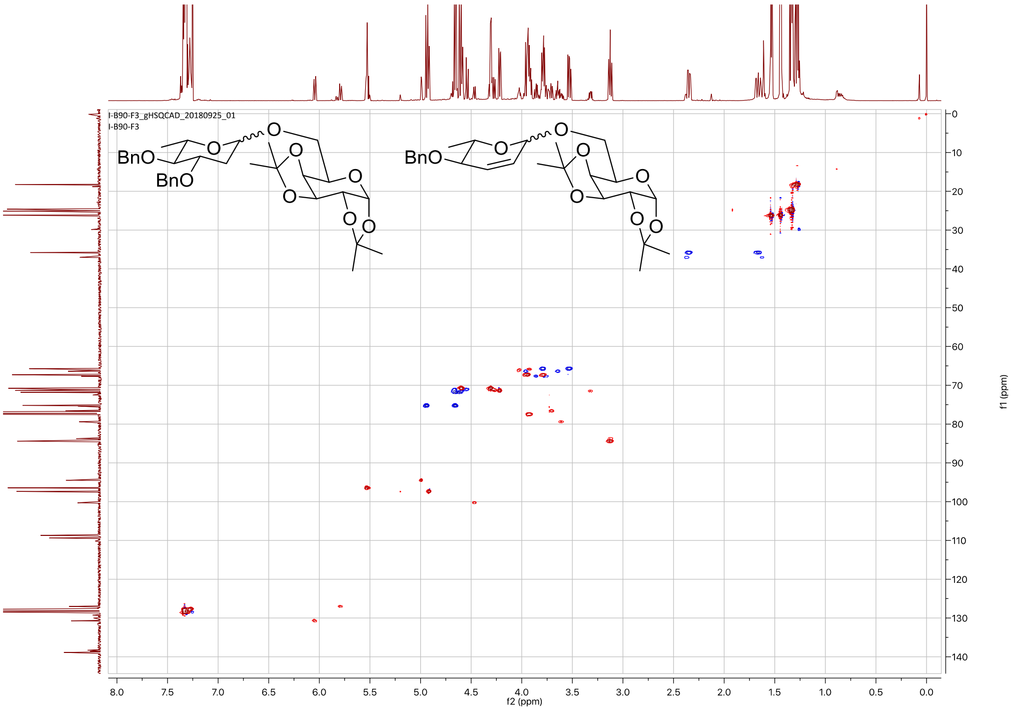

HMBC (600 × 151 MHz, Chloroform-*d*), (3,4-Di-*O*-benzyl-2,6-dideoxy-α/β-L-*erythro*-hexapyranosyl)-(1→6)-1,2:3,4-di-*O*-isopropylidene-α-D-galactopyranoside 5ga and 4-*O*-(benzyl)-2,3,6-trideoxy-α/β-L-hex-2-enopyranosyl-(1→6)-1,2;3,4-di-*O*-isopropylidene-α-D-galactopyranoside 5gb

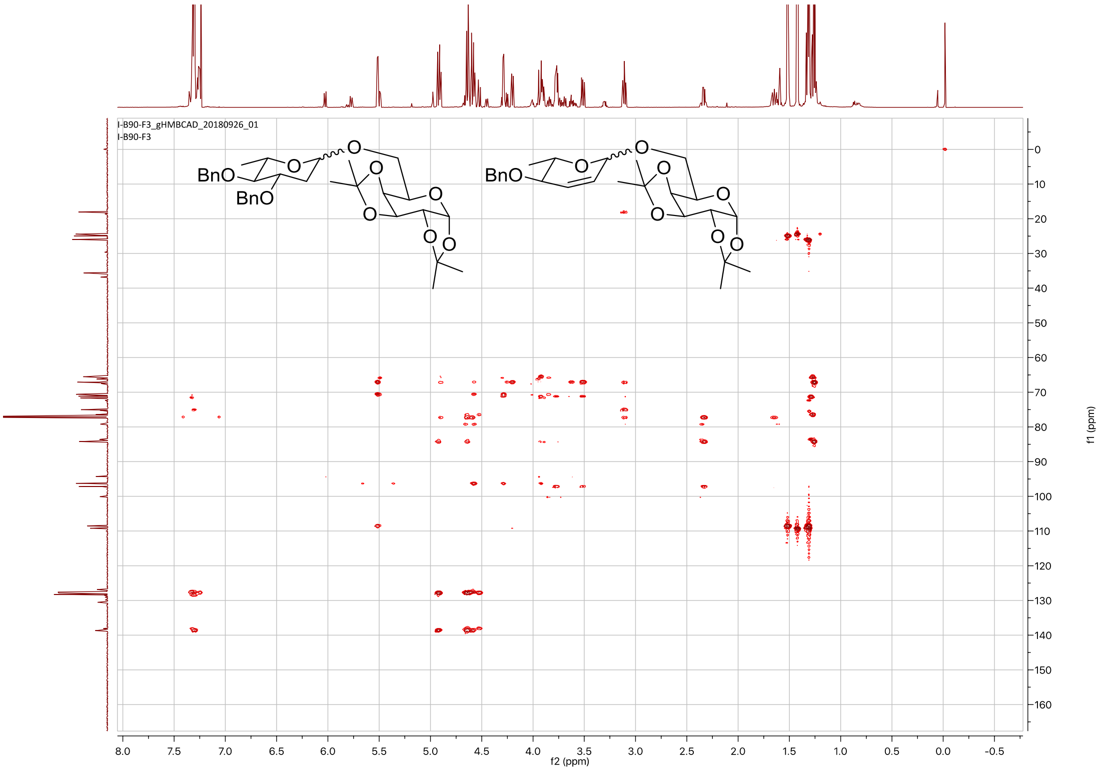

^{1}H NMR (400 MHz, Chloroform-*d*), (3,4-Di-*O*-benzyl-2-deoxy-α-L-fucopyranosyl)-(1→6)-1,2:3,4-di-*O*-isopropylidene-α-D-galactopyranoside α-5h and 3,4-Di-*O*-benzyl-2-deoxy-α-L-fucopyranosyl-(1→1)-(3',4'-Di-*O*-benzyl-2-deoxy-α-L-fucopyranosyl 9h

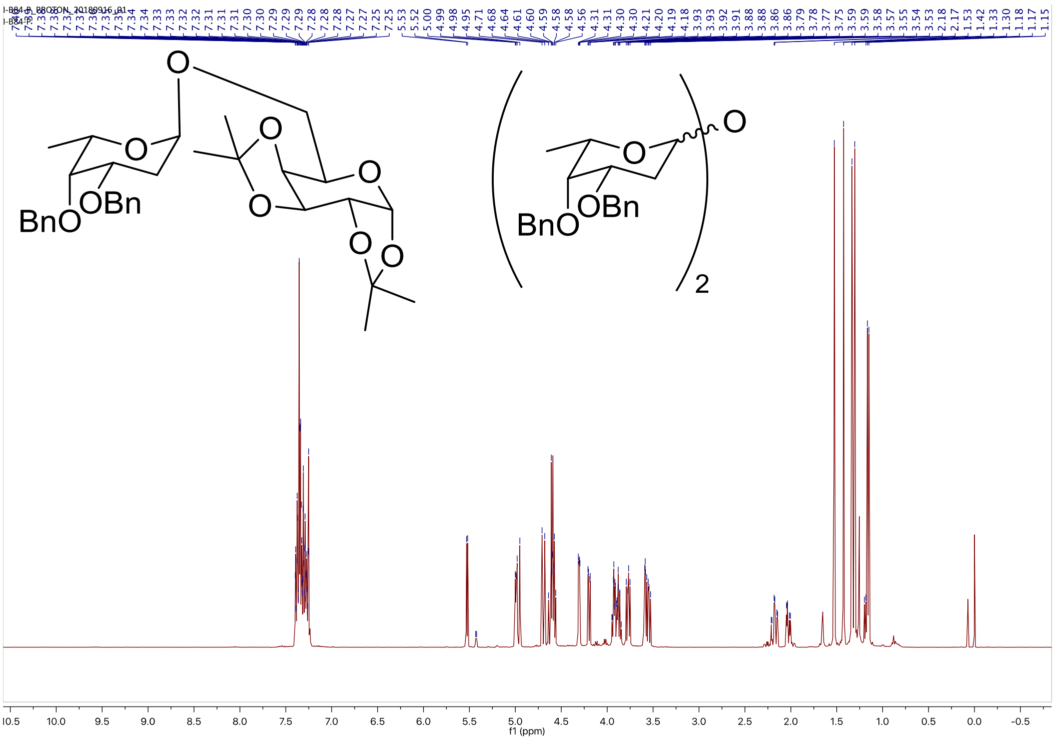

^{13}C NMR (101 MHz, Chloroform-*d*), (3,4-Di-*O*-benzyl-2-deoxy-α-L-fucopyranosyl)-(1→6)-1,2:3,4-di-*O*-isopropylidene-α-D-galactopyranoside α-5h and 3,4-Di-*O*-benzyl-2-deoxy-α-L-fucopyranosyl-(1→1)-(3',4'-Di-*O*-benzyl-2-deoxy-α-L-fucopyranosyl 9h

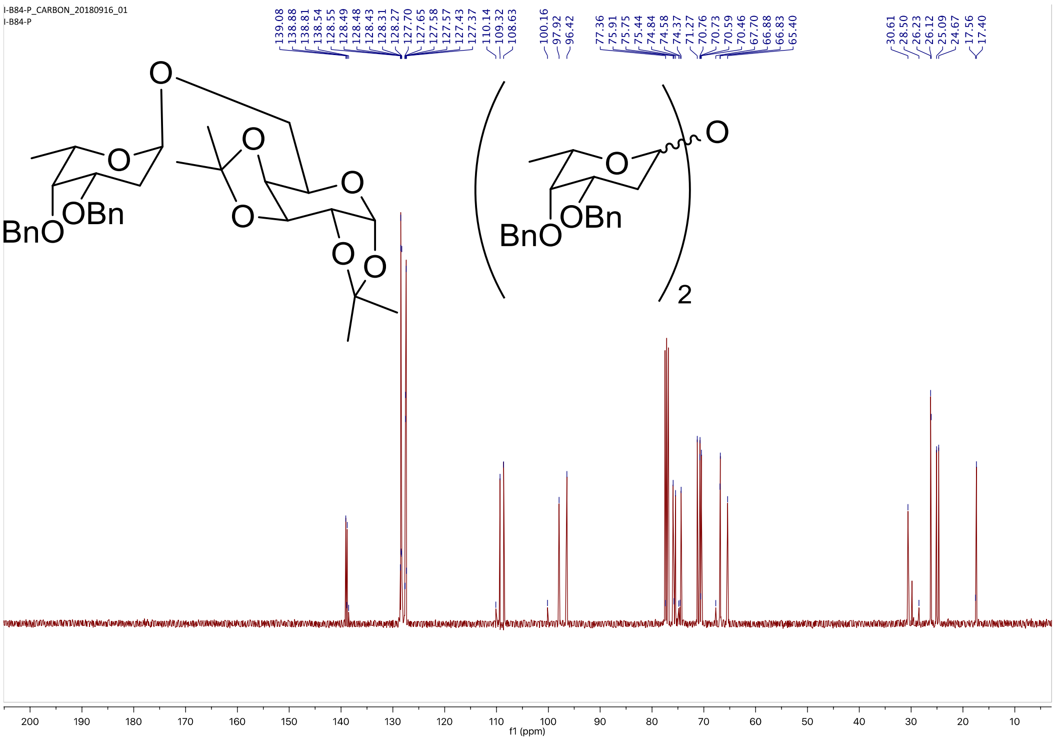

COSY (400 MHz, Chloroform-*d*), (3,4-Di-*O*-benzyl-2-deoxy-α-L-fucopyranosyl)-(1→6)-1,2:3,4-di-*O*-isopropylidene-α-D-galactopyranoside α-5h and 3,4-Di-*O*-benzyl-2-deoxy-α-L-fucopyranosyl-(1→1)-(3',4'-Di-*O*-benzyl-2-deoxy-α-L-fucopyranosyl 9h

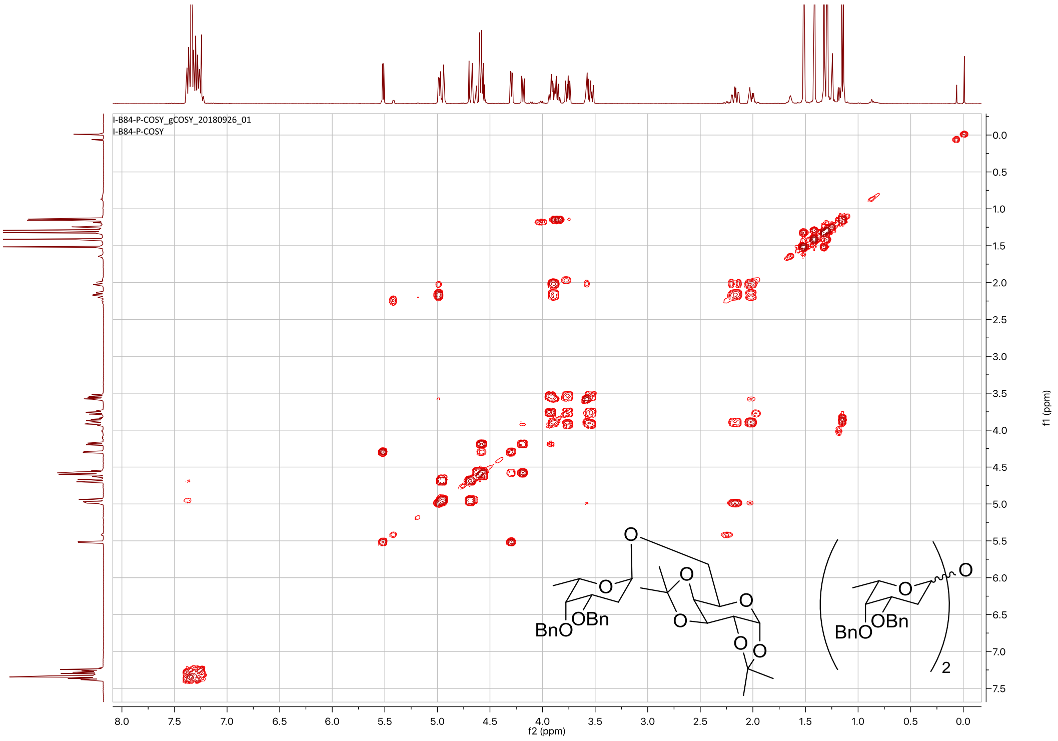

HSQC (400 × 101 MHz, Chloroform-*d*), (3,4-Di-*O*-benzyl-2-deoxy-α-L-fucopyranosyl)-(1→6)-1,2:3,4-di-*O*-isopropylidene-α-D-galactopyranoside α-5h and 3,4-Di-*O*-benzyl-2-deoxy-α-L-fucopyranosyl-(1→1)-(3',4'-Di-*O*-benzyl-2-deoxy-α-L-fucopyranosyl 9h

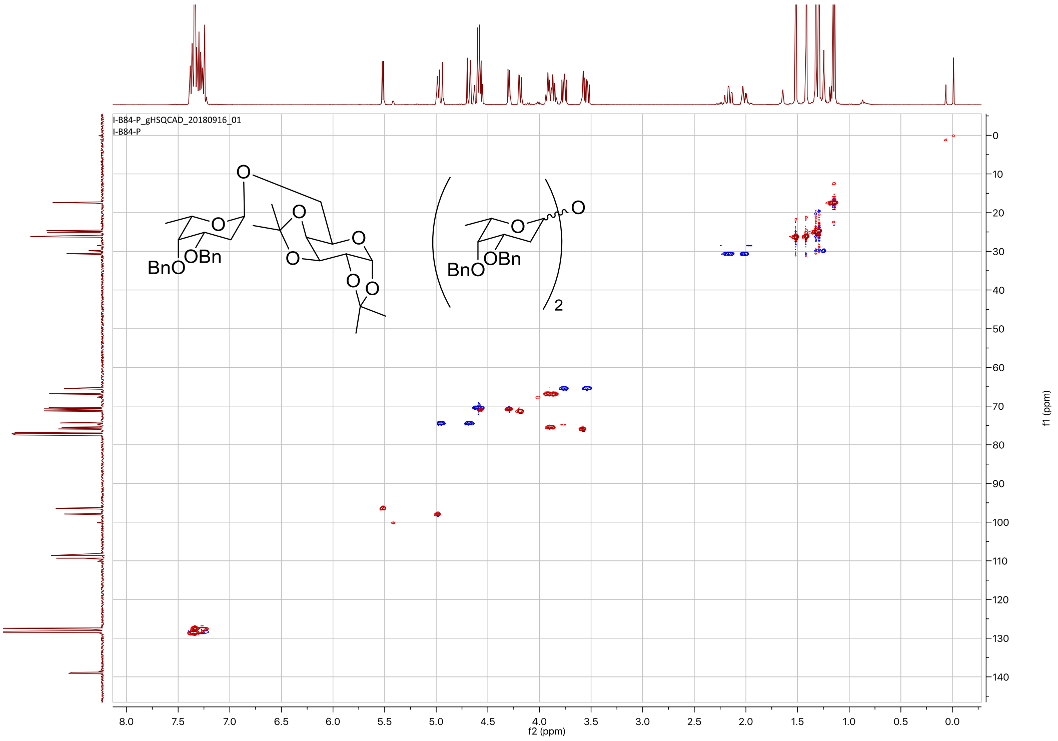

^{1}H NMR (600 MHz, Chloroform-*d*), (3,4-Di-*O*-benzyl-2-deoxy-β-L-fucopyranosyl)-(1→6)-1,2:3,4-di-*O*-isopropylidene-α-D-galactopyranoside β-5h

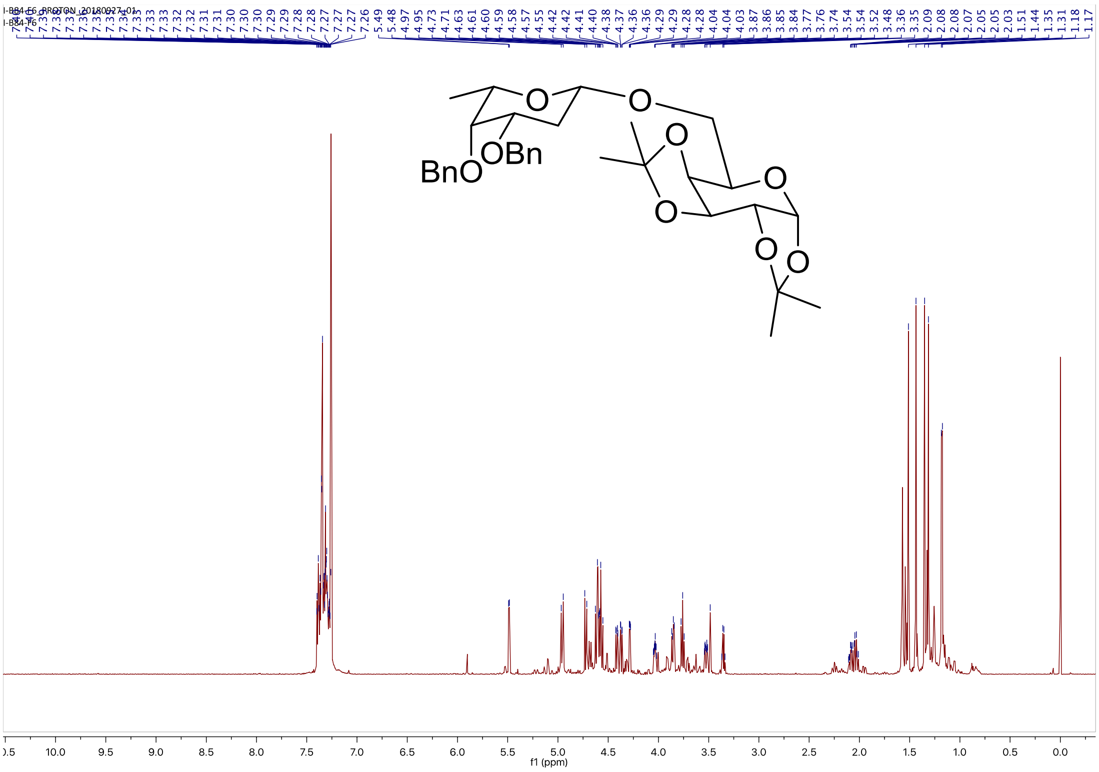

^{13}C NMR (151 MHz, Chloroform-*d*), (3,4-Di-*O*-benzyl-2-deoxy-β-L-fucopyranosyl)-(1→6)-1,2:3,4-di-*O*-isopropylidene-α-D-galactopyranoside β-5h

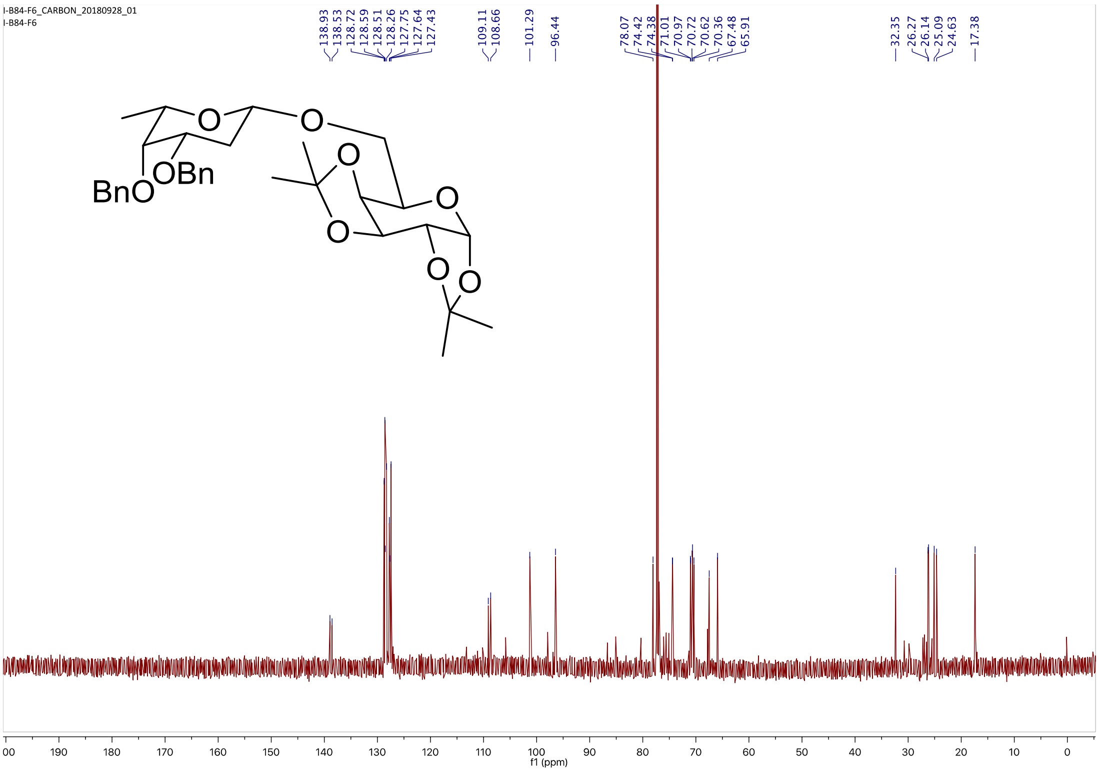

COSY (600 MHz, Chloroform-*d*), (3,4-Di-*O*-benzyl-2-deoxy-β-L-fucopyranosyl)-(1→6)-1,2:3,4-di-*O*-isopropylidene-α-D-galactopyranoside β-5h

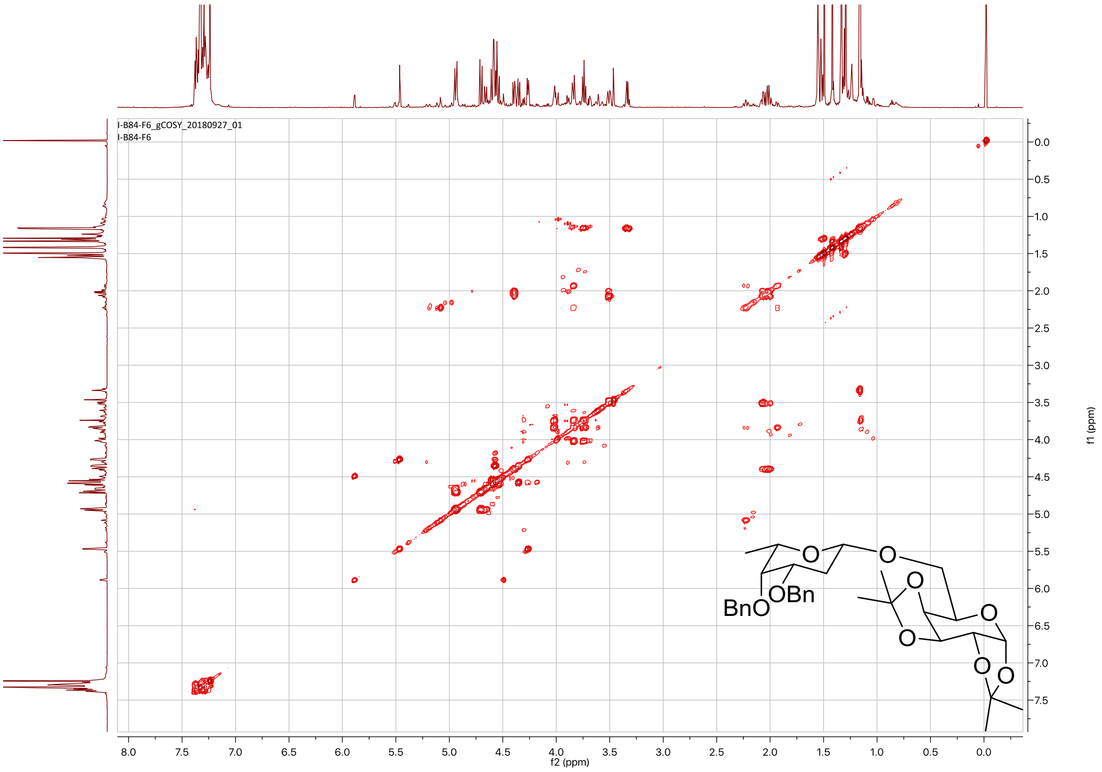

HSQC (600 × 151 MHz, Chloroform-*d*), (3,4-Di-*O*-benzyl-2-deoxy-β-L-fucopyranosyl)-(1→6)-1,2:3,4-di-*O*-isopropylidene-α-D-galactopyranoside β-5h

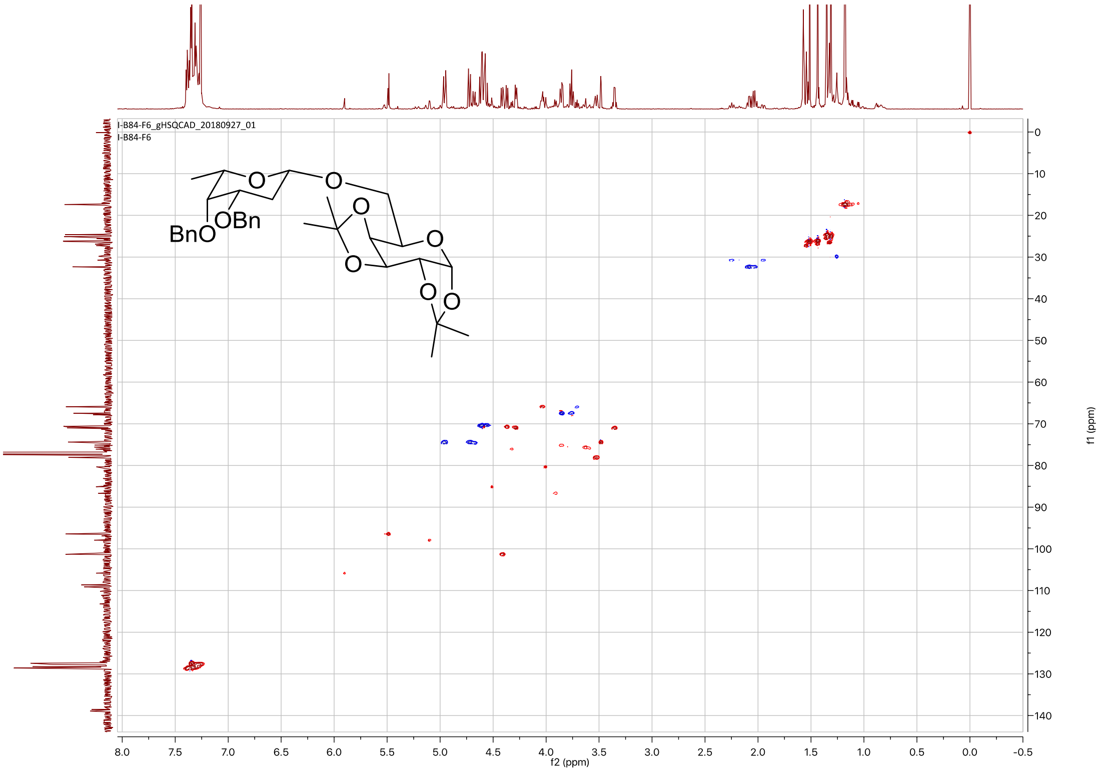

HMBC (600 × 151 MHz, Chloroform-*d*), (3,4-Di-*O*-benzyl-2-deoxy-β-L-fucopyranosyl)-(1→6)-1,2:3,4-di-*O*-isopropylidene-α-D-galactopyranoside β-5h

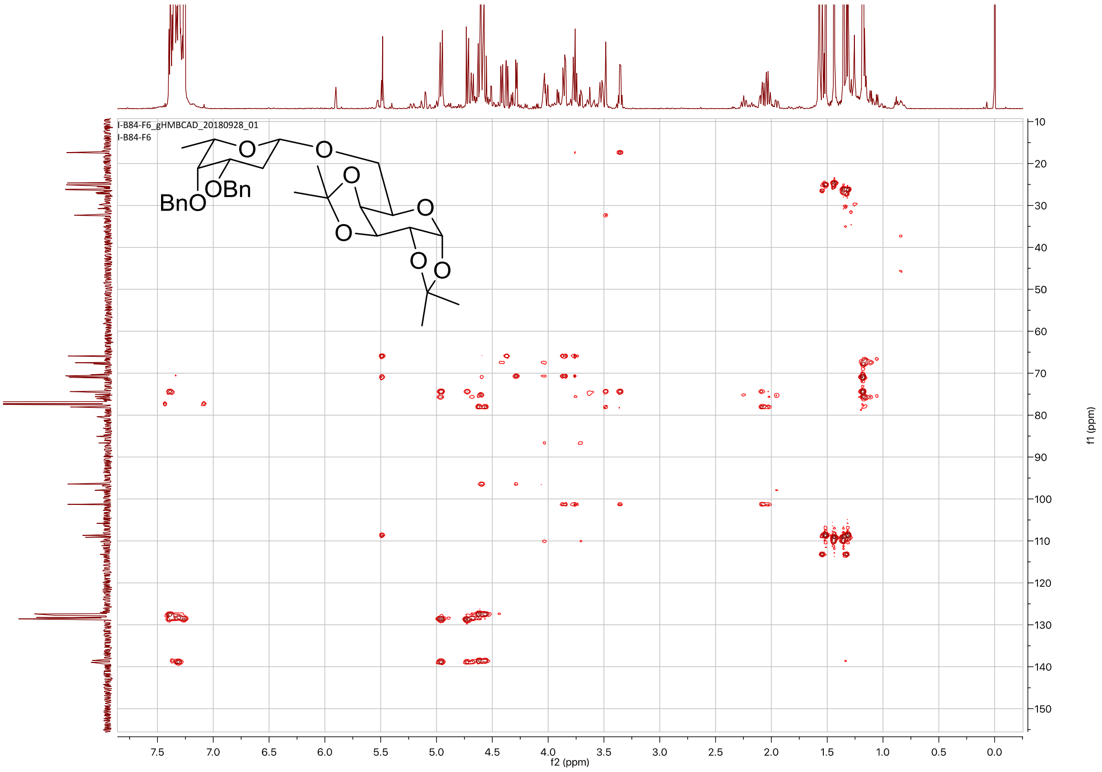

^{1}H NMR (400 MHz, $CDCl_3$), methyl 3-*O*-benzyl-2-*O*-(3,4,6-tri-*O*-benzyl-α-D-lyxo-hexapyranosyl)-4,6-*O*-benzylidene-α-D-glucopyranoside 7a

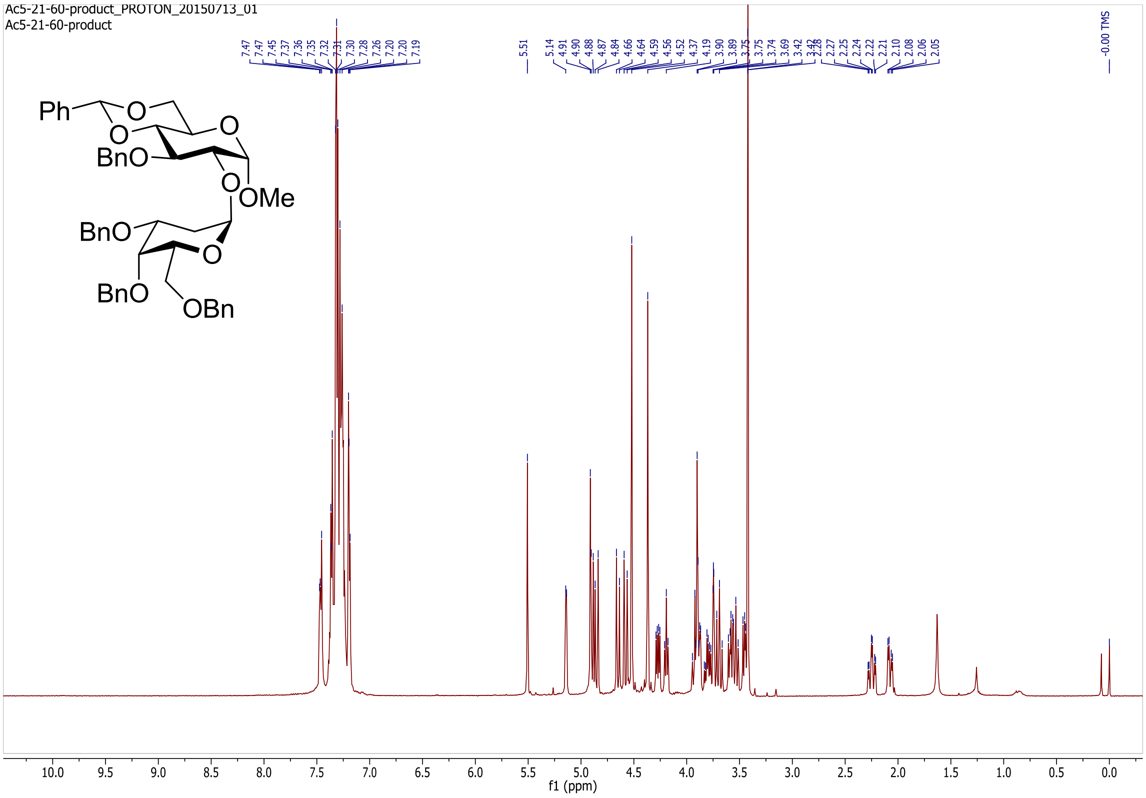

^{13}C NMR (101 MHz, $CDCl_3$), methyl 3-*O*-benzyl-2-*O*-(3,4,6-tri-*O*-benzyl-α-D-lyxo-hexapyranosyl)-4,6-*O*-benzylidene-α-D-glucopyranoside 7a

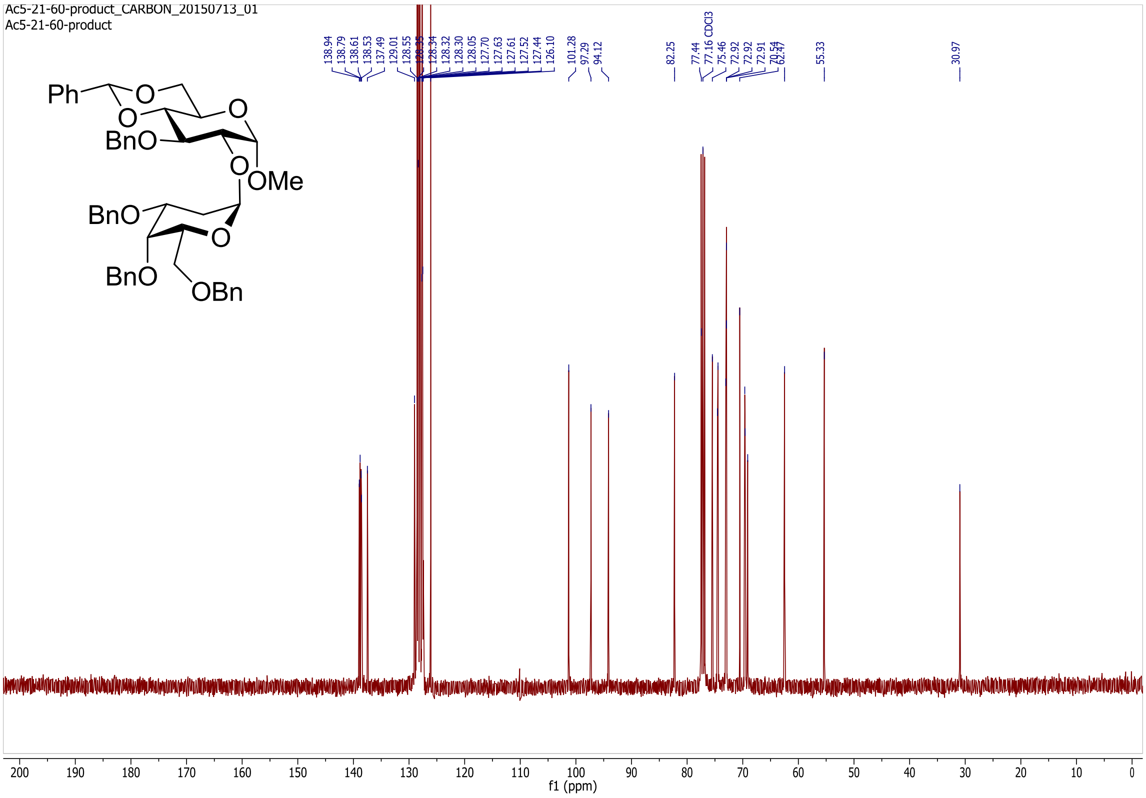

^{1}H NMR (400 MHz, $CDCl_3$), methyl 2-*O*-benzyl-3-*O*-(3,4,6-tri-*O*-benzyl-α-D-lyxo-hexapyranosyl)-4,6-*O*-benzylidene-α-D-glucopyranoside 7b

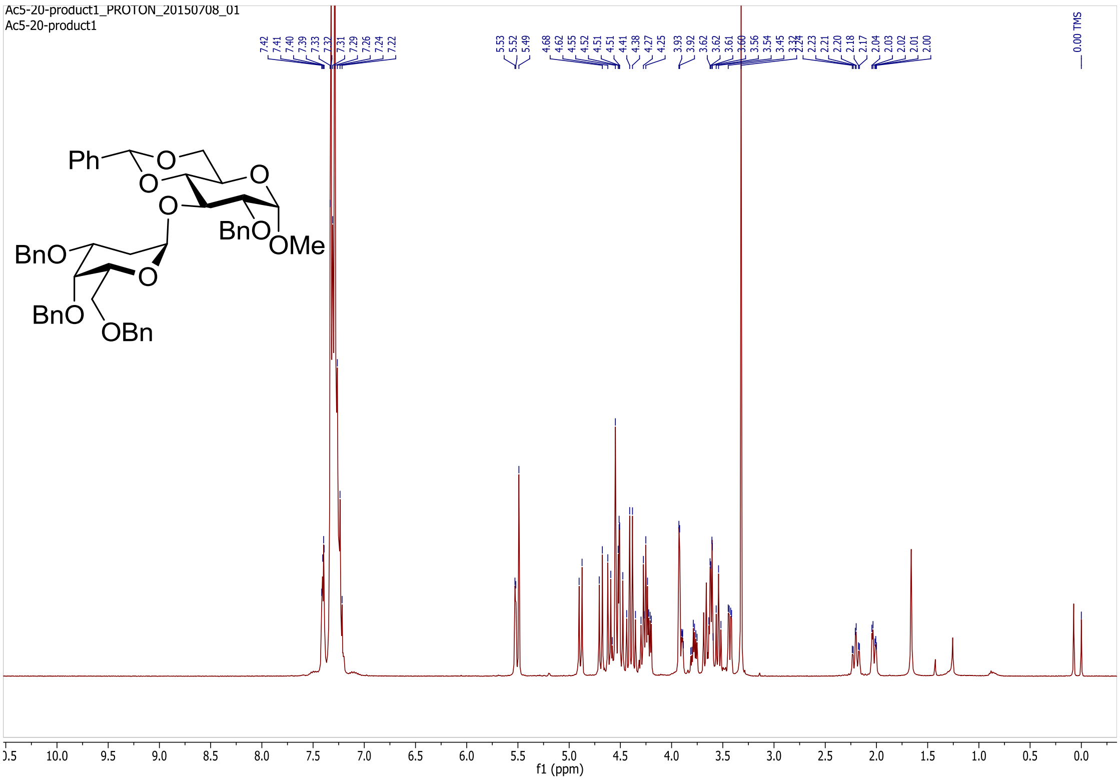

^{13}C NMR (101 MHz, $CDCl_3$), methyl 2-*O*-benzyl-3-*O*-(3,4,6-tri-*O*-benzyl-α-D-lyxo-hexapyranosyl)-4,6-*O*-benzylidene-α-D-glucopyranoside 7b

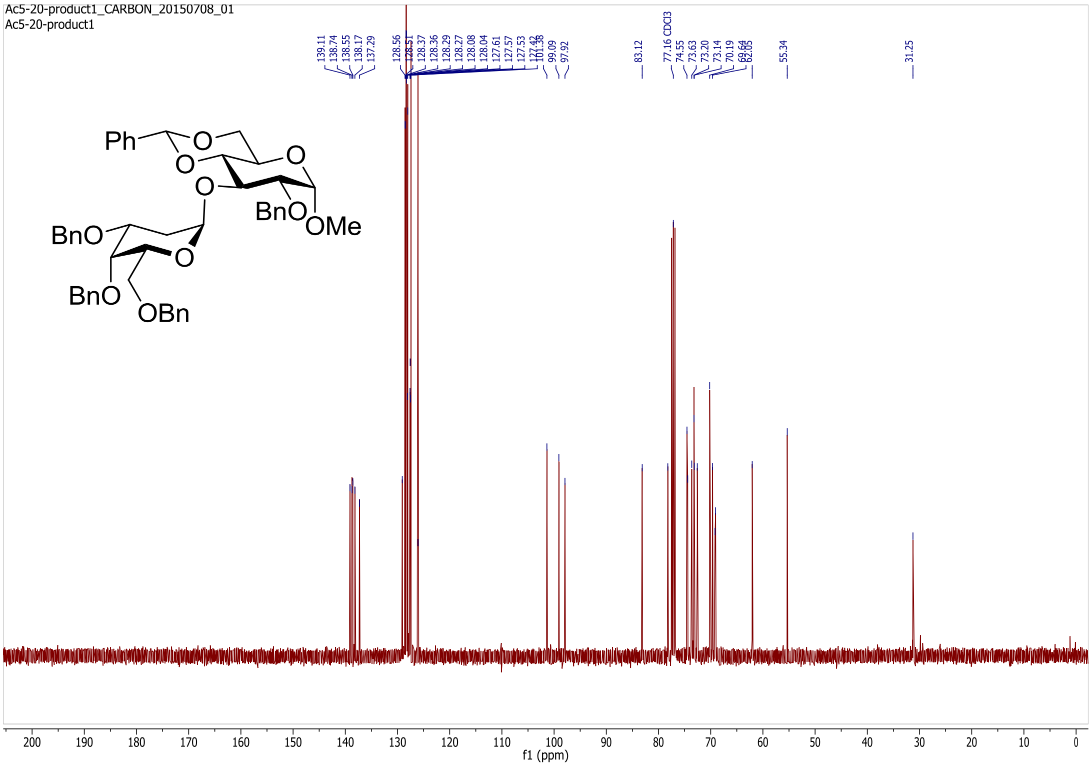

^{1}H NMR (400 MHz, CDCl$_3$), methyl 2,3-*O*-benzyl-4-*O*-(3,4,6-tri-*O*-benzyl-α-D-lyxo-hexapyranosyl)-α-D-glucopyranoside 7c

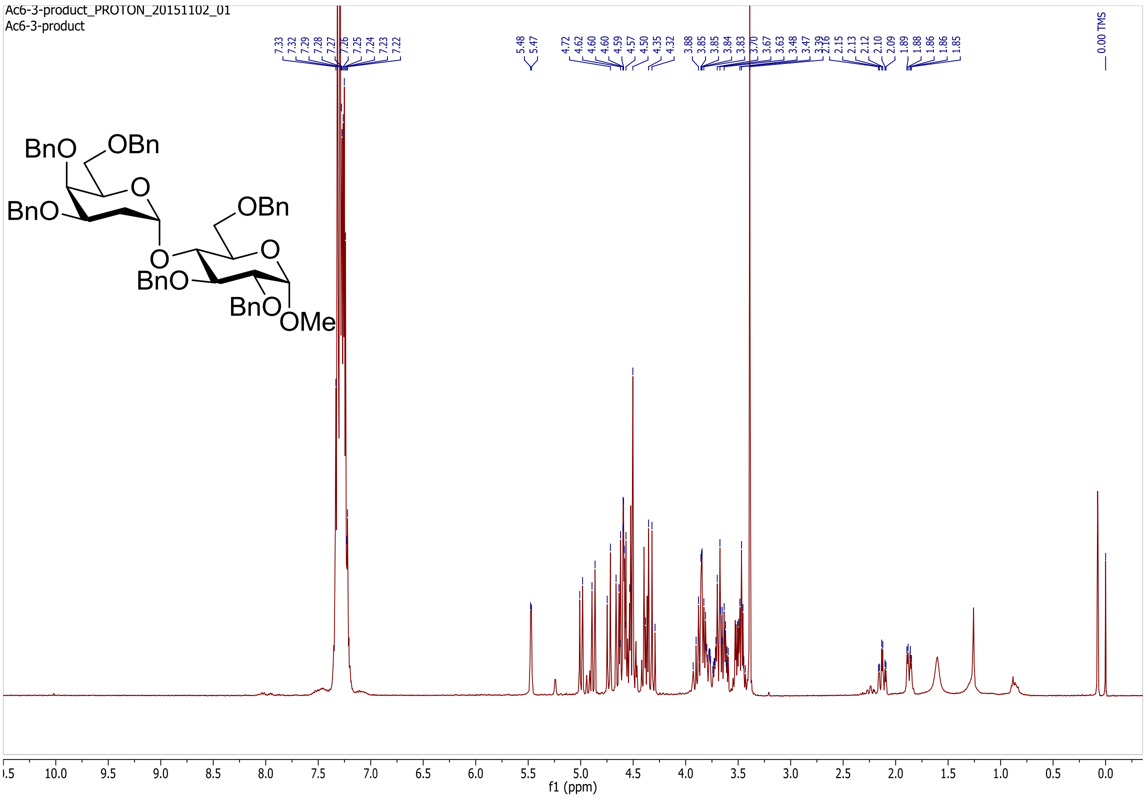

^{13}C NMR (101 MHz, CDCl$_3$), methyl 2,3-*O*-benzyl-4-*O*-(3,4,6-tri-*O*-benzyl-α-D-lyxo-hexapyranosyl)-α-D-glucopyranoside 7c

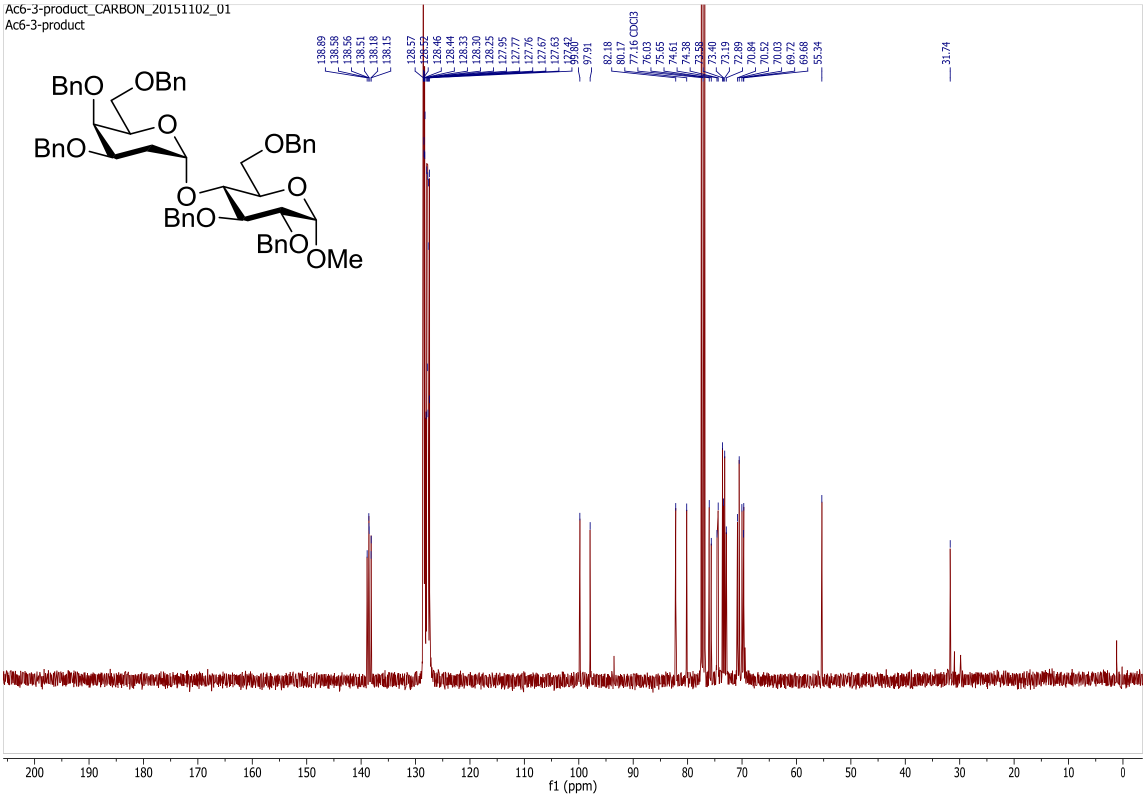

^{1}H NMR (300 MHz, $CDCl_3$), phenyl 2,3,4-tri-*O*-benzyl-6-*O*-(3,4,6-tri-*O*-benzyl-2-deoxy-α-D-lyxo-hexapyranosyl)-β-D-thioglucopyranoside 7d

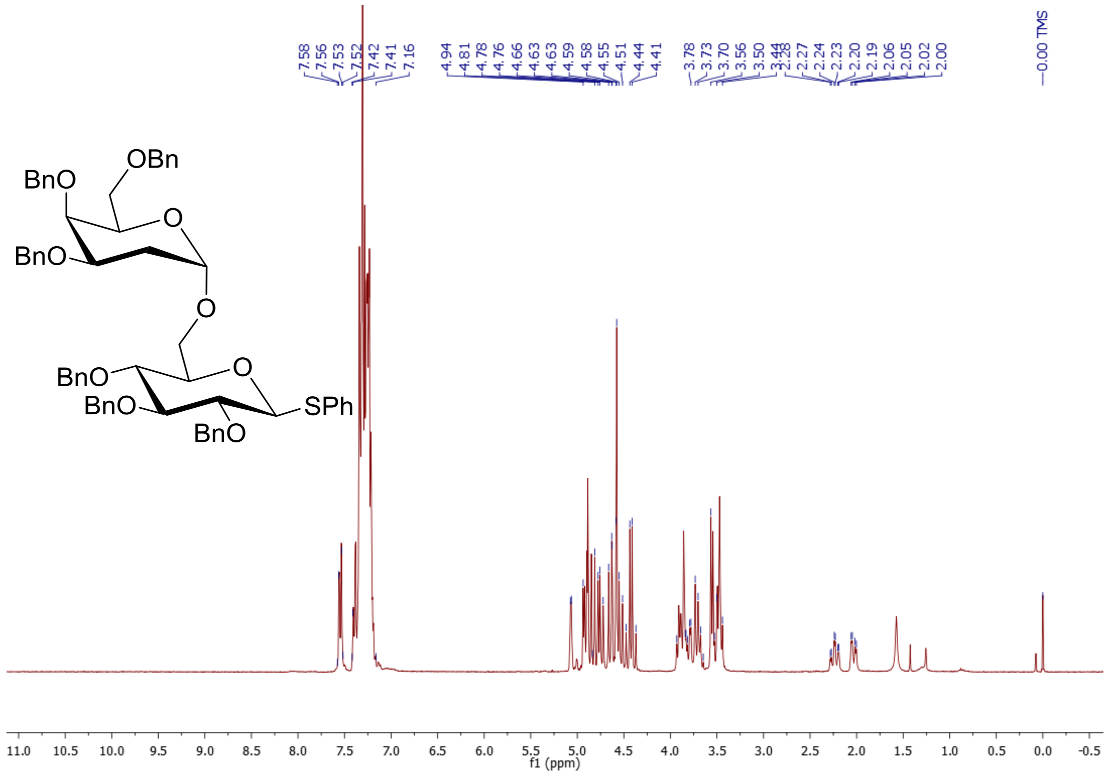

^{13}C NMR (101 MHz, $CDCl_3$), phenyl 2,3,4-tri-*O*-benzyl-6-*O*-(3,4,6-tri-*O*-benzyl-2-deoxy-α-D-lyxo-hexapyranosyl)-β-D-thioglucopyranoside 7d

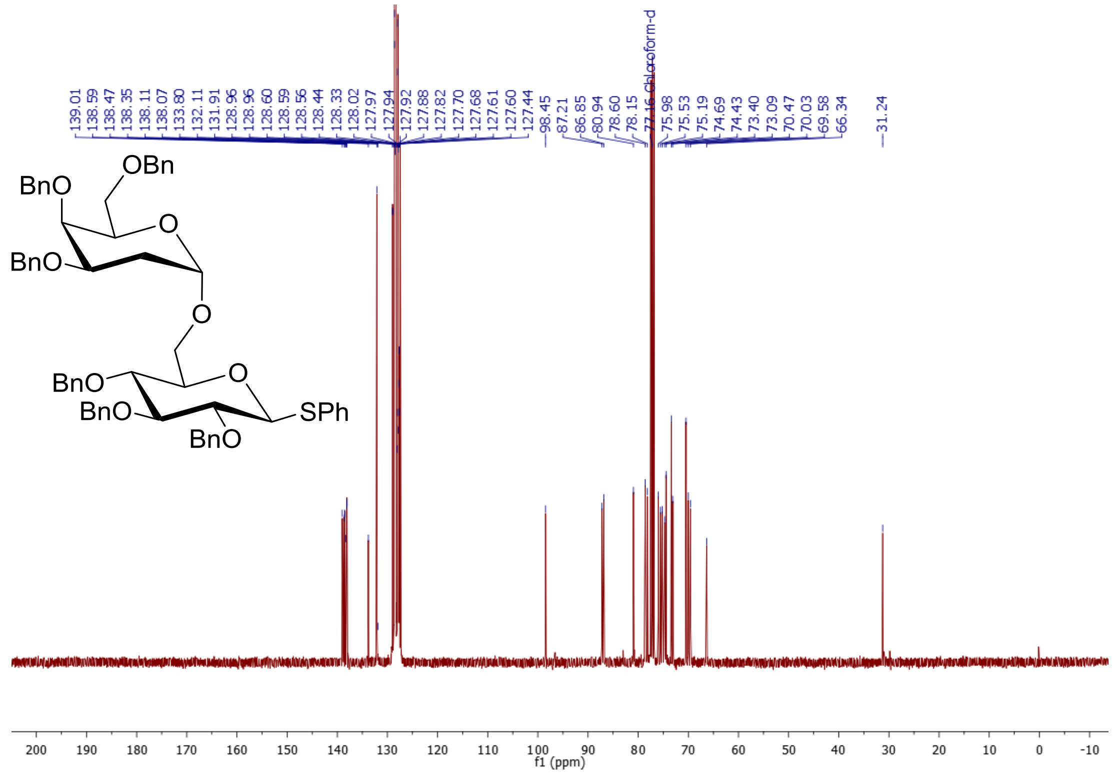

^{1}H NMR (500 MHz, CDCl$_3$), 2-deoxy-3,4,6-tri-*O*-benzyl-α-D-lyxo-hexapyranosyl-(1→*O*)-*N*-*tert*-butoxycarbonyl-L-serine methyl ester 7e

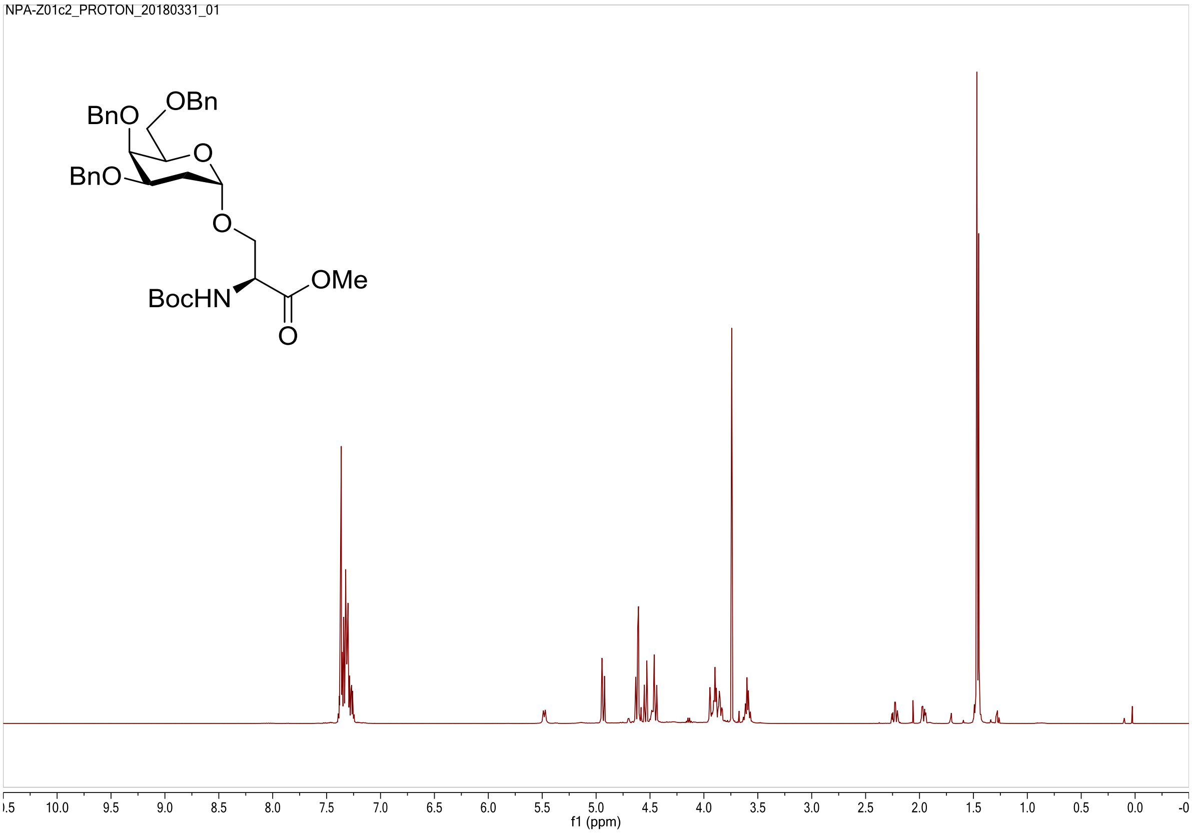

^{13}C NMR (126 MHz, CDCl$_3$), 2-deoxy-3,4,6-tri-*O*-benzyl-α-D-lyxo-hexapyranosyl-(1→*O*)-*N*-*tert*-butoxycarbonyl-L-serine methyl ester 7e

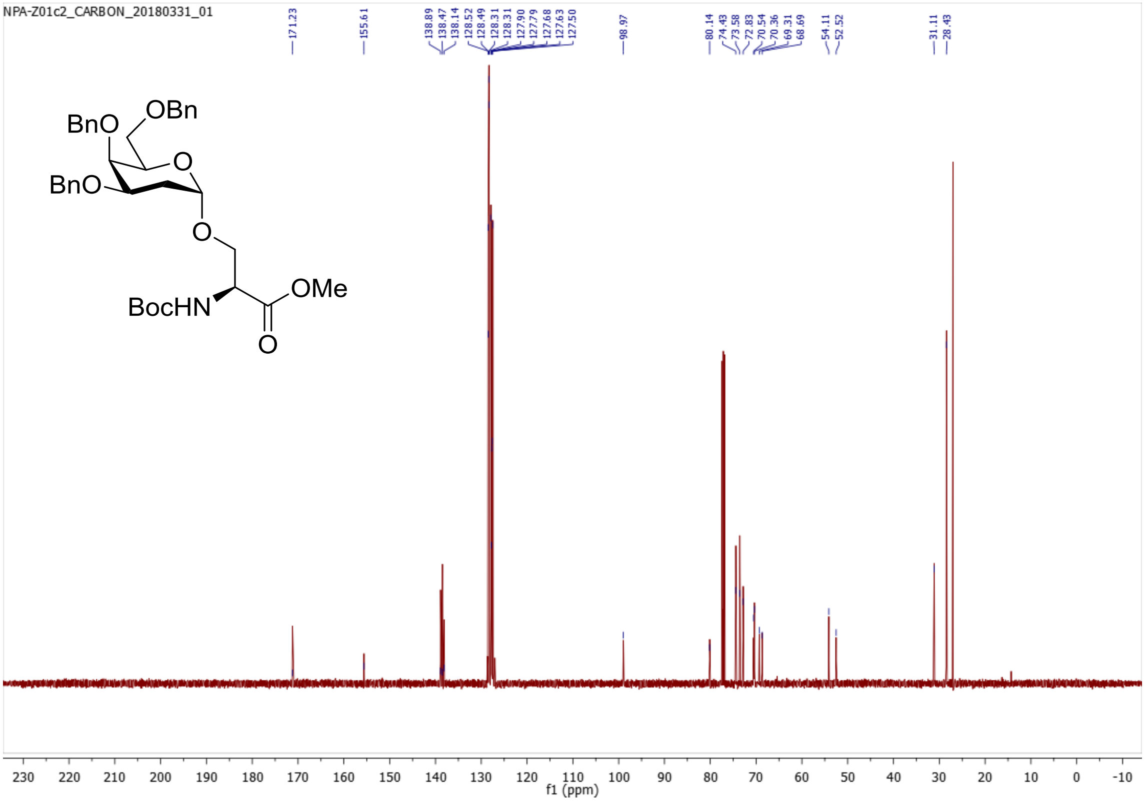

gHSQC NMR (500 × 126 MHz, CDCl₃), 2-deoxy-3,4,6-tri-*O*-benzyl-α-D-lyxo-hexapyranosyl-(1→*O*)-*N*-*tert*-butoxycarbonyl-L-serine methyl ester 7e

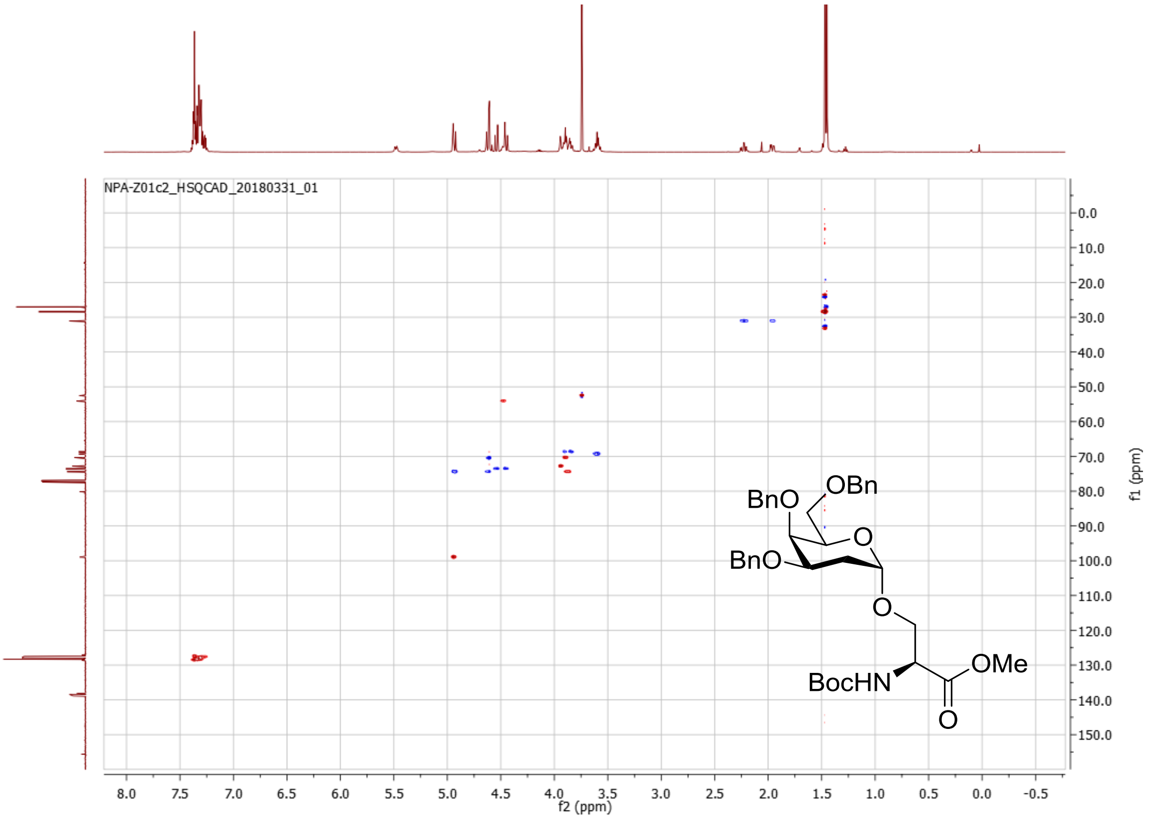

gHMBC NMR (500 × 126 MHz, CDCl₃), 2-deoxy-3,4,6-tri-*O*-benzyl-α-D-lyxo-hexapyranosyl-(1→*O*)-*N*-*tert*-butoxycarbonyl-L-serine methyl ester 7e

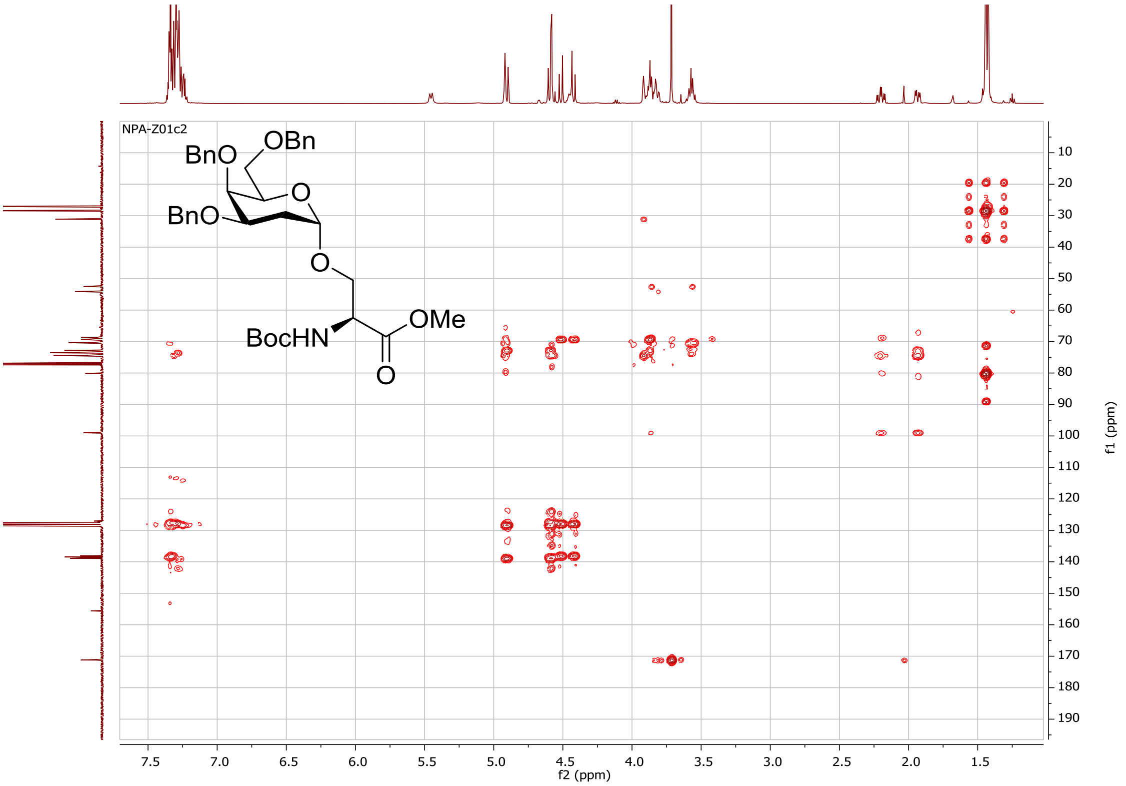

^{1}H NMR (400 MHz, $CDCl_3$), 2-deoxy-3,4,6-tri-*O*-benzyl-α-D-lyxo-hexopyranosyl-(1→*O*)-*N*-*tert*-butoxycarbonyl-L-threonine methyl ester 7f

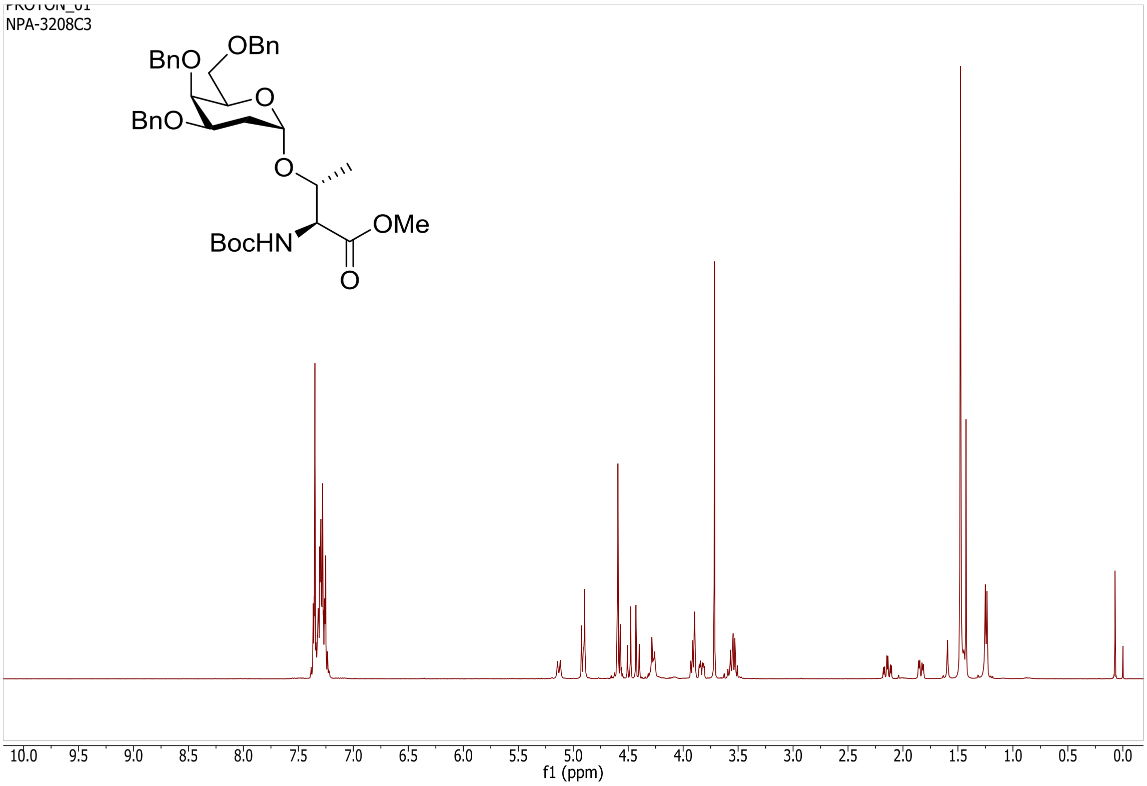

^{13}C NMR (101 MHz, $CDCl_3$), 2-deoxy-3,4,6-tri-*O*-benzyl-α-D-lyxo-hexopyranosyl-(1→*O*)-*N*-*tert*-butoxycarbonyl-L-threonine methyl ester 7f

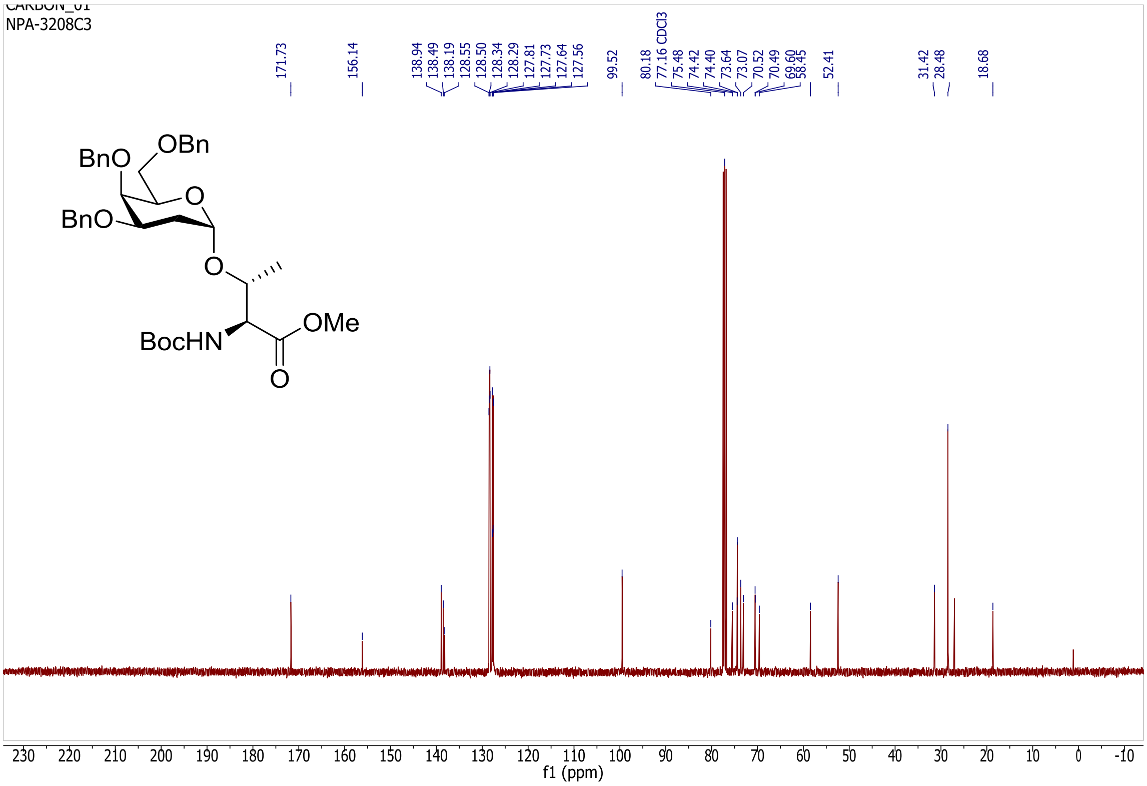

gHSQC NMR (400 × 101 MHz, $CDCl_3$), 2-deoxy-3,4,6-tri-*O*-benzyl-α-D-lyxo-hexopyranosyl-(1→*O*)-*N*-*tert*-butoxycarbonyl-L-threonine methyl ester 7f

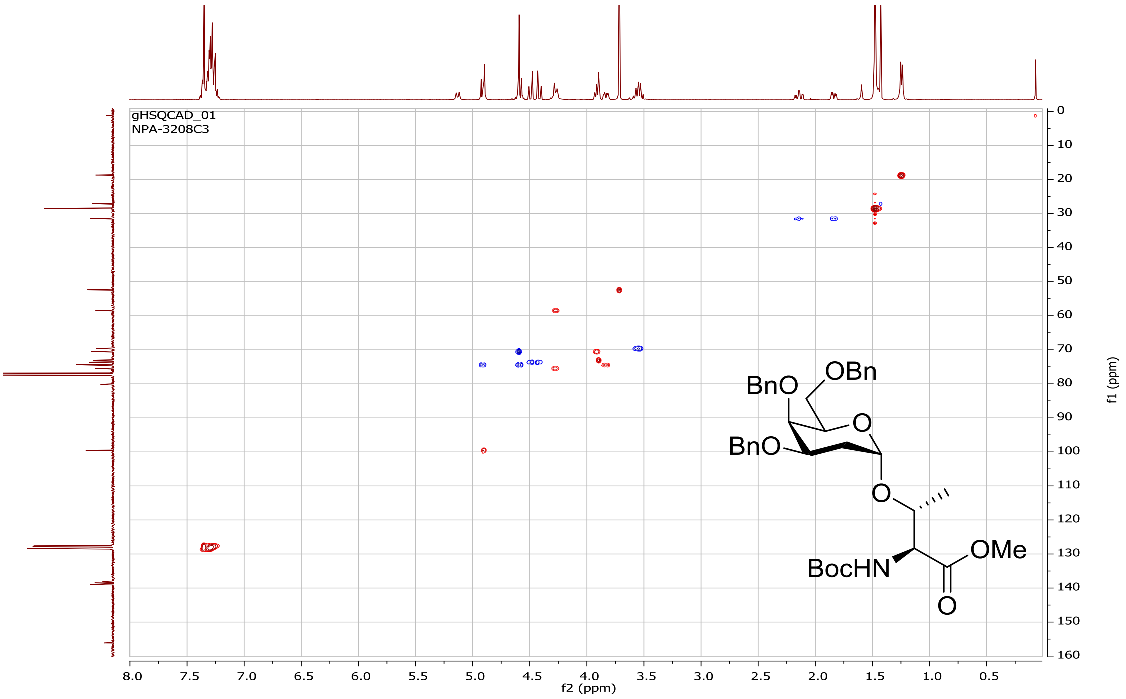

gHMBC NMR (400 × 100 MHz, $CDCl_3$), 2-deoxy-3,4,6-tri-*O*-benzyl-α-D-lyxo-hexopyranosyl-(1→*O*)-*N*-*tert*-butoxycarbonyl-L-threonine methyl ester 7f

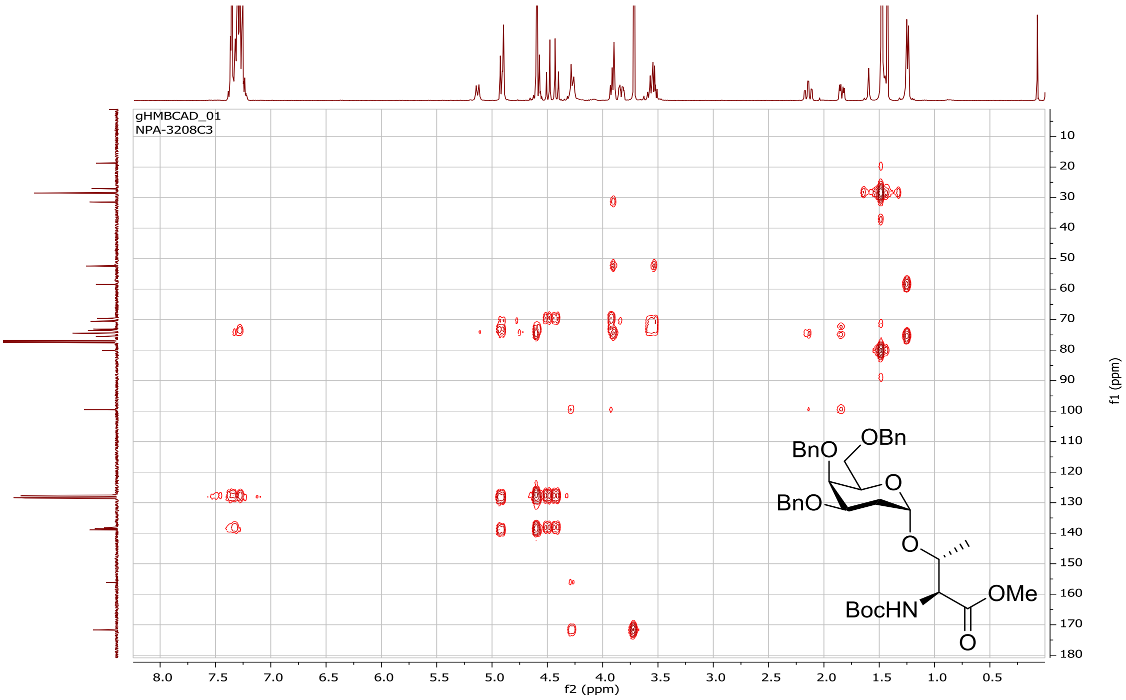

^{1}H NMR (400 MHz, CDCl$_3$), 2-deoxy-3,4,6-tri-*O*-benzyl-α-D-lyxo-hexopyranosyl-(1→*O*)-*N*-*tert*-butoxycarbonyl-L-tyrosine methyl ester 7g

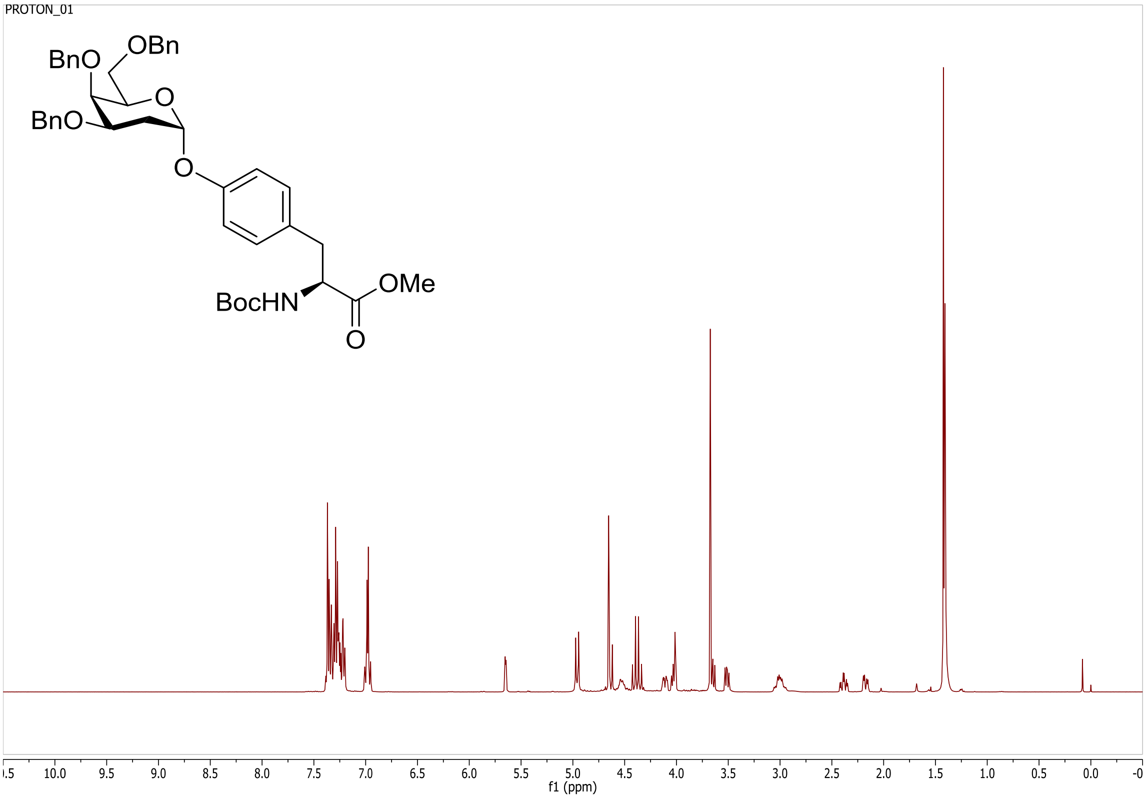

^{13}C NMR (101 MHz, CDCl$_3$), 2-deoxy-3,4,6-tri-*O*-benzyl-α-D-lyxo-hexopyranosyl-(1→*O*)-*N*-*tert*-butoxycarbonyl-L-tyrosine methyl ester 7g

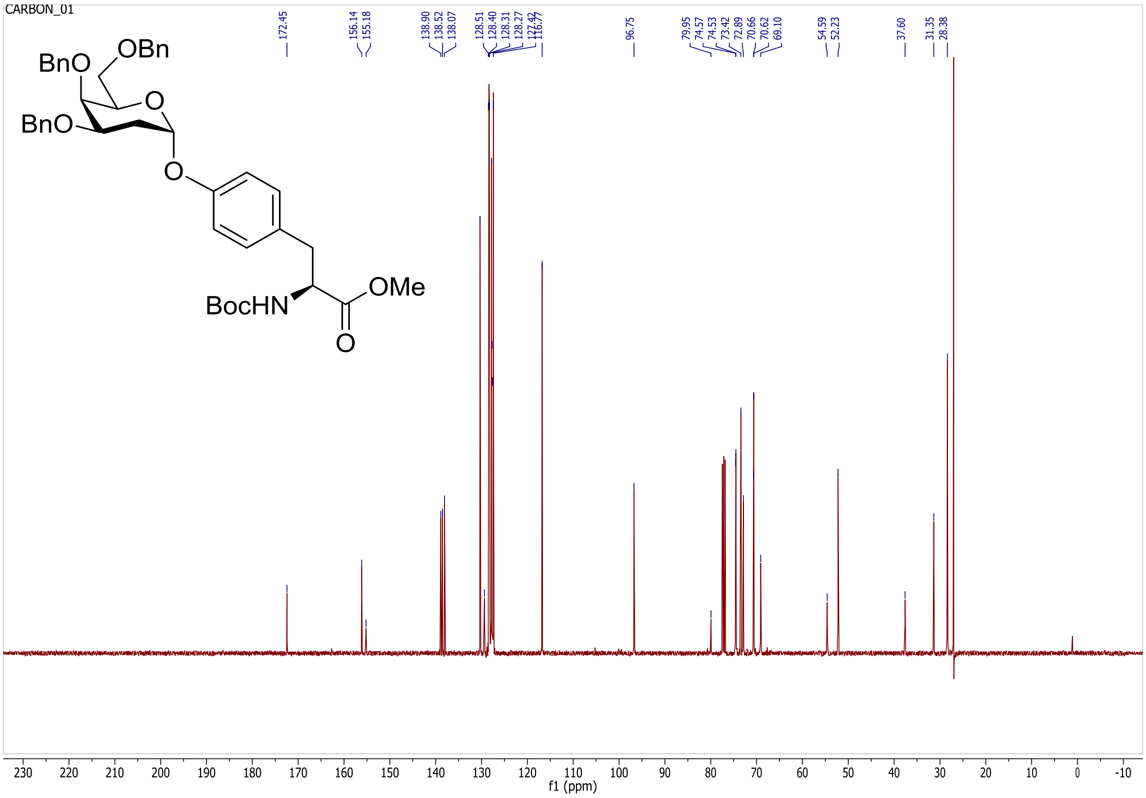

gHSQC NMR (400 × 101 MHz, CDCl$_3$), 2-deoxy-3,4,6-tri-*O*-benzyl-α-D-lyxo-hexopyranosyl-(1→*O*)-*N*-*tert*-butoxycarbonyl-L-tyrosine methyl ester 7g

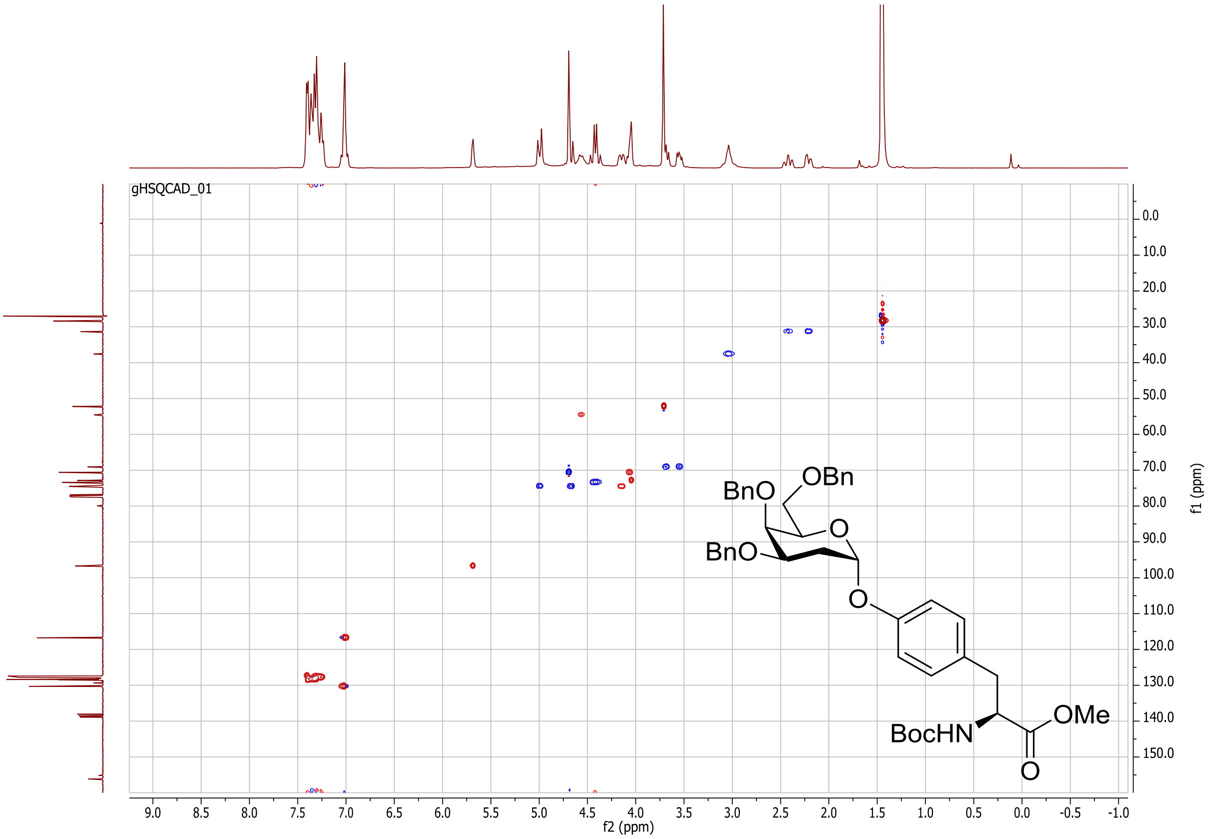

^1H NMR (500 MHz, $CDCl_3$), cholesteryl (3,4,6-tri-*O*-benzyl-2-deoxy-α-D-lyxo-hexapyranosyl) 7h

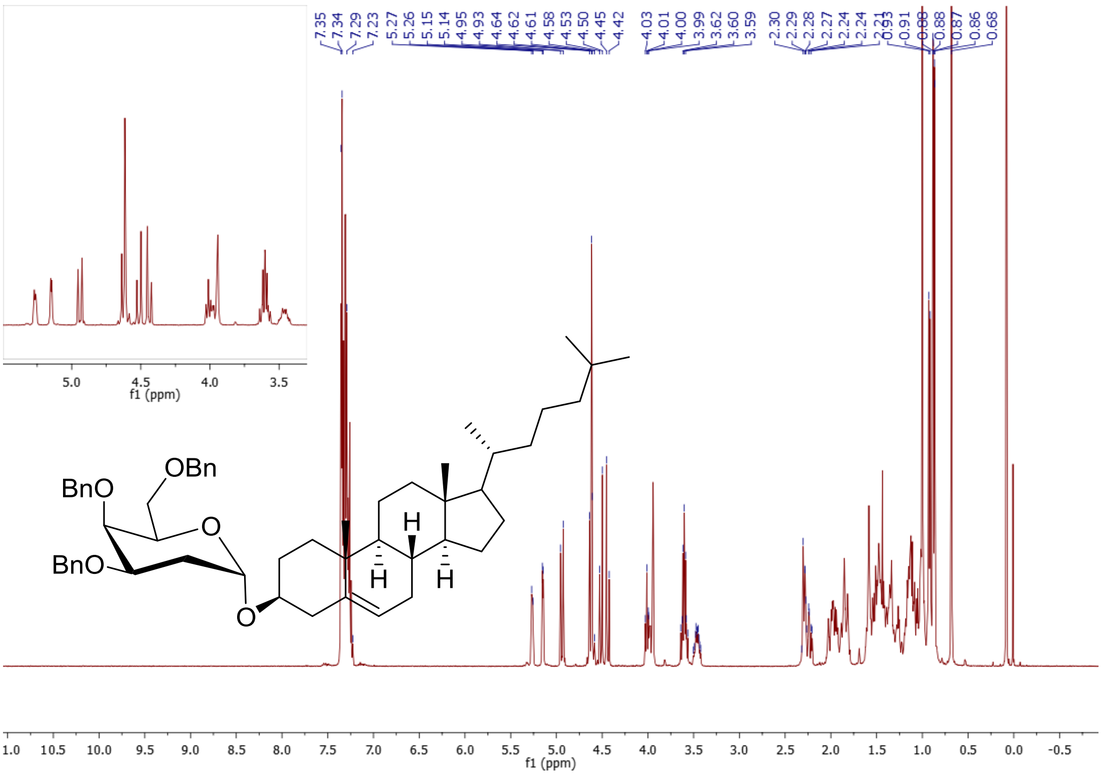

^{13}C NMR (125 MHz, $CDCl_3$), cholesteryl (3,4,6-tri-*O*-benzyl-2-deoxy-α-D-lyxo-hexapyranosyl) 7h

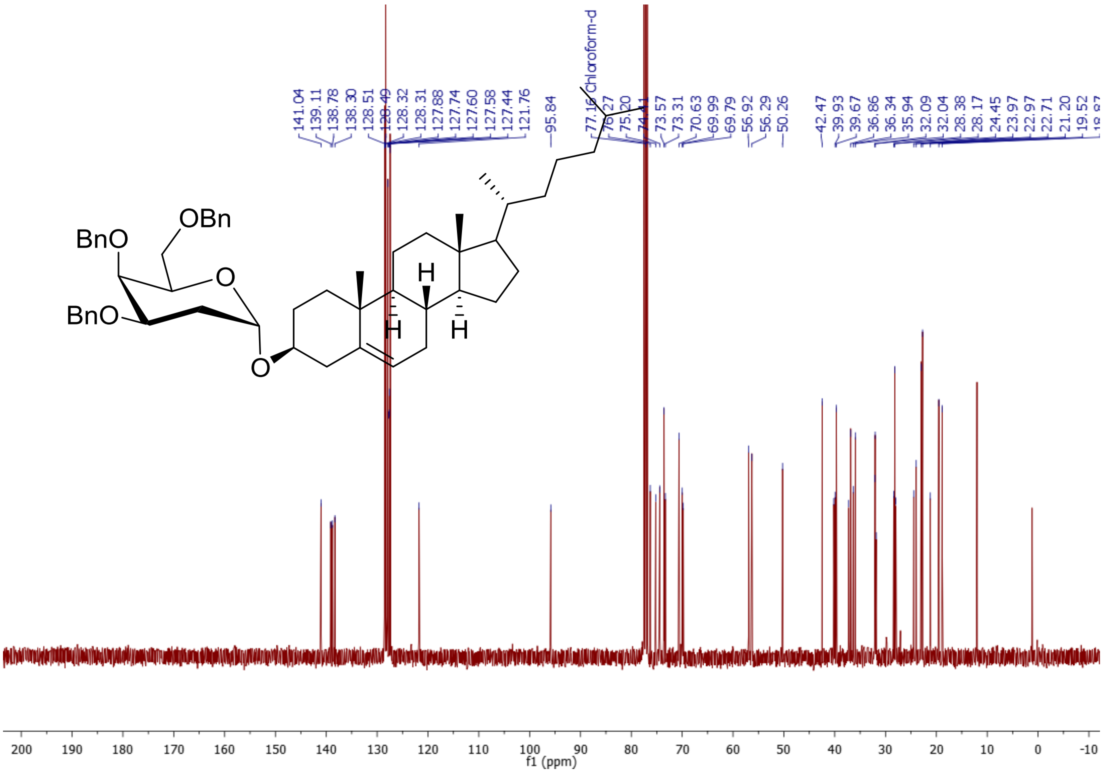

^{1}H NMR (500 MHz, CDCl$_3$), 3,4,6-Tri-*O*-benzyl-2-deoxy-α/β-D-galactopyranosyl *p*-toluenesulfonamide 12

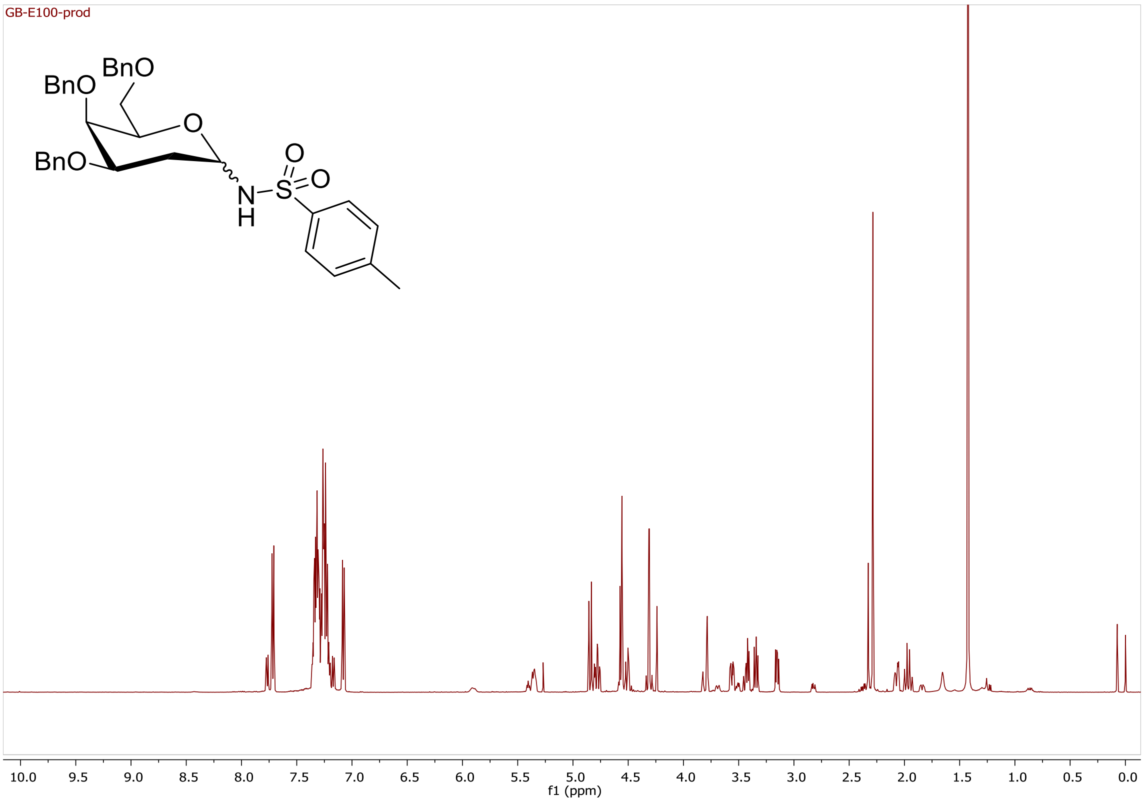

^{13}C NMR (125 MHz, CDCl$_3$) 3,4,6-Tri-*O*-benzyl-2-deoxy-α/β-D-galactopyranosyl *p*-toluenesulfonamide 12

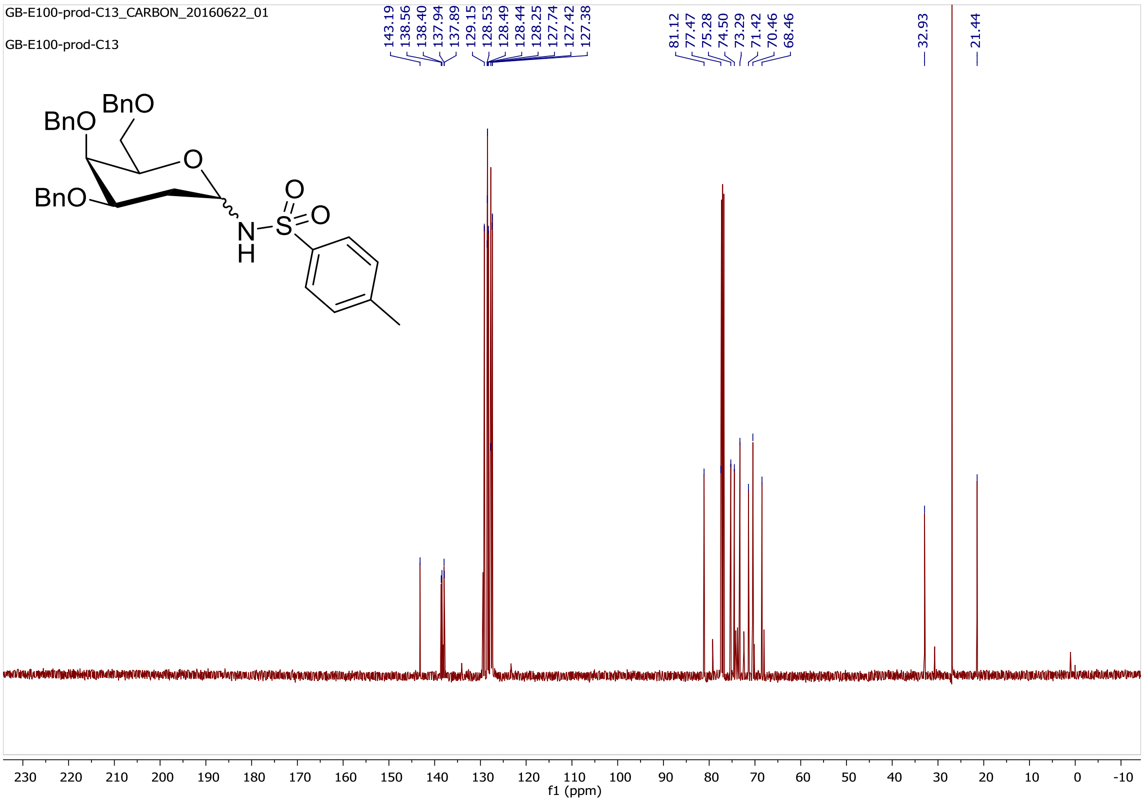

COSY NMR (500 MHz, $CDCl_3$), 3,4,6-Tri-*O*-benzyl-2-deoxy-α/β-D-galactopyranosyl *p*-toluenesulfonamide 12

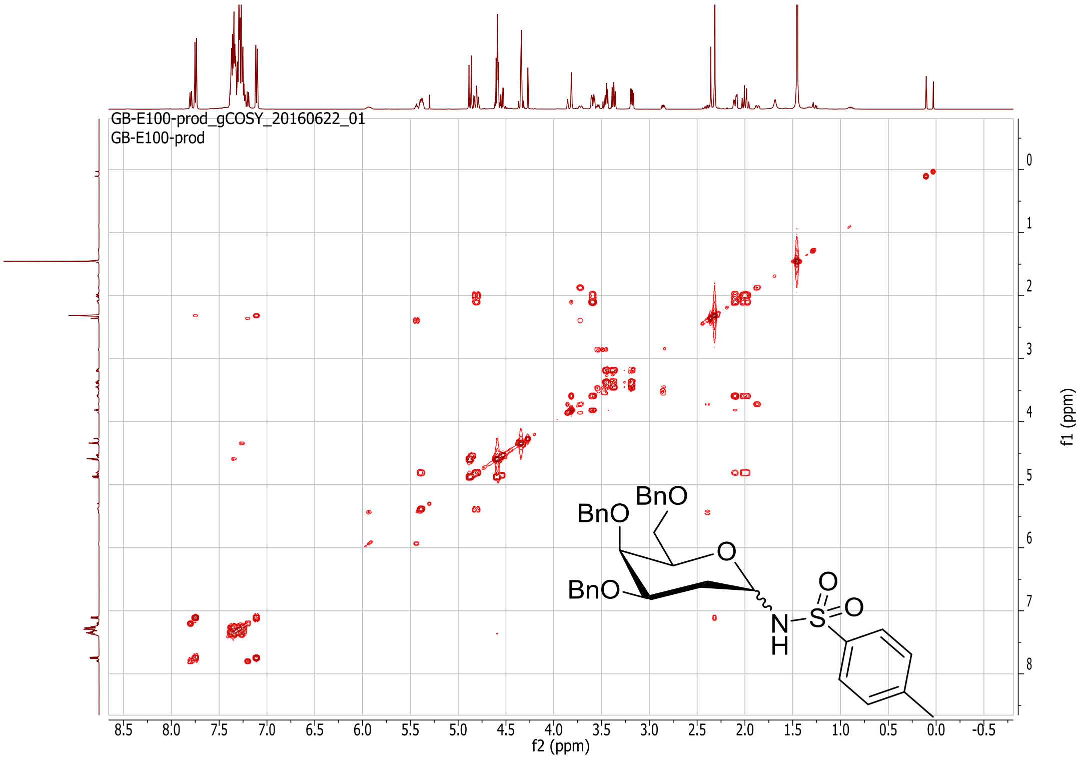

HSQC NMR (500 × 125 MHz, $CDCl_3$), 3,4,6-Tri-*O*-benzyl-2-deoxy-α/β-D-galactopyranosyl *p*-toluenesulfonamide 12

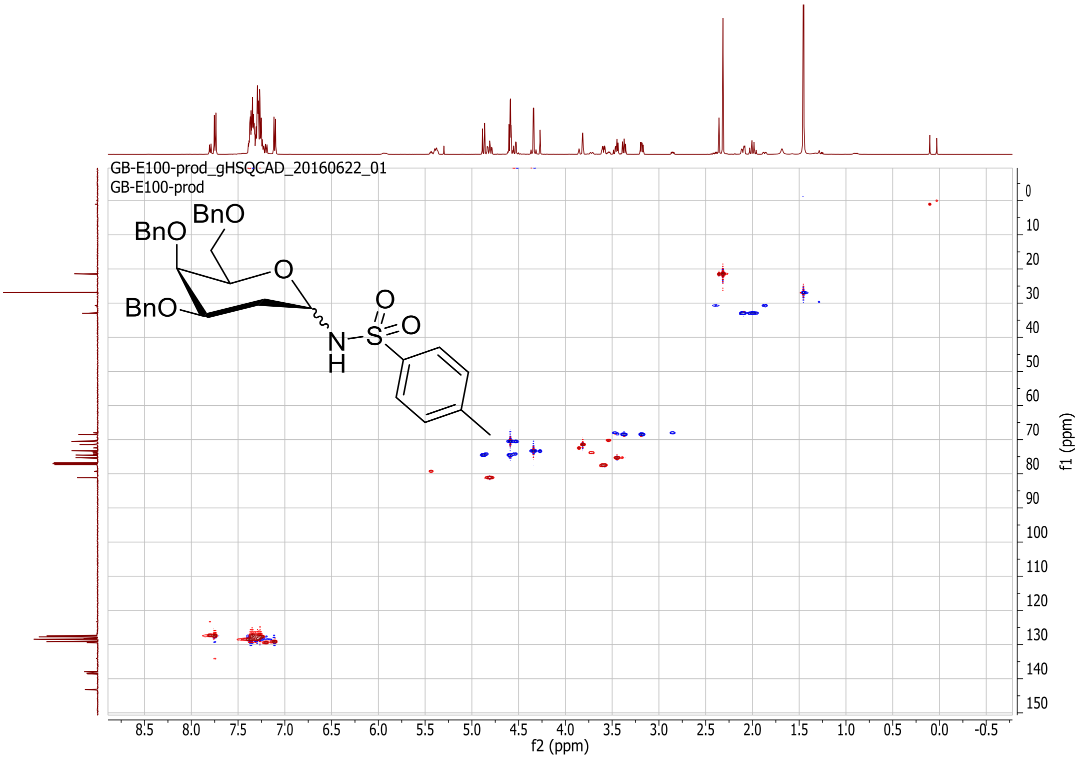

NMR Spectra of 1,1,-linked sugars

^{1}H NMR (400 MHz, CDCl$_3$), 3,4,6-tri-*O*-benzyl-α-D-lyxo-hexapyranosyl-(1→1')-3',4',6'-tri-*O*-benzyl-α-D-lyxo-hexapyranoside α,α-9a

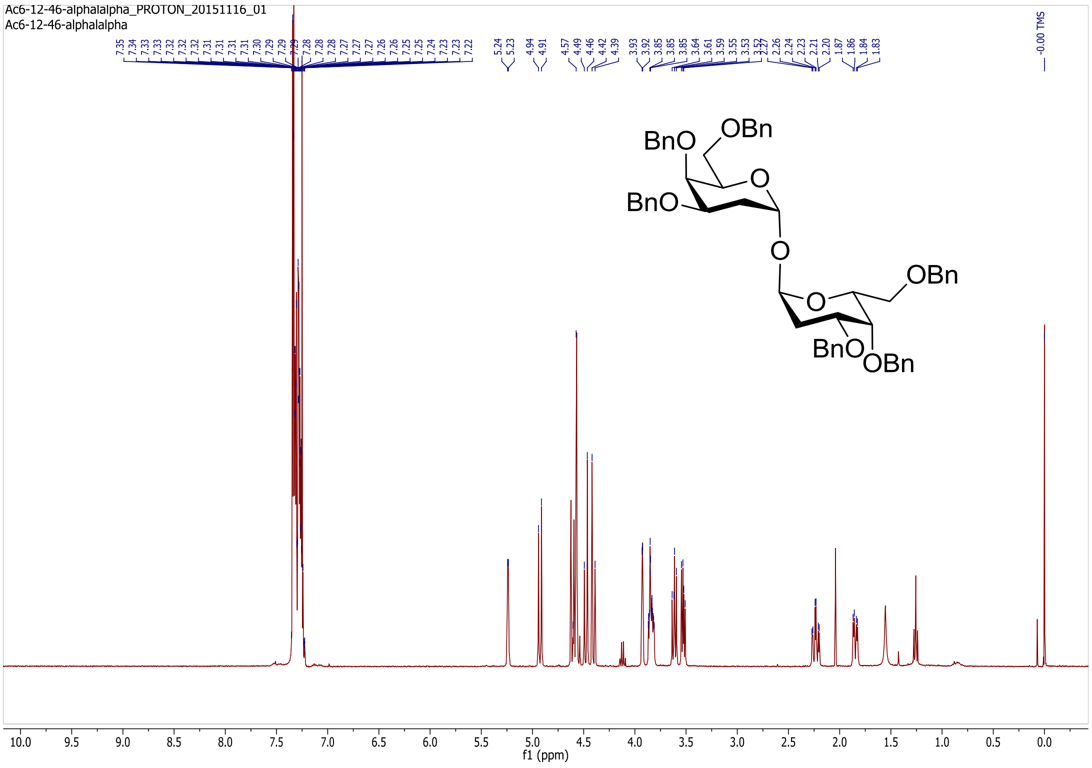

^{13}C NMR (101 MHz, CDCl$_3$), 3,4,6-tri-*O*-benzyl-α-D-lyxo-hexapyranosyl-(1→1')-3',4',6'-tri-*O*-benzyl-α-D-lyxo-hexapyranoside α,α-9a

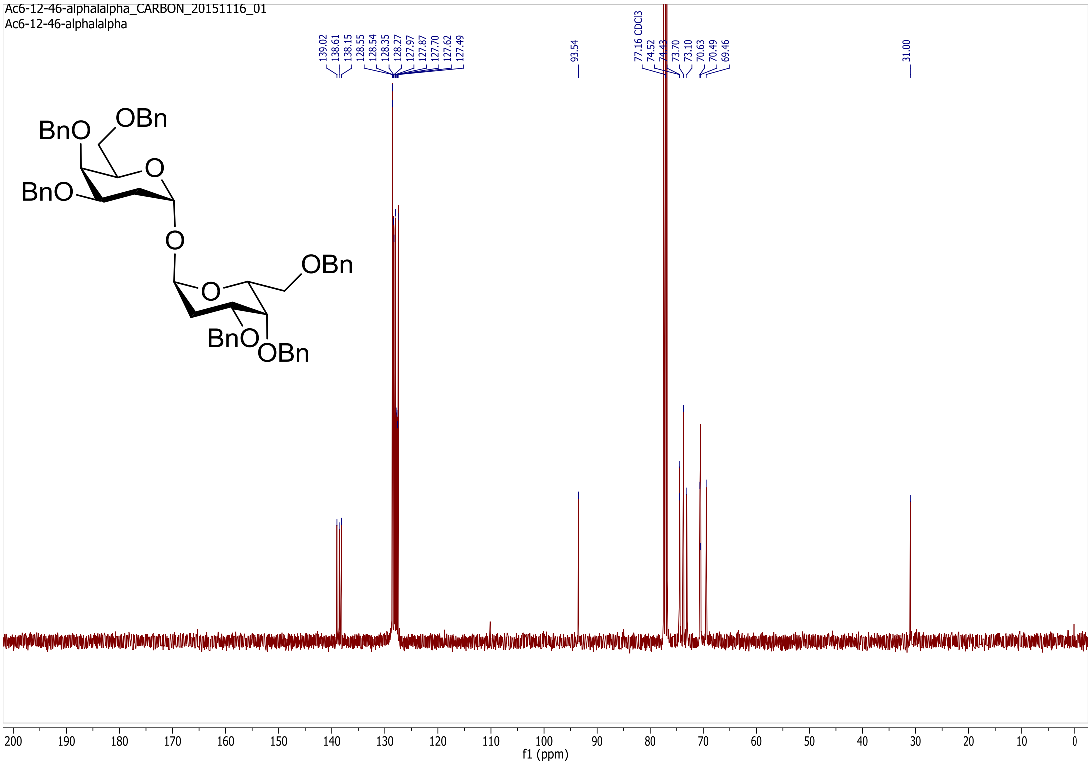

COSY (400 MHz, CDCl$_3$), 3,4,6-tri-*O*-benzyl-α-D-lyxo-hexapyranosyl-(1→1')-3',4',6'-tri-*O*-benzyl-α-D-lyxo-hexapyranoside α,α-9a

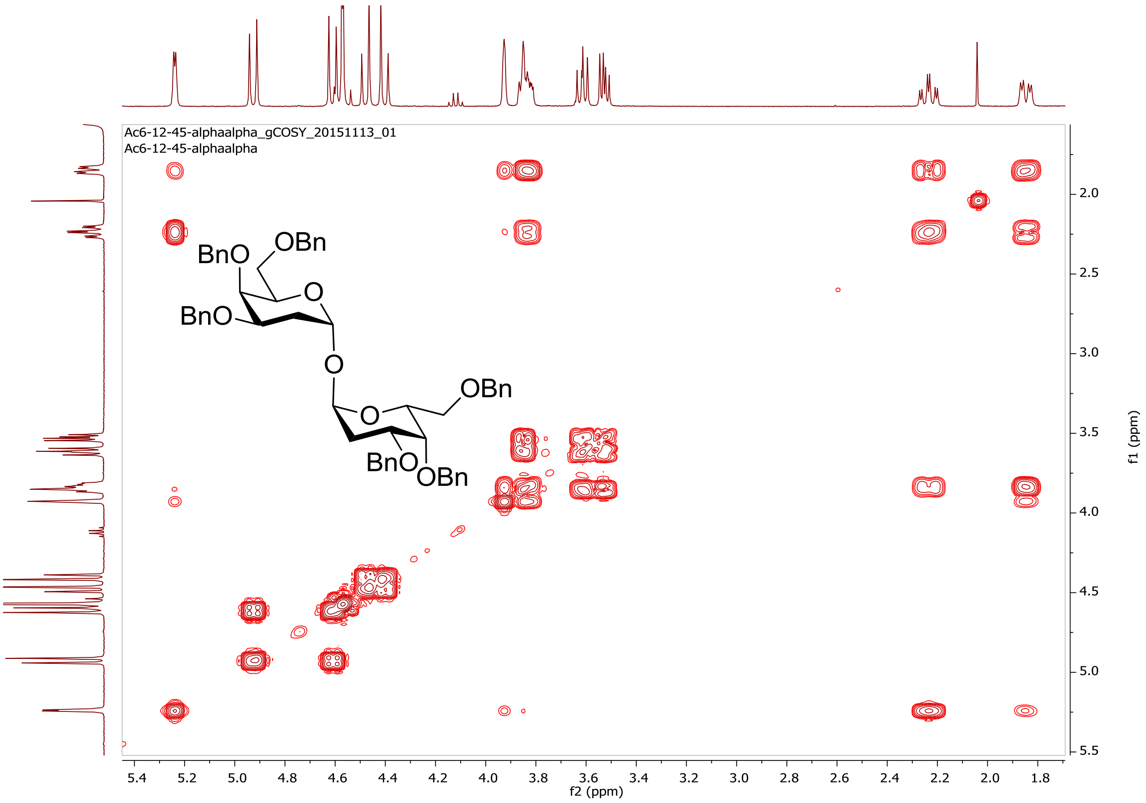

gHSQC (400 × 101 MHz, CDCl$_3$), 3,4,6-tri-*O*-benzyl-α-D-lyxo-hexapyranosyl-(1→1')-3',4',6'-tri-*O*-benzyl-α-D-lyxo-hexapyranoside α,α-9a

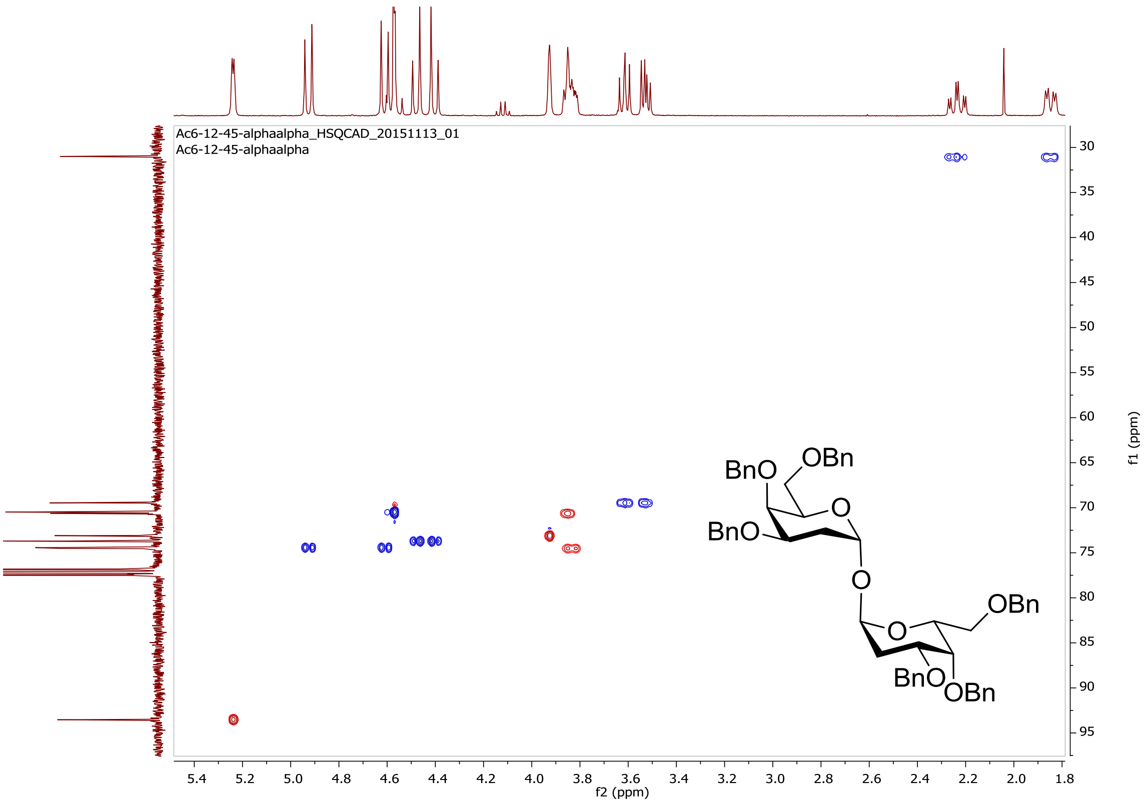

gHMBC; (400 × 101 MHz, $CDCl_3$), 3,4,6-tri-*O*-benzyl-α-D-lyxo-hexapyranosyl-(1→1')-3',4',6'-tri-*O*-benzyl-α-D-lyxo-hexapyranoside α,α-9a

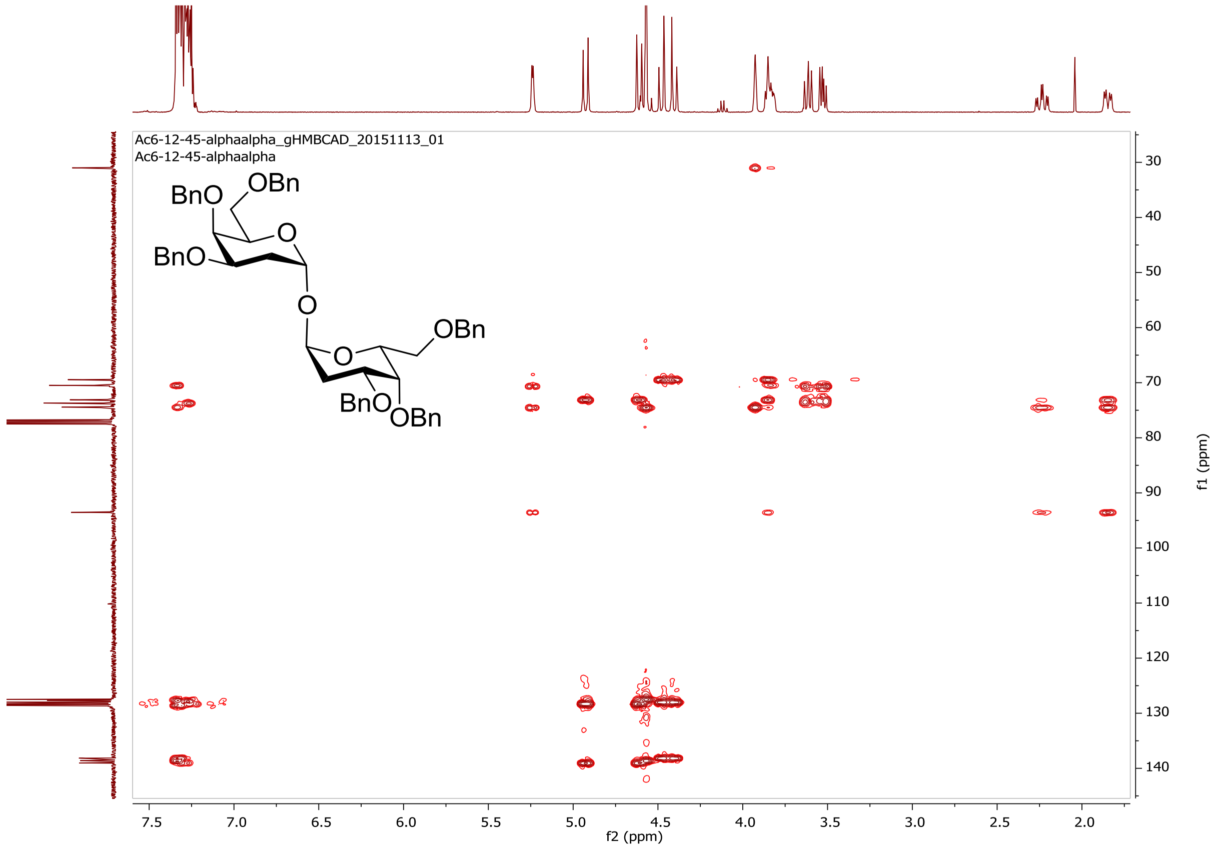

^{1}H NMR(400 MHz, CDCl$_3$), 3,4,6-tri-*O*-benzyl-α-D-lyxo-hexapyranosyl-(1→1')-3',4',6'-tri-*O*-benzyl-β-D-lyxo-hexapyranoside α,β-9a

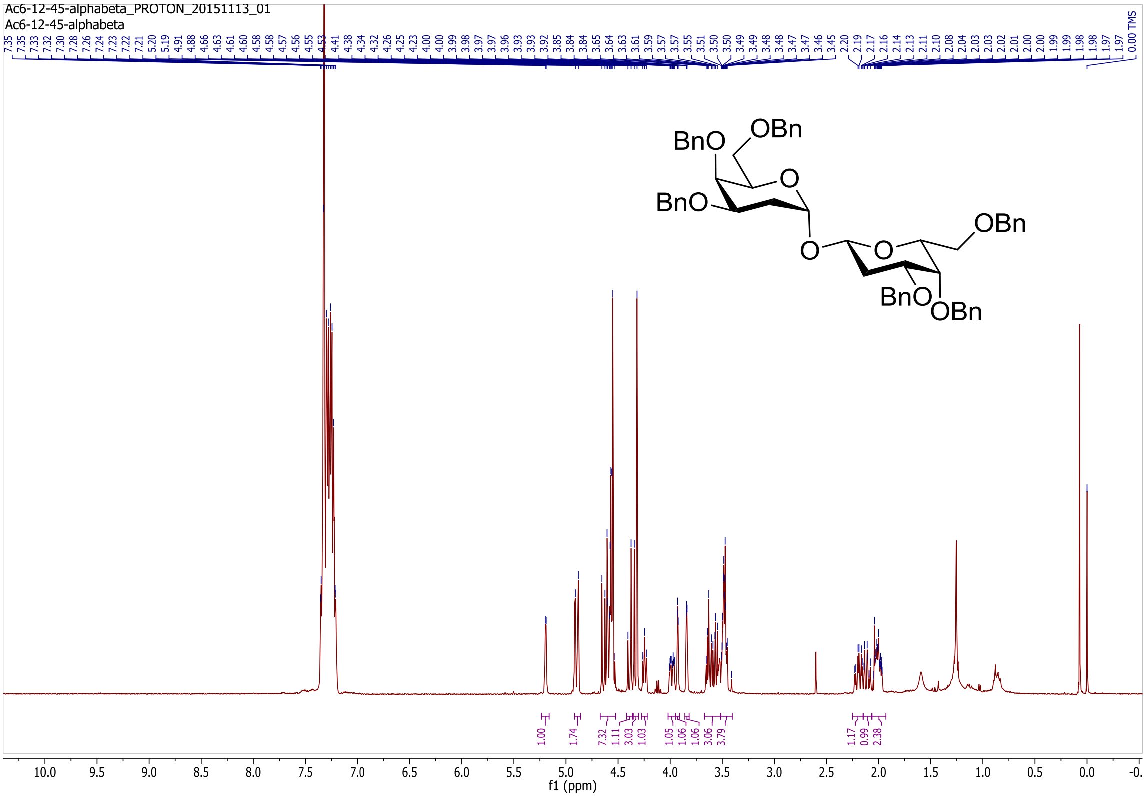

^{13}C NMR (101 MHz, CDCl$_3$), 3,4,6-tri-*O*-benzyl-α-D-lyxo-hexapyranosyl-(1→1')-3',4',6'-tri-*O*-benzyl-β-D-lyxo-hexapyranoside α,β-9a

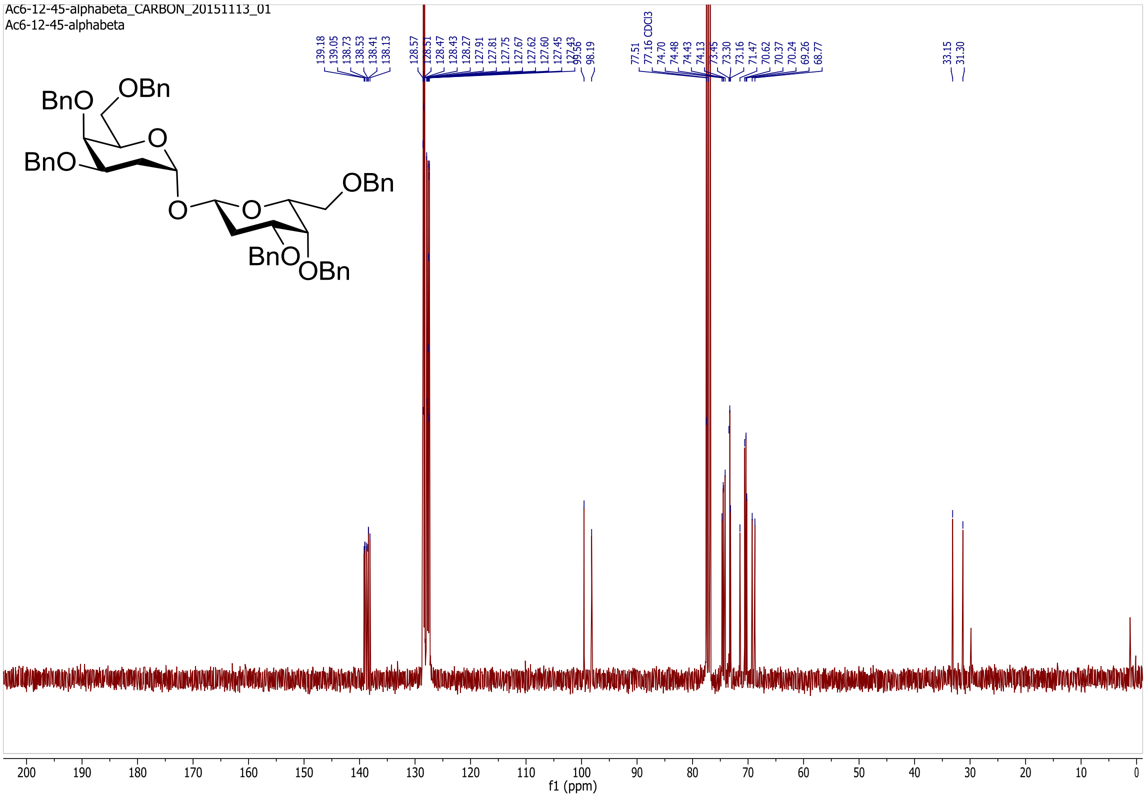

COSY (400 MHz, $CDCl_3$), 3,4,6-tri-*O*-benzyl-α-D-lyxo-hexapyranosyl-(1→1')-3',4',6'-tri-*O*-benzyl-β-D-lyxo-hexapyranoside α,β-9a

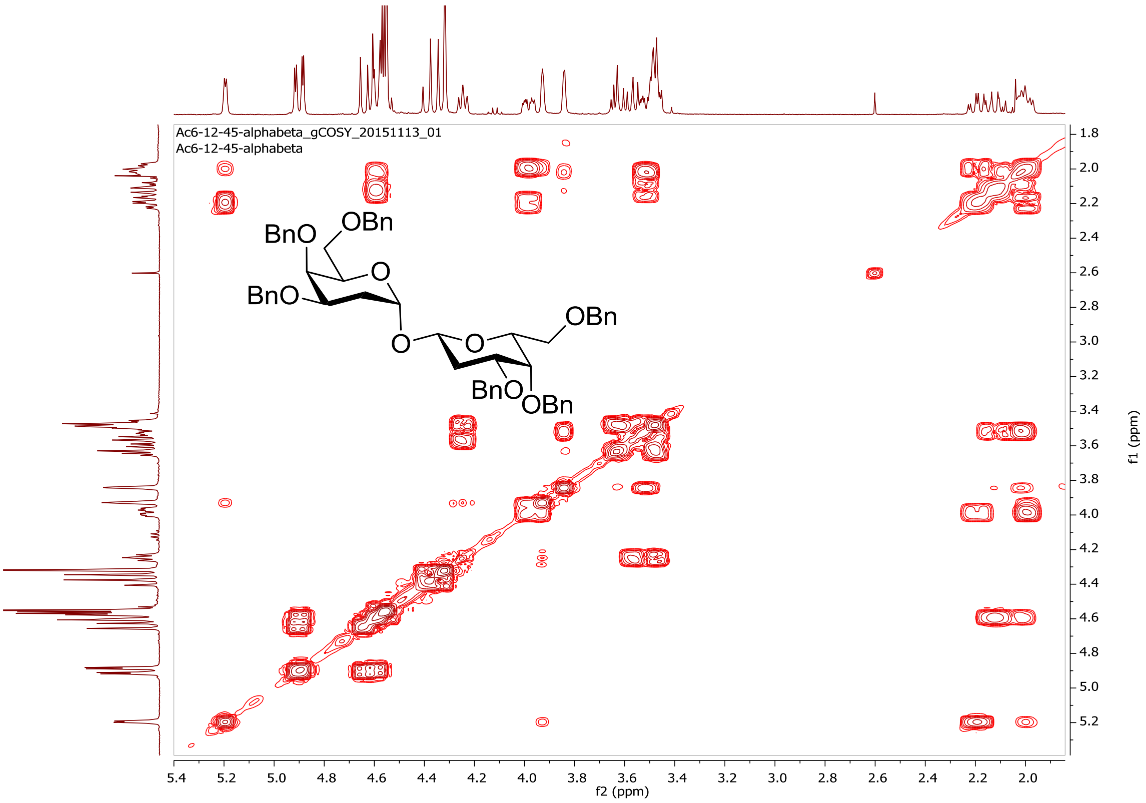

gHSQC (400 × 101 MHz, $CDCl_3$), 3,4,6-tri-*O*-benzyl-α-D-lyxo-hexapyranosyl-(1→1')-3',4',6'-tri-*O*-benzyl-β-D-lyxo-hexapyranoside α,β-9a

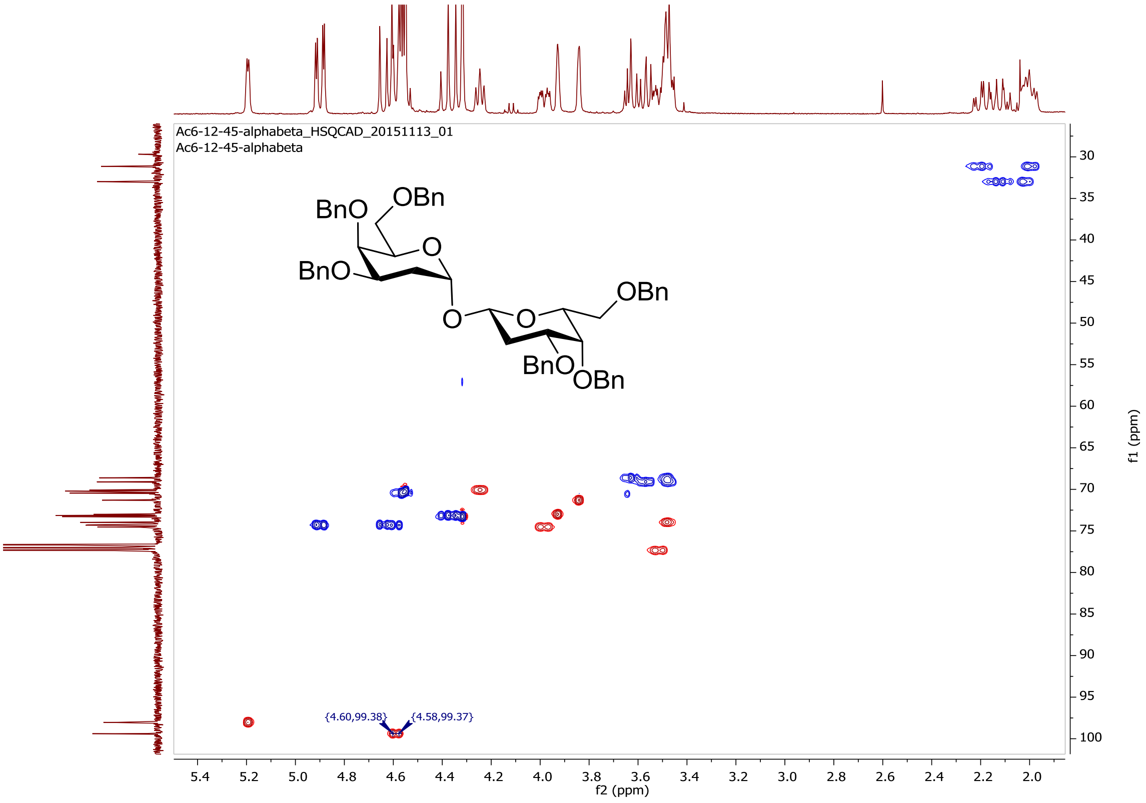

gHMBC (400 × 101 MHz, CDCl$_3$), 3,4,6-tri-*O*-benzyl-α-D-lyxo-hexapyranosyl-(1→1')-3',4',6'-tri-*O*-benzyl-β-D-lyxo-hexapyranoside α,β-9a

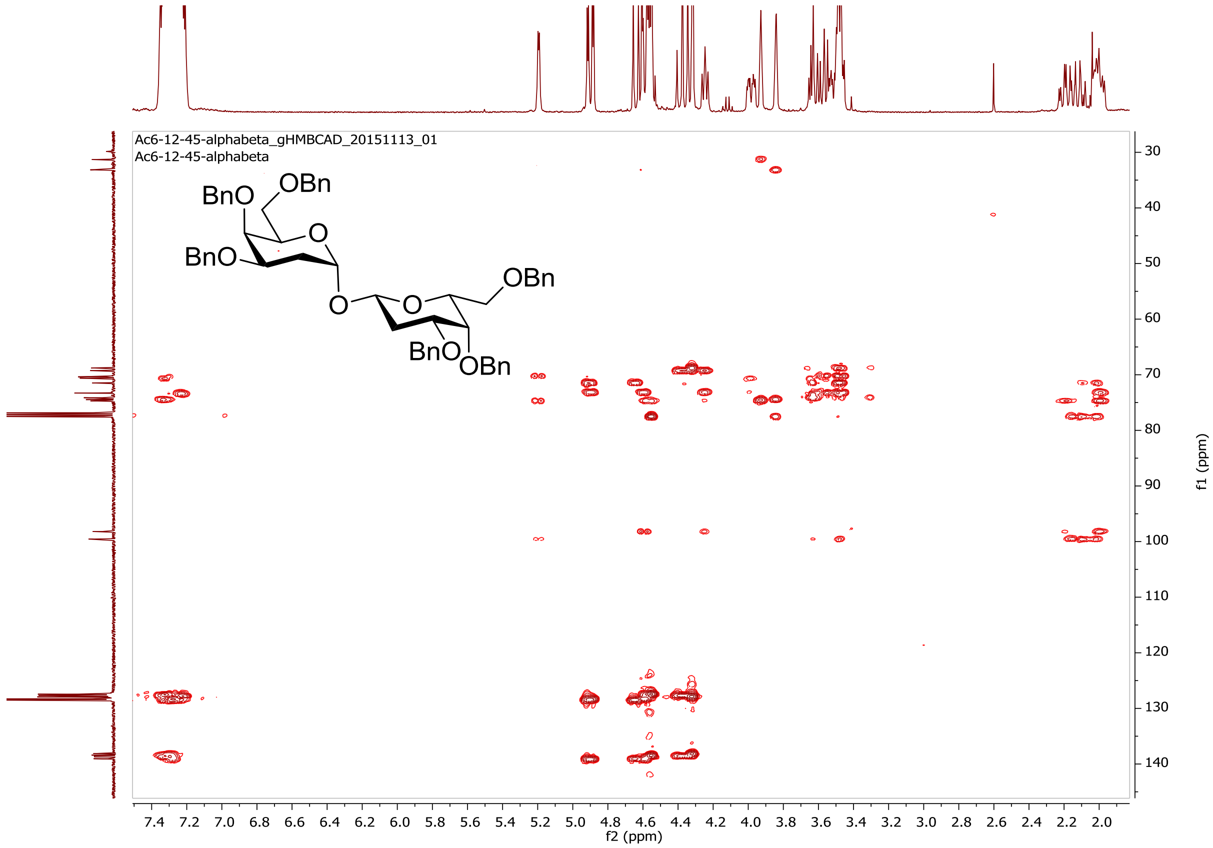

^{1}H NMR (400 MHz,$CDCl_3$), 2,3,4,6-Tetra-*O*-benzyl-α-D-glucopyranosyl-(1→1')-3',4',6'-tri-*O*-benzyl-α-D-lyxo-hexapyranoside α,α-11a

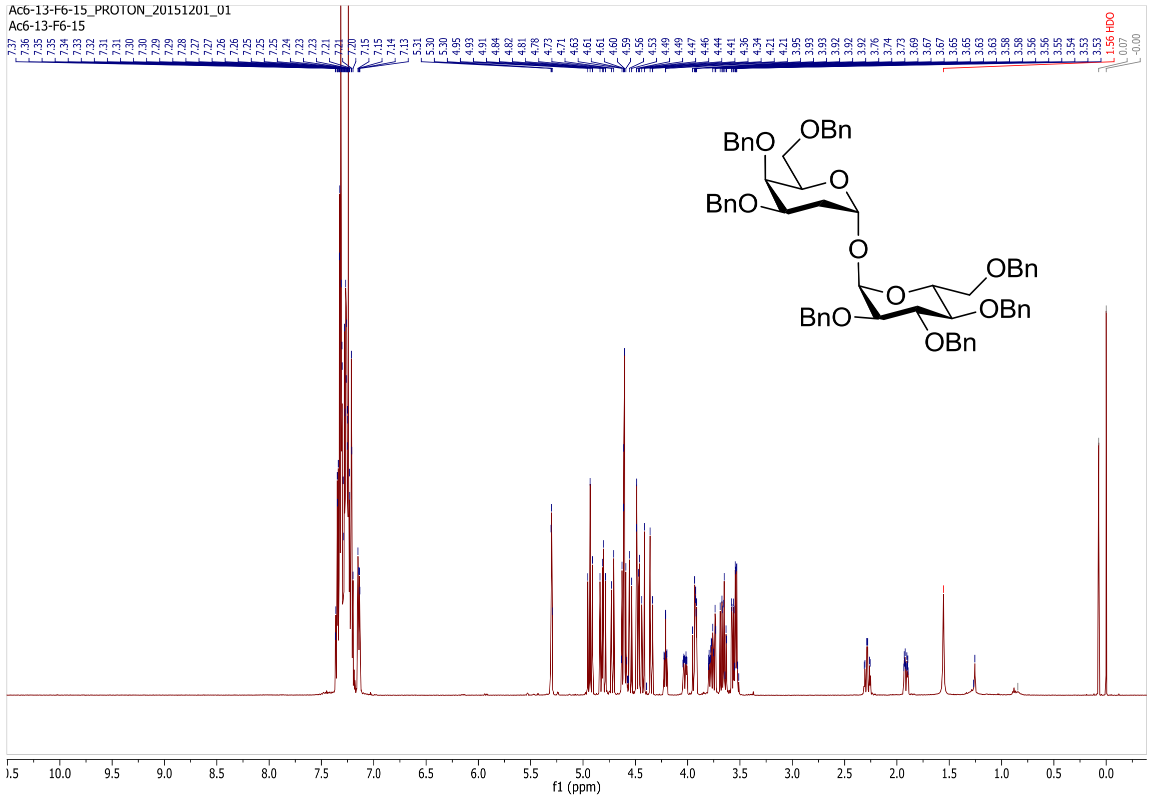

^{13}C NMR (101 MHz, $CDCl_3$), 2,3,4,6-Tetra-*O*-benzyl-α-D-glucopyranosyl-(1→1')-3',4',6'-tri-*O*-benzyl-α-D-lyxo-hexapyranoside α,α-11a

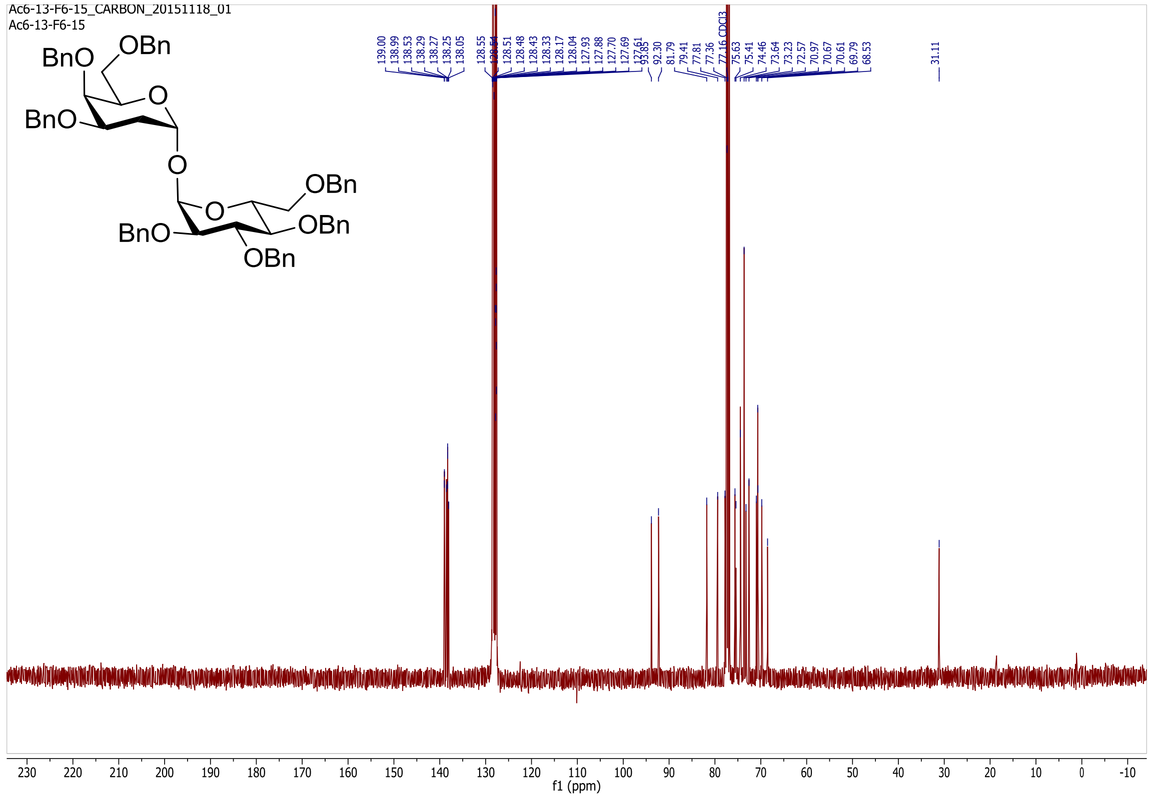

COSY (400 MHz, CDCl$_3$), 2,3,4,6-Tetra-*O*-benzyl-α-D-glucopyranosyl-(1→1')-3',4',6'-tri-*O*-benzyl-α-D-lyxo-hexapyranoside α,α-11a

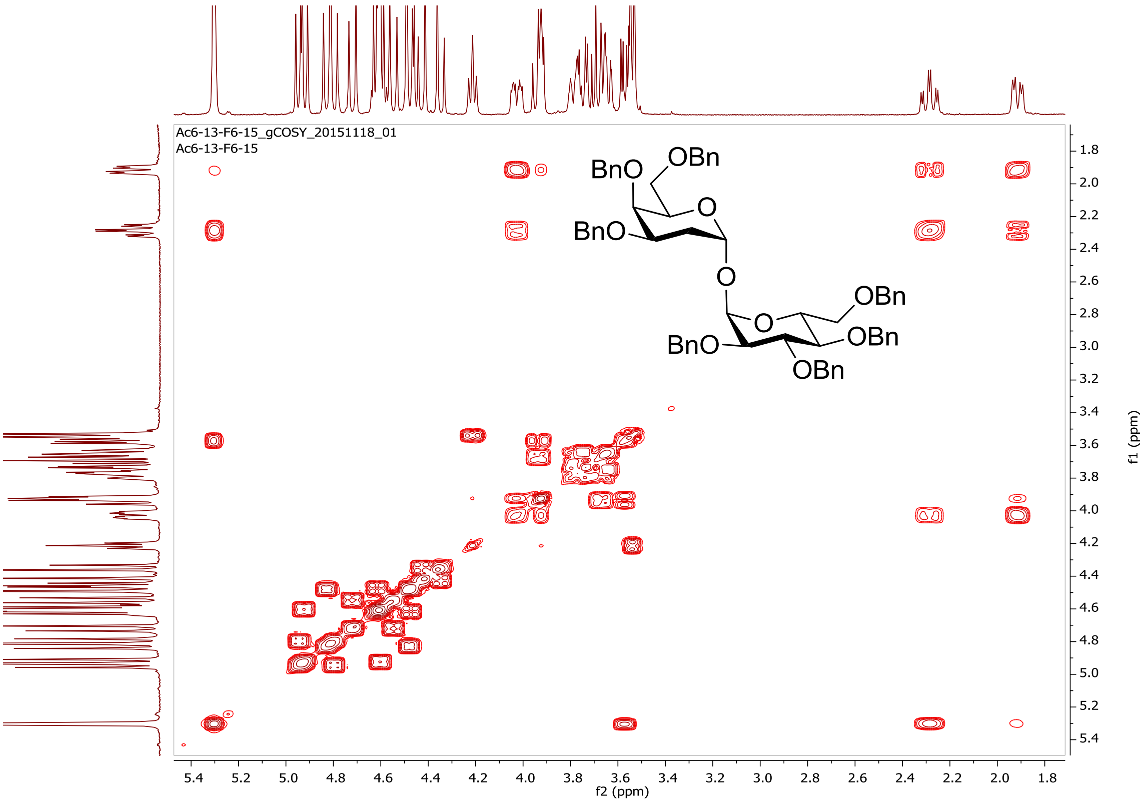

gHSQC; (400 × 101 MHz, CDCl$_3$), 2,3,4,6-Tetra-*O*-benzyl-α-D-glucopyranosyl-(1→1')-3',4',6'-tri-*O*-benzyl-α-D-lyxo-hexapyranoside α,α-11a

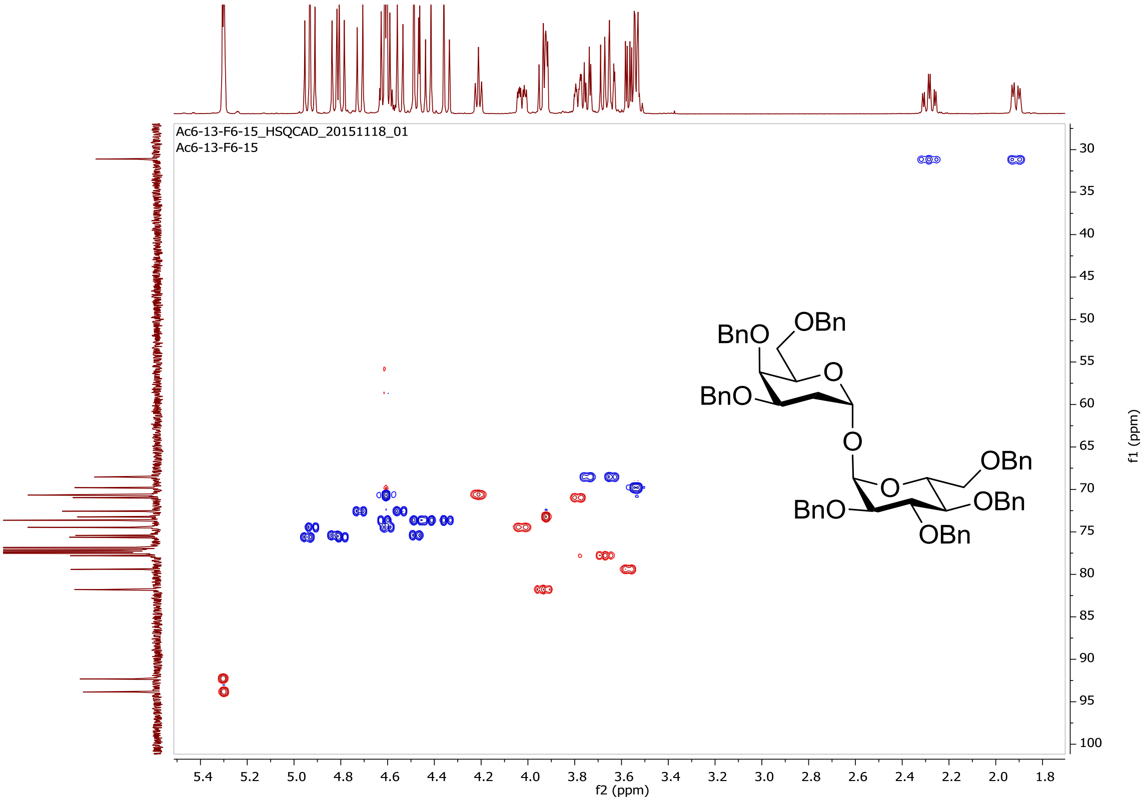

gHMBC (400 × 101 MHz, CDCl$_3$), 2,3,4,6-Tetra-*O*-benzyl-α-D-glucopyranosyl-(1→1')-3',4',6'-tri-*O*-benzyl-α-D-lyxo-hexapyranoside α,α-11a

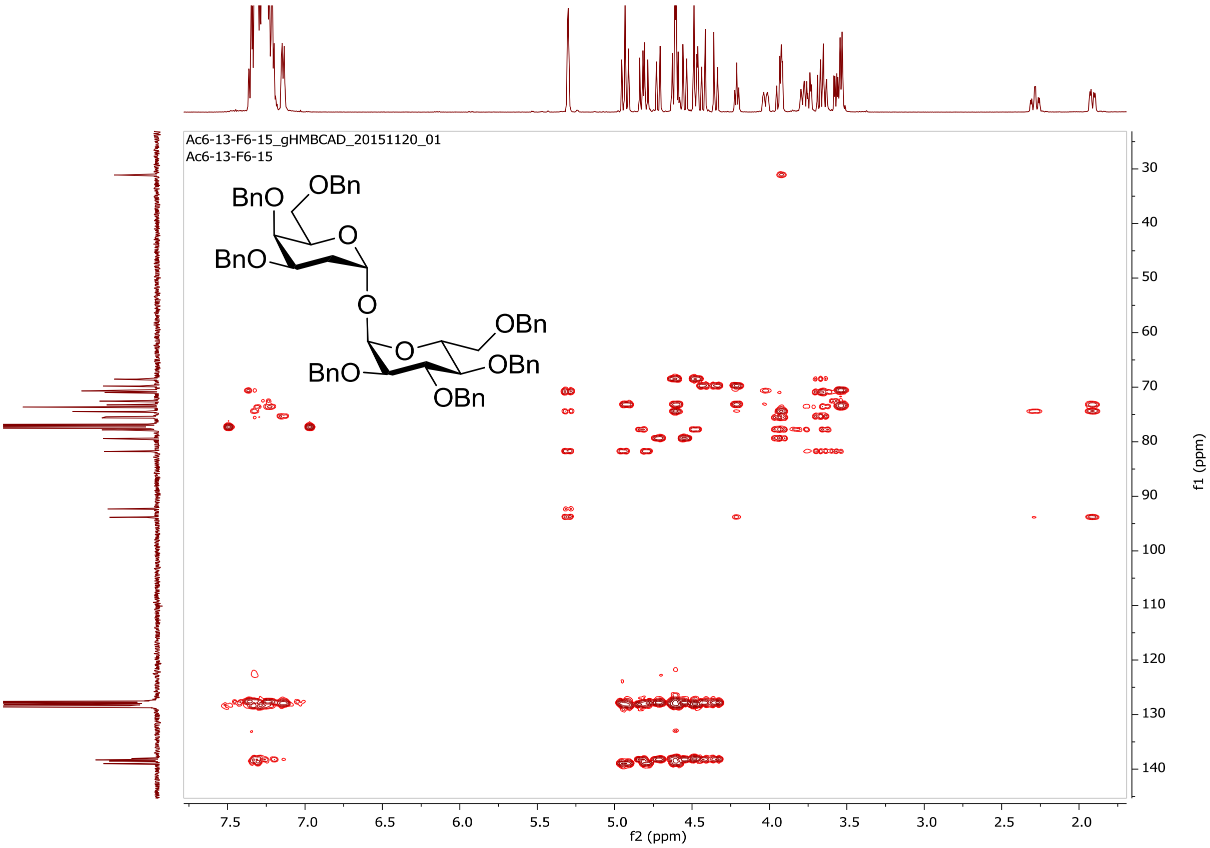

^{1}H NMR(400 MHz,CDCl$_3$), 2,3,4,6-Tetra-*O*-benzyl-β-D-glucopyranosyl-(1→1')-3',4',6'-tri-*O*-benzyl-α-D-lyxo-hexapyranoside α,β-11a

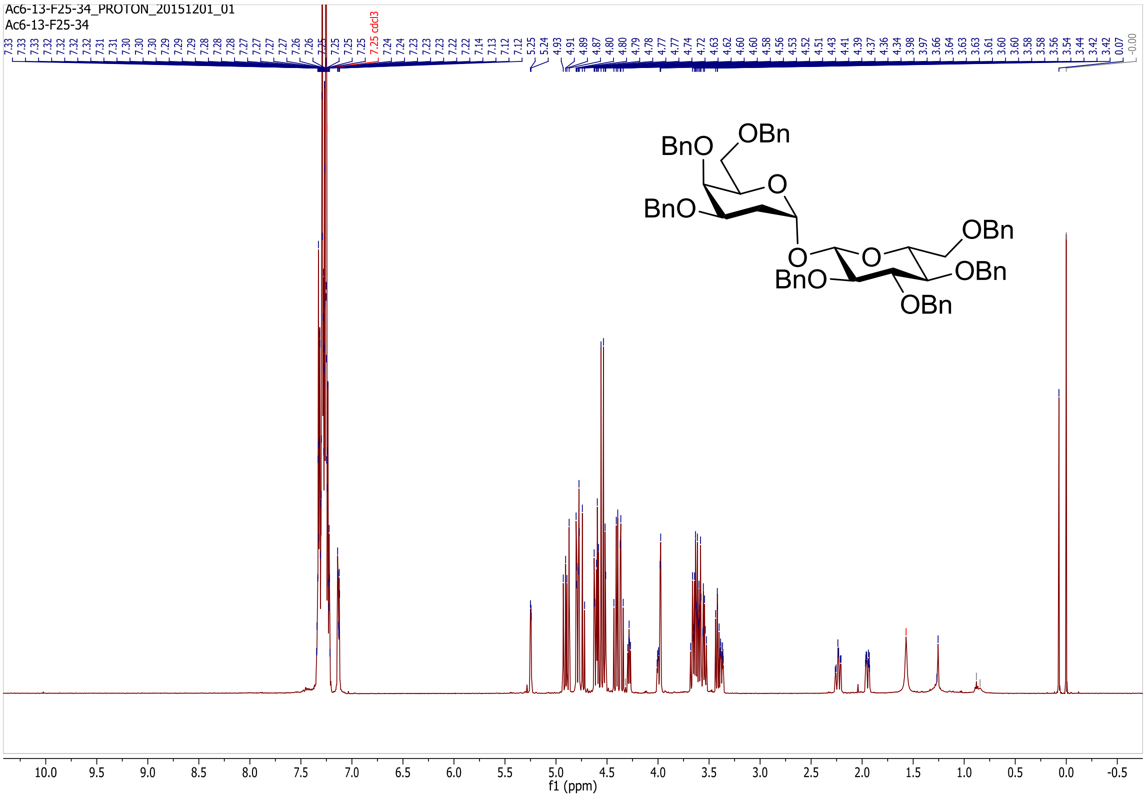

^{13}C NMR (101 MHz, CDCl$_3$), 2,3,4,6-Tetra-*O*-benzyl-β-D-glucopyranosyl-(1→1')-3',4',6'-tri-*O*-benzyl-α-D-lyxo-hexapyranoside α,β-11a

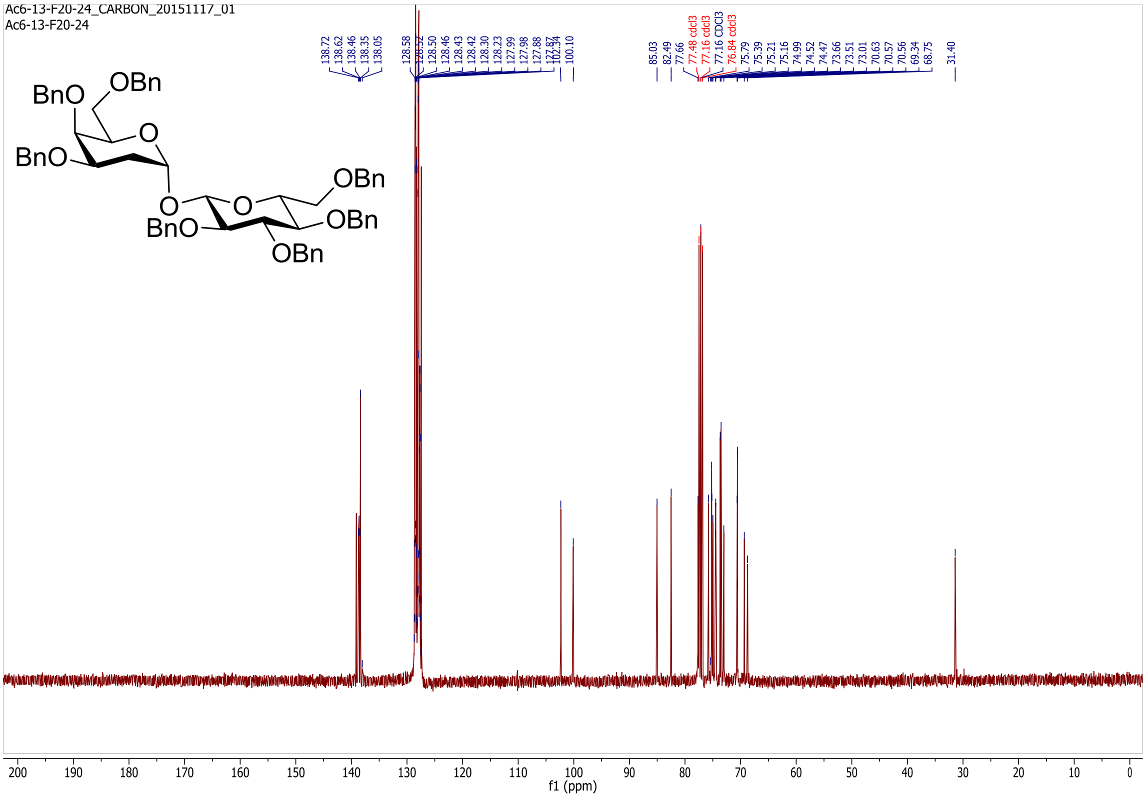

COSY (400 MHz, CDCl$_3$), 2,3,4,6-Tetra-*O*-benzyl-β-D-glucopyranosyl-(1→1')-3',4',6'-tri-*O*-benzyl-α-D-lyxo-hexapyranoside α,β-11a

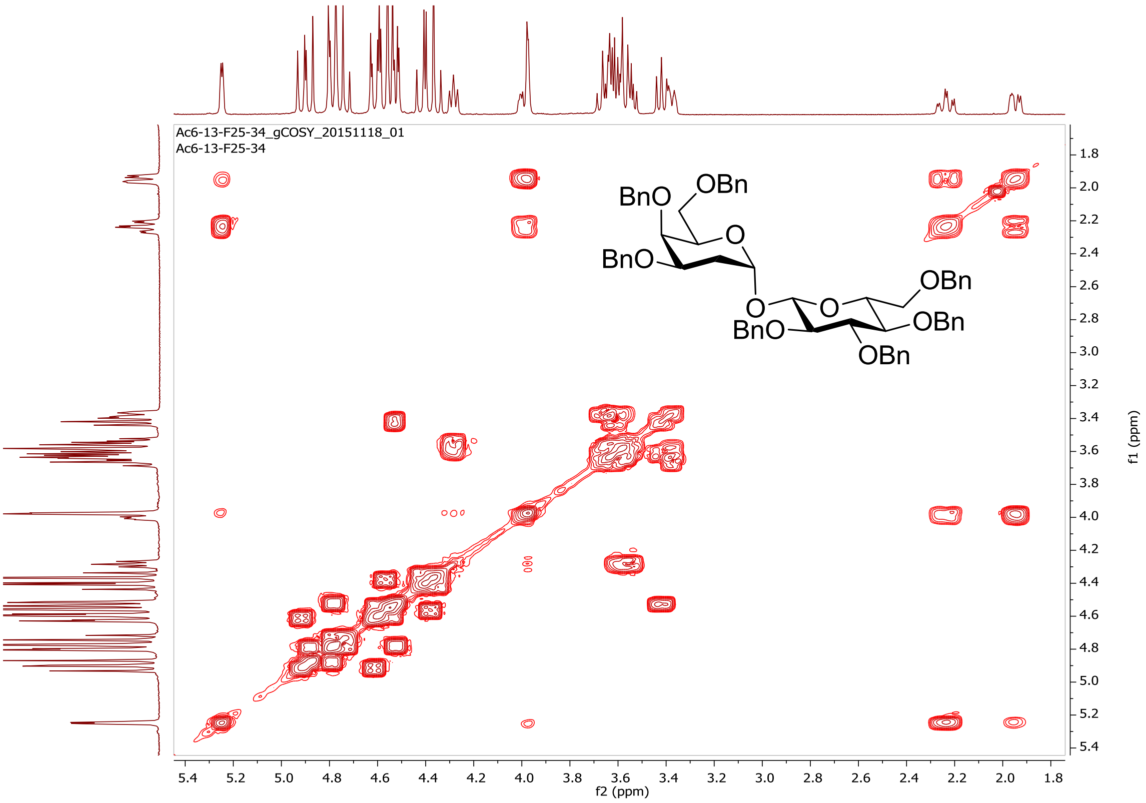

gHSQC (400 × 101 MHz, CDCl$_3$), 2,3,4,6-Tetra-*O*-benzyl-β-D-glucopyranosyl-(1→1')-3',4',6'-tri-*O*-benzyl-α-D-lyxo-hexapyranoside α,β-11a

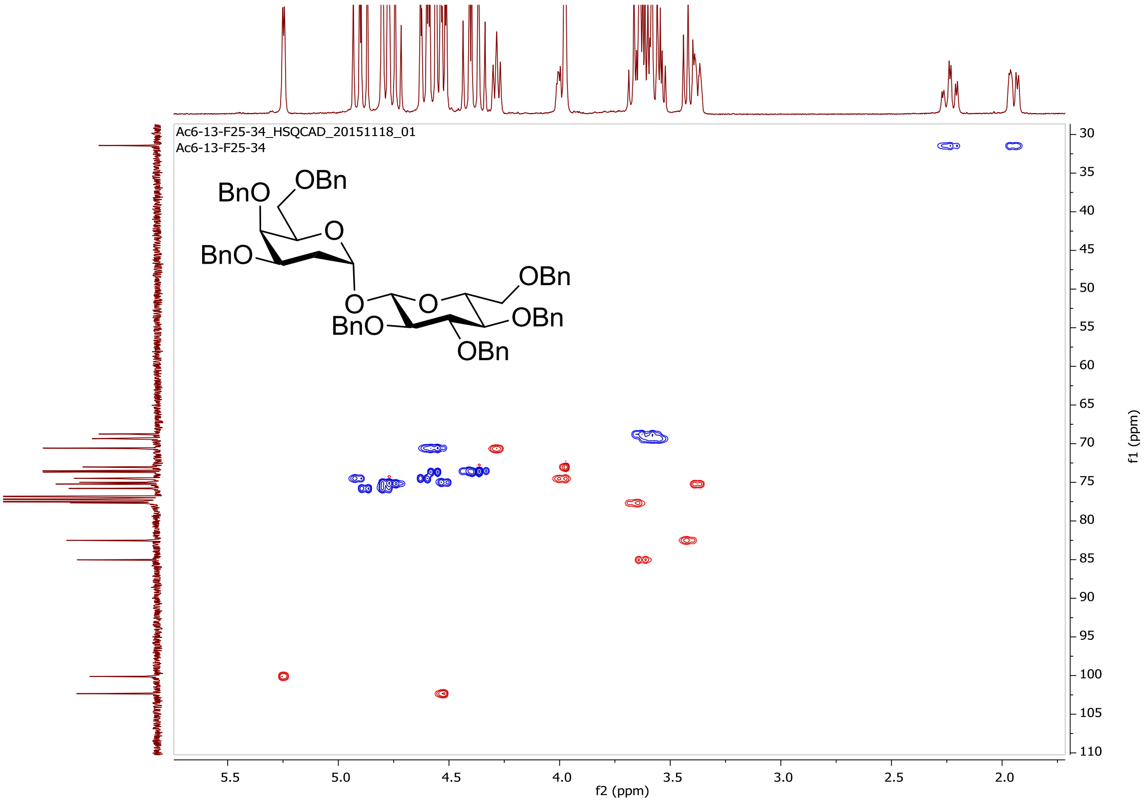

gHMBC; (400 × 101 MHz, CDCl$_3$), 2,3,4,6-Tetra-*O*-benzyl-β-D-glucopyranosyl-(1→1')-3',4',6'-tri-*O*-benzyl-α-D-lyxo-hexapyranoside α,β-11a

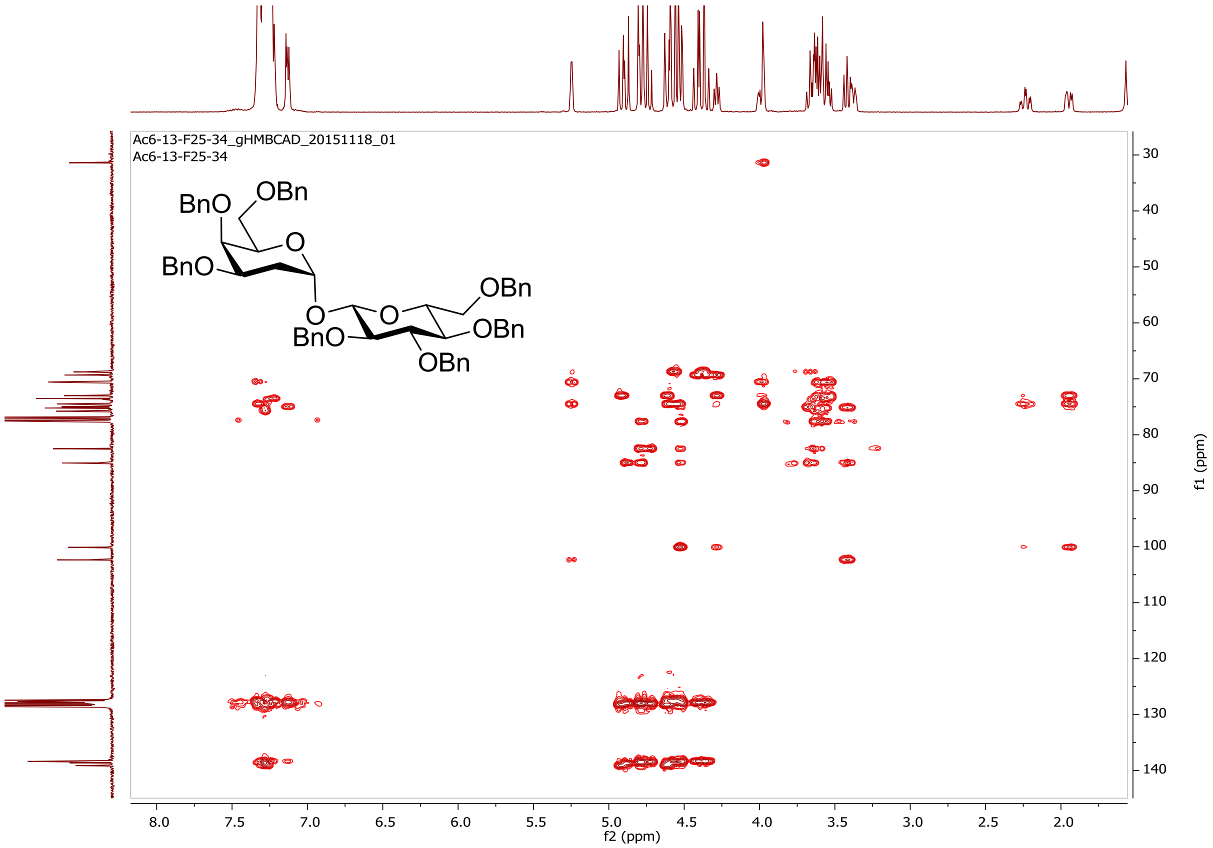

NMR Spectra of Deprotected sugars

^{1}H NMR (400 MHz, CD_3OD), 2,2'-dideoxy-lyxo-trehalose α,α-9b

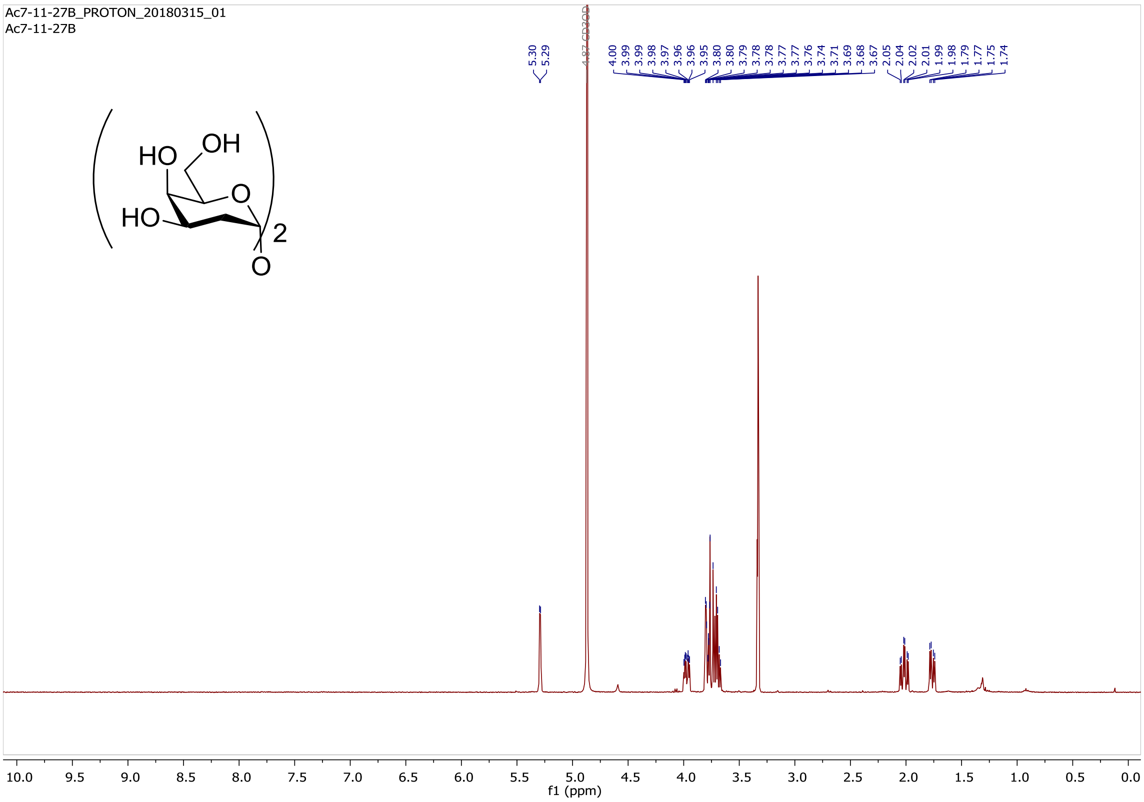

^{13}C NMR (101 MHz, CD_3OD), 2,2'-dideoxy-lyxo-trehalose α,α-9b

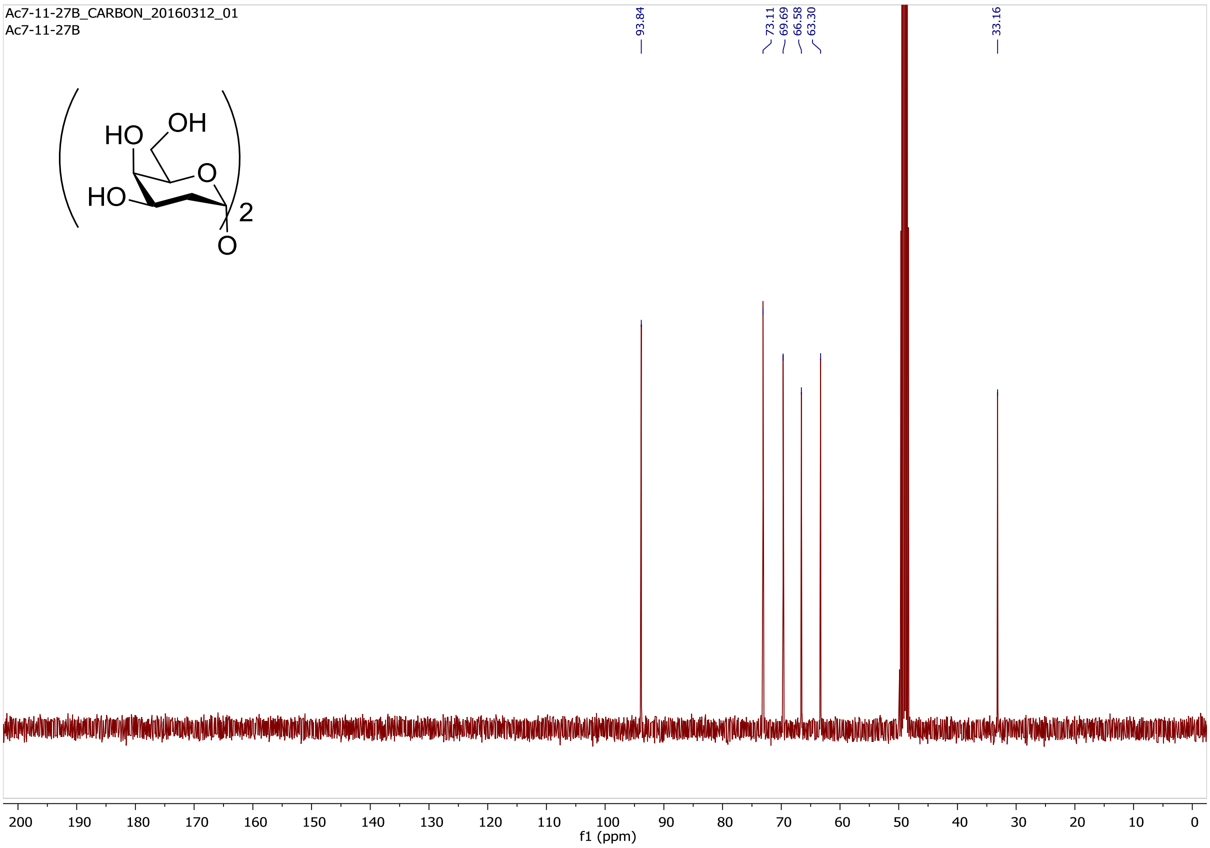

COSY (400 MHz, CD_3OD), 2,2'-dideoxy-lyxo-trehalose α,α-9b

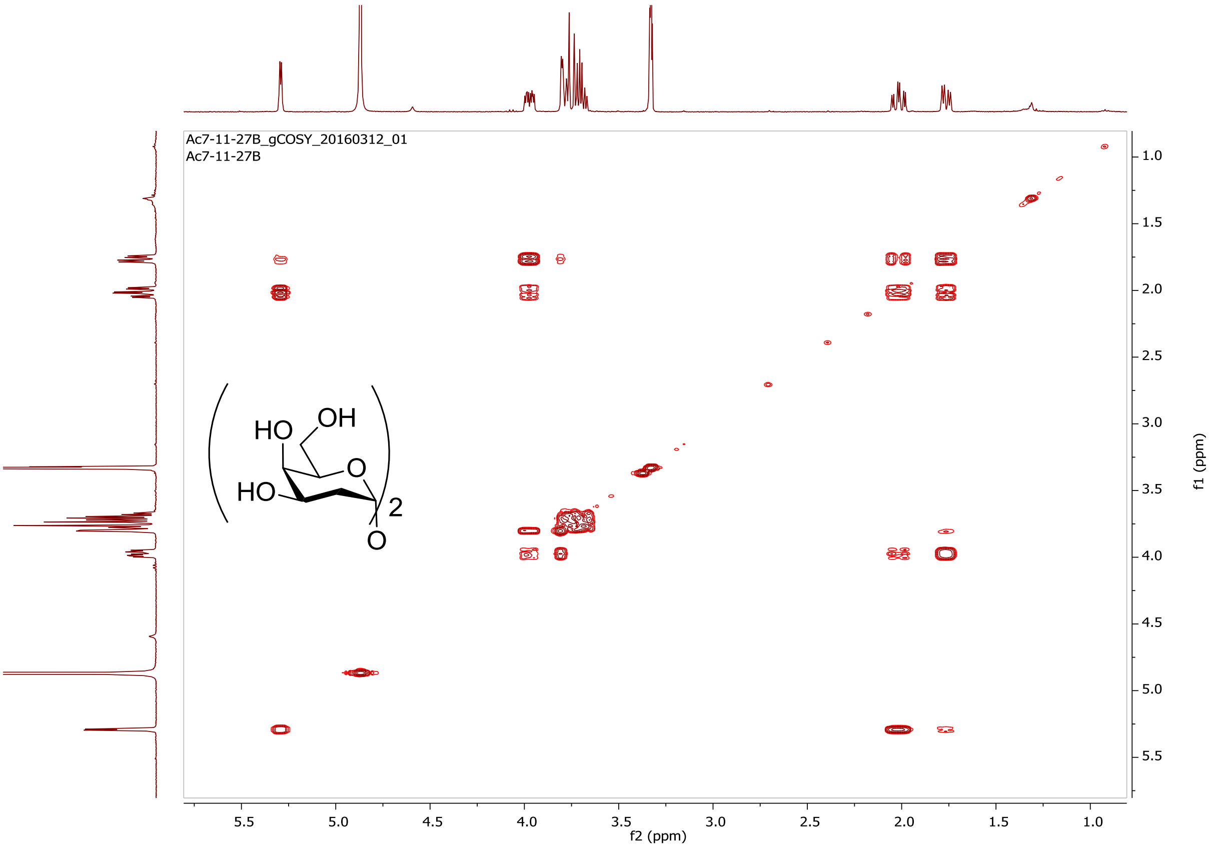

gHSQC (400 × 100 MHz, CD_3OD), 2,2'-dideoxy-lyxo-trehalose α,α-9b

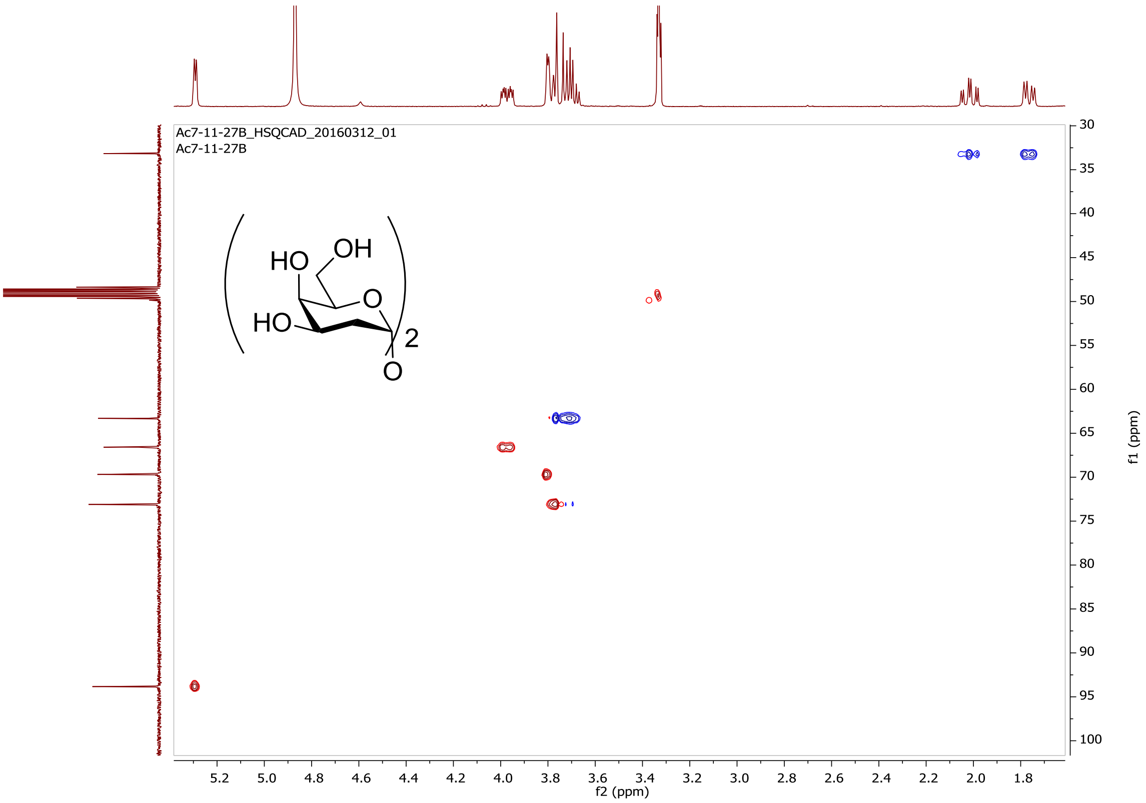

gHMBC (400 × 100 MHz, CD_3OD), 2,2’-dideoxy-lyxo-trehalose α,α-9b

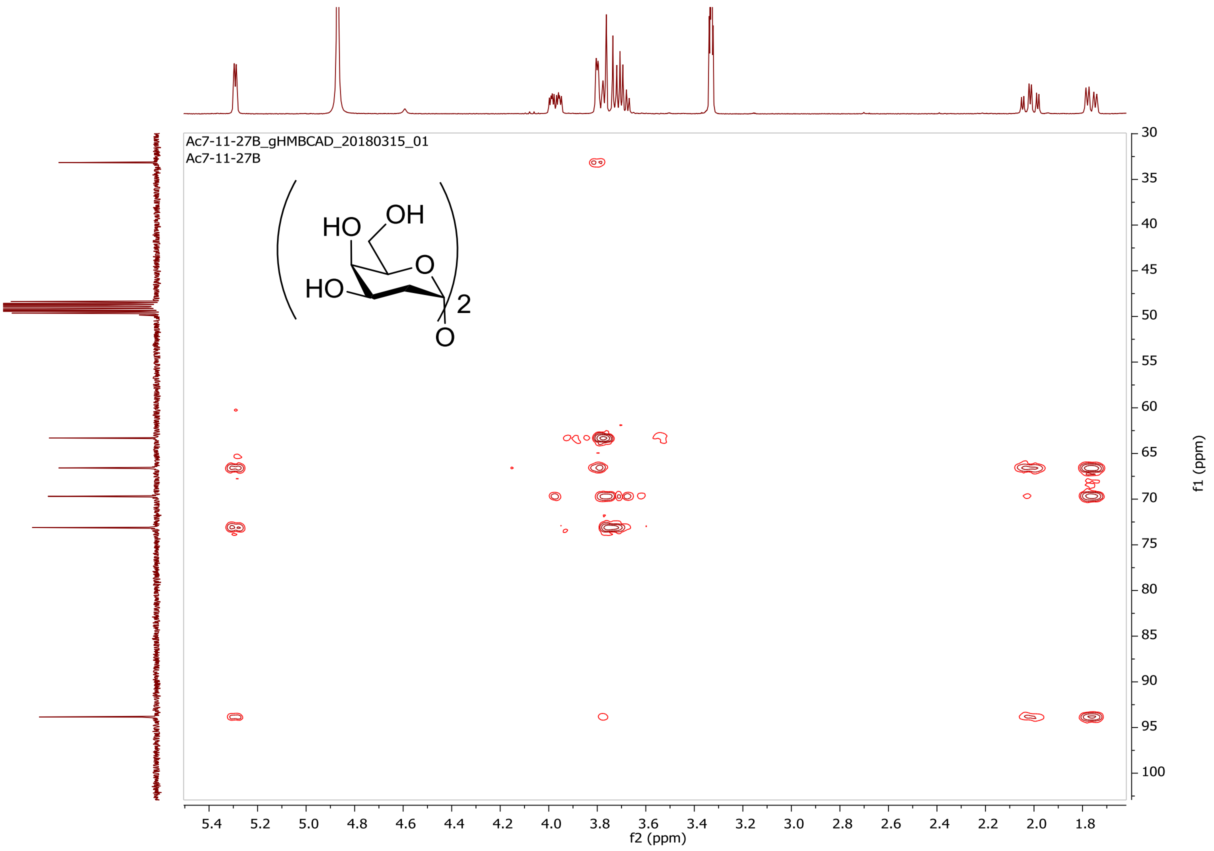

^{1}H NMR (500 MHz, CD$_3$OD), α-D-glucopyranosyl-(1→1')-α-D-lyxo-hexapyranoside α,α-11b

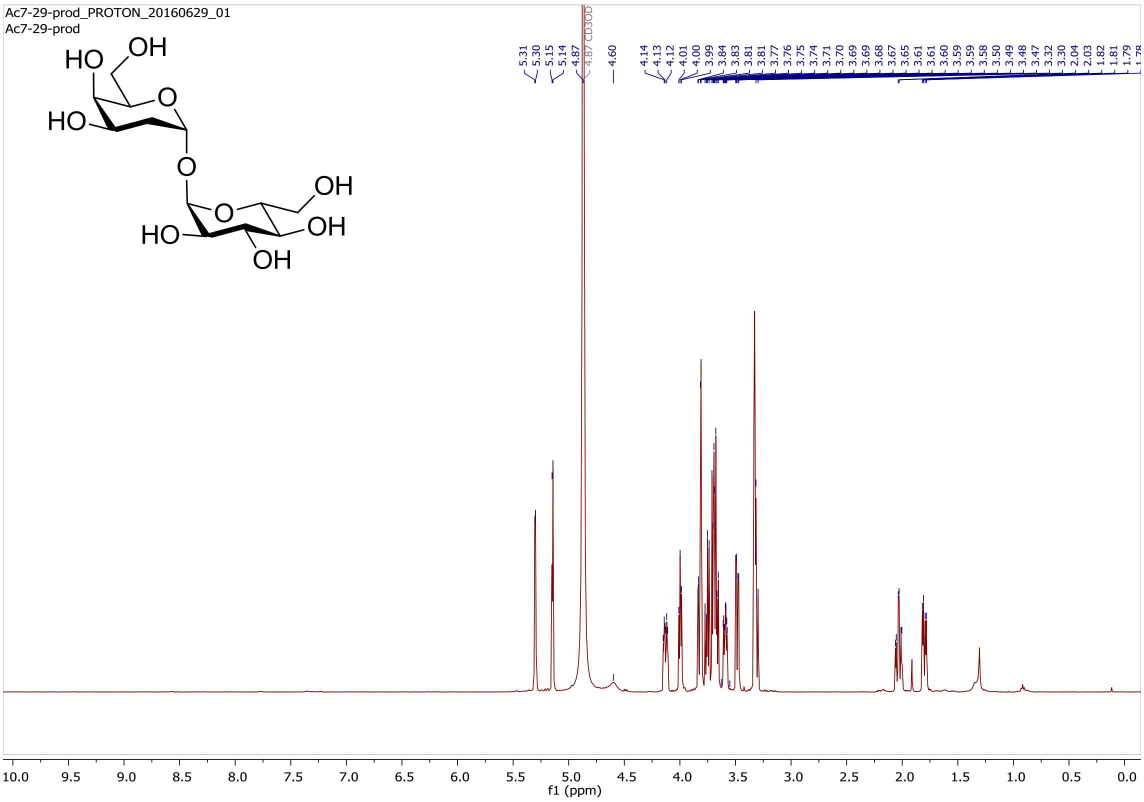

^{13}C NMR (126 MHz, CD$_3$OD), α-D-glucopyranosyl-(1→1')-α-D-lyxo-hexapyranoside α,α-11b

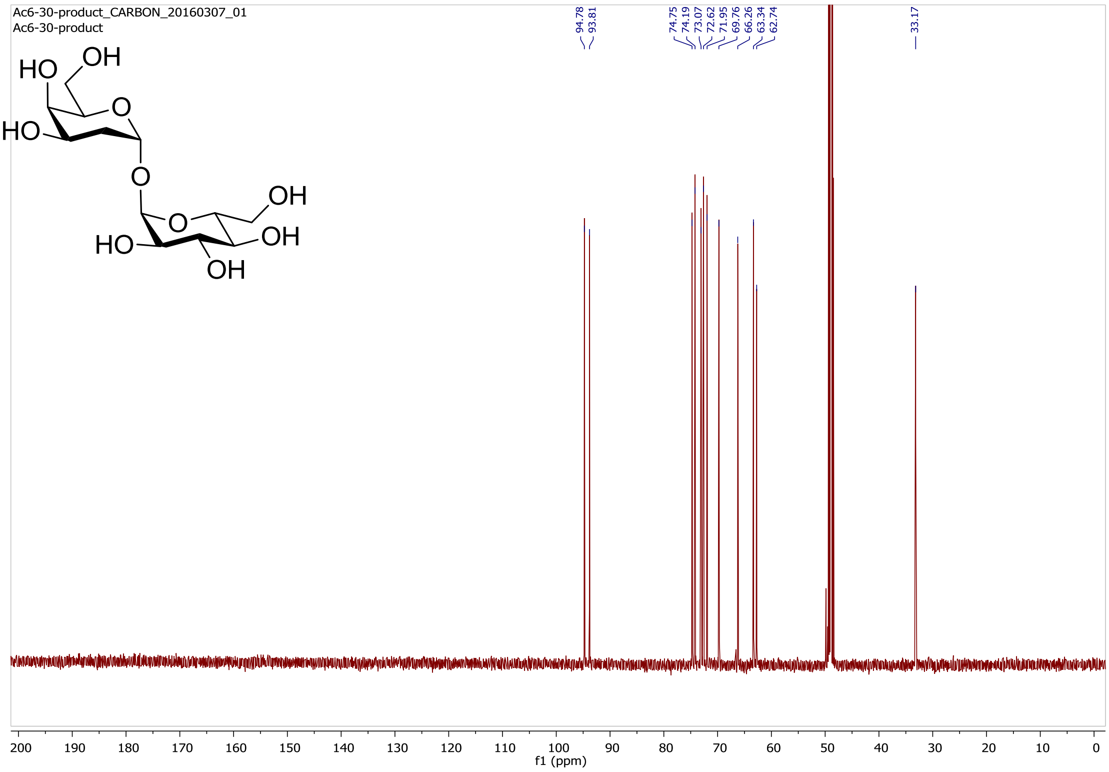

COSY (500 MHz, CD$_3$OD), α-D-glucopyranosyl-(1→1')-α-D-lyxo-hexapyranoside α,α-11b

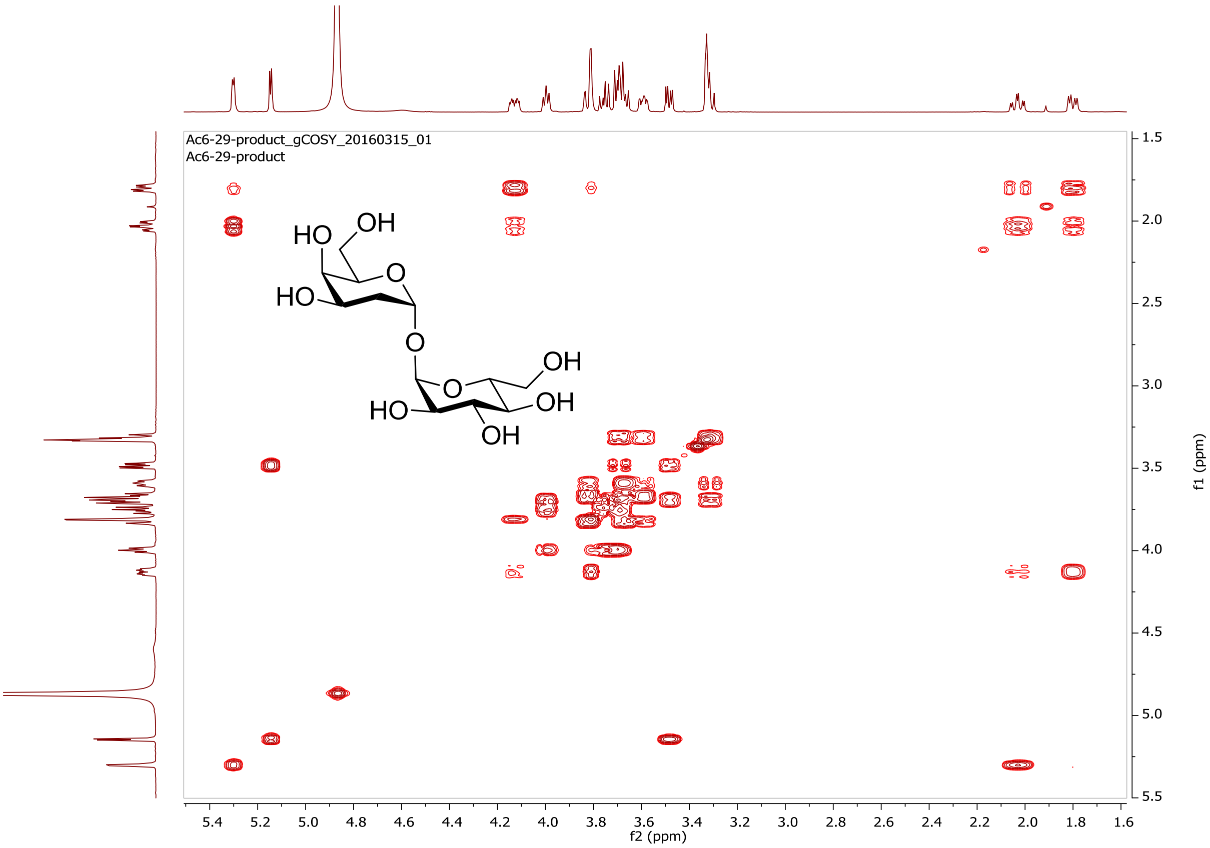

gHSQC (500 × 126 MHz, CD$_3$OD), α-D-glucopyranosyl-(1→1')-α-D-lyxo-hexapyranoside α,α-11b

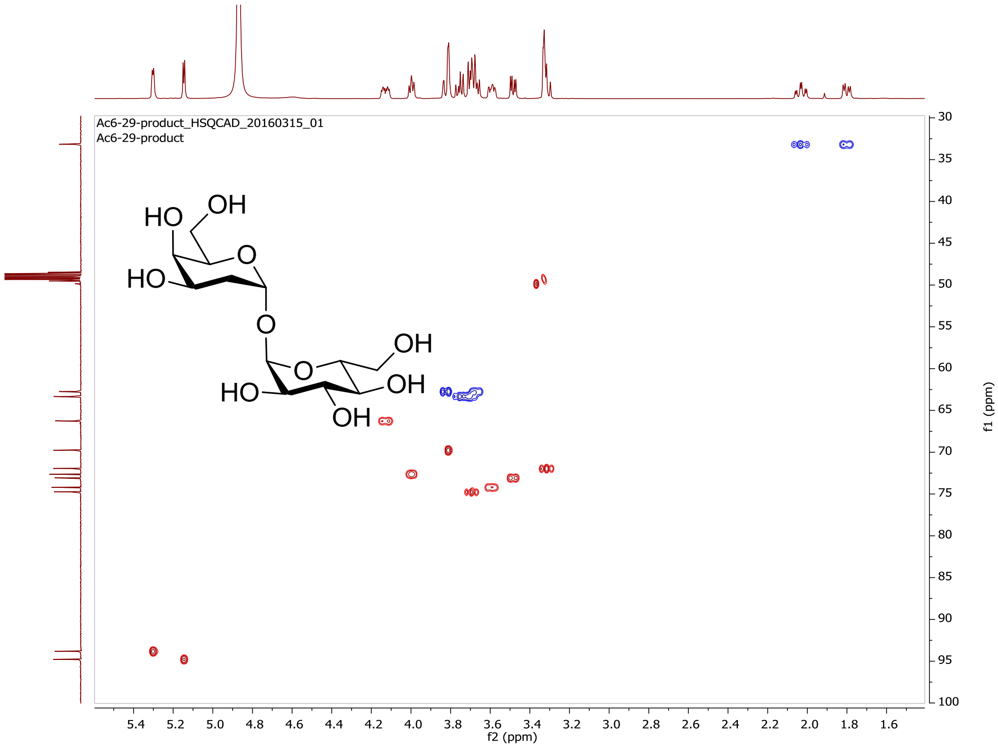

gHMBC (500 × 126 MHz, CD_3OD), α-D-glucopyranosyl-(1→1')-α-D-lyxo-hexapyranoside α,α-11b

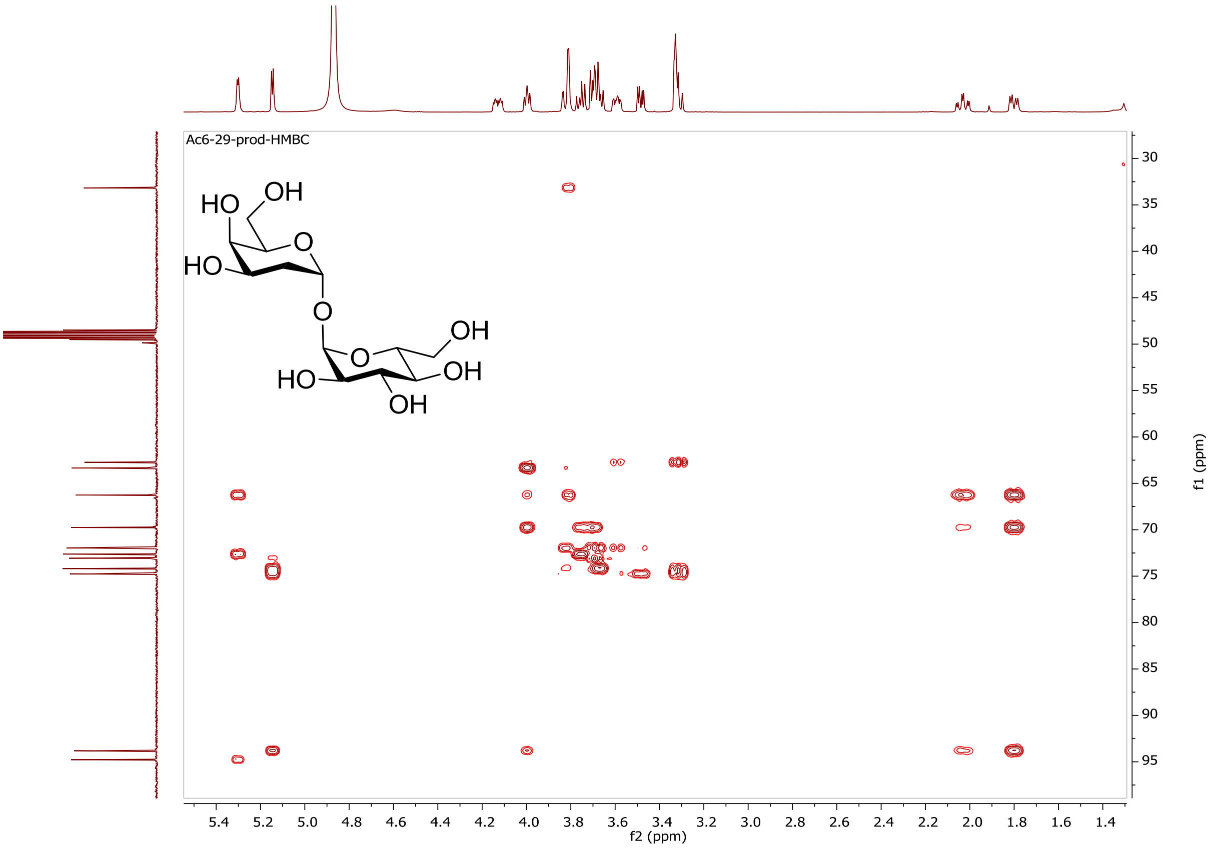

^{1}H NMR (500 MHz, CD_3OD), β-D-glucopyranosyl-(1→1')-α-D-lyxo-hexapyranoside α,β-11b

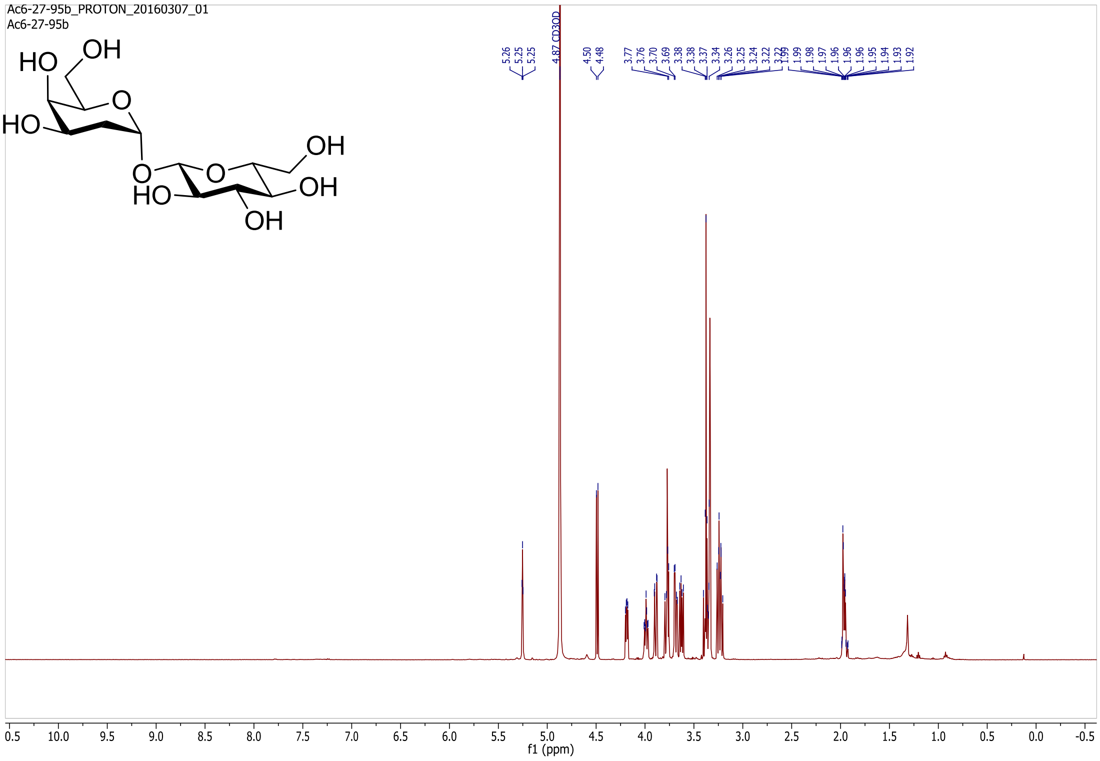

^{13}C NMR (126 MHz, CD_3OD), β-D-glucopyranosyl-(1→1')-α-D-lyxo-hexapyranoside α,β-11b

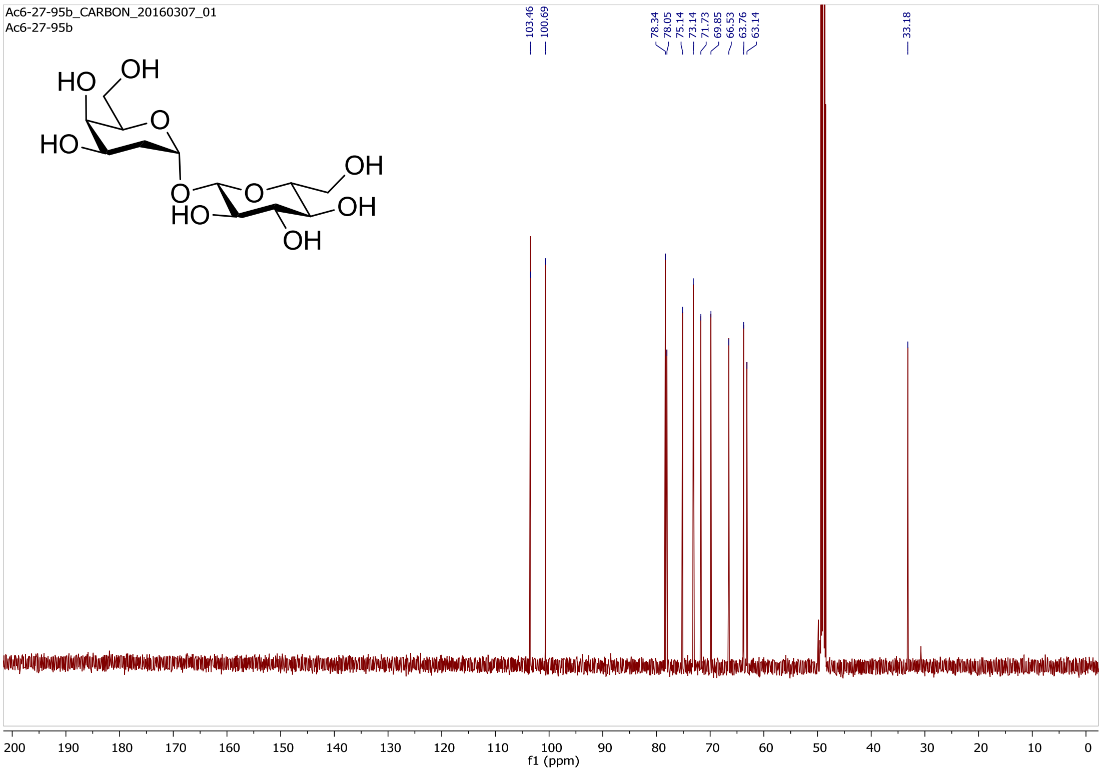

COSY (500 MHz, CD_3OD), β-D-glucopyranosyl-(1→1')-α-D-lyxo-hexapyranoside α,β-11b

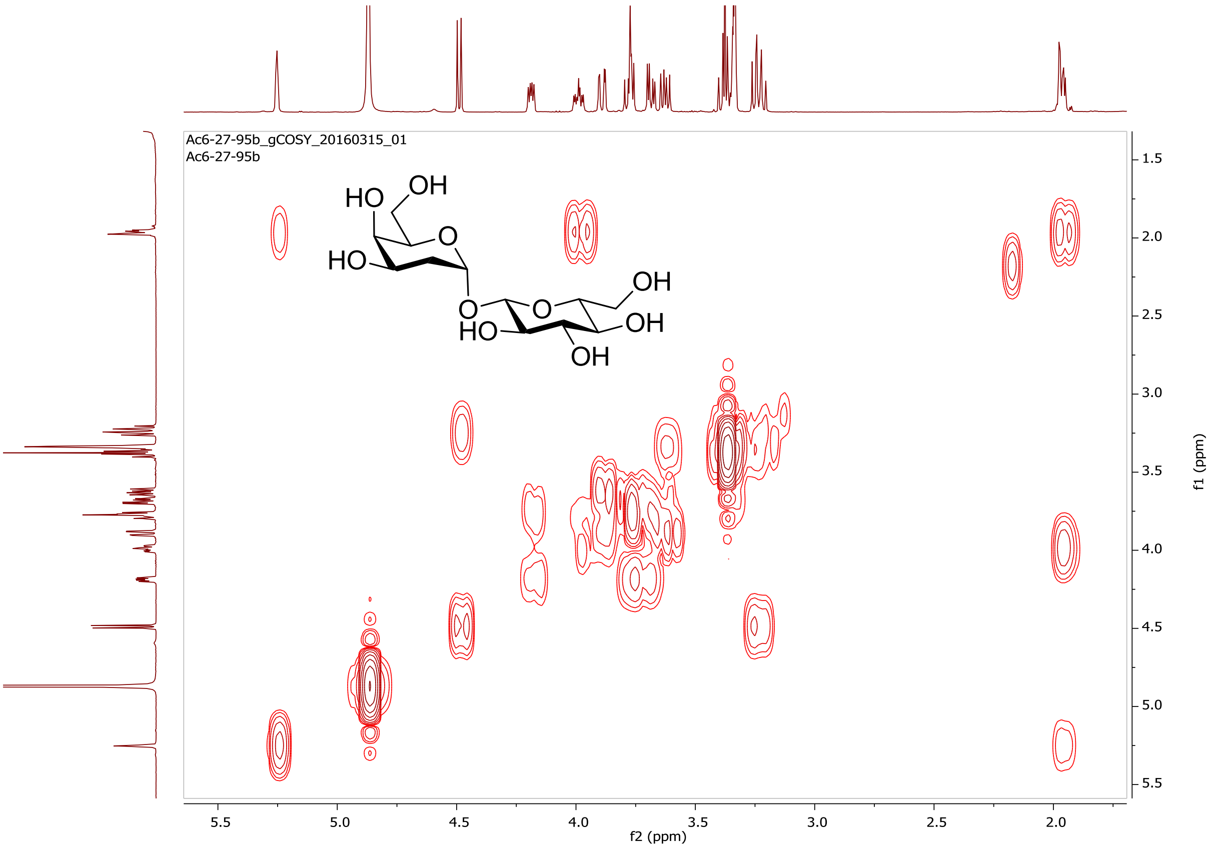

gHSQC (500 × 126 MHz, CD_3OD), β-D-glucopyranosyl-(1→1')-α-D-lyxo-hexapyranoside α,β-11b

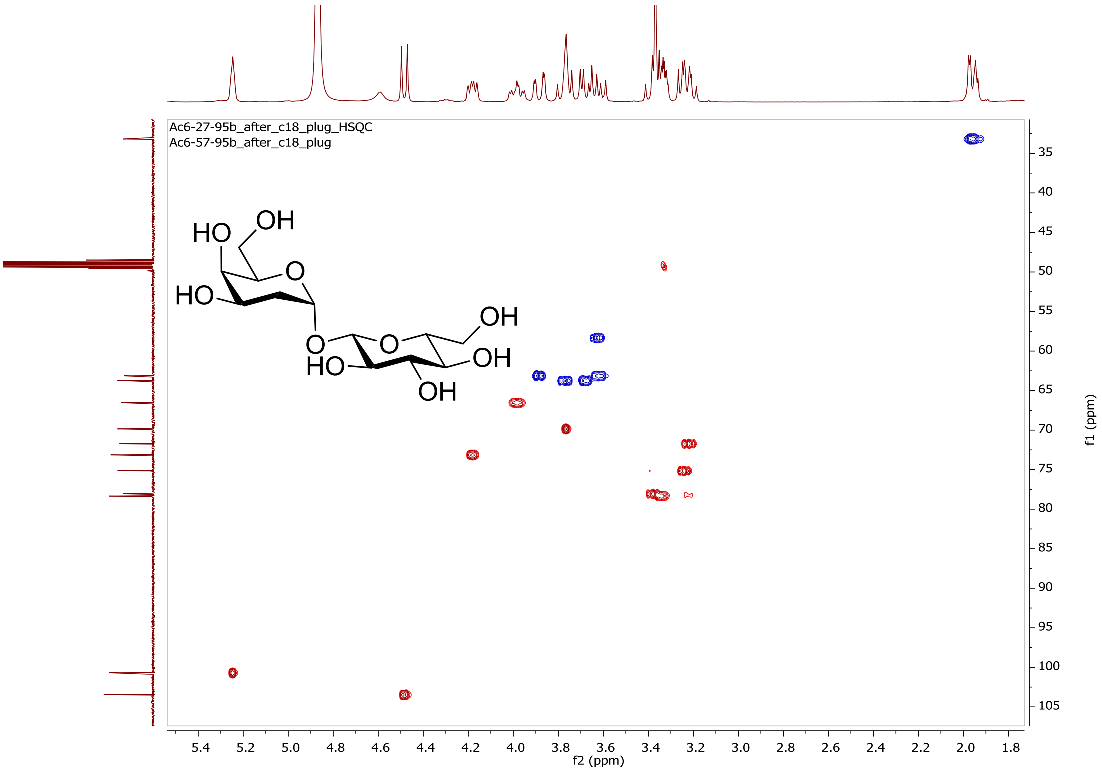

gHMBC (500 × 126 MHz, CD_3OD), β-D-glucopyranosyl-(1→1')-α-D-lyxo-hexapyranoside α,β-11b

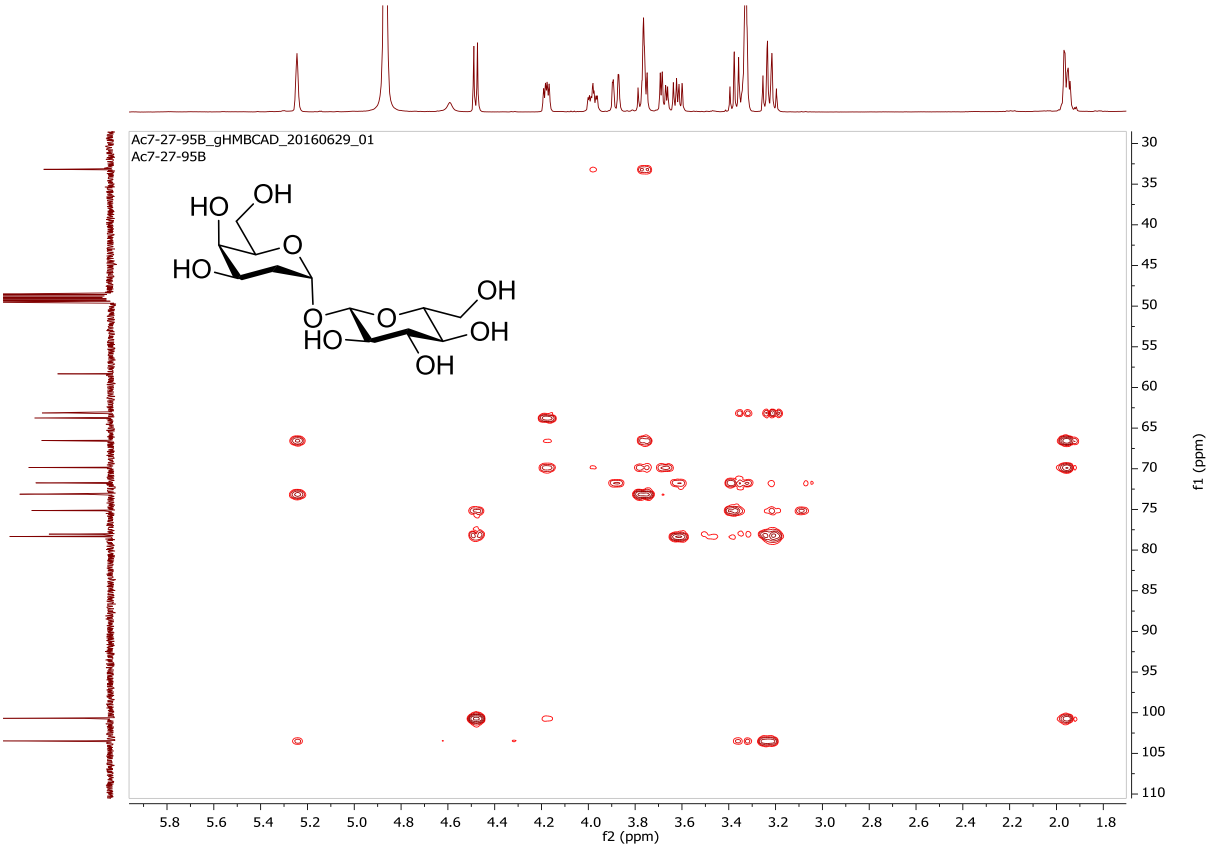

NMR Spectra relating to mechanistic experiments

^{1}H NMR (400 MHz, $CDCl_3$), monothiophthalimide 13

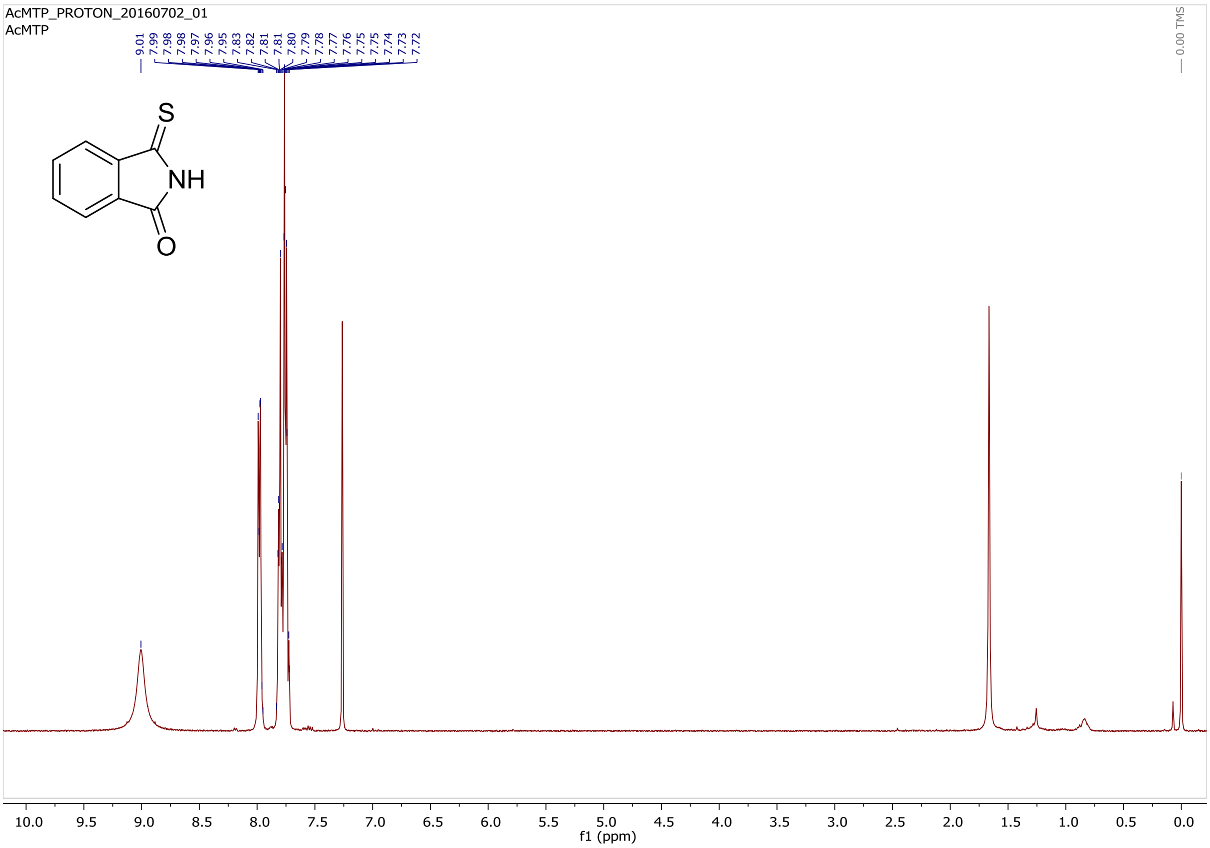

^{13}C NMR (101 MHz, $CDCl_3$), monothiophthalimide 13

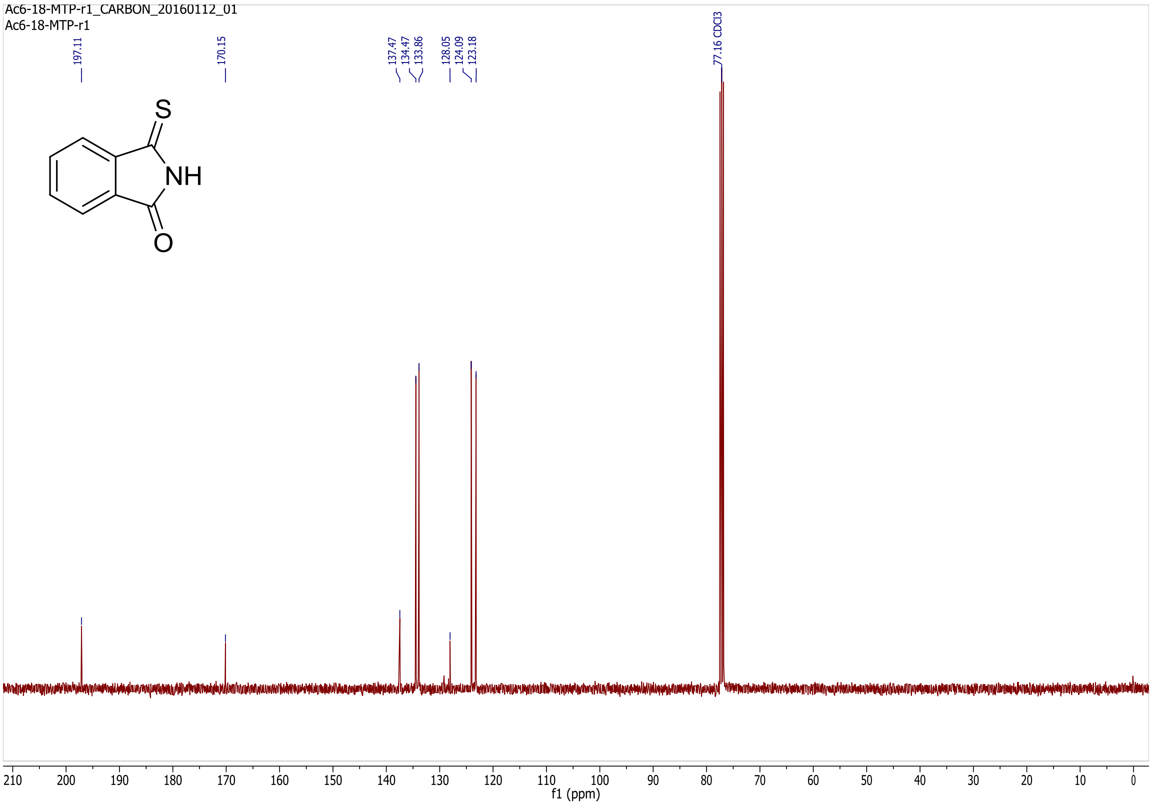

^{1}H NMR (400 MHz, $CDCl_3$), dithiophthalimide 20

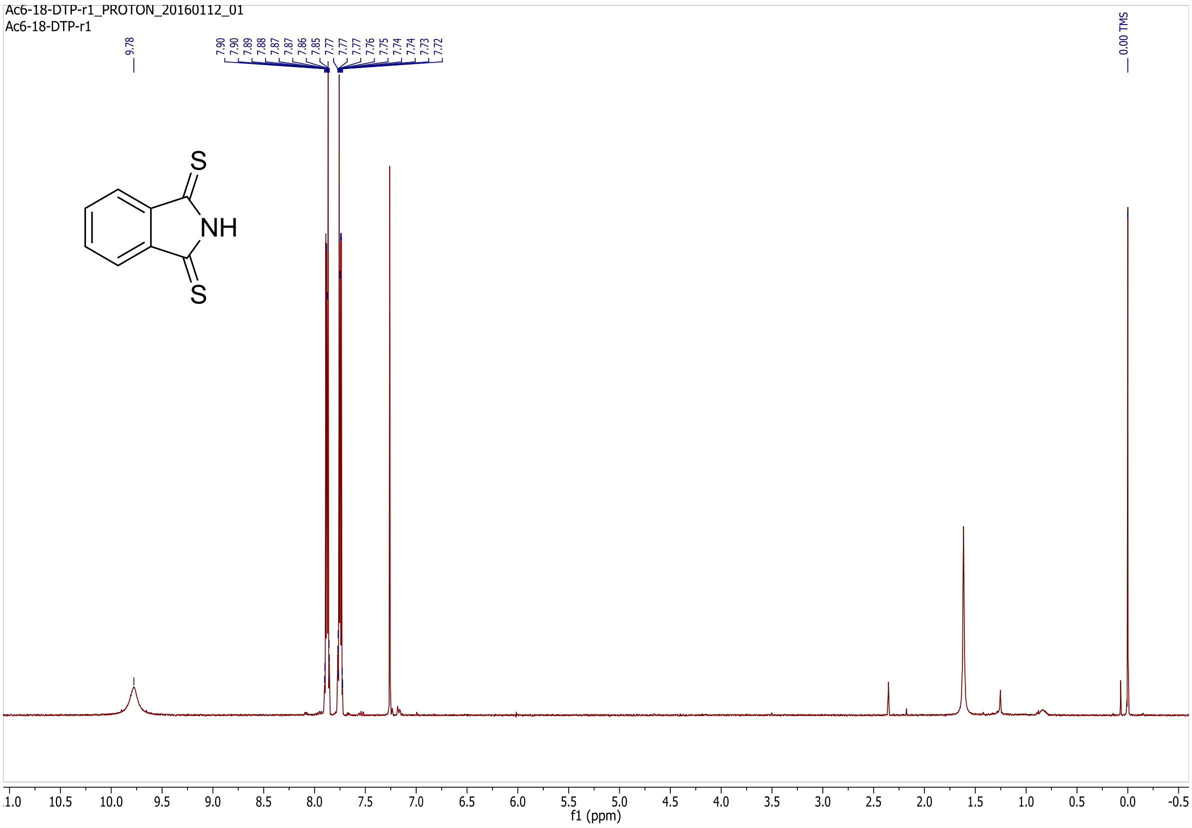

^{13}C NMR (101 MHz, $CDCl_3$), dithiophthalimide 20

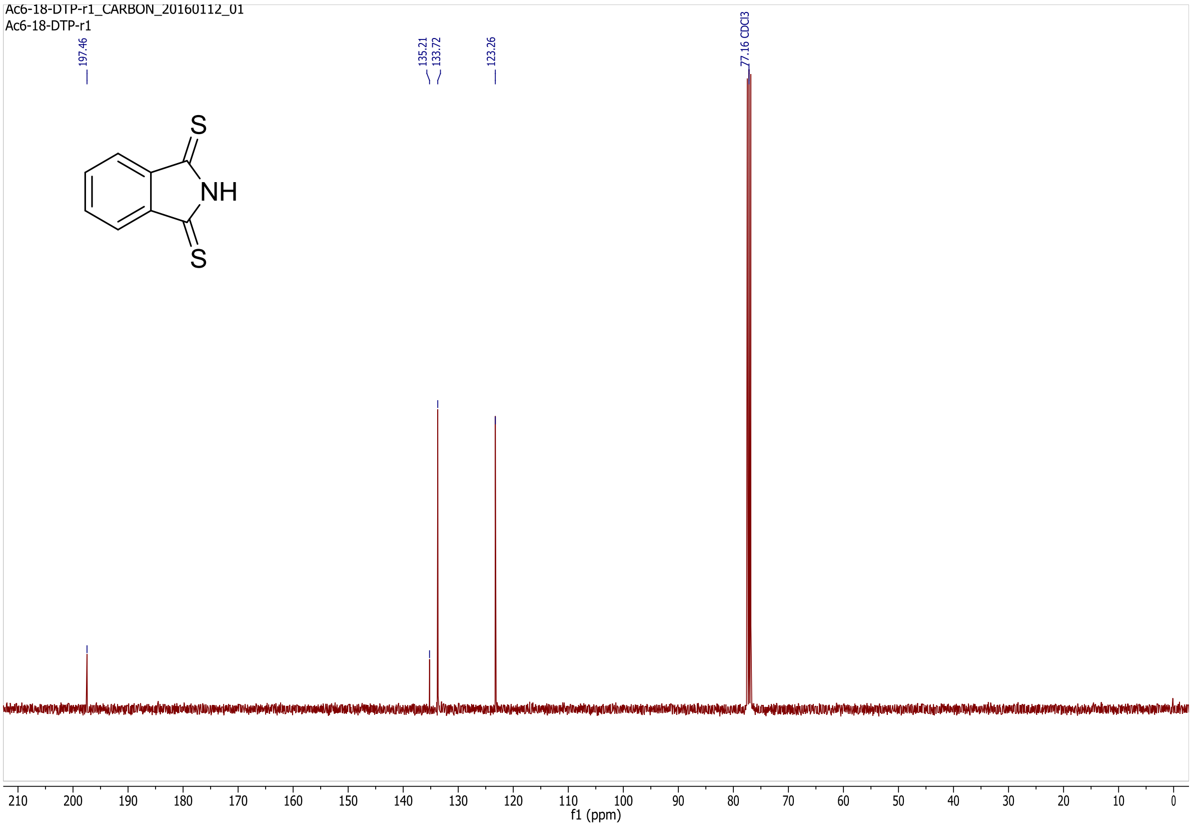

Control Experiments

^{1}H NMR (500 MHz, $CDCl_3$), α/β mixture of 6-*O*-(3,4,6-tri-*O*-benzyl-D-lyxo-hexapyranosyl)-1,2:3,4-di-*O*-isopropylidene-α-D-galactopyranose 5a

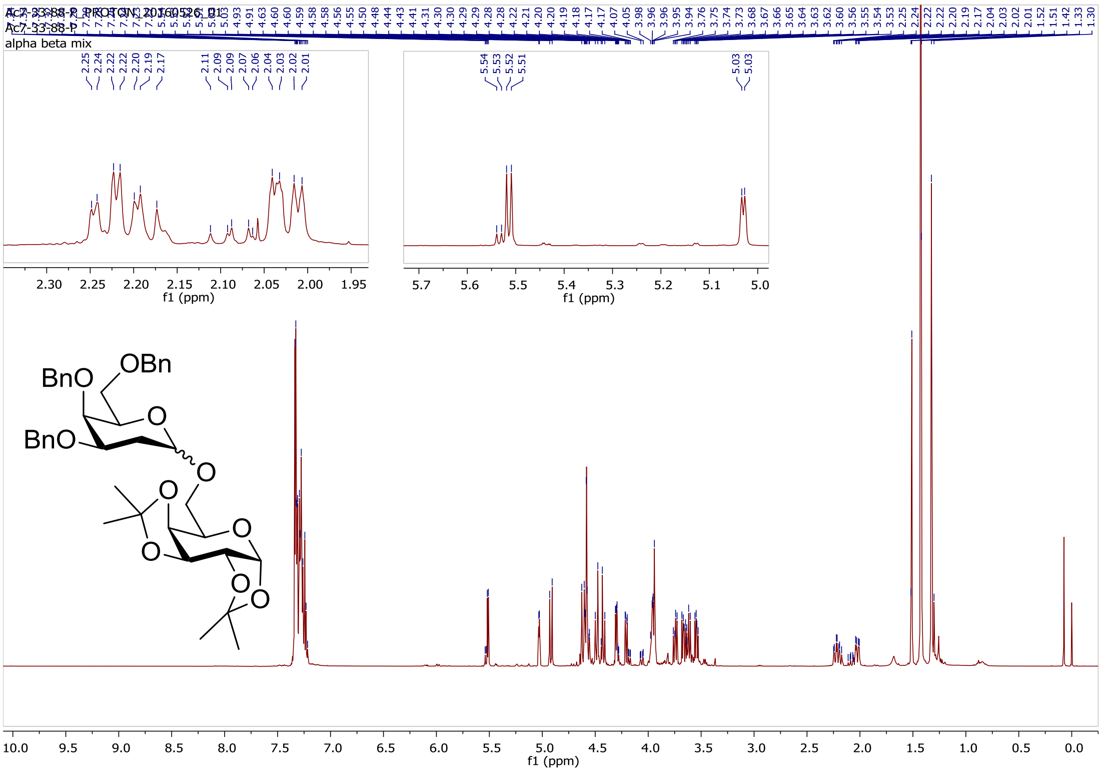

^{13}C NMR (126 MHz, $CDCl_3$), α/β mixture of 6-*O*-(3,4,6-tri-*O*-benzyl-D-lyxo-hexapyranosyl)-1,2:3,4-di-*O*-isopropylidene-α-D-galactopyranose 5a

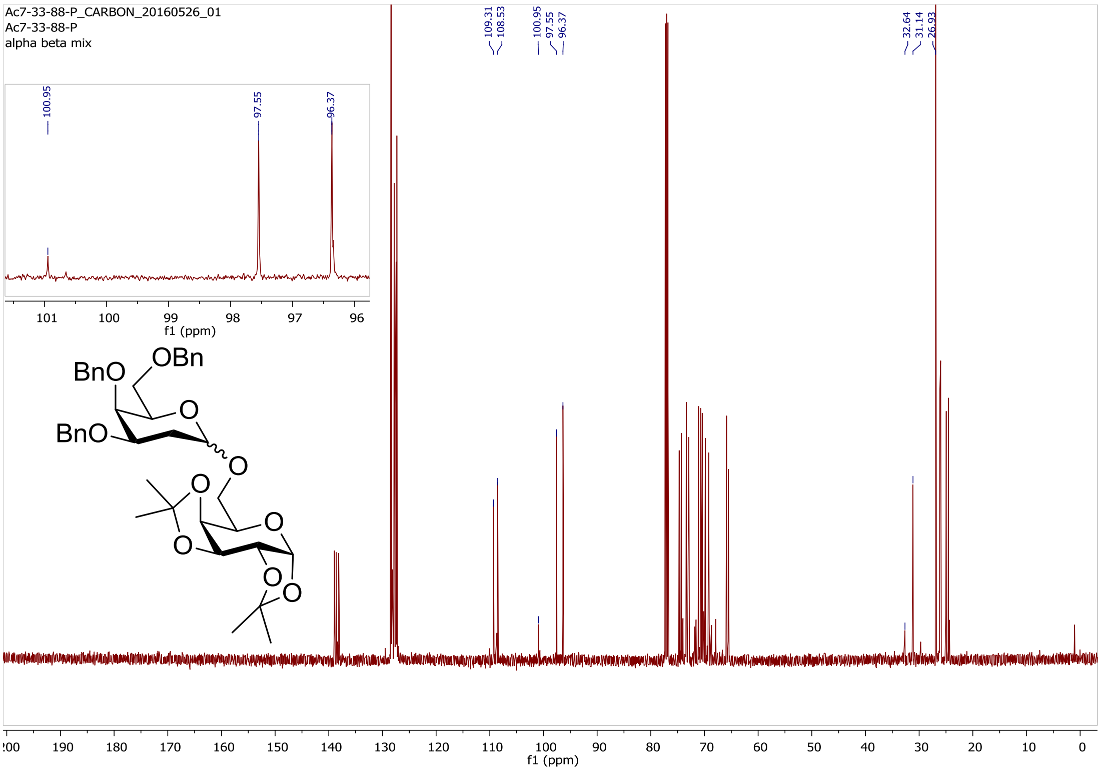

Cross-over experiment

^{1}H NMR (400 MHz, CDCl₃), α/β mixture of 6-*O*-(3,4,6-tri-*O*-benzyl-D-lyxo-hexapyranosyl)-1,2:3,4-di-*O*-isopropylidene-α-D-galactopyranose subjected to reaction conditions with phenyl 2,3,4-tri-O-benzyl-β-D-thioglucopyranose

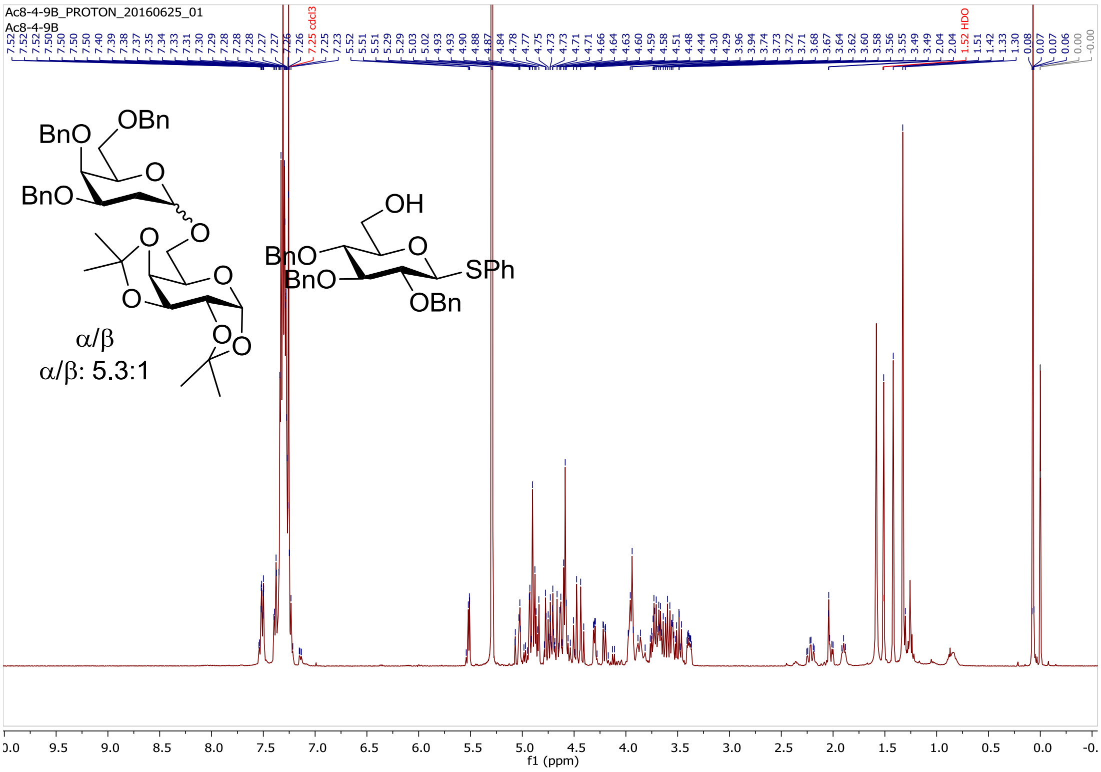

gHSQC (400× 100 MHz, CDCl₃) α/β mixture of 6-*O*-(3,4,6-tri-*O*-benzyl-D-lyxo-hexapyranosyl)-1,2:3,4-di-*O*-isopropylidene-α-D-galactopyranose subjected to reaction conditions with phenyl 2,3,4-tri-*O*-benzyl-β-D-thioglucopyranose:

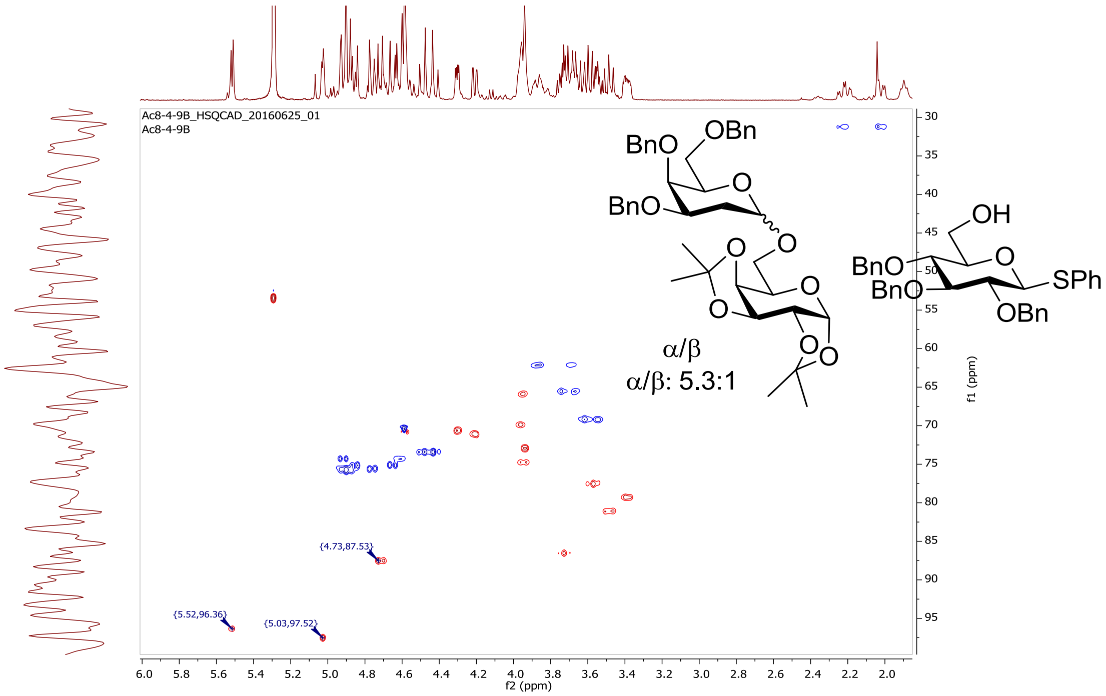

Catalyst poison experiment

^{1}H NMR (500 MHz, $CDCl_3$), α/β mixture of 6-*O*-(3,4,6-tri-*O*-benzyl-D-lyxo-hexapyranosyl)-1,2:3,4-di-*O*-isopropylidene-α-D-galactopyranose subjected to reaction conditions with phenyl 2,3,4-tri-*O*-benzyl-β-D-thioglucopyranose and 6-*O*-acetyl-3,4-di-*O*-benzyl-D-lyxo-hexapyranose

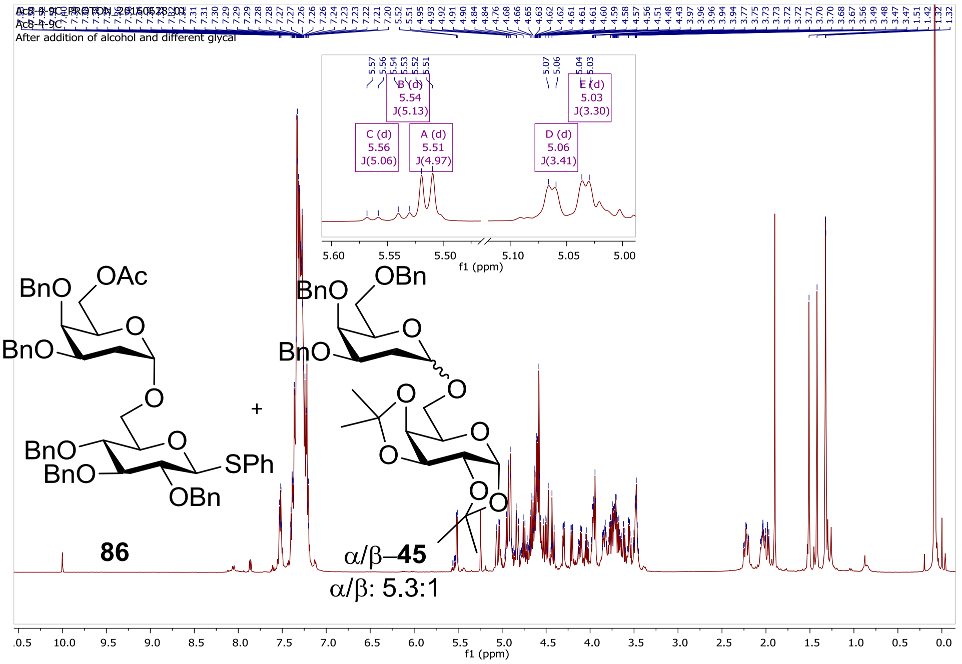

gHSQC (500 × 125 MHz, $CDCl_3$), α/β mixture of 6-*O*-(3,4,6-tri-*O*-benzyl-D-lyxo-hexapyranosyl)-1,2:3,4-di-*O*-isopropylidene-α-D-galactopyranose subjected to reaction conditions with phenyl 2,3,4-tri-*O*-benzyl-β-D-thioglucopyranose and 6-*O*-acetyl-3,4-di-*O*-benzyl-D-lyxo-hexapyranose

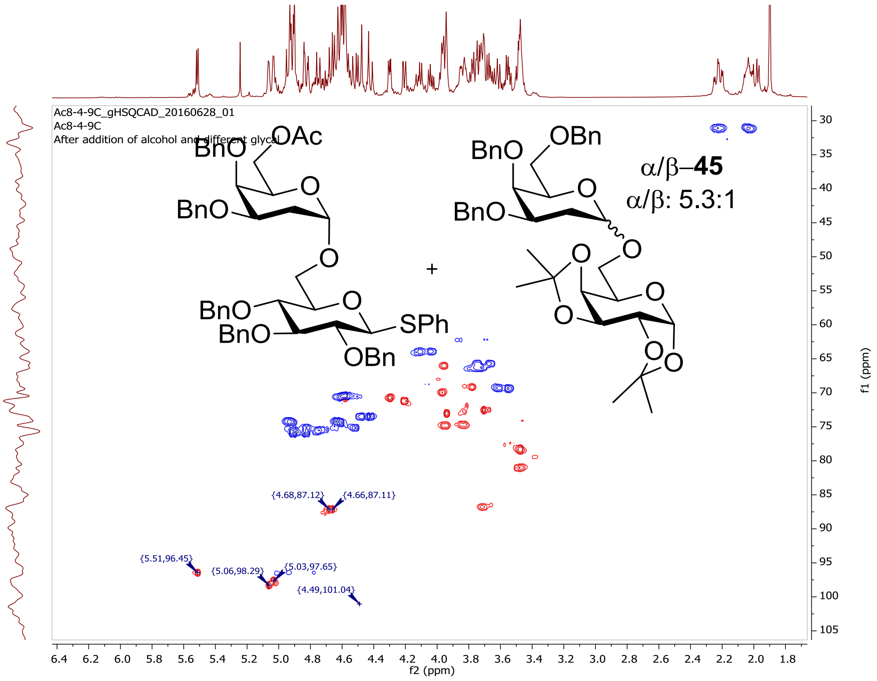

^{1}H NMR (500 MHz, CDCl$_3$), methyl 3,4,6-tri-*O*-benzyl-α-D-lyxo-hexapyranoside 19

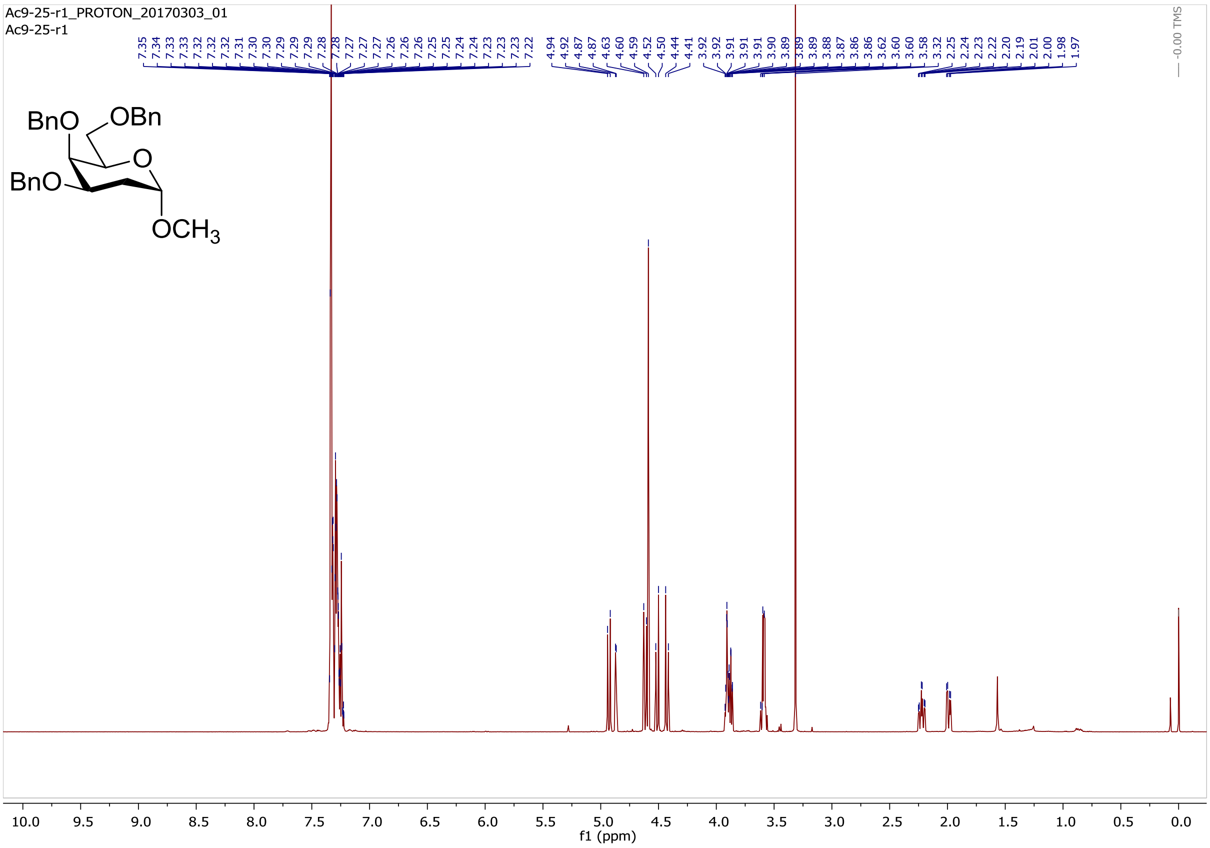

^{13}C NMR (126 MHz, CDCl$_3$), methyl 3,4,6-tri-*O*-benzyl-α-D-lyxo-hexapyranoside 19

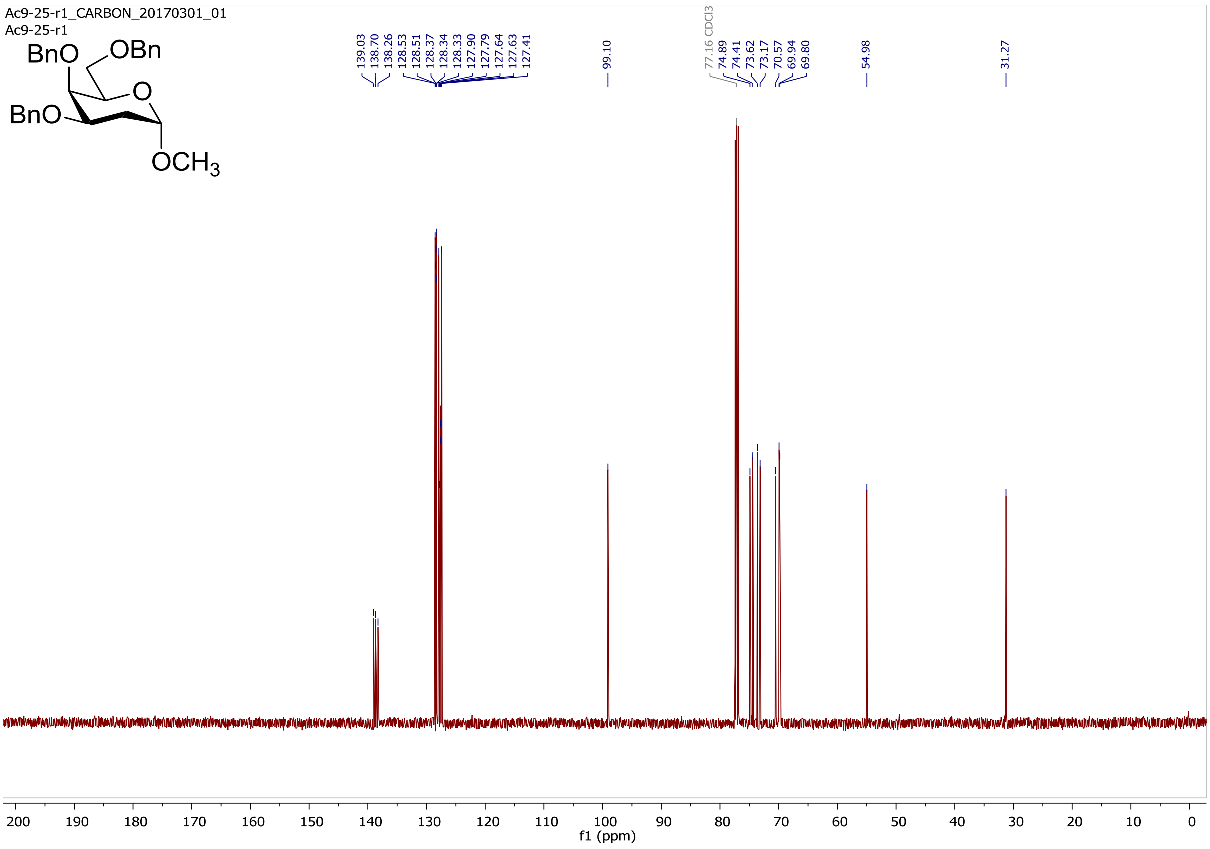

^{1}H NMR (500 MHz, CDCl$_3$), methyl (OCD$_3$) 3,4,6-tri-*O*-benzyl-(2-^{2}H)-α-D-lyxo-hexapyranoside 19

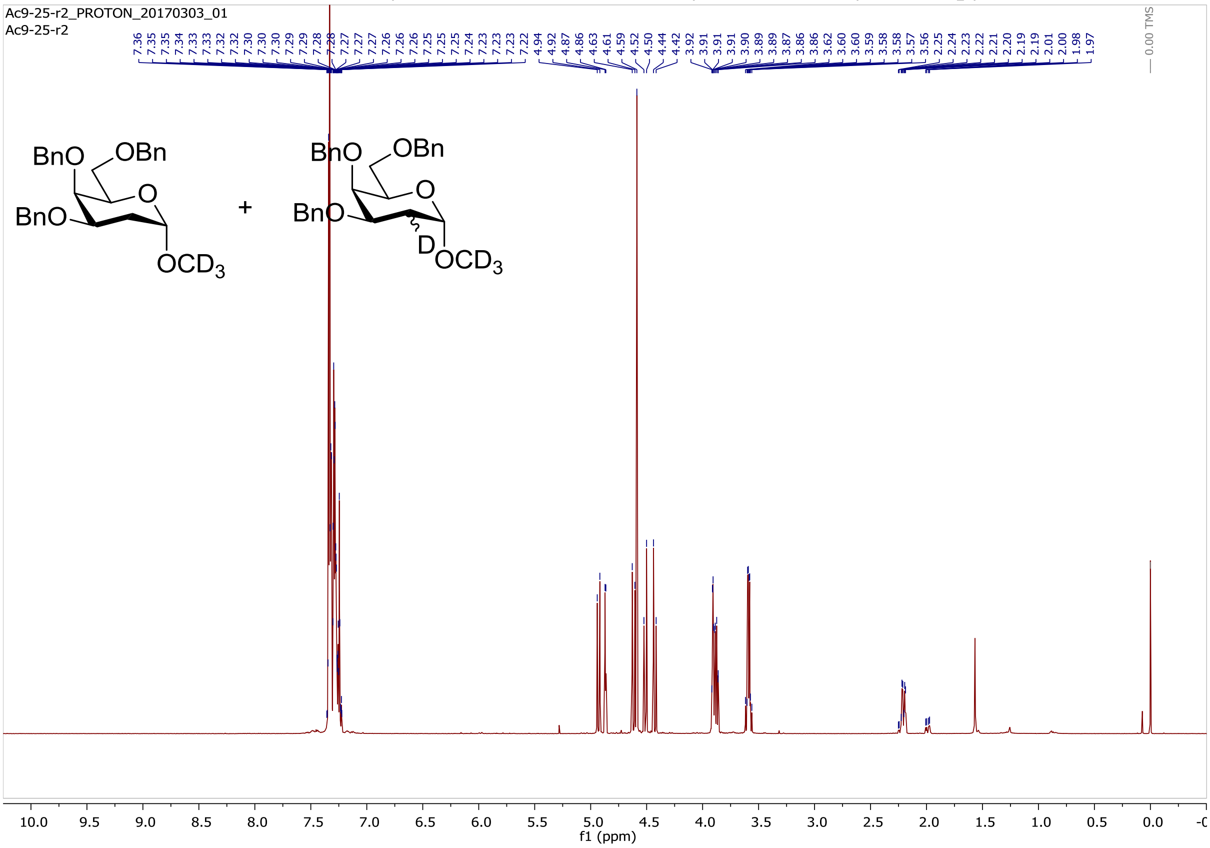

^{13}C NMR (126 MHz, CDCl$_3$), methyl (OCD$_3$) 3,4,6-tri-*O*-benzyl-(2-^{2}H)-α-D-lyxo-hexapyranoside 19

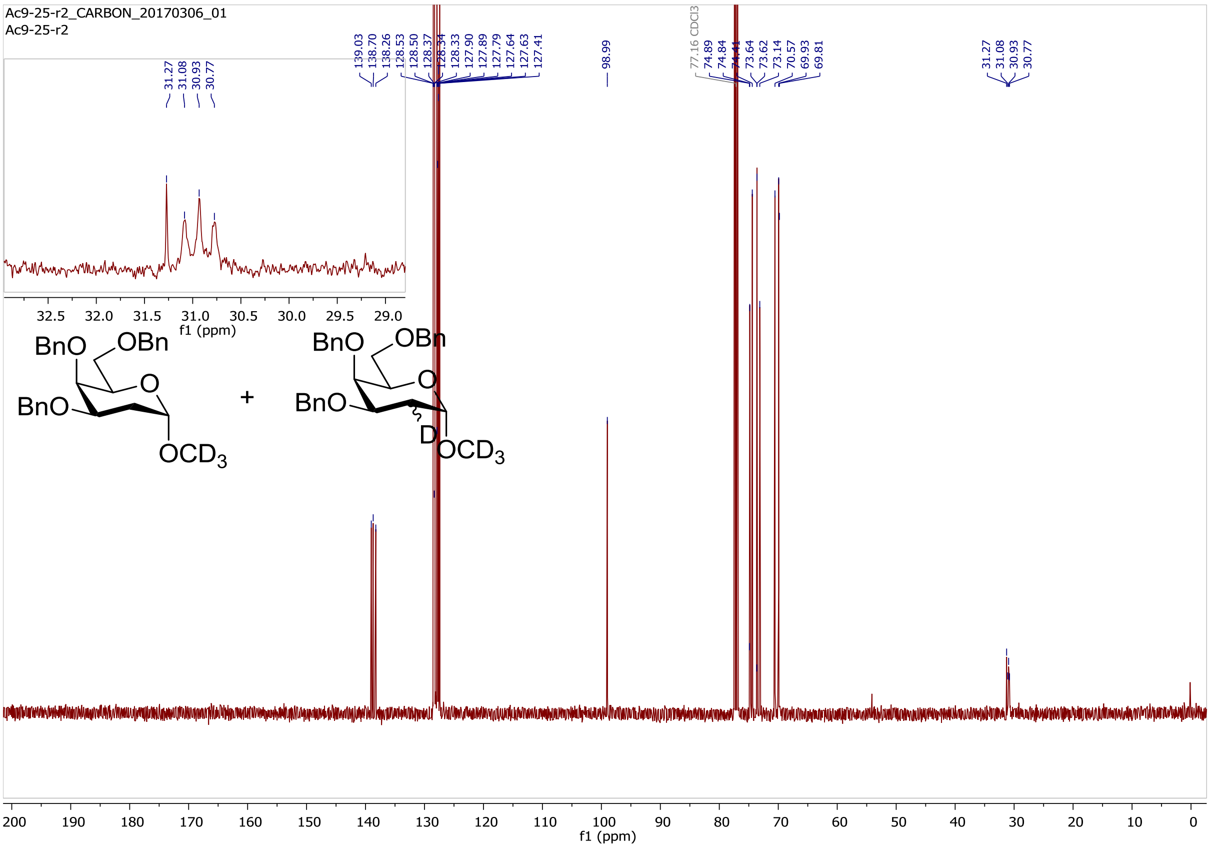

HSQC NMR (500 × 126 MHz, CDCl$_3$), methyl (OCD$_3$) 3,4,6-tri-*O*-benzyl-(2-^{2}H)-α-D-lyxo-hexapyranoside 19

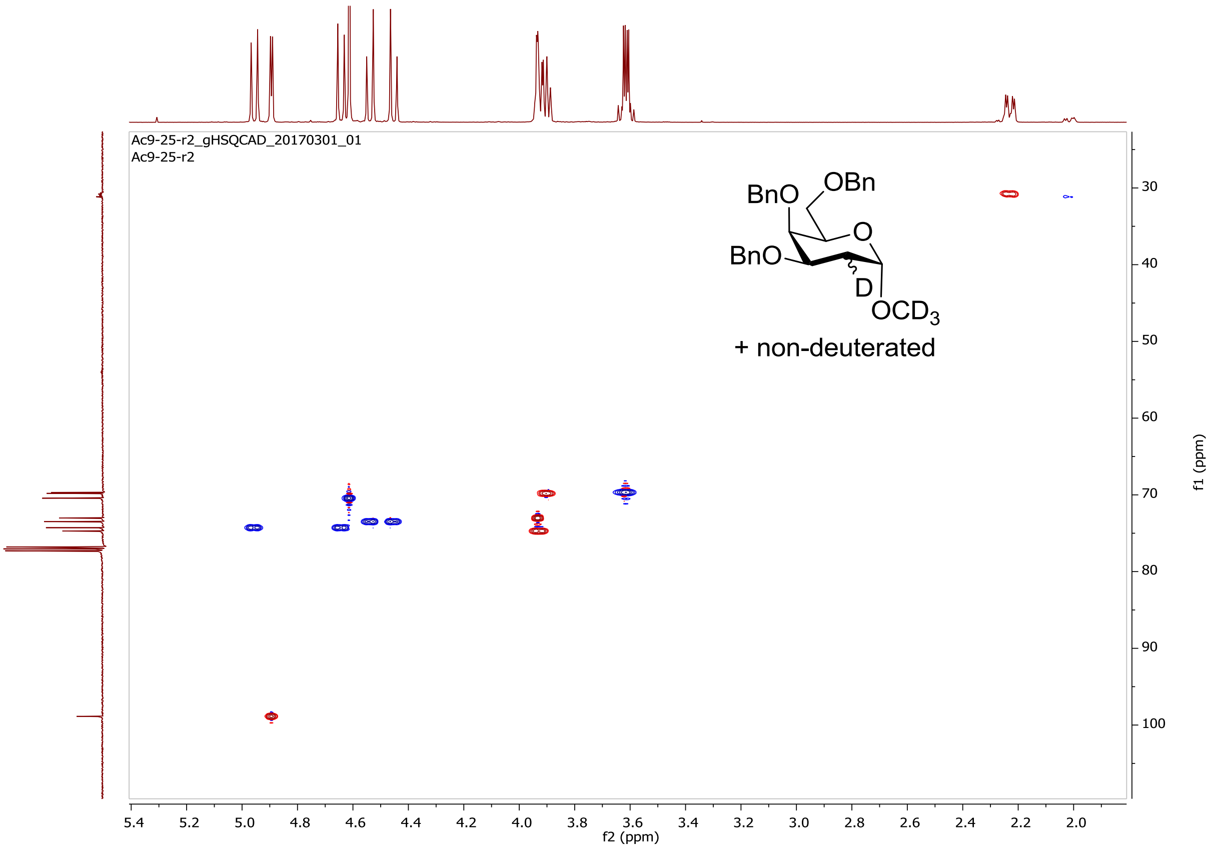

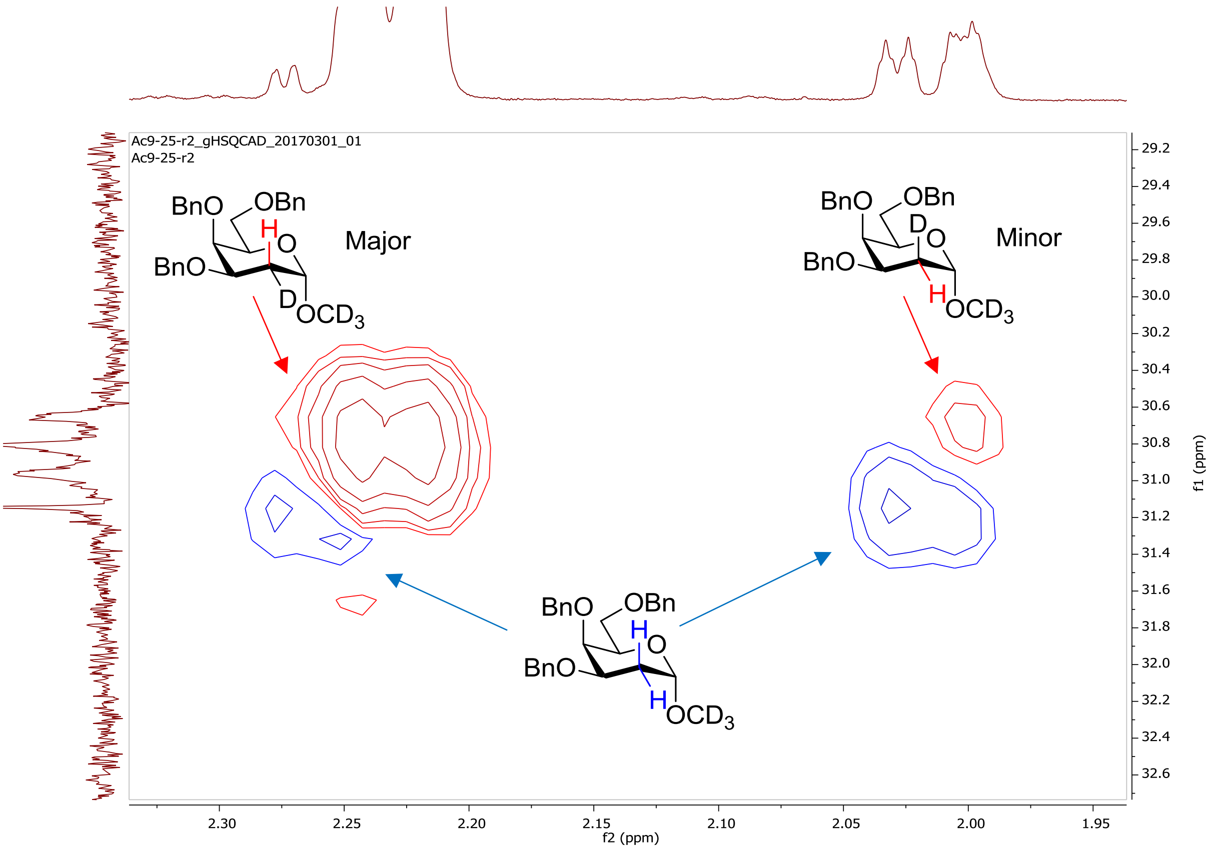

COSY NMR (500 × 500 MHz, $CDCl_3$), methyl (OCD_3) 3,4,6-tri-*O*-benzyl-(2-^{2}H)-α-D-lyxo-hexapyranoside 19

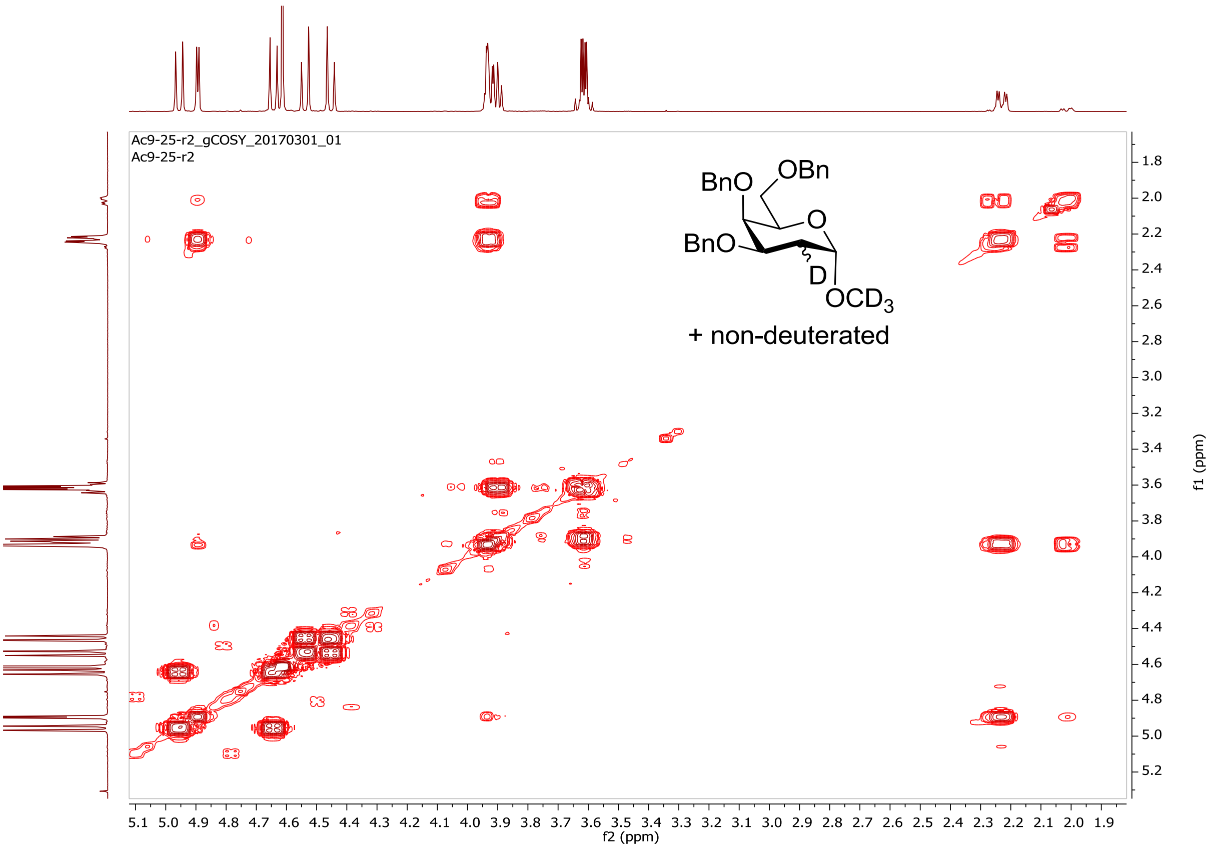

^{1}H NMR (500 MHz; $CDCl_3$), 3,4,6-tri-*O*-benzyl-(2-^{2}H)-α-D-*lyxo*-hexapyranosyl-(1→1')-3',4',6'-tri-*O*-benzyl-(2-^{2}H)-α-D-*lyxo*-hexapyranoside; 9a

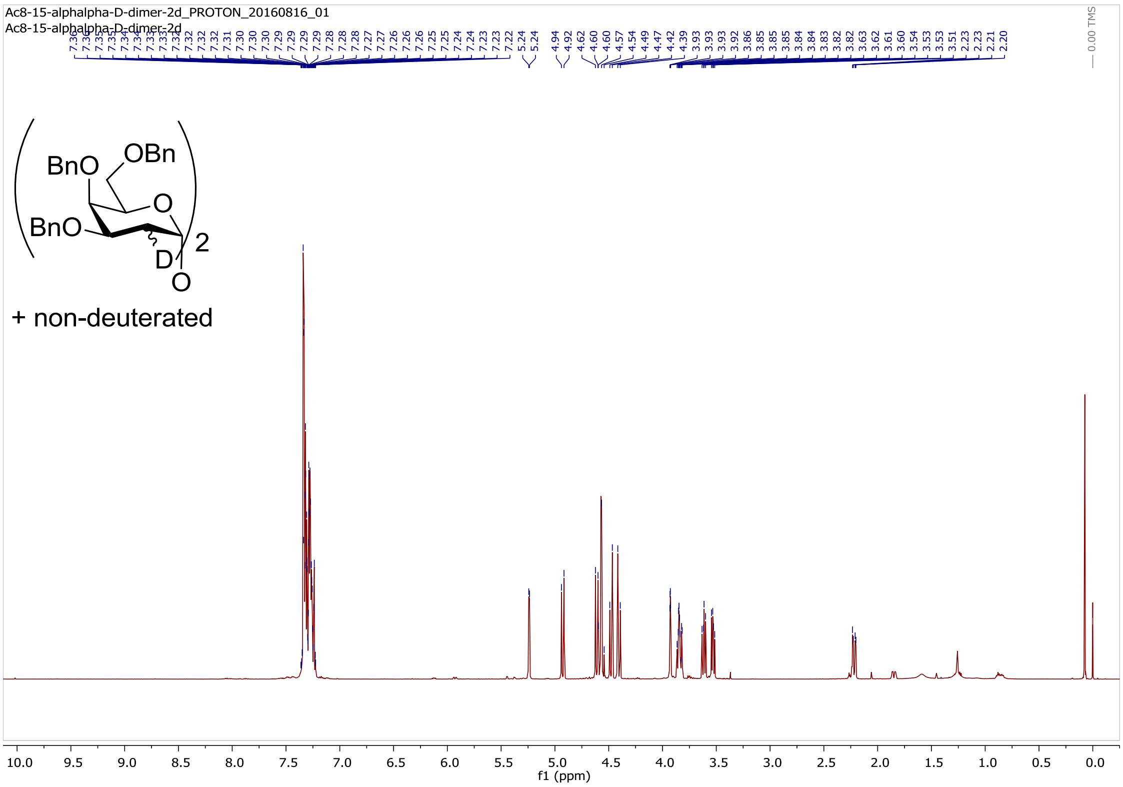

^{13}C NMR (126 MHz; $CDCl_3$), 3,4,6-tri-*O*-benzyl-(2-^{2}H)-α-D-*lyxo*-hexapyranosyl-(1→1')-3',4',6'-tri-*O*-benzyl-(2-^{2}H)-α-D-*lyxo*-hexapyranoside 9a

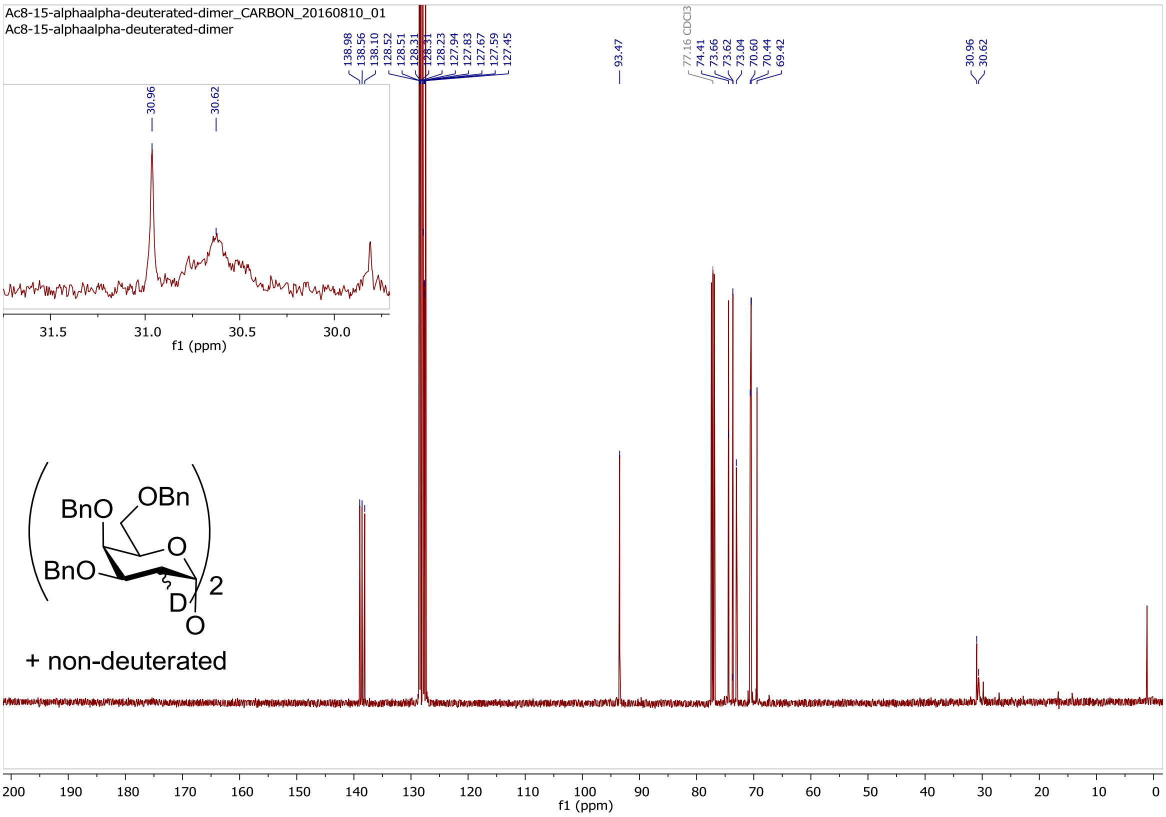

HSQC NMR (500 × 126 MHz, CDCl$_3$), 3,4,6-tri-*O*-benzyl-(2-^{2}H)-α-D-*lyxo*-hexapyranosyl-(1→1')-3',4',6'-tri-*O*-benzyl-(2-^{2}H)-α-D-*lyxo*-hexapyranoside 9a

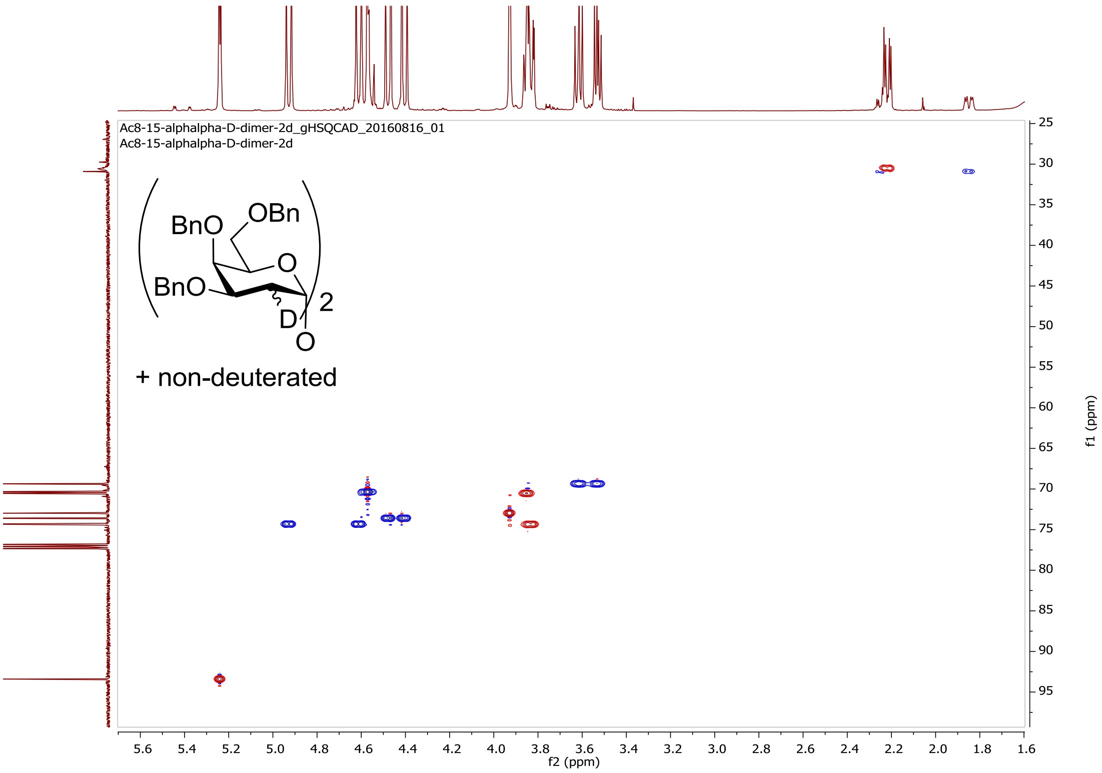

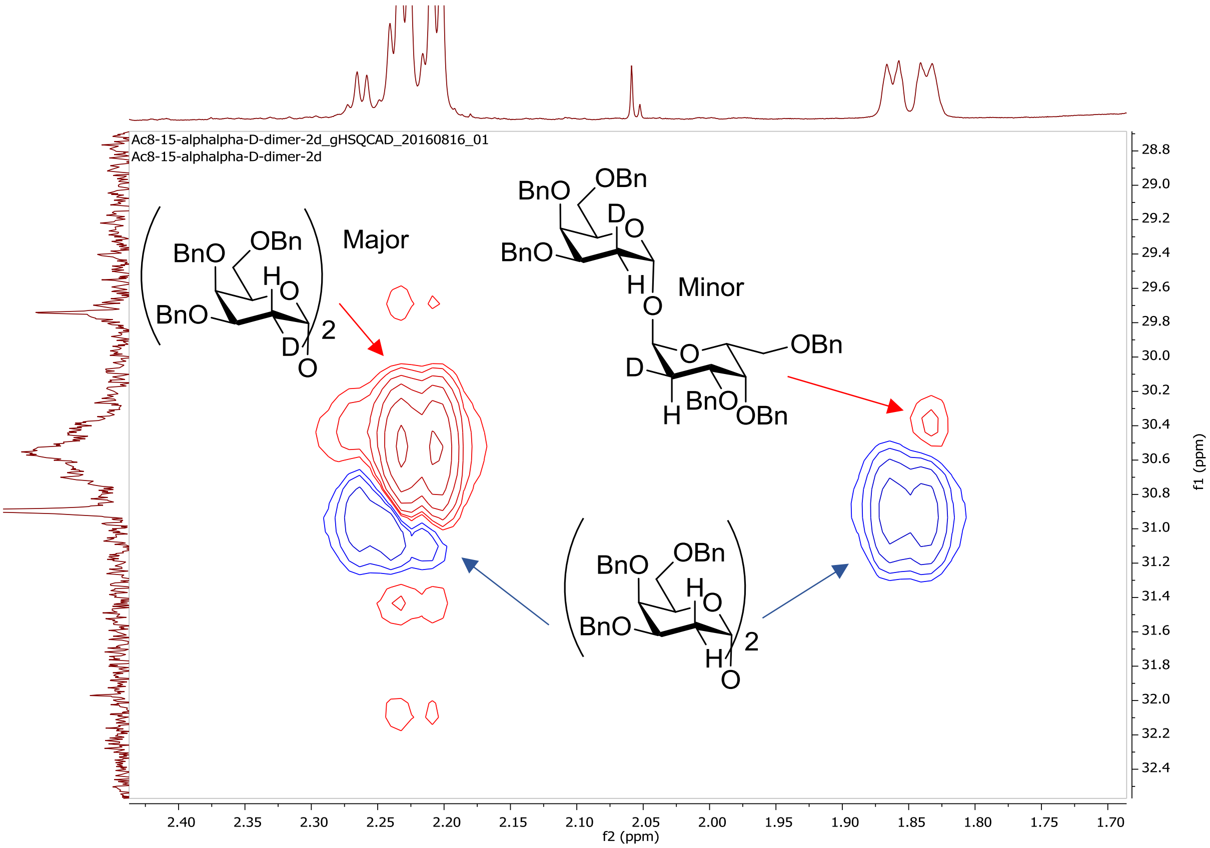

HMBC NMR (500 × 126 MHz, $CDCl_3$), 3,4,6-tri-*O*-benzyl-(2-^{2}H)-α-D-*lyxo*-hexapyranosyl-(1→1')-3',4',6'-tri-*O*-benzyl-(2-^{2}H)-α-D-*lyxo*-hexapyranoside 9a

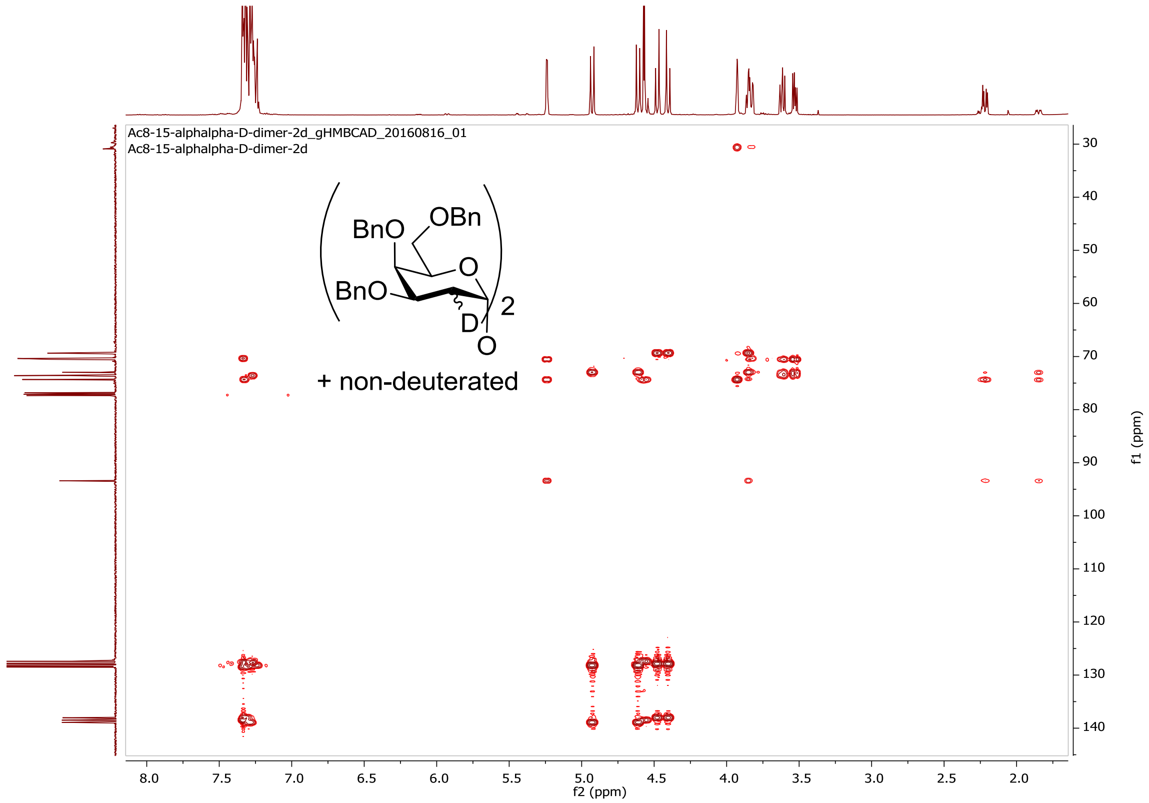

COSY NMR (500 × 500 MHz, $CDCl_3$), 3,4,6-tri-*O*-benzyl-(2-^{2}H)-α-D-*lyxo*-hexapyranosyl-(1→1')-3',4',6'-tri-*O*-benzyl-(2-^{2}H)-α-D-*lyxo*-hexapyranoside 9a

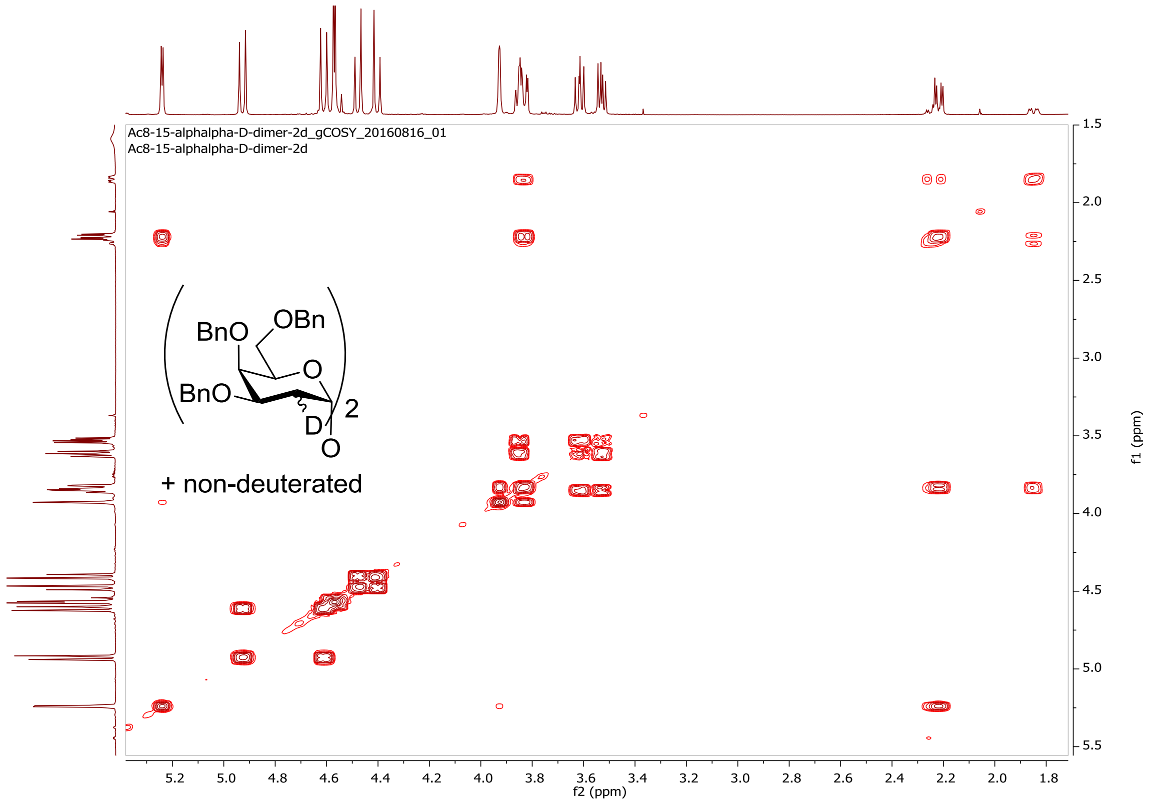

Supplement: Supplementary file 1 [file SC-010-C8SC02788A-s001.pdf]
